# Deep mining of oxysterols and cholestenoic acids in human plasma and cerebrospinal fluid: Quantification using isotope dilution mass spectrometry

**DOI:** 10.1016/j.aca.2021.338259

**Published:** 2021-04-15

**Authors:** Eylan Yutuc, Alison L. Dickson, Manuela Pacciarini, Lauren Griffiths, Paul R.S. Baker, Lisa Connell, Anders Öhman, Lars Forsgren, Miles Trupp, Sílvia Vilarinho, Youssef Khalil, Peter T. Clayton, Sinan Sari, Buket Dalgic, Philip Höflinger, Ludger Schöls, William J. Griffiths, Yuqin Wang

**Affiliations:** a Swansea University Medical School, ILS1 Building, Singleton Park, Swansea, SA2 8PP, Wales, UK; b Avanti Polar Lipids Inc, Alabaster, AL, USA; c Department of Integrative Medical Biology, Umeå University, SE-901 87, Umeå, Sweden; d Department of Clinical Science, Neurosciences, Umeå University, SE-901 85, Umeå, Sweden; e Departments of Internal Medicine, Section of Digestive Diseases, and of Pathology, Yale University School of Medicine, New Haven, CT, USA; f Inborn Errors of Metabolism, Genetics and Genomic Medicine, UCL Great Ormond Street Institute of Child Health, 30 Guilford Street, London, WC1N 1EH, UK; g Department of Pediatrics, Division of Gastroenterology, Gazi University Faculty of Medicine, Ankara, Turkey; h Department of Neurology and Hertie Institute for Clinical Brain Research, University of Tübingen, Tübingen, Germany; i German Center for Neurodegenerative Diseases (DZNE), Tübingen, Germany

**Keywords:** Cholesterol, Hydroxycholesterol, Cholestenoic acid, Bile acid, LC-MS, Derivatisation, Isotope-labelled standard

## Abstract

Both plasma and cerebrospinal fluid (CSF) are rich in cholesterol and its metabolites. Here we describe in detail a methodology for the identification and quantification of multiple sterols including oxysterols and sterol-acids found in these fluids. The method is translatable to any laboratory with access to liquid chromatography – tandem mass spectrometry. The method exploits isotope-dilution mass spectrometry for absolute quantification of target metabolites. The method is applicable for semi-quantification of other sterols for which isotope labelled surrogates are not available and approximate quantification of partially identified sterols. Values are reported for non-esterified sterols in the absence of saponification and total sterols following saponification. In this way absolute quantification data is reported for 17 sterols in the NIST SRM 1950 plasma along with semi-quantitative data for 8 additional sterols and approximate quantification for one further sterol. In a pooled (CSF) sample used for internal quality control, absolute quantification was performed on 10 sterols, semi-quantification on 9 sterols and approximate quantification on a further three partially identified sterols. The value of the method is illustrated by confirming the sterol phenotype of a patient suffering from ACOX2 deficiency, a rare disorder of bile acid biosynthesis, and in a plasma sample from a patient suffering from cerebrotendinous xanthomatosis, where cholesterol 27-hydroxylase is deficient.

## Highlights

• Absolute quantification of oxysterols and cholestenoic acids.
• Methodology applicable to plasma and cerebrospinal fluid.
• Data generated for non-esterified and total sterols.
• Diastereoisomers at C-24 and C-25 separated and quantified.

## Graphical abstract

**Figure:**
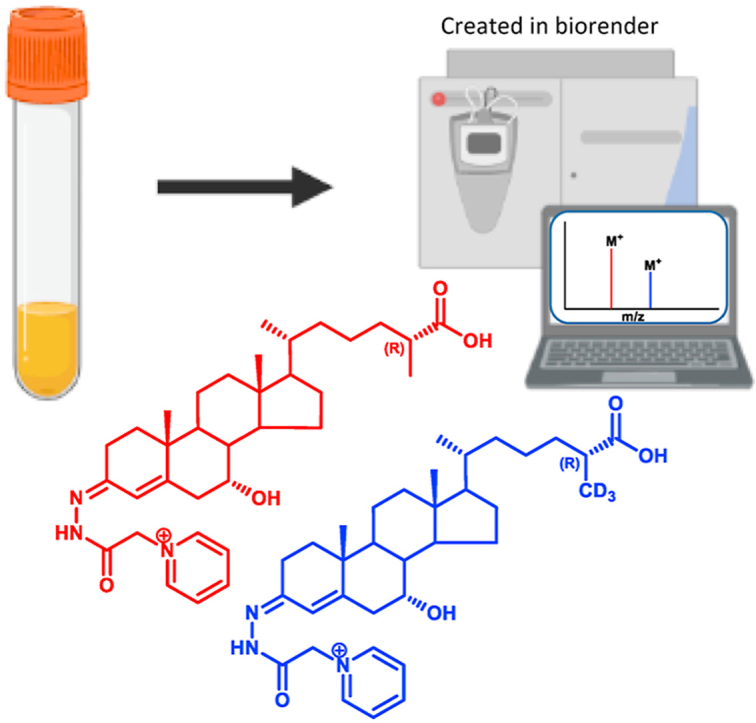

## 1 Introduction

Plasma/serum and cerebrospinal fluid (CSF) represent body fluids widely studied with an ultimate goal of revealing biomarkers of disease [[1], [2], [3], [4], [5], [6], [7]]. Plasma/serum analysis by mass spectrometry (MS) can prove particularly fruitful to reveal inborn errors of metabolism, especially those related to cholesterol biosynthesis and metabolism [[8], [9], [10], [11], [12]], while analysis of CSF may have value to monitor neurodegeneration [7,[13], [14], [15]]. However, comparing data across different laboratories can prove treacherous as a consequence of multiple different platforms and methods used, and differences in the use of standards for quantification [4,16,17].

Isotope-dilution (ID)-MS represents the most reliable methodology for the quantitative measurement of lipids, including sterols, in biological samples [18,19]. Despite this, large differences in inter-laboratory measurements may still occur even when using ID-MS [16,17]. These differences, should, however, be minimised by the use of common isotope-labelled standards accurately prepared in a suitable solvent for distribution to laboratories world-wide. In the era of “omic” science, there is a drive for the quantification of multiple analytes in a single sample and this has led to the development of commercial mixtures of accurately aliquoted combinations of different isotope-labelled standards to allow the quantification of multiple lipids in a single analysis [1,20]. At the more targeted level, a commercial kit containing a mixture of twenty different isotope-labelled bile acids is now available [21]. A second challenge for the inter-laboratory comparison of quantitative data is provided by the variation in the exact nature of the samples analysed and compared. This problem can be overcome by the use of well documented Standard Reference Materials (SRMs).

Here, we report the absolute quantification of 17 sterols, including oxysterols and cholestenoic acids in an SRM plasma sample (NIST SRM 1950 [22,23]) using isotope-labelled cholesterol and a recently commercialised mixture of other isotope-labelled sterols. We have quantified the oxysterols as non-esterified free molecules and, where possible, following saponification of esters. In addition, the mixture of isotope-labelled standards has been used to for the semi-quantification of 8 other sterols including oxysterols and sterol-acids in plasma where authentic, but not isotope-labelled standards, were available. Approximate quantification of one further sterol was made in the absence of an available authentic standard. Seven other sterols were identified but not quantified, while 8 further sterols were partially identified in the absence of authentic standards and were not quantified. Note, here we explicitly use the terms: *absolute quantification* to define quantification performed against an isotope-labelled surrogate of otherwise *exactly the same structure*, e.g. (25R)26-hydroxycholesterol [(25R)26-HC] against [25,26,26,27,27,27-^2^H_6_](25R)26-HC; *semi-quantification* to define quantification against an isotope labelled surrogate *of similar but not identical structure*, e.g. 3β,7β-dihydroxycholest-5-en-26-oic acid (3β,7β-diHCA) against [27,27,27-^2^H_3_]3β,7α-dihydroxycholest-5-en-26-oic acid ([^2^H_3_]3β,7α-diHCA); and *approximate quantification* to define quantification against an isotope labelled surrogate, but in the *absence of an authentic standard* of the sterol to be quantified i.e. 7α-hydroxy-27-*nor*cholest-4-ene-3,24-dione (7αH,27-nor-C-3,24-diO) against [^2^H_6_](25R)26-HC. The equivalent numbers of quantified/identified sterols in an internal quality control (QC) CSF sample were: absolute quantification of 10 sterols, semi-quantification of 9 sterols and approximate quantification of 3 sterols. In addition, 5 other sterols were presumptively identified in the absence of authentic standards but not quantified. It should be noted, that besides the sterols reported here in the SRM plasma and QC CSF, a very large number of additional oxysterols and sterol-acids have been detected in samples from patients suffering from inborn errors of sterol metabolism, which are quantitatively minor in samples from healthy individuals [[24], [25], [26], [27], [28]]. We demonstrate the value of the analytical method employed by confirming the sterol phenotype of two such inborn errors of metabolism i.e. ACOX2 (acyl-CoA oxidase 2) deficiency and cerebrotendinous xanthomatosis (CTX), two rare disorder of bile acid biosynthesis [10,[29], [30], [31]]. These disorders highlight the value of the methodology to discriminate between diastereomers with asymmetric carbons at C-24 e.g. 24S-hydroxycholesterol (24S-HC) and 24R–HC, and at C-25 e.g. 7α-hydroxy-3-oxocholest-4-en-(25R)26-oic acid [7αH,3O-CA(25R)] and 7αH,3O-CA(25S). Note, Supplemental Table S1 provides a list of systematic names, common names and abbreviations.

## 2 Experimental

### 2.1 Materials

OxysterolSPLASH™, a recently commercialised mixture of oxysterols and cholestenoic acids was provided by Avanti Polar Lipids Inc (AL, USA). The mixture consists of the following isotope-labelled standards in methanol solvent; [25,26,26,26,27,27,27-^2^H_7_]24R/S-HC ([^2^H_7_]24R/S-HC, 80 ng/mL), [26,26,26,27,27,27-^2^H_6_]25-hydroxycholesterol ([^2^H_6_]25-HC, 10 ng/mL), [25,26,26,27,27,27-^2^H_6_](25R)26-HC ([^2^H_6_](25R)26-HC, also called [^2^H_6_]27-hydroxycholesterol, 160 ng/mL, note [^2^H_5_](25R)26-HC is also present at a level of about 15% of that of the [^2^H_6_]-isotopolouge), [25,26,26,26,27,27,27-^2^H_7_]7α-hydroxycholesterol ([^2^H_7_]7α-HC, 60 ng/mL), [25,26,26,26,27,27,27-^2^H_7_]7β-hydroxycholesterol ([^2^H_7_]7β-HC, 10 ng/mL), [25,26,26,26,27,27,27-^2^H_7_]7-oxocholesterol ([^2^H_7_]7-OC, 30 ng/mL), [25,26,26,26,27,27,27-^2^H_7_]7α-hydroxycholest-4-en-3-one ([^2^H_7_]7α-HCO, 20 ng/mL), [26,26,26,27,27,27-^2^H_6_]7α,25-dihydroxycholesterol ([^2^H_6_]7α,25-diHC, 1 ng/mL), [25,26,26,27,27,27-^2^H_6_]7α,(25R/S)26-dihydroxycholesterol ([^2^H_6_]7α,(25R/S)26-diHC, also called [^2^H_6_]7α,27-dihydroxycholesterol, 2 ng/mL), [27,27,27-^2^H_3_]7αH,3O-CA(25R/S) ([^2^H_3_]7αH,3O-CA(25R/S), 70 ng/mL), [25,26,26,26,27,27,27-^2^H_7_]4β-hydroxycholesterol ([^2^H_7_]4β-HC, 30 ng/mL), [25,26,26,26,27,27,27-^2^H_7_]22R-hydroxycholesterol ([^2^H_7_]22R–HC, 5 ng/mL) and [25,26,26,26,27,27,27-^2^H_7_]cholestane-3β,5α,6β-triol ([^2^H_7_]5α,6β-diHC, 10 ng/mL). Additional quantitative isotope labelled standards provided in methanol in exact quantities from Avanti Polar Lipids were [26,26,26,27,27,27-^2^H_6_]24R/S-HC (51.95 μg/mL, LM-4110), [^2^H_7_]7α-HC (48.74 μg/mL, LM-4103), [^2^H_7_]7-OC (51.42 μg/mL, LM-4107) and [25,26,26,26,27,27,27-^2^H_7_]cholesterol (526.01 μg/mL, LM-4100). The isotope labelled standards listed above were provided at defined concentrations by Avanti Polar Lipids and were used without further purification or validation. Certificate of analysis for OxysterolSPLASH and the other quantitative isotope labelled standards are available at https://avantilipids.com/. Other isotope-labelled standards, [25,26,26,26,27,27,27-^2^H_7_]22S-hydroxycholesterol ([^2^H_7_]22S-HC), [^2^H_7_]5α,6β-diHC and [^2^H_6_]7α,25-diHC and were also from Avanti Polar Lipids Inc. Additional non-labelled standards were from Avanti Polar Lipids or as indicated in Table S1. Sterols with a 3-oxo-4-ene structure were generated from 3β-hydroxy-5-ene analogues by treatment with cholesterol oxidase [26]. Cholesterol oxidase from *Streptomyces* sp. and [^2^H_0_]Girard P ([^2^H_0_]GP, chloride salt) were from Merck, Dorset, UK and TCI Europe, respectively. [^2^H_5_]GP (bromide salt) was synthesised as described by Crick et al. [26]. Pooled human plasma was NIST SRM 1950, Gaithersburg, MD, USA [22]. Pooled CSF was QC material generated by combining individual CSF samples from multiple donors in a study performed in association with the NYPUM project at the University Hospital of Umeå, Sweden. Plasma from a patient suffering from ACOX2 deficiency was as described in Ref. [10]. Plasma from a CTX patient was from previous studies in our laboratories. All participants or their parents/guardians provided informed consent and the studies were performed with institutional review board approval and adhered to the principles of the Declaration of Helsinki.

### 2.2 Extraction of non-esterified sterols including oxysterols and sterol-acids

#### 2.2.1 Plasma - OxysterolSPLASH

Plasma (100 μL) was added *dropwise* to an alcohol solution (1.050 mL) made up of 50 μL of OxysterolSPLASH ([^2^H_7_]24R/S-HC, 4 ng; [^2^H_6_]25-HC, 0.5 ng; [^2^H_6_](25R)26-HC, 8 ng; [^2^H_7_]7α-HC, 3 ng; [^2^H_7_]7β-HC, 0.5 ng; [^2^H_7_]7-OC, 1.5 ng; [^2^H_7_]7α-HCO, 1 ng; [^2^H_6_]7α,25-diHC, 0.05 ng; [^2^H_6_]7α,(25R/S)26-diHC, 0.1 ng; [^2^H_3_]7αH,3O-CA(25R/S), 3.5 ng; [^2^H_7_]4β-HC, 1.5 ng; [^2^H_7_]22R–HC, 0.25 ng; and [^2^H_7_]5α,6β-diHC, 0.5 ng) and 1.000 mL of ethanol containing [25,26,26,26,27,27,27-^2^H_7_]22S-hydroxycholest-4-en-3-one ([^2^H_7_]22S–HCO, 10 ng) and [^2^H_7_]cholesterol (20 μg) in a microcentrifuge tube *under sonication* in an ultrasonic bath. The solution was diluted with 350 μL water to give a 70% alcohol solution (1.500 mL). This was sonicated for a further 5 min, then centrifuged at 17,000×*g* at 4 °C for 30 min (see Supplemental Methods for flowchart 1).

For standard addition experiments unlabelled authentic standards (5 quantities covering a 5-fold concentration range, see Supplemental Table S3A) were added in differing quantities to 100 μL of plasma with the protocol otherwise unchanged. For experiments to optimise the quantity of OxysterolSPLASH the original protocol was followed with 100 μL plasma added to 1.050 mL of alcohol containing different amounts of OxysterolSPLASH (100 μL, 50 μL, 25 μL, 12.5 μL or 6.25 μL, see Supplemental Table S2 and Supplemental Methods for flowchart 2).

Oxysterols and sterol-acids were separated from cholesterol and sterols of similar lipophilicity by solid phase extraction (SPE), using a “certified Sep-Pak tC_18_” column (200 mg, Waters Inc, Elstree, Herts, UK). The column, SPE1, was first washed with absolute ethanol (4 mL), then conditioned with 70% ethanol (6 mL). The sterol extract from above in 70% alcohol (1.5 mL) was applied to the column and allowed to flow at a rate of 0.25 mL/min. If necessary, flow was assisted by negative pressure at the column outlet. The column flow-through was collected and combined with a column wash of 70% ethanol (5.5 mL). Oxysterols and sterol-acids elute in this fraction SPE1-Fr1 (7 mL, 70% alcohol). The column was washed further with 70% ethanol (4 mL) to give SPE1-Fr2. Cholesterol and sterols of similar lipophilicity were eluted with absolute ethanol (2 mL) to give SPE1-Fr3. More lipophilic sterols were eluted with further absolute ethanol (2 mL) to give SPE1-Fr4. Each fraction was divided into two equal parts (A) and (B) and lyophilised.

#### 2.2.2 Plasma - quantitative isotope-labelled standards

Plasma (100 μL) was added *dropwise* to absolute ethanol (1.050 mL) containing quantitative isotope-labelled internal standards [^2^H_6_]24R/S-HC (20 ng), [^2^H_7_]7α-HC (20 ng), [^2^H_7_]7-OC (20 ng) and [^2^H_7_]cholesterol (20 μg) along with standards [25,26,26,26,27,27,27-^2^H_7_]22R-hydroxycholest-4-en-3-one ([^2^H_7_]22R–HCO, 20 ng) or [^2^H_7_]22S–HCO (20 ng), [^2^H_6_]7α,25-diHC (2 ng) and [^2^H_7_]5α,6β-diHC (20 ng) in a microcentrifuge tube under *sonication* in an ultrasonic bath. The solution was diluted with 350 μL of water to give a 70% alcohol solution. This was sonicated for a further 5 min, then centrifuged at 17,000×*g* at 4 °C for 30 min. Further sample preparation was exactly as in *2.2.1*.

#### 2.2.3 CSF - OxysterolSPLASH

CSF (100 μL) was added *drop-wise* to an alcohol solution (2.100 mL) made up of 20 μL OxysterolSPLASH (containing [^2^H_7_]24R/S-HC, 1.6 ng; [^2^H_6_]25-HC, 0.2 ng; [^2^H_6_](25R)26-HC, 3.2 ng; [^2^H_7_]7α-HC, 1.2 ng; [^2^H_7_]7β-HC, 0.2 ng; [^2^H_7_]7-OC, 0.6 ng; [^2^H_7_]7α-HCO, 0.4 ng; [^2^H_6_]7α,25-diHC, 0.02 ng; [^2^H_6_]7α,(25R/S)26-diHC, 0.04 ng; [^2^H_3_]7αH,3O-CA(25R/S), 1.4 ng; [^2^H_7_]4β-HC, 0.6 ng; [^2^H_7_]22R–HC, 0.1 ng; and [^2^H_7_]5α,6β-diHC, 0.2 ng), 10 μL methanol (containing [^2^H_7_]22S–HCO (1 ng) and [^2^H_7_]cholesterol (200 ng) and 2.070 mL of absolute ethanol in a 15 mL Corning tube under *sonication* in an ultrasonic bath. The solution was diluted to 70% ethanol by the addition of water (800 μL), sonicated for 5 min, then centrifuged at 2400×*g* at 4 °C for 30 min. Alternatively, the CSF volume was either 200 μL, 100 μL or 50 μL and the OxysterolSPLASH volume varied between 10 μL and 20 μL, maintaining overall alcohol and aqueous volumes as above. For standard addition experiments unlabelled authentic standards were added in differing quantities (5 quantities covering a 5-fold range, see Supplemental Table S3B) to 100 μL of CSF, with the protocol using 20 μL of OxysterolSPLASH otherwise unchanged (see Supplemental Methods for flowchart 2, lower panel).

Oxysterols and sterol-acids were separated from cholesterol and sterols of similar lipophilicity by SPE, using a “certified Sep-Pak tC_18_” column washed and conditioned as in *2.2.1*. The sterol extract from CSF now in 70% alcohol (3 mL) was applied to the column and allowed to flow at a rate of 0.25 mL/min. If necessary, flow was assisted by negative pressure at the column outlet. The flow-through was collected and combined with a column wash of 70% ethanol (4 mL). Oxysterols and sterol-acids elute in this fraction SPE1-Fr1 (7 mL, 70% alcohol). The column was washed further with 70% ethanol (4 mL) to give SPE1-Fr2. Cholesterol and sterols of similar lipophilicity were eluted with absolute ethanol (2 mL) to give SPE1-Fr3. More lipophilic sterols were eluted with an additional 2 mL of absolute ethanol to give SPE1-Fr4. Each fraction was divided into two equal parts A and B and lyophilised.

#### 2.2.4 CSF - quantitative isotope-labelled standards

The procedure described in *2.2.3* was repeated, except 250 μL of CSF was added to an ethanol solution (2.100 mL) containing [^2^H_7_]24R/S-HC (2 ng), [^2^H_7_]7α-HC (2 ng), [^2^H_7_]22R–HCO (2 ng) and [^2^H_7_]cholesterol (800 ng) prior to dilution to 3 mL of 70% ethanol.

### 2.3 Extraction and hydrolysis of esterified sterols including oxysterols and sterol-acids

#### 2.3.1 Plasma - OxysterolSPLASH

Plasma (100 μL) was added *drop-wise* to a freshly prepared solution of 0.35 M KOH [19] in 1.050 mL of alcohol made up of OxysterolSPLASH (50 μL), [^2^H_7_]22S–HCO (10 ng) and [^2^H_7_]cholesterol (40 μg) in methanol (100 μL) and ethanolic KOH (900 μL, 3.66 × 10^−4^ mol KOH), *under sonication* in a microcentrifuge tube. The solution was sonicated for a further 5 min and incubated at room temperature in the dark for 2 h, after which it was neutralised by addition of 350 μL of water containing 21 μL of glacial acetic acid (3.66 × 10^−4^ mol). The mixture was then ultrasonicated for 5 min and then centrifuged at 17,000×*g* at 4 °C to remove any precipitated matter. The solution (1.5 mL, 70% alcohol) was then applied to SPE1 and processed as in *2.2.1.*

The procedure was repeated with the same volume of plasma (100 μL) but the volume of OxysterolSPLASH was varied from 200 μL to 6.25 μL. To maintain the ultimate volume of alcohol at 1.050 mL at 0.35 M KOH the volume and molarity of ethanolic KOH was adjusted appropriately. An additional experiment was performed with a plasma volume of 10 μL, volume of OxysterolSPLASH of 10 μL and keeping the aqueous and alcohol proportions unchanged but reducing [^2^H_7_]cholesterol proportionately to the reduction in OxysterolSPLASH.

#### 2.3.2 CSF - OxysterolSPLASH

CSF (100 μL) was added *dropwise* to a freshly prepared solution made up of OxysterolSPLASH (20 μL), [^2^H_7_]22S–HCO (1 ng) and [^2^H_7_]cholesterol (200 ng) in methanol (10 μL) and 2.070 mL of 0.35 M ethanolic KOH (7.25‬ x 10^−4^ mol) *under* sonication. The solution was sonicated for a further 5 min and incubated at room temperature in the dark for 2 h, after which it was neutralised by addition of 800 μL of water containing 41.6 of μL glacial acetic acid (7.25 × 10^−4^ mol). The mixture was then ultrasonicated for 5 min and then centrifuged at 2400×*g* at 4 °C to remove any precipitated matter. The solution (3 mL, 70% alcohol) was then applied to SPE1 and processed as in 2.2.3.‬‬‬‬‬‬‬‬‬‬‬‬‬‬‬‬‬‬‬ The procedure was repeated with the CSF volume increased to 200 μL and the volume of water adjusted to give a final volume of 3 mL, 70% alcohol.‬‬‬‬‬ In a further experiment, OxysterolSPLASH was replaced by [^2^H_6_]24R/S-HC (2 ng) in ethanol. ‬‬‬‬‬‬‬‬‬‬‬‬‬‬‬‬‬‬‬‬‬‬‬‬‬‬‬‬‬‬‬‬‬‬‬‬‬‬‬‬‬‬‬‬‬‬‬‬‬‬‬‬‬‬‬‬‬‬‬‬‬‬‬‬‬‬‬‬‬‬‬‬‬‬‬‬‬‬‬‬‬‬‬‬‬‬‬‬‬‬‬‬‬‬‬‬‬‬‬‬‬‬‬‬‬‬‬‬‬‬‬‬‬‬‬‬‬‬‬‬‬‬‬‬‬‬‬‬

### 2.4 Enzyme-assisted derivatisation for sterol analysis (EADSA)

**Fig. 1:**
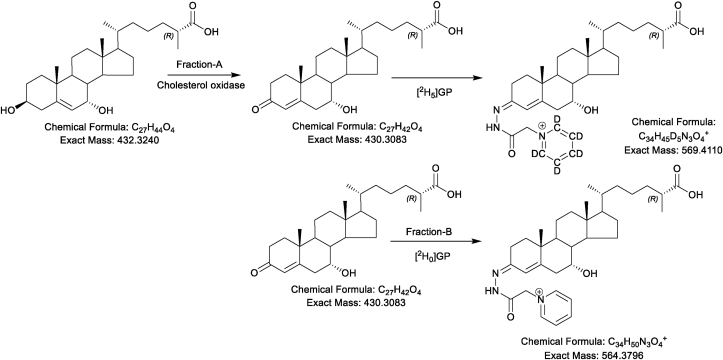
Schematic depicting the EADSA method. In fraction-A cholesterol oxidase converts 3β-hydroxy-5-ene functions to 3-oxo-4-ene groups which are derivatised with [^2^H_5_]GP. Any natural 3-oxo-4-ene containing sterols will be similarly derivatised with [^2^H_5_]GP. In fraction-B cholesterol oxidase is absent so only *oxosterols*, e.g. 3-oxo-4-enes (and 7-oxo-5-enes), will become derivatised, in this case with [^2^H_0_]GP. Deconvolution of data from fractions-A and -B provides the quantities of sterols with an original 3β-hydroxy-5-ene function (i.e. A-B), while fractions-B provide quantities of *oxosterols*.

To enhance the signal for sterol, oxysterol and sterol-acid analysis by liquid chromatography (LC)-MS derivatisation strategies are often used [[32], [33], [34], [35]]. Here to enhance the signal in LC - electrospray ionisation (ESI)-MS we have adopted EADSA technology described in Fig. 1 [26,31].

Each dried SPE1 fraction was reconstituted in propan-2-ol (100 μL) and thoroughly vortexed. To A-fractions 50 mM phosphate buffer (KH_2_PO_4_, pH 7, 1.000 mL) containing cholesterol oxidase (3 μL, 2 μg/μL in water, 44 mU/μg protein) was added and the mixture incubated at 37 °C for 1 h, after which the reaction was quenched with methanol (2.000 mL). Glacial acetic acid (150 μL) was then added and the solution thoroughly vortexed. [^2^H_5_]GP reagent (190 mg, bromide salt) was added to this solution which was thoroughly vortexed and incubated at room temperature overnight in the dark. B-fractions were treated in an identical manner but in the absence of cholesterol oxidase and with [^2^H_0_]GP (150 mg, chloride salt) replacing [^2^H_5_]GP. When a hydrolysis step was included, for plasma analysis the above mixture was centrifuged at 2400×*g* at room temperature for 30 min prior to further sample preparation. This was performed to avoid blocking of the second SPE column (see below).

To remove excess derivatisation reagent the reaction mixture was subjected to a second SPE step, i.e. SPE2. An Oasis HLB column (60 mg, Waters Inc) was washed with 100% methanol (6 mL), 10% methanol (6 mL) and conditioned with 70% methanol (4 mL). The reaction mixture from above (3.25 mL, 69% organic) was loaded onto the column followed by 70% methanol (1 mL), used to rinse the reaction vial. The combined eluent was diluted with water (4 mL) to 35% methanol. The column was equilibrated with 35% methanol (1 mL) which was added to the diluted eluent to give 9 mL of 35% methanol. This solution was re-applied to the column and the eluent diluted with water (9 mL) to give 18 mL of 17.5% methanol. The column was equilibrated with 17.5% methanol (1 mL) and eluents combined. The resultant solution (19 mL 17.5% methanol) was applied to the column and the effluent discarded. At this point all GP-derivatised sterols including oxysterols and sterol-acids are retained on the column. The column was finally washed with 10% methanol (6 mL) and GP-derivatives eluted in 3 × 1 mL of methanol followed by 1 mL of ethanol. Oxysterols and cholestenoic acids elute in the first two 1 mL fractions (SPE2-Fr1+Fr2), and cholesterol elutes across the first three 1 mL fractions (SPE2-Fr1+Fr2+Fr3). For oxysterol and sterol-acid analysis equal volumes of SPE2-Fr1+Fr2 derived from fraction-A and from fraction-B were then combined diluted to 60% methanol and analysed by LC-MS. Similarly, for cholesterol analysis, equal volumes of SPE2-Fr1+Fr2+Fr3 derived originally from SPE1-Fr3A and from SPE1-Fr3B were combined and diluted to 60% methanol, followed by dilution by a factor of up to 1000 in 60% methanol and analysed by LC-MS. Note, in 100% methanol the derivatives are stable for several months when stored at −20 °C [26,31].

### 2.5 LC-MS with multistage fragmentation (MS^n^)

Analysis was performed on either an Orbitrap Elite mass spectrometer equipped with an ESI probe (Thermo Fisher Scientific, Hemel Hempstead, UK) with prior chromatographic separations on an Ultimate 3000 LC system (Dionex, now Thermo Fisher Scientific), essentially as described previously [28,31] or on an Orbitrap IDX Tribrid mass spectrometer similarly equipped with an ESI probe and linked to an Ultimate 3000 LC system. The column used was Hypersil Gold C_18_ (50 × 2.1 mm, 1.9 μm, Thermo Fisher Scientific). Two chromatographic gradients were employed, a 17 min gradient and a 35 min gradient described in Refs. [28,31]. On the Orbitrap Elite instrument three to five scan events were performed: one high resolution (120,000, FWHM at *m/z* 400) MS scan event in the Orbitrap analyser in parallel with two to four multi-stage fragmentation (MS^n^) scan events in the linear ion trap (LIT). Similar scan parameters were utilised on the IDX instrument. One scan event was performed in the Orbitrap analyser (120,000 FWHM at *m/z* 400) in parallel to five scan events in the ion trap. One difference between MS^n^ scans on the Orbitrap Elite and IDX is that with the Elite all *m/z* selection is in the LIT, while on the IDX the first *m/z* selection was by the quadropole mass filter. Quantification was performed by stable isotope dilution or using isotope labelled structurally similar compounds.

## 3 Results

### 3.1 Non-esterified sterols including oxysterols and sterol-acids in plasma

#### 3.1.1 Chromatography of GP-derivatised sterols including oxysterols and cholestenoic acids targeted by OxysterolSPLASH

**Fig. 2:**
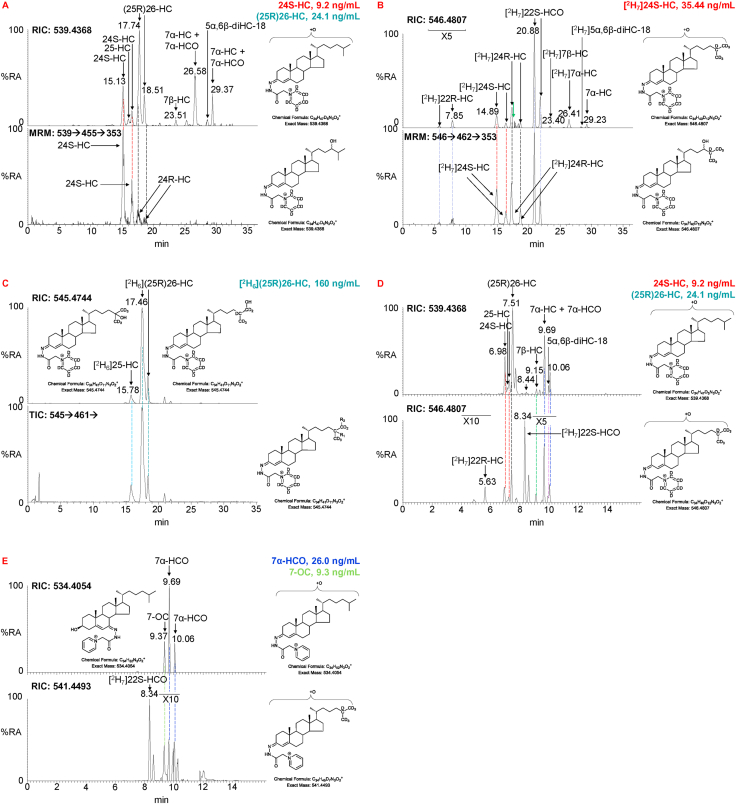
LC-MS separation of GP-derivatised monohydroxycholesterols (HC). (A) Upper panel, RIC of the [M]^+^ ions of monohydroxycholesterols (539.4368 ± 5 ppm) found in plasma. Lower panel, MRM 539.4 → 455.4→353.3 characteristic of 24R/S-HC. The red dashed line indicates the coincidence of 24S-HC in the upper and lower panels and the black dashed lines indicates where 24R–HC partially overlaps (in time, but not in MRM) with (25R)26-HC. (B) Upper panel, RICs for [^2^H_7_]-labelled monohydroxycholesterols (546.4807 ± 5 ppm). The green arrow indicated the distortion in the [^2^H_7_]24R–HC chromatographic peak as a consequence of the co-eluting and mass spectrometrically-unresolved [M+1]^+^ ion of [^2^H_6_](25R)26-HC (*m/z* 546.4777). Lower panel, MRM 546.5 → 462.4→353.3 characteristic of [^2^H_7_]24R/S-HC. Note the fragment ion at *m/z* 353.3 is also evident in MS^3^ spectra of [^2^H_7_]22R/S–HCO. Coloured dashed lines indicate the coincidence of peaks of the same oxysterol. (C) Upper panel, RICs for [^2^H_6_]-labelled monohydroxycholesterols (545.4744 ± 5 ppm). Lower panel total ion chromatogram (TIC) 545.5 → 461.4→ for [^2^H_6_]-labelled monohydroxycholesterols. (D) RIC for monohydroxycholesterols in plasma (upper panel) and [^2^H_7_]-labelled standards (lower panel) recorded on a shorter chromatographic time scale. (E) RIC for monohydroxycholestenones in plasma (534.4054 ± 5 ppm) and [^2^H_7_]-labelled standards (541.4493 ± 5 ppm). Note in all chromatograms the deuterium labelled oxysterols elute slightly earlier than their non-labelled analogues. Relevant MS^3^ spectra are presented in Supplemental Figs. S3 and S5. (For interpretation of the references to colour in this figure legend, the reader is referred to the Web version of this article.)

Most of the GP-derivatised monohydroxycholesterols (HC) targeted by the OxysterolSPLASH mix are chromatographically separated by the 17 min and 37 min gradients (Fig. 2). In fact, the high selectivity of the chromatographic system employed results in chromatographic separation of GP-derivatised epimeric oxysterols e.g. 24S-HC and 24R–HC (asymmetric carbon at C-24, see Fig. 2A, lower panel), which is not normally achieved in conventional LC-MS or gas chromatography (GC)-MS studies [36,37]. This is advantageous as it allows the detection of both the major (24S-HC) and minor (24R–HC) epimers of 24-HC in human plasma. However, this advantage comes with the penalty of complicating the ultimate chromatogram. The chromatographic system employed also has the selectivity to separate *syn* and *anti* conformers of the GP-derivative (see Supplemental Fig. S1), thereby enhancing the reliability of identification of oxysterols but further complicating the chromatogram and consequently 24S-HC and 24R–HC each give two chromatographic peaks (Fig. 2A, lower panel).

Of the monohydroxycholesterols targeted by the OxysterolSPLASH mix 24R–HC is only partially resolved in time from (25R)26-HC (Fig. 2A). This does not create a problem for human plasma or CSF samples as 24R–HC is only a minor component of both fluids (i.e. <10% of 24S-HC and <5% of (25R)26-HC) and there is minimal distortion of the peak shape for (25R)26-HC in the appropriate reconstructed ion chromatogram (RIC, *m/z* 539.4368 ± 5 ppm, Fig. 2A). [^2^H_7_]24-HC in the OxysterolSPLASH mix is a mixture of [^2^H_7_]24R–HC and [^2^H_7_]24S-HC and the certified concentration is for the combination of the two i.e. 80 ng/mL (Fig. 2B, lower panel). Assuming an identical response factor for the two epimers the concentration of the [^2^H_7_]24S-HC epimer in the OxysterolSPLASH mix was determined to be 35.44 ng/mL and can thus be used for quantification of endogenous 24S-HC. The partial chromatographic resolution (in time) of [^2^H_7_]24R–HC and [^2^H_6_](25R)26-HC in the OxysterolSPLASH mix does not affect the chromatographic peak for [^2^H_6_](25R)26-HC (Fig. 2C) as [^2^H_6_](25R)26-HC (*m/z*_*calc*_ 545.4744) is 1 Da lighter than [^2^H_7_]24R–HC (*m/z*_*calc*_ 546.4807) and they are resolved by mass. However, the M+1 peak of [^2^H_6_](25R)26-HC (*m/z*_*calc*_ 546.4777) has almost the same mass as [^2^H_7_]24R–HC and the two isomers are not resolved by mass (Supplemental Fig. S2A). This distorts the RIC peak of [^2^H_7_]24R–HC as indicated by the green arrow in Fig. 2B (upper panel). However, if 24R–HC is of interest in biological samples, this problem is readily overcome by generating multiple-reaction-monitoring (MRM) chromatograms utilising the LIT for fragmentation of 24R/S-HC and [^2^H_7_]24R/S-HC and exploiting the transitions [M]^+^→[M-Py]^+^→353.3, where Py corresponds to pyridine (Fig. 2A & B, lower panels). As the fragment ion at *m/z* 353.3 is a major ion in the MS^3^ spectra of 24R–HC and 24S-HC (Supplemental Figure S3A – S3D, see also Fig. S4A for mechanism of formation) but is essentially absent (RA <1%) from the fragmentation spectrum of (25R)26-HC (Supplemental; Figs. S3G and S3H), (25R)26-HC is essentially transparent to this transition. Besides 24S-HC, 24R–HC and (25R)26-HC, other targeted monohydroxycholesterols, 25-HC, 7α-HC and 7β-HC are separated as is evident in Fig. 2A (upper panel) and also by the shorter gradient as shown in Fig. 2D (upper panel). 22R–HC is a minor oxysterol in adult plasma and is not detected in the NIST SRM 1950 plasma sample (Fig. 2A & D, upper panels), although the isotope labelled form is clearly evident (Fig. 2B upper panel & 2D lower panel). It is noteworthy that 22R–HC is evident in plasma from pregnant women. While GP-derivatised monohydroxycholesterols are exclusive to the A-fractions and have an odd numbered mass, the hydroxycholestenones (HCO), 7α-HCO and 7-OC appear in B-fractions and have an even numbered mass (Fig. 2E & Supplemental Fig. S2B, see Fig. S5 for MS^3^ spectra). Note, during GP-derivatisation 5α,6β-diHC becomes dehydrated to 6β-hydroxycholesterol and this is the species monitored here (6β-HC, i.e. 5α,6β-diHC-18).

**Fig. 3:**
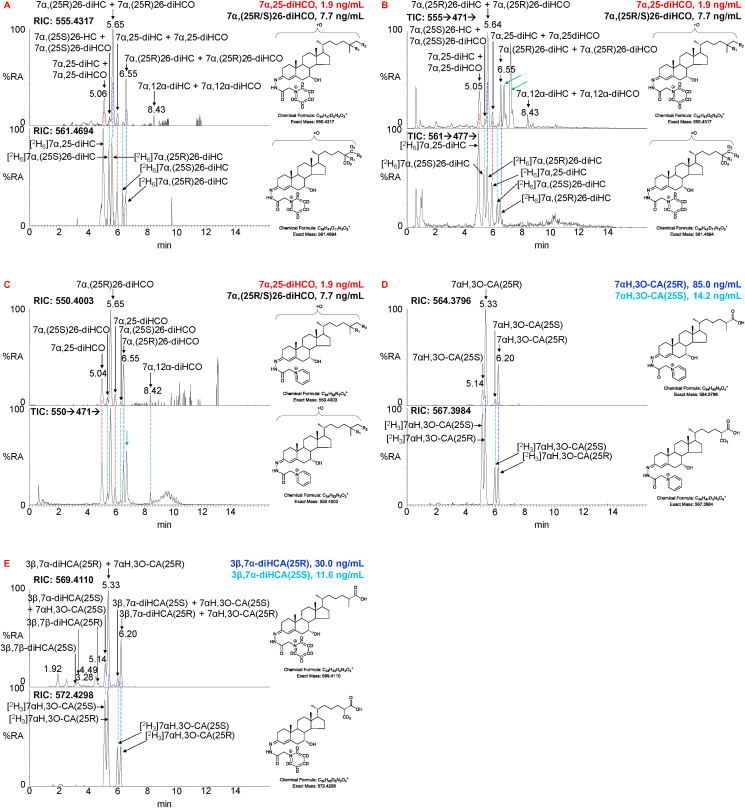
LC-MS separation of GP-derivatised dihydroxycholesterols (diHC), dihydroxycholestenones (diHCO), dihydroxycholestenoic (diHCA) and hydroxyoxocholestenoic (H,O-CA) acids. (A) RIC for the [M]^+^ ions of (upper panel) 7α,25-diHC + 7α,25-diHCO and 7α,(25R/S)26-diHC + 7α,(25R/S)26-diHCO (555.4317 ± 5 ppm) found in plasma, and (lower panel) [^2^H_6_]7α,25-diHC and [^2^H_6_]7α,(25R/S)26-diHC (561.4694 ± 5 ppm) over a 17 min gradient. (B) MS^3^ ([M]^+^→[M-Py]^+^→) TICs for (upper panel) 7α,25-diHC + 7α,25-diHCO and 7α,(25R/S)26-diHC + 7α,(25R/S)26-diHCO (555.4 → 471.4→) found in plasma and (lower panel) [^2^H_6_]7α,25-diHC and [^2^H_6_]7α,(25R/S)26-diHC (561.5 → 477.4→) over a 17 min, gradient. Note the additional peaks in the upper panel labelled by green arrows arise from fragmentation of the [M+2]^+^ peaks with monoisotopic *m/z* of 553.4161. In the sterol structures R_1_ is OH in 7α,25-diHC and R_2_ is OH in 7α,(25R/S)26-diHC. (C) RIC for the [M]^+^ ions (550.4003 ± 5 ppm, upper panel) and TICs for the MS^3^ fragmentation (550.4 → 471.4→, lower panel) of 7α,25-diHCO and 7α,(25R/S)26-diHCO found in plasma. Note the additional peak in the lower panel labelled by the green arrow arises from fragmentation of the [M+2]^+^ peak with monoisotopic *m/z* 548.3847. (D) RIC for the [M]^+^ ions of (upper panel) 7αH,3O-CA(25R/S) (564.3796 ± 5 ppm) found in plasma and (lower panel) [^2^H_3_]7αH,3O-CA(25R/S) (567.3984 ± 5 ppm) over a 17 min gradient. (E) RIC for the [M]^+^ ions of (upper panel) 7αH,3O-CA(25R/S) + 3β,7α-diHCA(25R/S) (569.4110 ± 5 ppm) found in plasma and (lower panel) [^2^H_3_]7αH,3O-CA(25R/S) (572.4298 ± 5 ppm) over a 17 min gradient. Coloured dashed lines indicate the coincidence of peaks of the same oxysterol. Chromatograms recorded over a 37 min gradient can be found in Supplemental Fig. S6. MS^3^ spectra are presented in Supplemental Figs. S7 and S8. (For interpretation of the references to colour in this figure legend, the reader is referred to the Web version of this article.)

Isotope-labelled dihydroxycholesterols (diHC) in the OxysterolSPLASH mix include [^2^H_6_]7α,25-diHC and a mixture of [^2^H_6_]7α,(25R)26-diHC and [^2^H_6_]7α,(25S)26-diHC epimers (asymmetric carbon at C-25). Following GP-derivatisation all three isomer are resolved almost to base line in the 17 min gradient (Fig. 3A & B, lower panels) and to base line in the 37 min gradient (Supplemental Fig. S6A, lower panel). A similar response factor is assumed for [^2^H_6_]7α,(25R)26-diHC and [^2^H_6_]7α,(25S)26-diHC and we have measured the quantity of the 7α,(25R)26-diHC and 7α,(25S)26-diHC epimers in combination in the plasma sample. If the individual epimers are of interest, their quantities could be measured. In human plasma the dihydroxycholestenones (diHCO) 7α,(25R/S)26-dihydroxycholest-4-en-3-one (7α,(25R/S)26-diHCO) and 7α,25-dihydroxycholest-4-en-3-one (7α,25-diHCO) are more abundant than the analogous dihydroxycholesterols (Table 1, Fig. 3C). The dihydroxycholesterol isomers are only found in fraction-A while the dihydroxycholestenone isomers are in both A- and B-fractions. The amount of dihydroxycholestenone is that measured in fraction-B and that of dihydroxycholesterol calculated by subtracting values in fraction-B from those in fraction-A. MS^3^ spectra of dihydroxycholesterols and dihydroxycholestenones are presented in Supplemental Fig. S7.

**Table 1:**
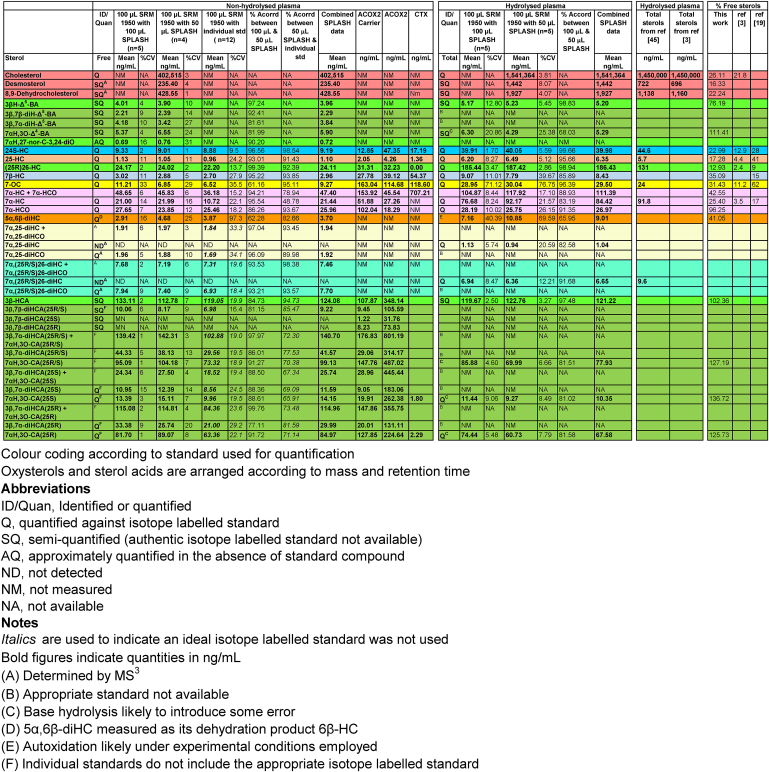
Sterols including oxysterols and sterol-acids quantified, semi-quantified or approximately quantified in human plasma.

In human plasma the cholestenoic acid 7αH,3O-CA(25R/S) is abundant, predominantly as the 25R-epimer (asymmetric carbon at C-25), although the 25S-epimer is also present to a lesser extent (Fig. 3D). In both the 17 min and 37 min (see Supplemental Fig. S6C) gradients the two epimers are almost completely resolved, but not quite to base line. Here we assume an equivalent response factor for both epimers and have determined their quantity in combination and also as individual epimers using the [^2^H_3_]7αH,3O-CA(25R/S) internal standard (Table 1). In addition to 7αH,3O-CA(25R/S), both epimers of 3β,7α-dihydroxycholest-5-en-(25R/S)26-oic acid [(3β,7α-diHCA(25R/S)] (Fig. 3E) are observed in plasma and were quantified using [^2^H_3_]7αH,3O-CA(25R/S). Their combined concentration is reported and also for the individual epimers (Table 1).

Besides the oxysterols and cholestenoic acids targeted in the OxysterolSPLASH mix, cholesterol was also quantified via an additional quantitative standard, [^2^H_7_]cholesterol. Cholesterol and similarly lipophilic sterols, 8(9)-dehydrocholesterol (8-DHC), an enzymatically formed isomer of 7-dehydrocholesterol (7-DHC) [38], and desmosterol are separated from oxysterols and sterol-acids on SPE1 during sample preparation and analysed in a separate LC-MS(MS^n^) run (Supplemental Fig. S9).

#### 3.1.2 Chromatography of additional GP-derivatised sterols including oxysterols and sterol-acids

Besides the oxysterols and cholestenoic acids targeted for absolute quantification by the OxysterolSPLASH mix there are numerous other sterols and cholesterol metabolites revealed by EADSA and LC-MS(MS^n^) analysis of plasma (see Supplemental Methods for flowchart 3 illustrating the identification process) [39,40], these include 25-hydroxyvitamin D_3_ (25-D_3,_ Supplemental Fig. S10), 7α,12α-dihydroxycholesterol (7α,12α-diHC) and 7α,12α-dihydroxycholest-4-en-3-one (7α,12α-diHCO, Fig. 3A–C); 3β-hydroxycholest-5-en-(25R)26-oic (3β-HCA) and 3-oxocholest-4-en-(25R)26-oic (3O-CA) acids (Supplemental Fig. S10D); both 25R and 25S epimers of 3β,7β-dihydroxycholest-5-en-(25R/S)26-oic acid ([3β,7β-diHCA(25R/S)], Fig. 3E); 3β,7α,24-trihydroxycholest-5-en-26-oic acid (3β,7α,24-triHCA) in combination with 7α,24-dihydroxy-3-oxocholest-4-en-26-oic acid (7α,24-diH,3O-CA, Supplemental Figs. S10E and F); 3β,7α,25-trihydroxycholest-5-en-26-oic acid (3β,7α,25-triHCA) in combination with 7α,25-dihydroxy-3-oxocholest-4-en-26-oic acid (7α,25-diH,3O-CA, Supplemental Figs. S10E and G); 3β-hydroxychol-5-en-24-oic acid (3βH-Δ^5^-BA, Supplemental Fig. S10B); 3β,7α-dihydroxychol-5-en-24-oic (3β,7α-diH-Δ^5^-BA), 7α-hydroxy-3-oxochol-4-en-24-oic (7αH,3O-Δ^4^-BA) and 3β,7β-dihydroxychol-5-en-24-oic (3β,7β-diH-Δ^5^-BA, Supplemental Fig. S10C) acids. Authentic standards are available for all these metabolites allowing their definitive identification. It should be noted that if 25-D_3_ is to be accurately quantified initial extraction from plasma should be into acetonitrile rather than ethanol [41].

In addition to the oxysterols and sterol-acids listed above we have made partial identifications of 8 other sterols based on their exact mass and MS^3^ spectra. The interpretation of their MS^3^ spectra is provided in fragmentation schemes illustrated in Supplemental Fig. S4 and their structures are listed in Supplemental Table S1. Simplified rules for structure determination are provided in Table S4.

#### 3.1.3 Quantification GP-derivatised sterols including oxysterols and cholestenoic acids targeted by OxysterolSPLASH

24S-HC, 25-HC, (25R)26-HC, 7β-HC and 5α,6β-diHC do not have a natural 3-oxo analogue and their GP-derivatives are only found in fraction-A (Table 1). Cholesterol oxidase is required for their GP-derivatisation. 7-OC is derivatised in the absence of cholesterol oxidase, hence its quantity in plasma is determined using data from fraction-B alone. 7α-HC, 7α,25-diHC, 7α,(25R/S)26-diHC, 3β,7α-diHCA(25R/S) and their analogous 3-oxo compounds may be present in plasma and following GP-derivatisation both 3β-hydroxy and 3-oxo entities are found in fraction-A but only the 3-ones are present in fraction-B. Note, hydroxysteroid dehydrogenase (HSD) 3B7, the dominant enzyme that converts sterols with a 3β-hydroxy-5-ene structure to a 3-oxo-4-ene in the bile acid biosynthesis pathways requires a 7α-hydroxy group in the substrate [42].

##### 3.1.3.1 Optimal amounts, standard curves, reproducibility, apparent extraction efficiency and accuracy

Each of the sterols, including oxysterols and cholestenoic acids, to be quantified is naturally present in plasma. Unfortunately, unlike the situation for exogenous compounds, for sterol analysis a true blank plasma sample does not exist and neither is it possible to prepare one [43]. As pointed out by Sjövall, as the cholesterol level in plasma is so high, if sterols were to be removed from plasma, the matrix would no longer be plasma [43]. Thus, to investigate the proportionality of response to concentration, equation (1) was tested by varying the ratio of [^2^H_0_]Sterol (un-labelled) to [^2^H_n_]Sterol (isotope-labelled standard) using otherwise unadulterated plasma.

(1) PA[^2^H_0_]Sterol / PA[^2^H_n_]Sterol = (Rf[^2^H_0_]Sterol / Rf[^2^H_n_]Sterol) x (Conc. [^2^H_0_]Sterol / Conc. [^2^H_n_]Sterol) + constant

Where PA[^2^H_0_]Sterol corresponds to peak area measured for an unlabelled-sterol present in (or added to) plasma; Conc. [^2^H_0_]Sterol corresponds to the concentration of unlabelled sterol present in (or added to) plasma and Rf[^2^H_0_]Sterol corresponds to the response factor for [^2^H_0_]Sterol in (or added to) plasma. The equivalent terms, but where [^2^H_n_] substitutes for [^2^H_0_], correspond to peak area, concentration and response factor of isotope-labelled sterols added to plasma in the OxysterolSPLASH mix.

*Varying the amount of OxysterolSPLASH.* Initial experiments were performed with 100 μL of plasma and adding different amounts of OxysterolSPLASH to find an optimal amount of internal standard for quantitative analysis. The experiment was performed over five concentration levels, ranging from 0.0625 units (6.25 μL) of OxysterolSPLASH to 1 unit (100 μL, i.e. the volumes of plasma and OxysterolSPLASH are equivalent). For 7α,25-diHC and 7α,(25R/S)26-diHC measured in combination with their 3-ones only data for 1–0.25 and 1–0.5 units, respectively, were included due to the low levels of [^2^H_6_]7α,25-diHC and [^2^H_6_]7α,(25R/S)26-diHC in OxysterolSPLASH. The resultant data is provided in Supplemental Table S2. Equation (1) is in the form of y = mx + c and apart from 7-OC (R^2^ = 0.97) and 5α,6β-diHC (R^2^ = 0.91), all analytes tested gave an R^2^ > 0.99. It should be noted that 7α-HC, 7β-HC, 7-OC and 5α,6β-diHC can all be formed *ex vivo* during sample handling as well as being present *in vivo* [44]. With the exception of the metabolites derived by *ex vivo* oxidation all other analytes gave %CVs <15% and all metabolites gave accuracy ≥70%, accuracy being is defined as the agreement between actual measured concentration and that derived from eq. (1).

As 7α,(25R/S)26-diHC is of interest in the current study, further data analysis was confined to OxysterolSPLASH quantities of 1 and 0.5 units (100 μL and 50 μL) with 100 μL of plasma. The data set was expanded by deconvoluting endogenous 3β-hydroxy compounds from their 3-oxo analogues by simply subtracting quantities measured in fraction-B from those measured in fraction-A. This provides data sets for 7α-HC, 7α,25-diHC, 7α,(25R/S)26-diHC, 3β,7α-diHCA(25R) and 3β,7α-diHCA(25S) separately from 7α-HCO, 7α,25-diHCO, 7α,(25R/S)26-diHCO, 7αH,3O-CA(25R) and 7αH,3O-CA(25S), respectively (Table 1). The agreement in data obtained with 1 unit and 0.5 units of OxysterolSPLASH was good (>80%) except for 3β,7α-diHCA(25R), where the agreement was acceptable at 77%, and for two of the oxysterols that can also be formed by *ex vivo* autoxidation of cholesterol i.e. 7-OC and 5α,6β-diHC (both 60%).

*Standard additions.* To further confirm the validity of eq. (1), a standard additions approach was followed in which known amounts of unlabelled standard compounds were added, over a 5-fold range, to 100 μL of plasma prior to quantification with 50 μL (0.5 units) of OxysterolSPLASH. This confirmed the validity of eq. (1), as in all cases R^2^ > 0.99 and at each concentration accuracy, as determined as the % difference between the measured concentration at each level and that determined by solving equation (1), was >90% (Supplemental Table S3A). The standard additions experiment also allowed calculation of “apparent” extraction efficiency which is given by the efficiency of extraction of the added un-labelled standard. In all cases this was >90%.

It is not possible to extend the calibration line to concentrations lower than those that are present endogenously in an unadulterated matrix. Instead we have exploited technical dilutions of prepared samples to estimate a lower limit of quantification as the lowest concentration at which the measured concentration of analyte differs from the calculated concentration by less than 30% (Supplemental Table S3).

*Comparison of OxysterolSPLASH to individual isotope-labelled standards.* The current data set for 100 μL plasma and 0.5 units OxysterolSPLASH was compared to data generated using the same plasma sample but exploiting individual quantitative isotope-labelled standards [^2^H_6_]24R/S-HC, [^2^H_7_]7α-HC and [^2^H_7_]7-OC. Using [^2^H_6_]24(R/S)-HC as an internal standard for 24S-HC, 25-HC and (25R)26-HC, the agreement between the methods was good (>90%), as it was also using [^2^H_7_]7α-HC for 7α-HCO, and [^2^H_7_]7-OC for 7-OC was (90%, Table 1). The agreement for 7α-HC was poor, presumably as a consequence of its formation or that of [^2^H_7_]7α-HC by *ex vivo* autoxidation of cholesterol or [^2^H_7_]cholesterol, respectively. We have previously shown that using the EADSA approach [^2^H_6_]24R/S-HC can be used as a reasonable surrogate for not only side-chain mono-hydroxycholesterols but also other oxysterols [26]. This is confirmed here by the good agreement (≥90%) for the quantification of 7α,25-diHCO and 7α,(25R/S)26-diHCO against their [^2^H_6_]-labelled authentic standards and against [^2^H_6_]24R/S-HC. Agreements for 3β,7α-diHC(25R/S) and 7αH,3O-CA(25R/S) were only moderately good (>70%) on account of the [^2^H_6_]24R/S-HC standard not taking account of the lability of the 7-hydroxy-5-ene and 7-hydroxy-4-en-3-one skeletons, both of which are susceptible to dehydration [43].

To summarise, with 100 μL of plasma and either 1 or 0.5 units of OxysterolSPLASH reproducible data is generated for the target oxysterols and cholestenoic acids, with the exception of those that can be generated by *ex vivo* autoxidation of cholesterol during sample work-up.

#### 3.1.4 Semi-quantification of other oxysterol and sterol-acids in the absence of isotope-labelled standards

Besides cholesterol and the 16 oxysterols and cholestenoic acids listed in Table 1, semi-quantitative values were determined for another 8 sterols, oxysterols and sterol-acids in the absence of identical isotope-labelled surrogates and approximate quantification of one other oxysterol identified presumptively based on exact mass, MS^3^ spectrum and retention time (Table 1). The isotope-labelled standards used for each analyte were chosen based on structural similarity and are colour coded in Table 1.

A further 7 oxysterols and sterol-acids were identified but not quantified, while 8 further sterols were partially identified in the absence of authentic standards and were not quantified (see Supplemental Table S1).

### 3.2 Esterified oxysterols in plasma

Oxysterols are found in plasma in both the non-esterified (free) and esterified forms, where a hydroxy group is esterified to a fatty acyl group in a reaction predominantly catalysed by lecithin-cholesterol acyl transferase (LCAT). The esterified form is dominant [3,19] and most GC-MS and LC-MS studies are performed after a base-hydrolysis step and measure the sum of esterified and non-esterified oxysterols [3,16,[45], [46], [47]]. We have thus hydrolysed the NIST SRM 1950 plasma sample and investigated the use of the OxysterolSPLASH mix for sterol, including oxysterol and cholestenoic acid, quantification.

#### 3.2.1 Quantification

Potassium hydroxide is a strong base and besides hydrolysis of esters can catalyse the dehydration of labile hydroxy groups in sterols e.g. 7-hydroxy-5-ene and particularly 7-hydroxy-4-en-3-one [43]. If these compounds are to be analysed, an isotope-labelled version is required to take dehydration into account.

#### 3.2.2 Optimal amounts and reproducibility

Having investigated earlier the proportionality of analyte response to concentration as defined by eq. (1) through standard additions, we evaluated the optimum amount of OxysterolSPLASH for use when analysing 100 μL of hydrolysed plasma. The experiment was performed over five concentration levels, ranging from 0.0625 units (6.25 μL) of OxysterolSPLASH to 1 unit (100 μL). For the targeted oxysterols 24S-HC, 25-HC, (25R)26-HC, 7α-HC plus 7α-HCO, 7β-HC and 7-OC R^2^ ≥ 0.99, but for 7α-HCO, 5α,6β-diHC, 7α,25-diHC, 7α,(25R/S)26-diHC and 7αH,3O-CA(25R/S) sufficient signal of the isotope-labelled standard could only be achieved with 1 unit and 0.5 units of OxysterolSPLASH (data not shown). As the latter analytes are of interest, further data analysis was restricted to experiments with 100 μL of plasma and 1 or 0.5 units of OxysterolSPLASH. The agreement in analyte concentrations at these two levels of standard was >80% in all cases, except for 5α,6β-diHC (66%), which can be formed by *ex vivo* autoxidation of cholesterol during sample handling (Table 1).

In summary, 100 μL of plasma with either 1 or 0.5 units of OxysterolSPLASH generates reproducible data for the target oxysterols and cholestenoic acids.

#### 3.2.3 Semi-quantification of other sterols including oxysterol and sterol-acids in the absence of isotope-labelled standards

In comparison to non-esterified oxysterols and acids, the number of analytes that can be semi-quantified is reduced as a consequence of the lability of the 7-hydroxy-5-ene and 7-hydroxy-4-en-3-one structures in strongly basic solutions and a lack of authentic isotope-labelled standards available to compensate for this. The data generated is presented in Table 1.

#### 3.2.4 Comparison of data for esterified and non-esterified sterols

In agreement with earlier reports, about 25% of cholesterol is present in its non-esterified form [3], while levels of non-esterified side-chain hydroxycholesterols varied from about 10 to 25% [3,19] (Table 1). The % of non-esterified ring-oxidised sterols was higher, ranging from about 30% for 7-OC to 96% for 7α-HCO where there is no 3β-hydroxy group available for esterification. 3β-HCA was found to be essentially all in the free form; this is likely to be true for both epimers of 7αH,3O-CA where the % free form was in excess of 100%. The high % can be explained by the imperfect correction, even with the use of an authentic isotope-labelled standard, to account for loss of 7α-hydroxy-4-en-3-one analyte in strong base.

In summary, in addition to the 17 free sterols quantified in section *3.1.3.1,* 12 sterols, including oxysterols and cholestenoic acids were quantified as “total sterol” representing the sum of non-esterified and esterified sterols. Semi-quantitative measurements were made on a further 5 sterols.

### 3.3 Sterols including oxysterols and sterol-acids in CSF

Non-esterified oxysterols are present in CSF at much lower concentrations (<1 ng/mL) than in plasma (ng/mL) [14,39,40]. However, cholestenoic acids are comparatively abundant in CSF [39,40,48]. Thus, if CSF material is limited in its availability, it may be optimal to analyse cholestenoic acids as the non-esterified entities in a volume of non-hydrolysed CSF and oxysterols following hydrolysis in a separate volume.

First, we confirmed the linearity of eq. (1) in CSF (100 μL) using 20 μL of OxysterolSPLASH in a standard addition experiment over a 5-fold concentration range (Supplemental Table S3B). All analytes targeted by OxysterolSPLASH gave R^2^ ≥ 0.99, except low abundance 7α,25-diHC (R^2^ ≥ 0.98). This experiment also provided a value for experimental accuracy (>80% in all cases), where accuracy is defined as the agreement between actual measured concentration and that derived from eq. (1), and apparent extraction efficiency (99%–122%). Accuracy was least good for 7α,25-diHC and 7α,(25R/S)26-diHC where the concentration of internal standard is low and for 7α-HC that can be formed *ex vivo* from cholesterol by autoxidation. Again, we exploited technical dilutions of prepared samples to estimate a lower limit of quantification as the lowest concentration at which the measured concentration of analyte differs from the calculated concentration by less than 30%.

Additional experiments were performed in which the volumes of CSF and OxysterolSPLASH were reduced. There was consequent reduction in signal for both analytes and standard and these experiments were not perused further.

#### 3.3.1 Non-esterified sterols including oxysterols and sterol-acids in CSF

**Fig. 4:**
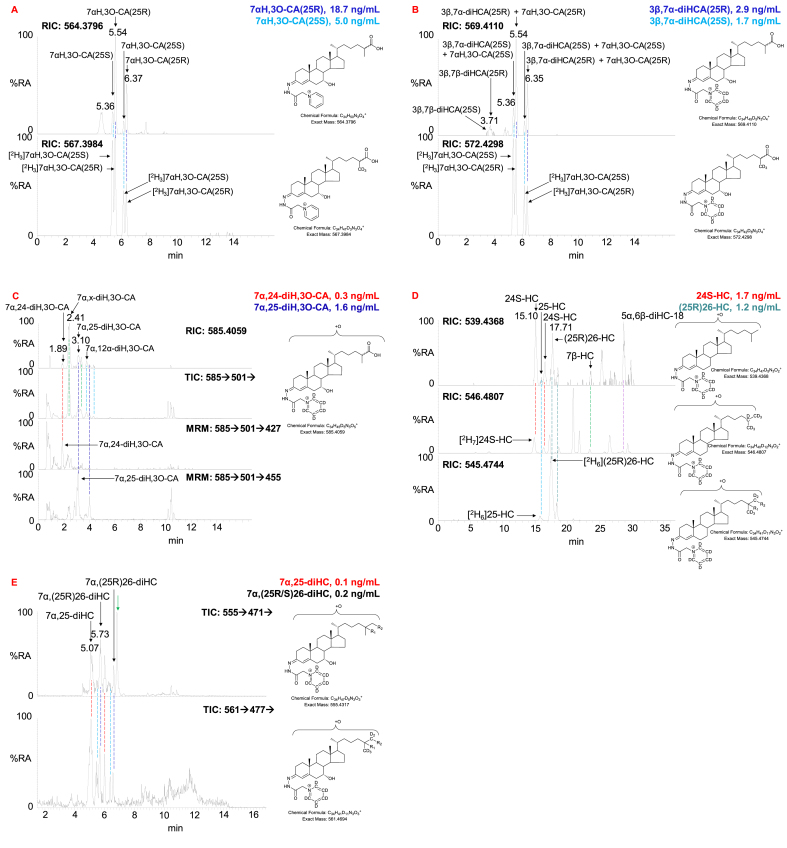
LC-MS separation of GP-derivatised cholestenoic acids, and mono- and dihydroxycholesterols in CSF. (A) RIC for the [M]^+^ ions of (upper panel) 7αH,3O-CA(25R/S) (564.3796 ± 5 ppm) found in CSF, and (lower panel) [^2^H_3_]7αH,3O-CA(25R/S) (567.3984 ± 5 ppm). (B) RIC for the [M]^+^ ions of (upper panel) 7αH,3O-CA(25R/S) + 3β,7α-diHCA(25R/S) (569.4110 ± 5 ppm) found in CSF, and (lower panel) [^2^H_3_]7αH,3O-CA(25R/S) (572.4298 ± 5 ppm). In (A) the derivatisation agent was [^2^H_0_]GP and in (B) [^2^H_5_]GP. (C) RIC for the [M]^+^ ions of diH,3O-CA isomers (585.4059 ± 5 ppm) found in CSF (upper panel), note the triHCA equivalents are absent. TIC for the MS^3^ fragmentation (585.4 → 501.3→) for diH,3O-CA isomers (2nd panel). MRM (585.4 → 501.3→427.3) targeting 7α,24-diH,3O-CA (3rd panel), and MRM (585.4 → 501.3→455.3) targeting 7α,25-diH,3O-CA (bottom panel). See Supplemental Figures S4P & S4Q for relevant fragmentation schemes. Chromatograms in (A–C) are from non-hydrolysed CSF. (D) RIC of the [M]^+^ ions of monohydroxycholesterols (539.4368 ± 3 ppm) found in CSF (upper panel). RIC (546.4807 ± 3 ppm) for [^2^H_7_]24R/S-HC, [^2^H_7_]7β-HC, [^2^H_7_]7α-HC and dehydrated [^2^H_7_]5α,6β-diHC (central panel). RIC (545.4744 ± 3 ppm) for [^2^H_6_]25-HC and [^2^H_6_](25R)26-HC (lower panel). (E) TIC for the MS^3^ fragmentations (555.4 → 471.4→) of 7α,25-diHC and 7α,(25R/S)26-diHC found in CSF (upper panel) and for the fragmentations (561.5 → 477.4→) of [^2^H_6_]7α,25-diHC and [^2^H_6_]7α,(25R/S)26-diHC. Chromatograms (D & E) are for hydrolysed CSF. Coloured dashed lines indicate the coincidence of peaks of the same oxysterol. All chromatograms were recorded over a 17 min gradient.

Using 20 μL of OxysterolSPLASH to provide the isotope-labelled standard, the 25R and 25S epimers of 7αH,3O-CA can be reliably quantified from 100 μL of non-hydrolysed CSF (%CV ≤ 20%) and by considering data in fraction-A and fraction-B, so can the individual epimers of 3β,7αH-diHCA (%CV < 20%, Table 2, Fig. 4A and B). Increasing the volume of CSF to 200 μL gave data of similar precision. Cholesterol is likewise measured by reference to added isotope-labelled standard with acceptable precision (%CV <10%). We did not attempt to quantify 7α-HC, 7β-HC, 7-OC or 5α,6β-diHC in CSF, as they can be formed by *ex vivo* autoxidation of cholesterol. Even a small degree of *ex vivo* autoxidation will introduce major errors in quantification when the endogenous molecules are of low abundance.

**Table 2:**
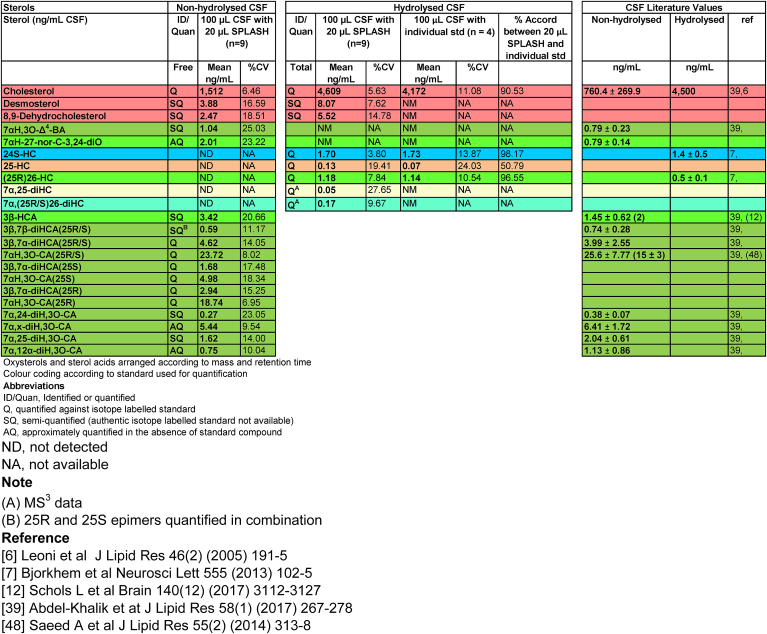
Analysis of non-hydrolysed and hydrolysed CSF.

In addition to the 4 cholestenoic acids and cholesterol quantified by direct reference to isotope-labelled surrogates, we also obtained semi-quantitative data on another 5 sterol-acids and two sterols in the absence of authentic isotope-labelled standards (Fig. 4B and C), and approximate quantification on a further two sterol-acids and one oxysterol, partially identified in the absence of internal standards (Table 2).

#### 3.3.2 Esterified sterols and oxysterols in CSF

As in plasma, oxysterols found in CSF are present as free alcohols and esterified to fatty acids. By extracting oxysterols in 0.35 M KOH in ethanol the esters are hydrolysed, allowing measurement of “total” oxysterols. In this way 24S-HC, 25-HC, (25R)26-HC and 7α,(25R/S)26-diHC could be reliably measured (%CV ≤ 20%) from 100 μL of CSF (Table 2, Fig. 4D & E). 7α,25-diHC could also be measured but at lower precision (%CV < 30%). Other 7-hydroxy-5-ene or 7-hydroxy-4-en-3-one compounds were not reliably measured in the absence of the exact isotope labelled surrogate.

The current data set for 100 μL of CSF and 20 μL of OxysterolSPLASH was compared to data generated using the same CSF sample but exploiting quantitative isotope-labelled standards [^2^H_6_]24R/S-HC and [^2^H_7_]cholesterol. Using [^2^H_6_]24R/S-HC as an internal standard for 24S-HC, 25-HC and (25R)26-HC, the agreement between the methods was good for 24S-HC and (25R)26-HC (>96%) but only moderate for low abundance 25-HC (51%). The agreement of cholesterol measurements was also good at 91%.

### 3.4 Quantification of oxysterols in patient samples

#### 3.4.1 ACOX2

**Fig. 5:**
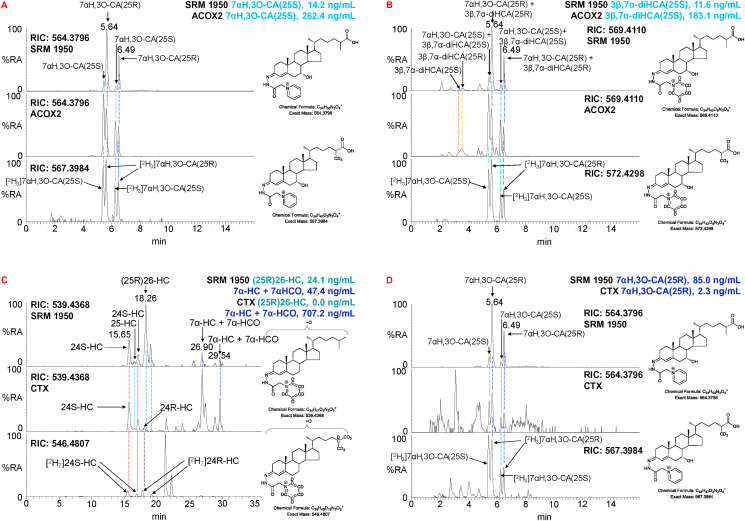
LC-MS separation of GP-derivatised cholestenoic acids, monohydroxycholesterols and monohydroxycholestenones in plasma samples representative of the inborn errors of cholesterol metabolism ACOX2 deficiency and CTX. (A) RIC (564.3796 ± 5 ppm) for [M]^+^ ions corresponding to 7αH,3O-CA(25R/S) in NIST SRM 1950 plasma (upper panel), from a patient suffering from ACOX2 deficiency (central panel), and the RIC (567.3984 ± 5 ppm) corresponding to the [M]^+^ ion of [^2^H_3_]7αH,3O-CA(25R/S) (lower panel). (B) RIC (569.4110 ± 5 ppm) for [M]^+^ ions corresponding to 3β,7α-diHCA(25R/S) + 7αH,3O-CA(25R/S) in NIST SRM 1950 plasma (upper panel), from a patient suffering from ACOX2 deficiency (central panel), and the RIC (572.4298 ± 5 ppm) corresponding to the [M]^+^ ion of [^2^H_3_]7αH,3O-CA(25R/S) (lower panel). Samples in (A) have been treated with [^2^H_0_]GP and those in (B) with [^2^H_5_]GP. (C) RIC (539.4368 ± 5 ppm) for [M]^+^ ions corresponding to monohydroxycholesterols and monohydroxycholestenones in NIST SRM 1950 plasma (upper panel), from a sample from a patient suffering from CTX (central panel), and the RIC (546.4807 ± 5 ppm) corresponding to the [M]^+^ ion of [^2^H_7_]24R/S-HC (lower panel). (D) RIC (564.3796 ± 5 ppm) for [M]^+^ ions corresponding to 7αH,3O-CA(25R/S) in NIST SRM 1950 plasma (upper panel), from a sample from a patient suffering from CTX (central panel), and the RIC (567.3984 ± 5 ppm) corresponding to the [M]^+^ ion of [^2^H_3_]7αH,3O-CA(25R/S) (lower panel). Coloured dashed lines indicate the coincidence of oxysterols between chromatograms.

Mass spectrometry is an ideal method to diagnose inborn errors of cholesterol metabolism [8]. One such disorder is ACOX2 deficiency [10]. ACOX2 is a peroxisomal enzyme involved in the side-chain shortening of C_27_ to C_24_ acids as part of the bile acid biosynthesis pathways (see Ref. [49] for details of metabolic pathways). Its substrates are CoA thioesters of C_27_ acids with 25S-steriochemistry, which themselves are derived from the corresponding CoA thioesters with 25R-steriochemistry in a reaction catalysed by alpha-methylacyl-CoA racemase (AMACR) [50]. Plasma analysis of bile acid precursors reveals C_27_ acids rather than their CoA thioesters, hence, it is anticipated that 3β,7α-diHCA(25S) and 7αH,3O-CA(25S) should be elevated in plasma from patients with ACOX2 deficiency. The availability of the [^2^H_3_]-labelled forms 7αH,3O-CA(25S) and 7αH,3O-CA(25R) allows quantification using the EADSA method of these two endogenous acids and also of 3β,7α-diHCA(25S) and 3β,7α-diHCA(25R) (Table 1 and Fig. 5A & B). In normal plasma the two 25R-epimers are about three and six times more abundant than the 25S-epimers, but in plasma from the ACOX2 deficient patient the 25S-epimers are more abundant, confirming the biochemical phenotype of the patient. It is also noteworthy that the ratio of 3β,7β-diHCA(25R) to 3β,7β-diHCA(25S) in an ACOX2 heterozygote is seven, while in the ACOX2 deficient patient only about two (Table 1). This suggests that 3β,7β-diHCA(25S) as the Co-A thioester is a substrate for ACOX2, which provides a route to side-chain shortened 7β-hydroxy C_24_ bile acids, usually characterised as secondary bile acids [43].

ACOX2 deficiency is one of a number of peroxisomal disorders which present to differing extents with cholestatic liver disease in infants and children [8,51]. It is known that 3β-hydroxy-5-ene and 3-oxo-4-ene C_24_ acids can inhibiting the bile acid export pump [52] and we speculate that the corresponding C_27_ acids may similarly inhibit the export pump and contribute to infantile/childhood cholestasis in peroxisomal disorders. It will be interesting to study if infants with these high C_27_ acids are those that develop cholestasis.

#### 3.4.2 CTX

CTX results from a deficiency of CYP27A1 the enzyme that introduces the (25R)26-hydroxy and (25R)26-carboxylate functions to the sterol skeleton [8], see Ref. [49] for details of metabolic pathways. The result is an absence of (25R)26-HC in plasma and an elevation in 7α-HCO [31,53]. This is evident in Fig. 5C which shows a RIC for monohydroxycholesterols and monohydroxycholestenones in a plasma sample from a CTX patient. Note the absence of a peak corresponding to (25R)26-HC in the RIC for monohydroxycholesterols and that 24R–HC becomes evident without the need to plot a specific MRM chromatogram targeting 24R/S-HC cf. Fig. 2A lower panel. The availability of both the [^2^H_7_]24R–HC and [^2^H_7_]24S-HC standards allows the definitive identification of these epimers in human plasma and also their quantification. A similar pattern of monohydroxycholesterols was revealed upon analysis of CSF from CTX patients following hydrolysis. It is also of interest to explore the RIC of 7αH,3O-CA (Fig. 5D). Surprisingly, both 25R and 25S epimers are present in the CTX sample at about equal levels, in stark contrast to the situation in the NIST SRM 1950 sample where the 7αH,3O-CA(25R) epimer is dominant (Fig. 5D). This finding will be discussed in more detail in a future report.

## 4 Discussion

Stable isotope dilution MS with the use of authentic isotope-labelled standards represents the most reliable method for sterol quantification [18,19]. Here we have utilised a recently introduced commercial mixture of standards (OxysterolSPLASH) to make quantitative measurements on the NIST SRM 1950 plasma sample. Unsurprisingly, we achieve good agreement when utilising the standard mix or when using an in-house mixture of isotope-labelled standards. The data generated in this study for hydrolysed plasma can be compared to that provided by NIST for cholesterol and to work from McDonald et al. who measured cholesterol and other oxysterols [22,45]. The cholesterol concentration determined in the current study 1.541 mg/mL agrees well with both the NIST value of 1.514 mg/mL and that of McDonald et al. 1.45 mg/mL [22,45]. Similarly, there is good agreement with the values determined here and those by McDonald et al. for most oxysterols(Table 1). Although we took considerable care to minimise *ex vivo* autoxidation of cholesterol and avoid artefactual formation of oxysterols, this can never be fully achieved when samples are prepared in air, and this is reflected in the poorer performance of the analytical method in terms of precision for 7β-HC, 7-OC and 5α,6β-diHC, and in the lesser agreement between measured values when using different batches of standard also for 7α-HC. 7β-HC, 7α-HC and 7-OC can all be formed by non-enzymatic free radical autoxidation reactions [49,54]. This is also true of 5,6-epoxycholesterol the *ex vivo* precursor of 5α,6β-diHC. Although not using the same plasma it is interesting to compare the % of free sterol determined here for NIST SRM 1950 and by Dzeletovic et al. in their classic study where 31 plasma samples were investigated (Table 1) [19]. In both studies the % of free 24S-HC was about 25%, (25R)26-HC about 10%, 7α-HC about 20%, while values for 7-OC were higher at 30–60%.

In the present study we have “deep mind” the NIST SRM 1950 plasma in terms of sterol identification and quantification (see Table 1 and Supplemental Table S1). We only report absolute quantification for those sterols for which an isotope-labelled authentic standard was included. This gave data for 17 sterols, with another 8 sterols semi-quantified without using an authentic isotope-labelled standard, while one further sterol was approximately quantified but only partially identified. In addition, 7 other sterols were identified but not quantified while 8 additional sterols were partially identified. While this study covers most of the cholesterol metabolites routinely analysed in plasma [46], many more are present at lower levels and may only be revealed in patients suffering from inborn errors of cholesterol metabolism, biosynthesis or transport [[24], [25], [26], [27], [28],^55^,^56^]. For comparison McDonald and colleagues have also “mined” NIST SRM 1950 plasma by LC-MS for oxysterols. In one study they quantified 8 oxysterols, both in the non-esterified form and as the total of non-esterified plus esterified forms [3], and in a later study 10 oxysterols, cholesterol and 6 precursors as the combination of non-esterified and esterified forms [45].

Besides analysing plasma, we have also explored the use of the standard mix to quantify oxysterols in a QC sample of CSF. As levels of most non-esterified oxysterols are low in CSF (<1 ng/mL) [14,39,40,57] we have analysed CSF in a non-hydrolysed and hydrolysed form. The non-hydrolysed form reveals non-esterified cholestenoic acids which are relatively abundant (Table 2), while the hydrolysed sample reveals oxysterols which are released as alcohols from their fatty acyl esters by strong base. It is of interest to note the comparatively high levels of both 25R and 25S epimers of 7αH,3O-CA in the QC CSF sample. In our previous studies, only the combined value for both epimers had been measured [39,40,57] using [^2^H_7_]24R/S-HC as the internal standard. Saeed et al. reported the concentration of 7αH,3O-CA in CSF samples from patients with headache, suffering from Alzheimer’s disease or from vascular dementia to be about 15 ng/mL [48], which is in good agreement with that reported here of about 20 ng/mL for our QC sample. Importantly, as in the current study, Saeed et al. used an authentic isotope-labelled standard [48]. They used [25,27,27,27-^2^H_4_]7αH,3O-CA which should have exclusively 25R-stereochemistry as it was derived by CYP27A1 oxidation of [25,26,26,26,27,27,27-^2^H_7_]7α-HCO [48]. Saeed et al. emphasised the importance of the use of an authentic isotope-labelled standard, which is particularly important for compounds with a 7-hydroxy-3-oxo-4-ene structure that are labile to both acid and base catalysed dehydration [43,48].

In the hydrolysed CSF sample we analysed 24S-HC, 25-HC, (25R)26-HC, 7α,25-diHC and 7α,(25R/S)26-diHC and the values we report for our QC sample are in general agreement with those in the literature for 24S-HC and (25R)26-HC [7], we could not find literature values for 25-HC, 7α,25-diHC or 7α,(25R/S)26-diHC following base hydrolysis [58,59]. There appear to be few reported values for other oxysterols in CSF. In the current study we did not analyse 7α-HC, 7β-HC or 5α,6β-HC due to the presence of late eluting contaminants resulting from the hydrolysis of other lipids. In previous studies we have measured monohydroxycholesterols in non-hydrolysed samples, however, to achieve this goal we needed to pre-concentrate samples [39,40], something we have not done in this study. It should be noted, that at the low levels of oxysterols in non-hydrolysed CSF (<0.1 ng/mL) there is the possibility of significant analyte loss by absorption into plastics. To avoid this Sidhu et al. have suggested addition of 2.5% 2-hydroxypropyl-β-cyclodextrin to CSF during collection [14].

Finally, with respect to the drive of the lipidomic community for standardisation [60], we have made our best effort to report the figures of merit of the current methodology in terms of lower limit of quantification, linearity of response, apparent extraction efficiency, accuracy and precision (see Supplemental Table S3 and Table 1). We also make our data publicly available in a data repository (OFS, Center for Open Science, https://osf.io/5ce3p/).

In summary, we report here the absolute and semi-quantification of sterols, including oxysterols and cholestenoic acids in NIST SRM 1950 plasma and in a laboratory QC CSF sample. Where available, the data generated is in good agreement with other studies. The current report extends the range of sterols that can be routinely measured in plasma and CSF samples.

## Declaration of competing interest

The authors declare the following financial interests/personal relationships which may be considered as potential competing interests: WJG and YW are listed as inventors on the patent “Kit and method for quantitative detection of steroids” US9851368B2. WJG, EY and YW are shareholders in CholesteniX Ltd.
