# Supplementary material for: Deep mining of oxysterols and cholestenoic acids in human plasma and cerebrospinal fluid: Quantification using isotope dilution mass spectrometry
Source: Anal Chim Acta. 2021 Apr 15;1154:338259. doi: 10.1016/j.aca.2021.338259 (PMC7988461; doi:10.1016/j.aca.2021.338259)
Supplement: Multimedia component 1 [file mmc1.pdf]

SUPPLEMENTAL MATERIAL

Deep Mining of Oxysterols and Cholestenoic Acids in Human Plasma and Cerebrospinal Fluid: Quantification using Isotope Dilution Mass Spectrometry

Eylan Yutuc[1], Alison L. Dickson[1], Manuela Pacciarini[1], Lauren Griffiths[1], Paul R.S. Baker[2], Lisa Connell[2], Anders Öhman[3], Lars Forsgren[4], Miles Trupp[4], Sílvia Vilarinho[5], Youssef Khalil[6], Peter T. Clayton[6], Sinan Sari[7], Buket Dalgic[7], Philip Höflinger[8], Ludger Schöls[8,9], William J. Griffiths[1] and Yuqin Wang[1]

[1]Swansea University Medical School, ILS1 Building, Singleton Park, Swansea SA2 8PP, Wales, UK

[2]Avanti Polar Lipids Inc, Alabaster, AL, USA

[3]Department of Integrative Medical Biology, Umeå University, SE-901 87 Umeå, Sweden

[4]Department of Clinical Science, Neurosciences, Umeå University, SE-901 85 Umeå, Sweden

[5]Departments of Internal Medicine, Section of Digestive Diseases, and of Pathology, Yale University School of Medicine, New Haven, CT, USA

[6]Inborn Errors of Metabolism, Genetics and Genomic Medicine, UCL Great Ormond Street Institute of Child Health, 30 Guilford Street, London WC1N 1EH, UK

[7]Department of Pediatrics, Division of Gastroenterology, Gazi University Faculty of Medicine, Ankara, Turkey

[8]Department of Neurology and Hertie Institute for Clinical Brain Research, University of Tübingen, Tübingen, Germany

[9]German Center for Neurodegenerative Diseases (DZNE), Tübingen

Supplemental Information

Supplemental Methods

Flowchart 1. Schematic illustration of sample preparation. Single-phase liquid extraction is performed into ethanol containing internal standards in the presence or absence of 0.35 M KOH.

Flowchart 2. Different protocols for single-phase liquid extraction.

Flowchart 3. Flowchart showing sterol identification and quantification routine.

Supplemental Figures and Tables

Figure S1. *Syn* (*Z*) and *anti* (*E*) conformers of the GP-derivative exemplified for 24S-HC. Also shown are 3D minimised energy structures (Chem 3D Pro). MS^2 fragmentation results in loss of the pyridine (Py) group with the formation of the $[M-Py]^+$ fragment ion.

Figure S2. LC-MS separation of GP-derivatised monohydroxycholesterols (HC). (A) Mass spectrometric peaks at *m/z* 546.48 corresponding to the $[M]^+$ ion of $[^2H_7]$24S-HC (upper panel); the $[M+1]^+$ ion of $[^2H_6]$26-HC (lower panel); and unresolved $[M]^+$ ion of $[^2H_7]$24R-HC and the $[M+1]^+$ ion of $[^2H_6]$26-HC (central panel). The structure of the $[M+1]^+$ ion of $[^2H_6]$26-HC is arbitrarily drawn with a ^{13}C at C-21. (B) Upper panel, TIC 534.4→455.4→ for monohydroxycholestenones in plasma. Lower panel TICs 541.5→462.4 for $[^2H_7]$-labelled monohydroxycholesterols.

Figure S3. MS^3 fragmentation of GP-derivatised monhydroxycholesterols in plasma. MS^3 ($[M]^+$→$[M-Py]^+$→) spectra of (A) 24S-HC; (B) 24R-HC; (C) $[^2H_7]$24S-HC; (D) $[^2H_7]$24R-HC; (E)25-HC; (F) $[^2H_6]$25-HC; (G) (25R)26-HC; (H) $[^2H_6]$(25R)26-HC; (I) $[^2H_7]$22R-HC; (J) 22S-HC; (K) $[^2H_7]$22S-HC. The formation of key fragment ions is illustrated in Figure S4A-D.

Figure S4. Key fragmentation routes in the MS^n spectra of GP-derivatised oxysterols. Mechanisms of side-chain cleavage are indicated by purple arrows. (A) 24R/S-HC and $[^2H_6]$24R/S-HC; (B) 25-HC and $[^2H_6]$25-HC; (C) (25R)26-HC and $[^2H_6]$(25R)26-HC; (D) 22R/S-HC and $[^2H_7]$22R/S-HC; (E) 7-HC and $[^2H_7]$7-HC; (F) 6β-HC and $[^2H_7]$6β-HC; (G) 7-OC and $[^2H_7]$7-OC; (H) 12α-HC; (I) 7α,25-diHC and $[^2H_6]$7α,25-diHC; (J) 7α,(25R/S)26-diHC and $[^2H_6]$7α,(25R/S)26-diHC; (K) 7α,12α-diHC; (L) 3β,7-diHCA(25R/S) and $[^2H_3]$3β,7-diHCA(25R/S); (M) 25-D_3 and $[^2H_6]$25-D_3; (N) 3βH-Δ^5-BA and 3β,7-diH-Δ^5-BA; (O) 3β-HCA; (P) 3β,7α,24-triHCA; (Q) 3β,7α,25-triHCA; (R) 3β,7α,12α-triHCA; (S) 3β,25-diHCA; (T) 3β,27-diHCA; (U) 3β,22,25-triHC-24O; (V) 3β,22-diHC-24O.

Figure S5. MS^3 fragmentation of GP-derivatised monohydroxycholesterols and monohydroxycholestenones in plasma. MS^3 ($[M]^+$→$[M-Py]^+$→) spectra of (A) 7β-HC; (B) $[^2H_7]$7β-HC; (C) 7α-HC; (D) $[^2H_7]$7α-HC; (E) 6β-HC (5α,6β-diHC-18), generated from the acid catalysed dehydration of 5α,6β-diHC; (F) $[^2H_7]$6β-HC ($[^2H_7]$5α,6β-18) generated from the acid catalysed dehydration of $[^2H_7]$5α,6β-diHC; (G) 7-OC; (H) $[^2H_7]$7-OC; (I) 7α-HCO; (J) $[^2H_7]$7α-HCO; (K) 12α-HC (no authentic standard). The formation of key fragment ions is illustrated in Figure S4E-H.

Figure S6. LC-MS separation of GP-derivatised dihydroxycholesterols, dihydroxycholestenones (diHCO), dihydroxycholestenoic (diHCA) and hydroxyoxocholestenoic (H,O-CA) acids. (A) RIC for the $[M]^+$ ions of (upper panel) 7α,25-diHC + 7α,25-diHCO and 7α,(25R/S)26-diHC + 7α,(25R/S)26-diHCO (555.4317 ± 5 ppm) found in plasma and (lower panel) $[^2H_6]$7α,25-diHC and $[^2H_6]$7α,(25R/S)26-diHC (561.4694 ± 5 ppm) over a 37 min gradient. (B) RIC of the $[M]^+$ ions of dihydroxycholestenones (550.4003 ± 5 ppm) found in plasma. Upper panel 37 min gradient, lower panel 17 min gradient. (C) RIC for the $[M]^+$ ions of (upper panel) 7αH,3O-CA(25R/S) (564.3796 ± 5 ppm) found in plasma and (lower panel) $[^2H_3]$7αH,3O-CA(25R/S) (567.3984 ± 5 ppm) over a 37 min gradient. (D) RIC for the $[M]^+$

ions of (upper panel) 7αH,3O-CA(25R/S) + 3β,7α-diHCA(25R/S) (569.4110 ± 5 ppm) found in plasma and (lower panel) [$^2H_3$]7αH,3O-CA(25R/S) (572.4298 ± 5 ppm) over a 37 min gradient. Coloured dashed lines indicate the coincidence of oxysterols between chromatograms.

Figure S7. MS^3 fragmentation of GP-derivatised dihydroxycholesterols (diHC) and dihydroxycholestenones (diHCO) in plasma. MS^3 ($[M]^+$→$[M\text{-}Py]^+$→) spectra of (A) 7α,25-diHC + 7α,25-diHCO; (B) [$^2H_6$]7α,25-diHC; (C) 7α,(25S)26-diHC + 7α,(25S)26-diHCO; (D) [$^2H_6$]7α,(25S)26-diHC; (E) 7α,(25R)26-diHC + 7αH,(25R)26-diHCO; (F) [$^2H_6$]7α,(25R)26-diHC; (G) 7α,25-diHCO; (H) 7α,(25R)26-diHCO; (I) 7α,12α-diHC. The formation of key fragment ions is illustrated in Figure S4I – S4K.

Figure S8. MS^3 fragmentation of GP-derivatised dihydroxycholestenoic (diHCA) and hydroxyoxocholestenoic (H,O-CA) acids in plasma. MS^3 ($[M]^+$→$[M\text{-}Py]^+$→) spectra of (A) 3β,7β-diHCA(25R); (B) 3β,7α-diHCA(25S) + 7αH,3O-CA(25S); (C) [$^2H_3$]3β,7α-diHCA(25S); (D) 3β,7α-diHCA(25R) + 7αH,3O-CA(25R); (E) [$^2H_3$]3β,7α-diHCA(25R); (F) 7αH,3O-CA(25S); (G) 7αH,3O-CA(25R); (H) [$^2H_3$]7αH,3O-CA(25R). The formation of key fragment ions is illustrated in Figure S4L.

Figure S9. LC-MS(MS^3) of cholesterol and other lipophilic sterols in plasma. (A) RIC for the $[M]^+$ ions of cholesterol (upper panel, 523.4419 ± 5 ppm), [$^2H_7$]cholesterol (central panel, 530.4858 ± 5 ppm) and isomers of cholestadien-3β-ol (lower panel 521.4262 ± 5 ppm). MS^3 ($[M]^+$→$[M\text{-}Py]^+$→) spectra of (B) cholesterol, (C) [$^2H_7$]cholesterol, (D) desmosterol (24-DHC) and (E) 8(9)-dehydrocholesterol (8-DHC). The formation of key fragment ions is illustrated in (F – H).

Figure S10. LC-MS separation of GP-derivatised 25-hydroxyvitanin D_3 and sterol-acids in plasma. (A) RIC for the $[M]^+$ ions of 25-D_3 (537.4211 ± 5 ppm, upper panel) and [$^2H_6$]25-D_3 (543.4588 ± 5 ppm, lower panel); (B) 3βH-Δ^5-BA (511.3691 ± 5 ppm); (C) 3β,7β-diH-Δ^5-BA and 3α,7α-diH-Δ^5-BA + 7αH,3O-Δ^4-BA (527.3640 ± 5 ppm, upper panel); 7αH,3O-Δ^4-BA (522.3326 ± 5 ppm, lower panel,); (D) 3β-HCA + 3O-CA (553.4161 ± 5 ppm, upper panel,); 3O-CA (548.3847 ± 5 ppm, lower panel); (E - H) 3β,7α,24-triHCA + 7α,24-diH,3O-CA, 3β,7α,25-triHCA + 7α,25-diH,3O-CA and 3β,7α,12α-triHCA + 7α,12α-diH,3O-CA (585.4059 ± 5 ppm, upper panels); (E) Lower panel, 7α,24-diH,3O-CA, 7α,25-diH,3O-CA and 7α,12α-diH,3O-CA (580.3739 ± 5 ppm); (F – H) Lower panels show MRM chromatograms targeting (F) 3β,7α,24-triHCA + 7α,24-diH,3O-CA; (G) 3β,7α,25-triHCA + 7α,25-diH,3O-CA; (H) 3β,7α,12α-triHCA and 7α,12α-diH,3O-CA. Authentic standards are not available for 3β,7α,12α-triHCA and 7α,12α-diH,3O-CA. Coloured dashed lines indicate the coincidence of oxysterols between chromatograms. MS^3 spectra are presented in Figures S11 & S12.

Figure S11. MS^3 fragmentation of GP-derivatised 25-hydroxyvitamin D_3 and sterol acids in plasma. MS^3 ($[M]^+$→$[M\text{-}Py\text{-}H_2O]^+$→) spectra of (A) 25-$D_3$ and (B) [$^2H_6$]25-D_3. MS^3 ($[M]^+$→$[M\text{-}Py]^+$→) spectra of (C) 3βH-Δ^5-BA; (D) 3β,7β-diH-Δ^5-BA; (E) 3β,7α-diH-Δ^5-BA + 7αH,3O-Δ^4-BA; (F) 7αH,3O-Δ^4-BA; (G) 3β-HCA + 3O-CA; (H) 3O-CA. The formation of key fragment ions is illustrated in Figure S4M-O.

Figure S12. MS^3 fragmentation of GP-derivatised of trihydroxycholestenoic (triHCA) and dihydroxy-3-oxocholestenoic acids (diH,3O-CA) in plasma. MS^3 ($[M]^+$→$[M\text{-}Py]^+$→) spectra of (A) 3β,7α,24-triHCA + 7α,24-diH,3O-CA; (B) 3β,7α,25-triHCA + 7α,25-diH,3O-CA; (C) 3β,7α,12α-triHCA + 7α,12α-diH,3O-CA (no authentic standard). The formation of key fragment ions is illustrated in Figure S4P-R.

Figure S13. MS^3 fragmentation of GP-derivatised oxysterols in plasma. MS^3 spectra of the postulated structures (A) 3β,25-diHCA or *3β,25,x-trihydroxycholest-5-en-y-one* (no authentic standards available); (B) *3β,27-diHCA* or 3β,27,x-trihydroxycholest-5-en-y-one (no authentic standards available); (C) 3β,x-diHCA or 3β,x,y-trihydroxycholest-5-en-z-one (no authentic standards available); (D) *3β,22,25-trihydroxycholest-5-en-24-one* or 3β,x-diHCA (no authentic standard); (E) *3β,22-dihydroxycholest-5-en-24-one* or 3β,20-dihydroxycholest-5-en-22-one (no authentic standard); (F) 7α,x-diH,3O-CA (no

authentic standard). The more likely structures are given in *italics.* The formation of key fragment ions is illustrated in Figure S4S-V.

Figure S14. MS^3 fragmentation of GP-derivatised of trihydroxycholestenoic (triHCA) and dihydroxy-3-oxocholestenoic acids (diH,3O-CA) in CSF. MS^3 ($[M]^+ \rightarrow [M-Py]^+ \rightarrow$) spectra of (A) 3β,7α,24-triHCA + 7α,24-diH,3O-CA; (B) 3β,7α,25-triHCA + 7α,25-diH,3O-CA; (C) 3β,7α,12α-triHCA + 7α,12α-diH,3O-CA (no authentic standard) and (D) 3β,7α,x-triHCA + 7α,x-diH,3O-CA. The formation of key fragment ions is illustrated in Figure S4P-R.

Table S1. Systematic and common names of sterols including oxysterols and sterol-acids identified or partially identified in the current study. Supplier of reference standards are indicated when available.

Table S2. Figures of merits when varying amount of OxysterolSPLASH at a plasma volume of 100 µL.

Table S3. Figures of merit revealed by standards additions to (A) 100 µL of plasma using 50 µL of OxysterolSPLASH and (B) 100 µL CSF using 20 µL of OxysterolSPLASH.

Table S4. Diagnostic fragment ions and simple rules for structure determination of GP-derivatised sterols.

Flowchart 1

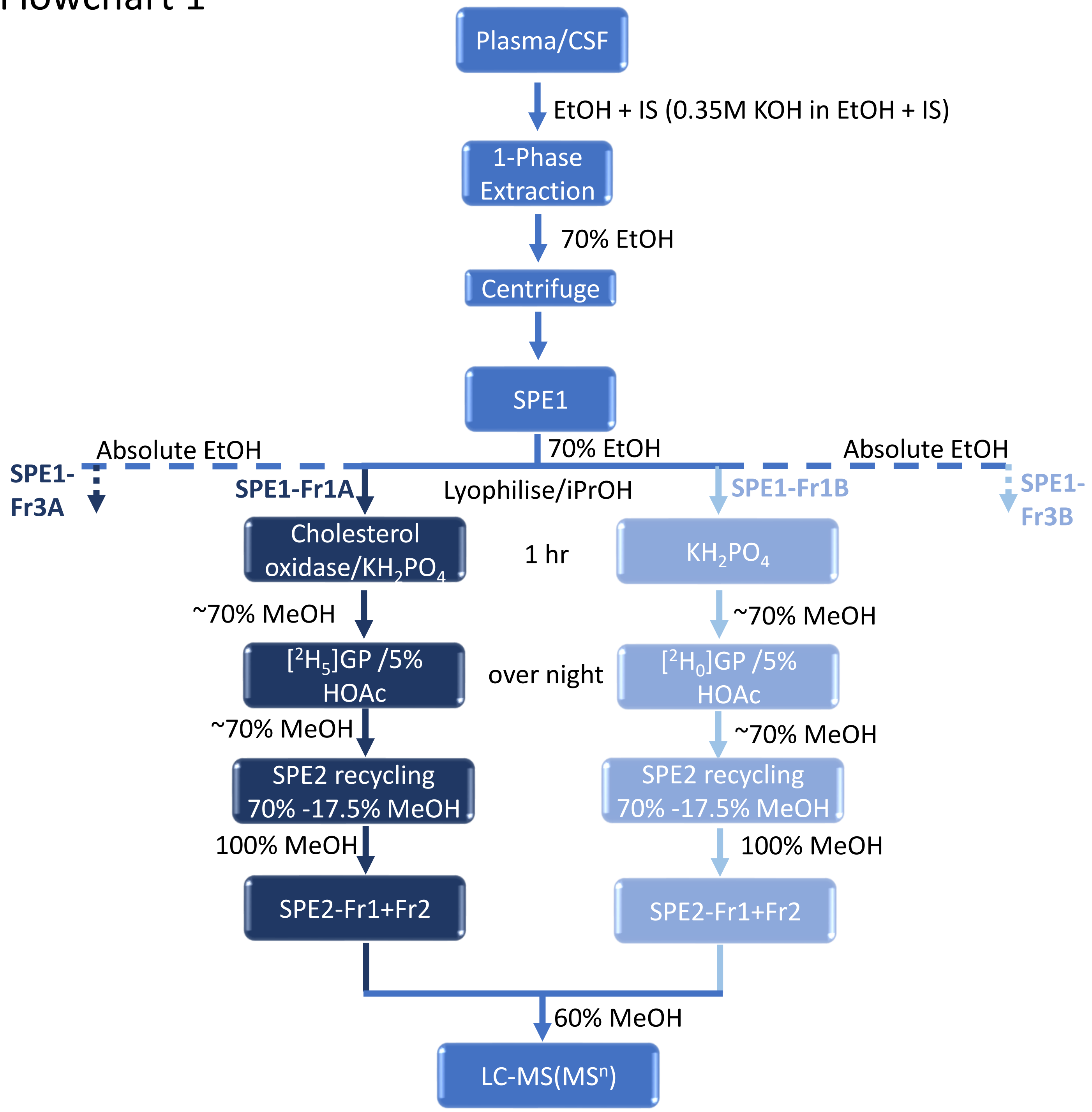

Schematic illustration of sample preparation. Single-phase liquid extraction is performed into ethanol containing internal standards in the presence or absence of 0.35 M KOH

Flowchart 2. Different protocols for single-phase liquid extraction.

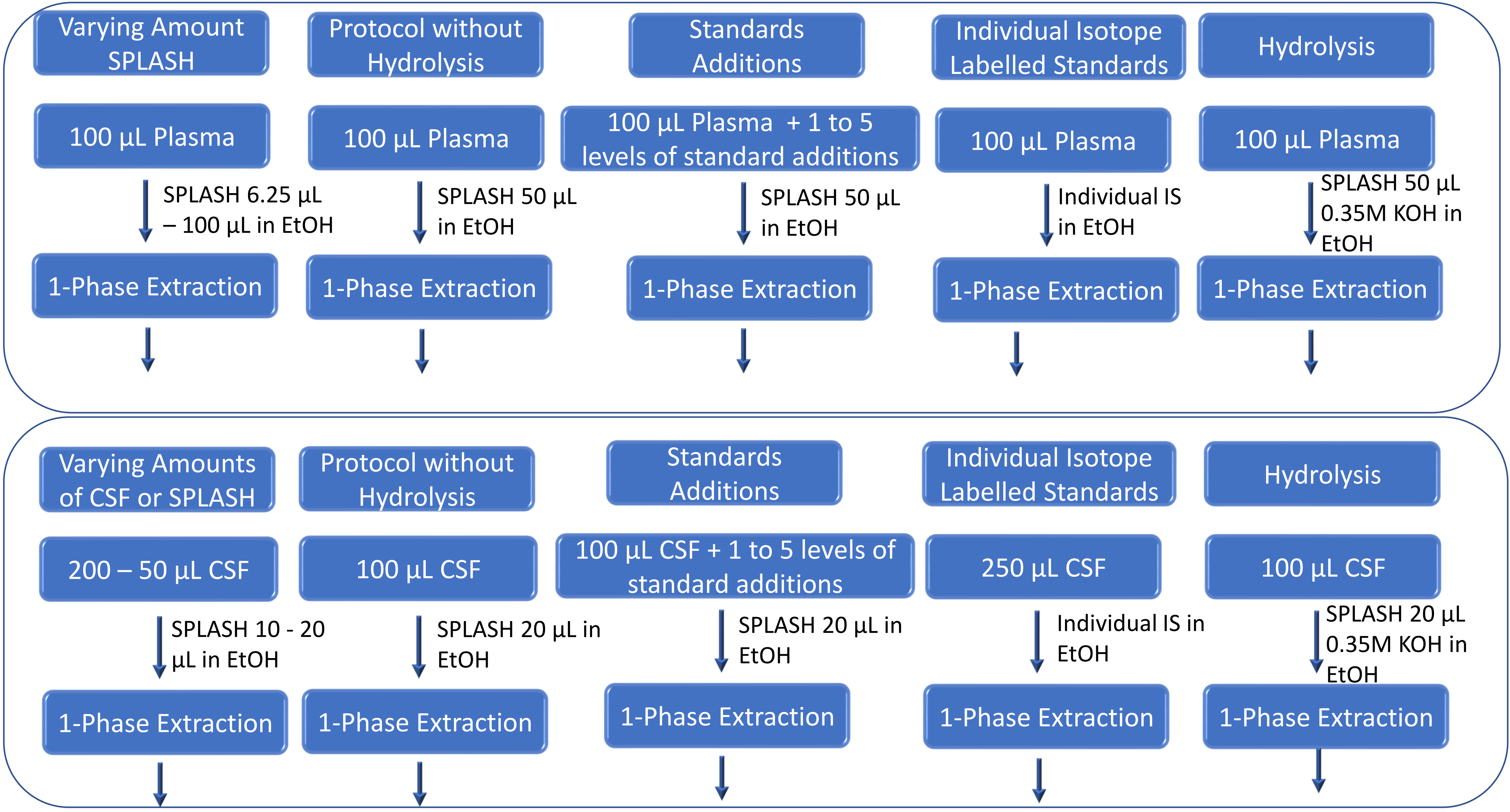

Flowchart 3

1. Record high resolution accurate mass spectra via LC-MS
2. Generate RICs for potential oxysterols, cholestenoic and cholenoic acids
3. Record MS^3 spectra on candidate sterols
4. Postulate structures from retention time data, accurate mass measurement and MS^3 spectra
5. Identify structures where possible against reference standards or library thereof
6. Where standards not available postulate structures to reveal a "presumptive" identification
7. Perform absolute quantification, where possible, on identified structures against exact isotope labelled surrogates
8. If absolute quantification not possible perform semi-quantification against isotope labelled structural analogue
9. In the absence of authentic standard perform approximate quantification against isotope labelled structural analogue

Flowchart showing sterol identification and quantification routine

S1

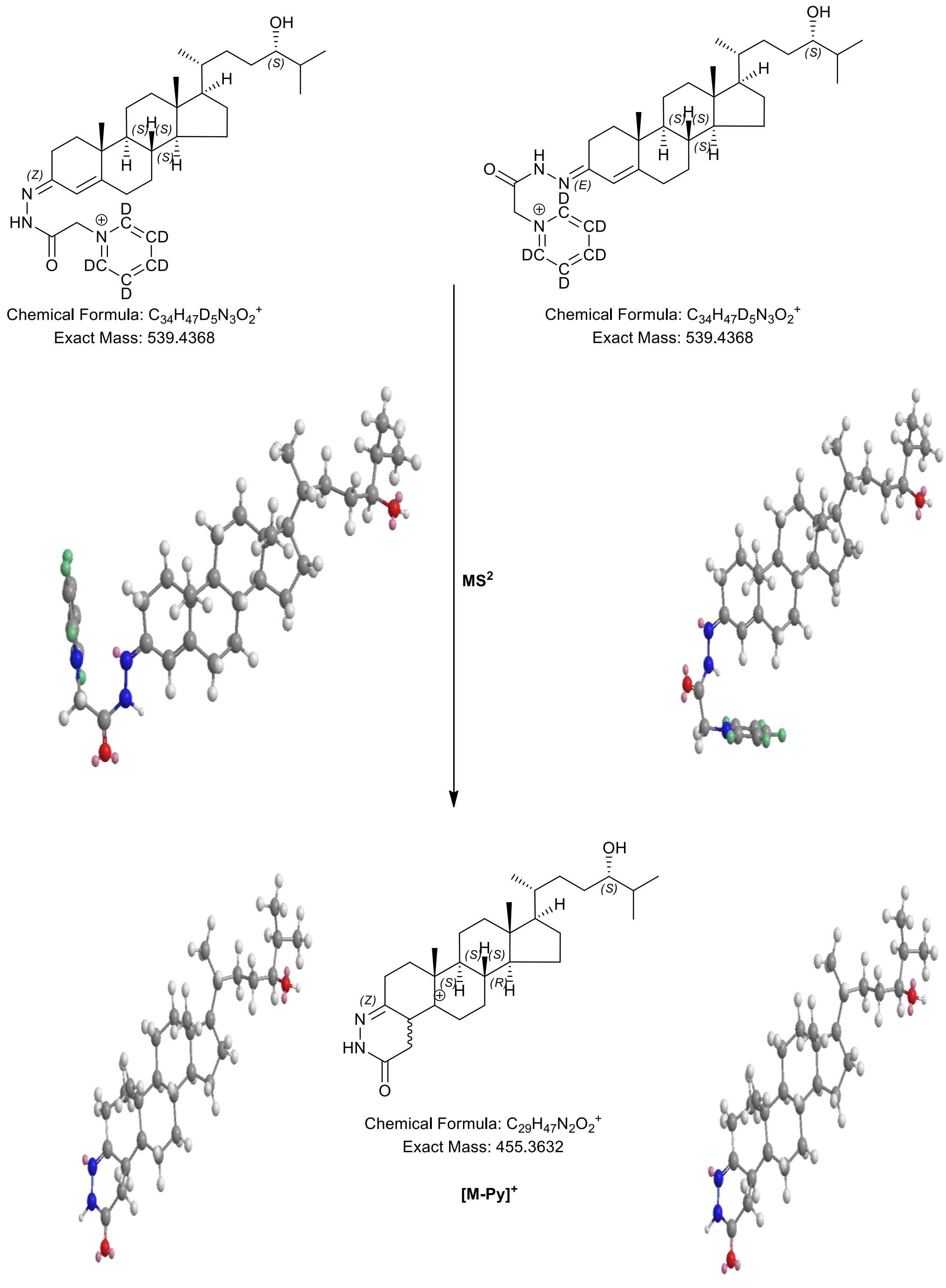
Chemical Formula: C34H47D5N3O2+
Exact Mass: 539.4368
Chemical Formula: C34H47D5N3O2+
Exact Mass: 539.4368
MS2
Chemical Formula: C29H47N2O2+
Exact Mass: 455.3632
[M-Py]+

S2A

EY_191014_100uL-NIST-QC_100uL-OxySpla... 10/15/19 02:44:12
100uL NIST (2018) + 100ul OxysterolSPLASH + 10ng 22S-HCO-D7 + 20u[illegible]

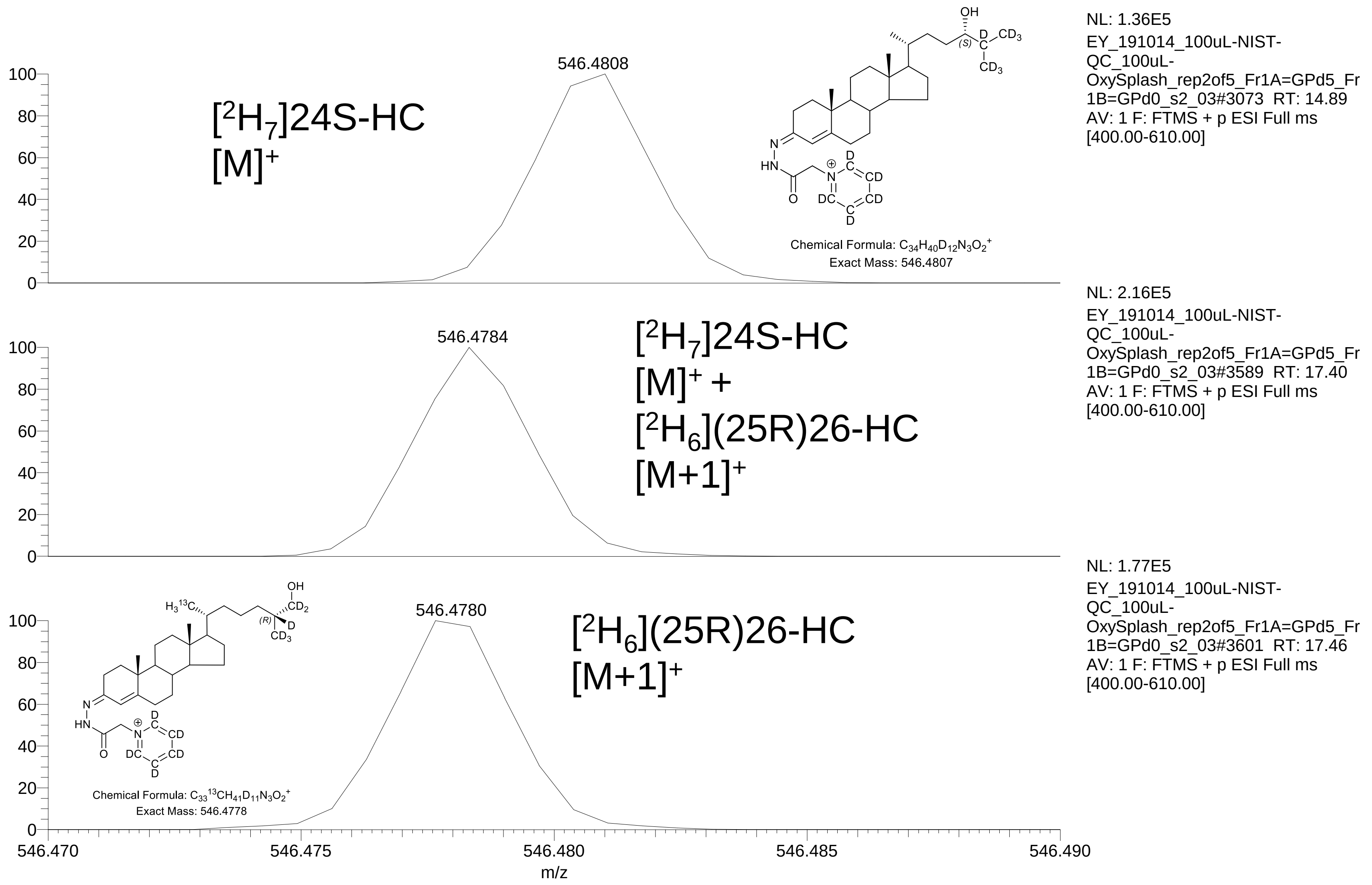

S2B

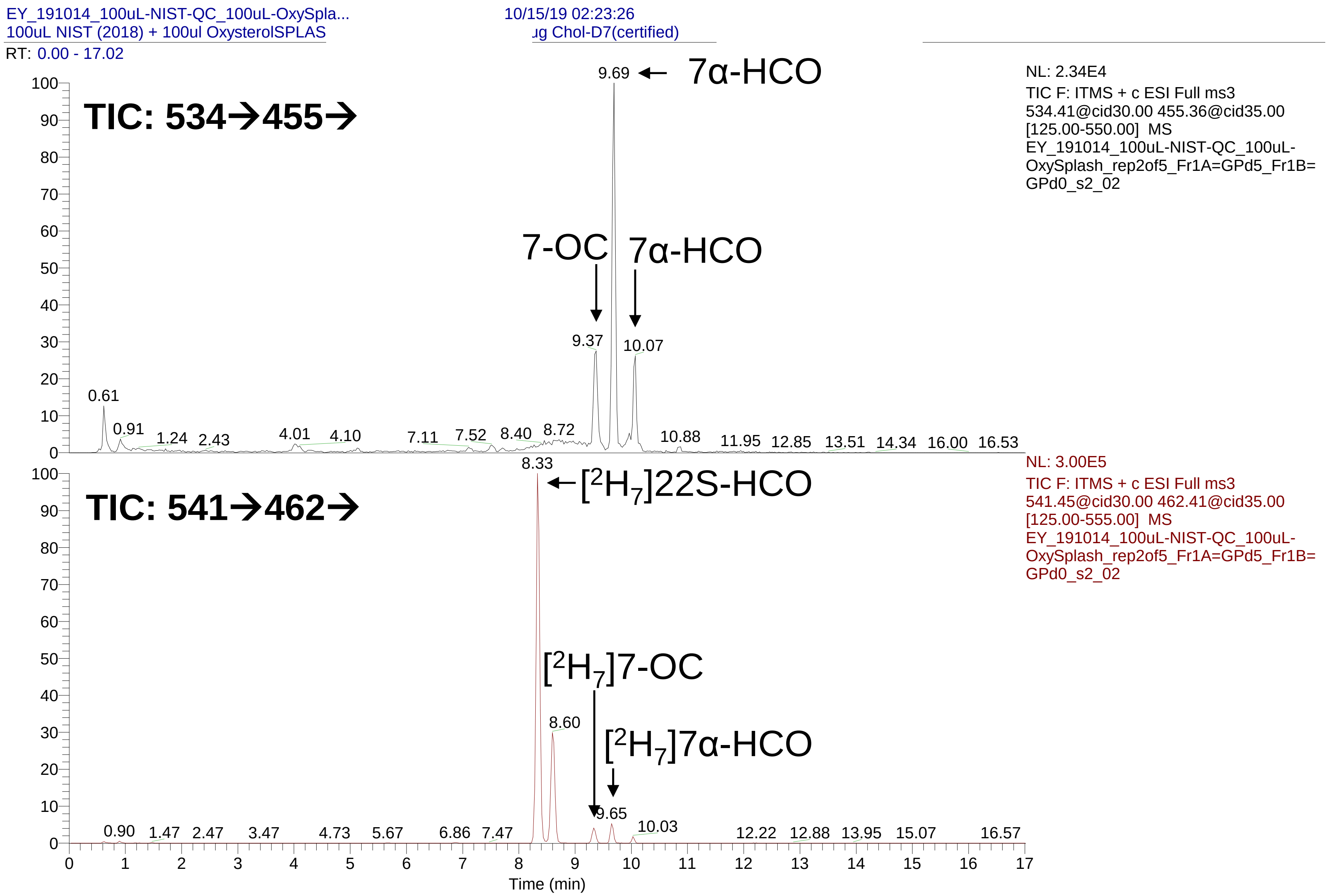
EY_191014_100uL-NIST-QC_100uL-OxySpla...
100uL NIST (2018) + 100ul OxysterolSPLAS
10/15/19 02:23:26
ɹg Chol-D7(certified)
RT: 0.00 - 17.02
TIC: 534→455→
7α-HCO
7-OC
7α-HCO
NL: 2.34E4
TIC F: ITMS + c ESI Full ms3 534.41@cid30.00 455.36@cid35.00 [125.00-550.00] MS EY_191014_100uL-NIST-QC_100uL-OxySplash_rep2of5_Fr1A=GPd5_Fr1B=GPd0_s2_02
TIC: 541→462→
[2H7]22S-HCO
[2H7]7-OC
[2H7]7α-HCO
NL: 3.00E5
TIC F: ITMS + c ESI Full ms3 541.45@cid30.00 462.41@cid35.00 [125.00-555.00] MS EY_191014_100uL-NIST-QC_100uL-OxySplash_rep2of5_Fr1A=GPd5_Fr1B=GPd0_s2_02
Time (min)

S3A

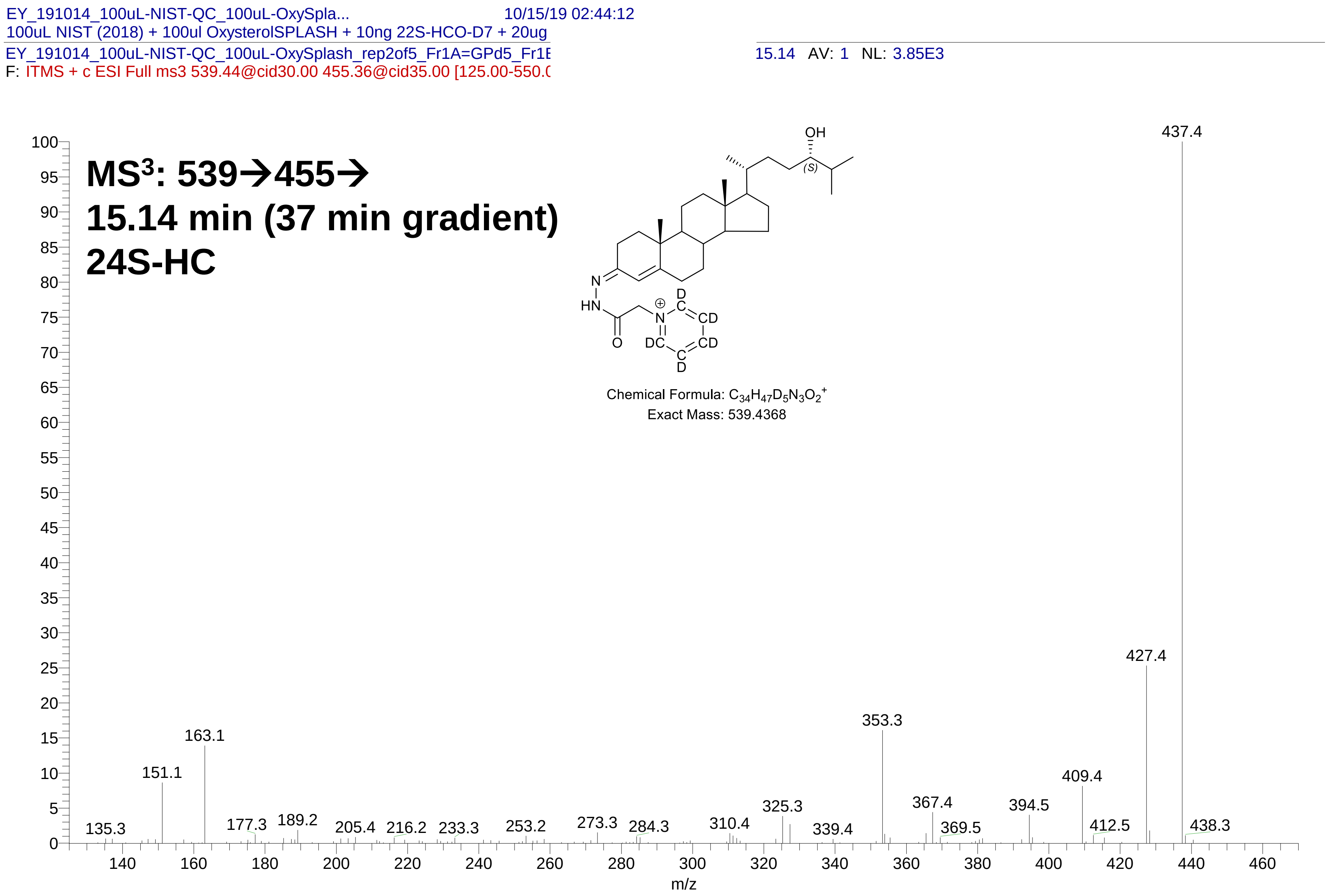
EY_191014_100uL-NIST-QC_100uL-OxySpla...
10/15/19 02:44:12
100uL NIST (2018) + 100ul OxysterolSPLASH + 10ng 22S-HCO-D7 + 20ug
EY_191014_100uL-NIST-QC_100uL-OxySplash_rep2of5_Fr1A=GPd5_Fr1E
15.14 AV: 1 NL: 3.85E3
F: ITMS + c ESI Full ms3 539.44@cid30.00 455.36@cid35.00 [125.00-550.0
MS³: 539→455→
15.14 min (37 min gradient)
24S-HC
OH
(S)
N
HN
O
D
C
CD
CD
DC
C
D
N
Chemical Formula: $C_{34}H_{47}D_5N_3O_2^+$
Exact Mass: 539.4368
437.4
427.4
353.3
163.1
151.1
409.4
325.3
367.4
394.5
135.3
177.3
189.2
205.4
216.2
233.3
253.2
273.3
284.3
310.4
339.4
369.5
412.5
438.3
m/z

S3B

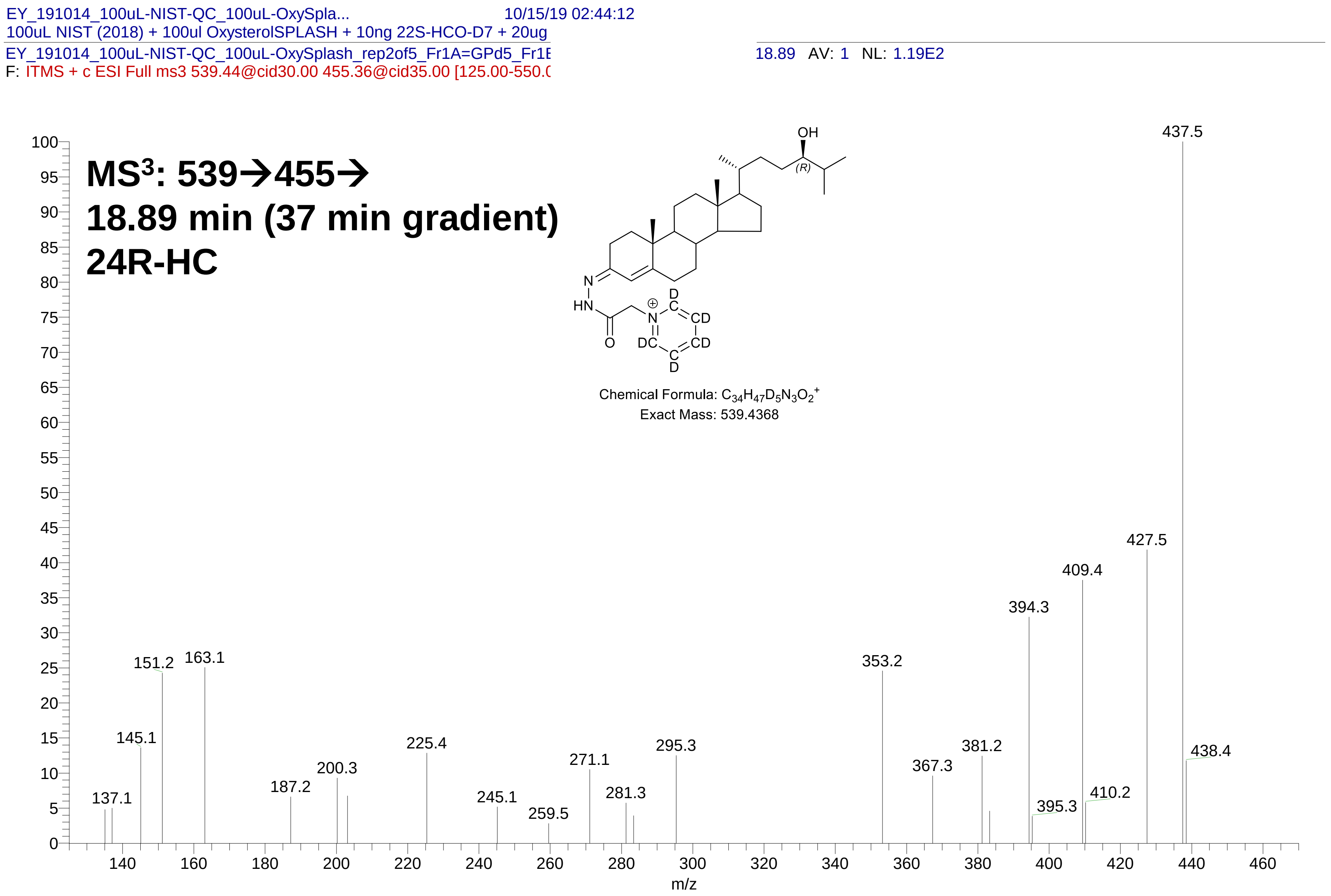

EY_191014_100uL-NIST-QC_100uL-OxySpla... 10/15/19 02:44:12
100uL NIST (2018) + 100ul OxysterolSPLASH + 10ng 22S-HCO-D7 + 20ug
EY_191014_100uL-NIST-QC_100uL-OxySplash_rep2of5_Fr1A=GPd5_Fr1E
F: ITMS + c ESI Full ms3 539.44@cid30.00 455.36@cid35.00 [125.00-550.0
18.89 AV: 1 NL: 1.19E2
MS3: 539→455→
18.89 min (37 min gradient)
24R-HC
OH
(R)
N
HN
O
D
C
CD
CD
DC
N
D
Chemical Formula: C34H47D5N3O2+
Exact Mass: 539.4368
437.5
427.5
409.4
394.3
353.2
151.2
163.1
145.1
225.4
295.3
381.2
438.4
271.1
367.3
200.3
187.2
137.1
245.1
281.3
259.5
395.3
410.2
m/z

S3C

EY_191014_100uL-NIST-QC_100uL-OxySpla... 10/15/19 02:44:12
100uL NIST (2018) + 100ul OxysterolSPLASH + 10ng 22S-HCO-D7 + 20ug
EY_191014_100uL-NIST-QC_100uL-OxySplash_rep2of5_Fr1A=GPd5_Fr1E
F: ITMS + c ESI Full ms3 546.48@cid30.00 462.41@cid35.00 [125.00-555.0

14.92 AV: 1 NL: 1.24E4

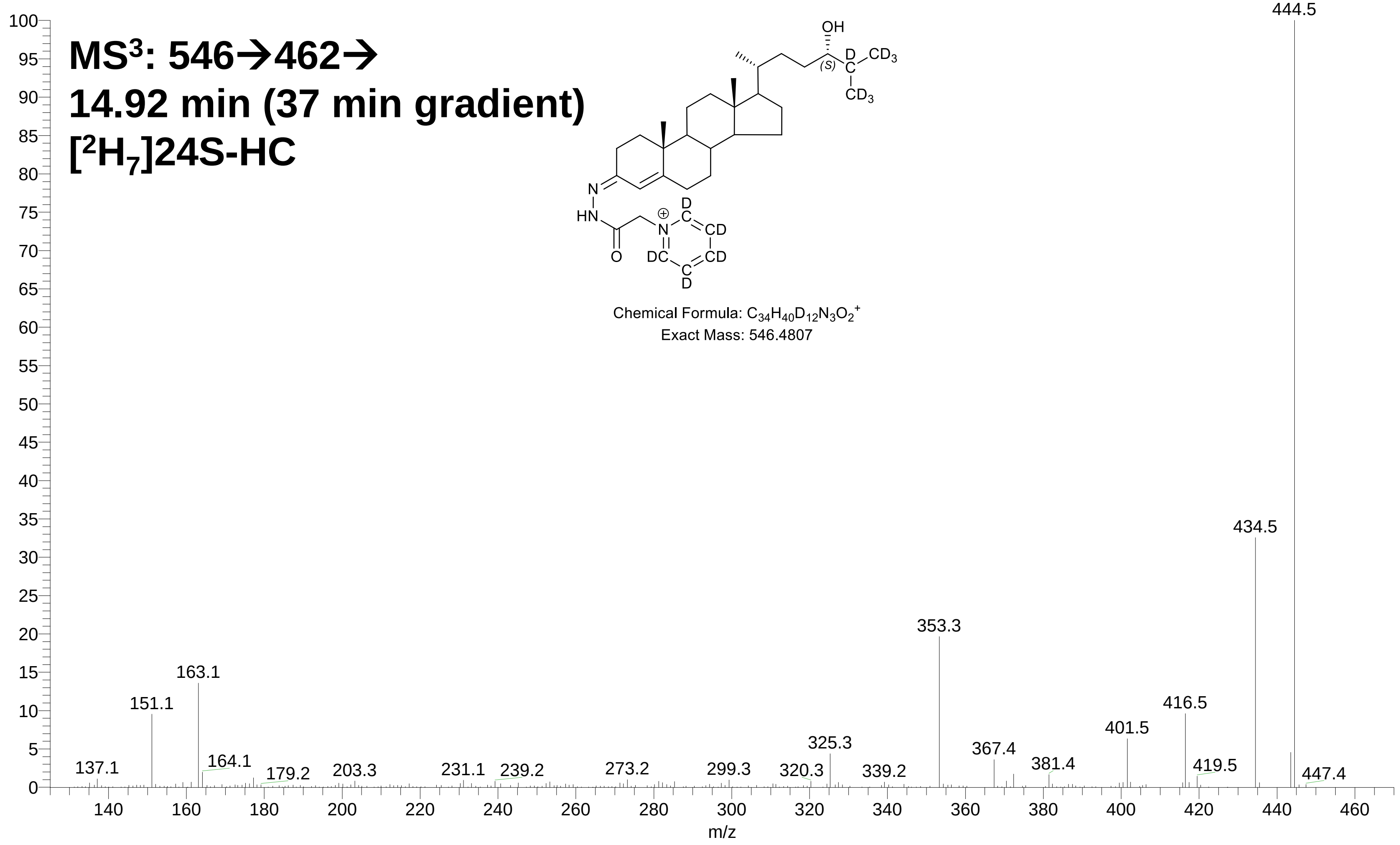

S3D

EY_191014_100uL-NIST-QC_100uL-OxySpla... 10/15/19 02:44:12
100uL NIST (2018) + 100ul OxysterolSPLASH + 10ng 22S-HCO-D7 + 20ug
EY_191014_100uL-NIST-QC_100uL-OxySplash_rep2of5_Fr1A=GPd5_Fr1E
F: ITMS + c ESI Full ms3 546.48@cid30.00 462.41@cid35.00 [125.00-555.0

18.71 AV: 1 NL: 4.78E3

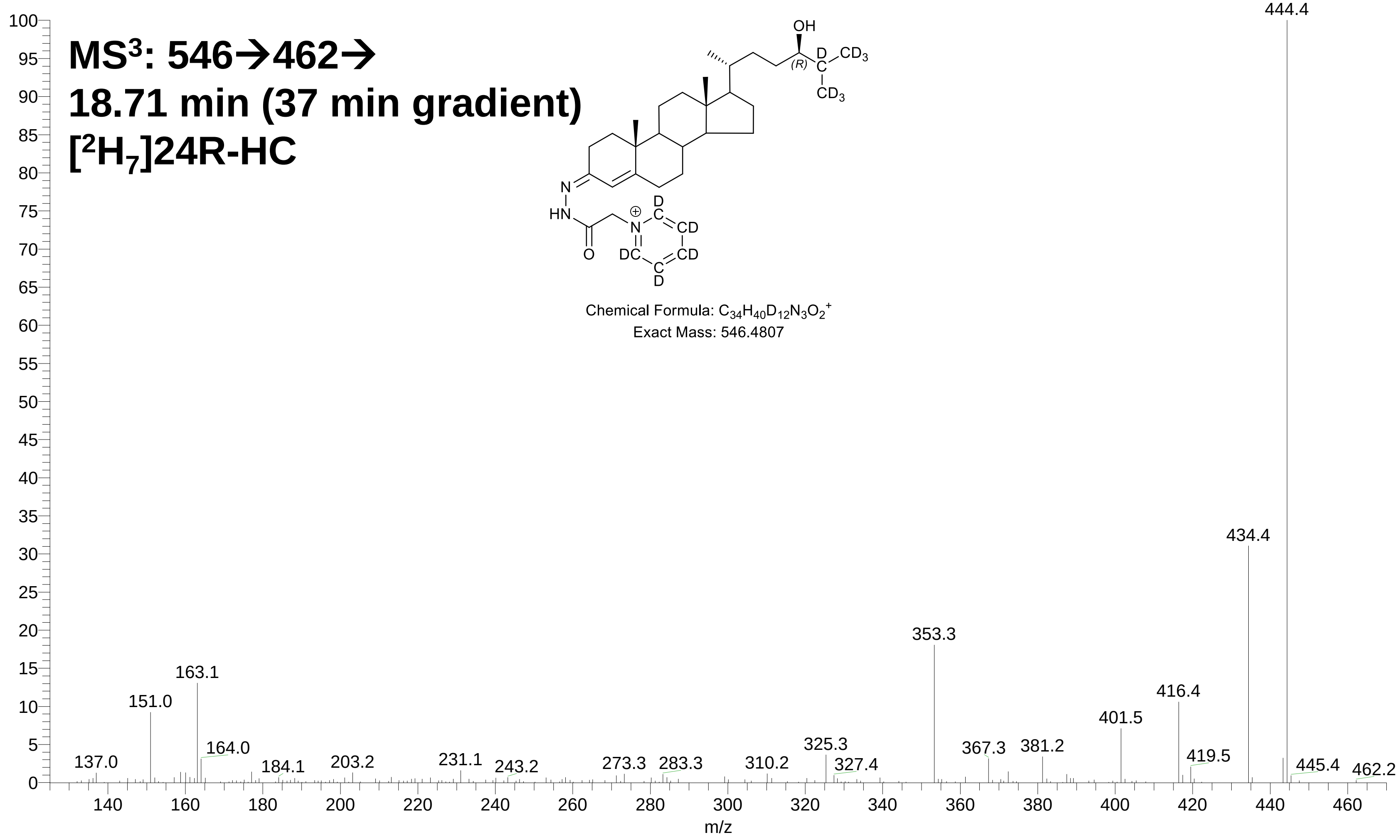

S3E

EY_191014_100uL-NIST-QC_100uL-OxySpla... 10/15/19 02:44:12
100uL NIST (2018) + 100ul OxysterolSPLASH + 10ng 22S-HCO-D7 + 20ug
EY_191014_100uL-NIST-QC_100uL-OxySplash_rep2of5_Fr1A=GPd5_Fr1E
F: ITMS + c ESI Full ms3 539.44@cid30.00 455.36@cid35.00 [125.00-550.0

15.94 AV: 1 NL: 2.08E3

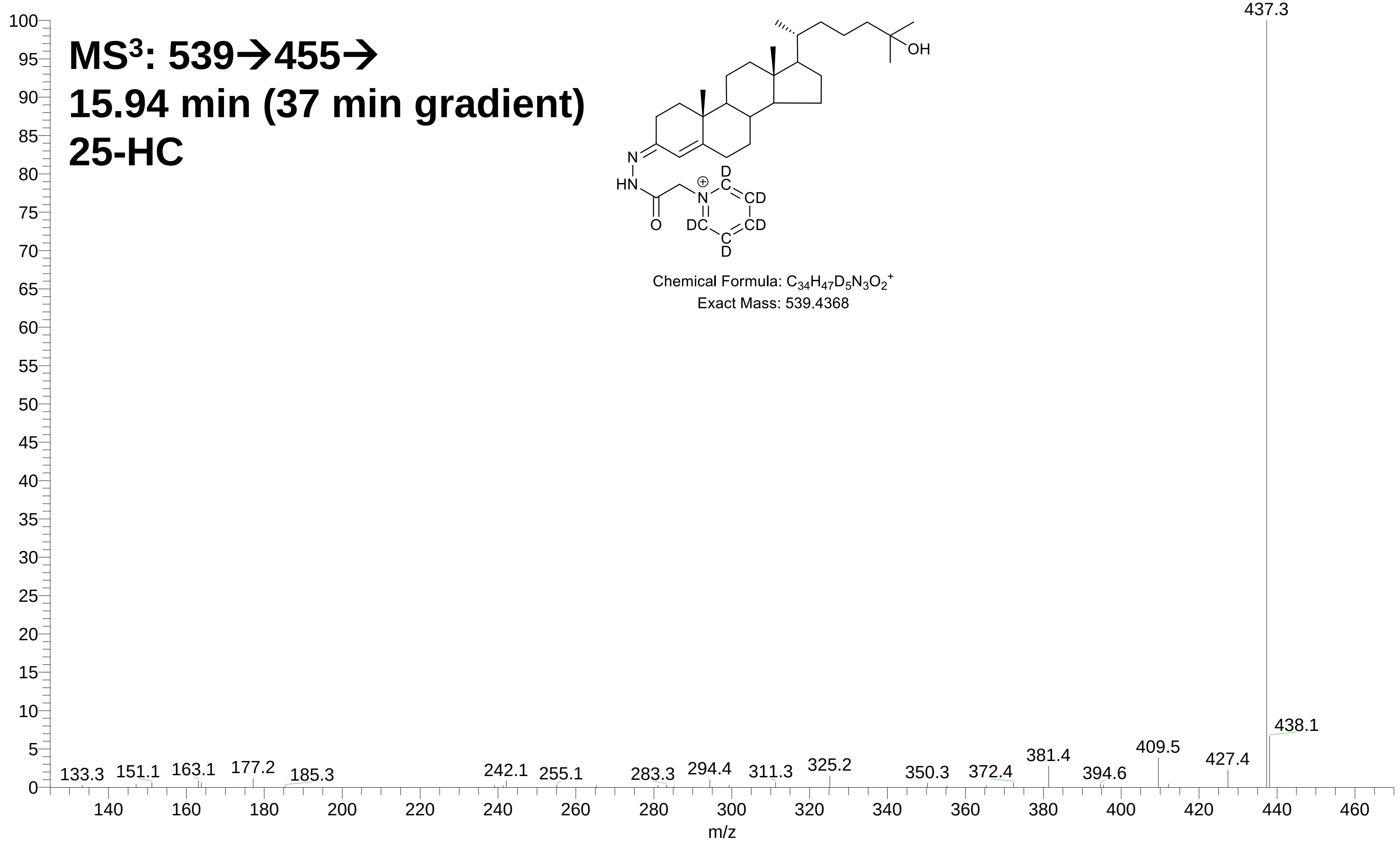

S3F

EY_191014_100uL-NIST-QC_100uL-OxySpla... 10/15/19 02:44:12
100uL NIST (2018) + 100ul OxysterolSPLASH + 10ng 22S-HCO-D7 + 20ug

EY_191014_100uL-NIST-QC_100uL-OxySplash_rep2of5_Fr1A=GPd5_Fr1E
F: ITMS + c ESI Full ms3 545.47@cid30.00 461.40@cid35.00 [125.00-555.0

15.79 AV: 1 NL: 1.12E4

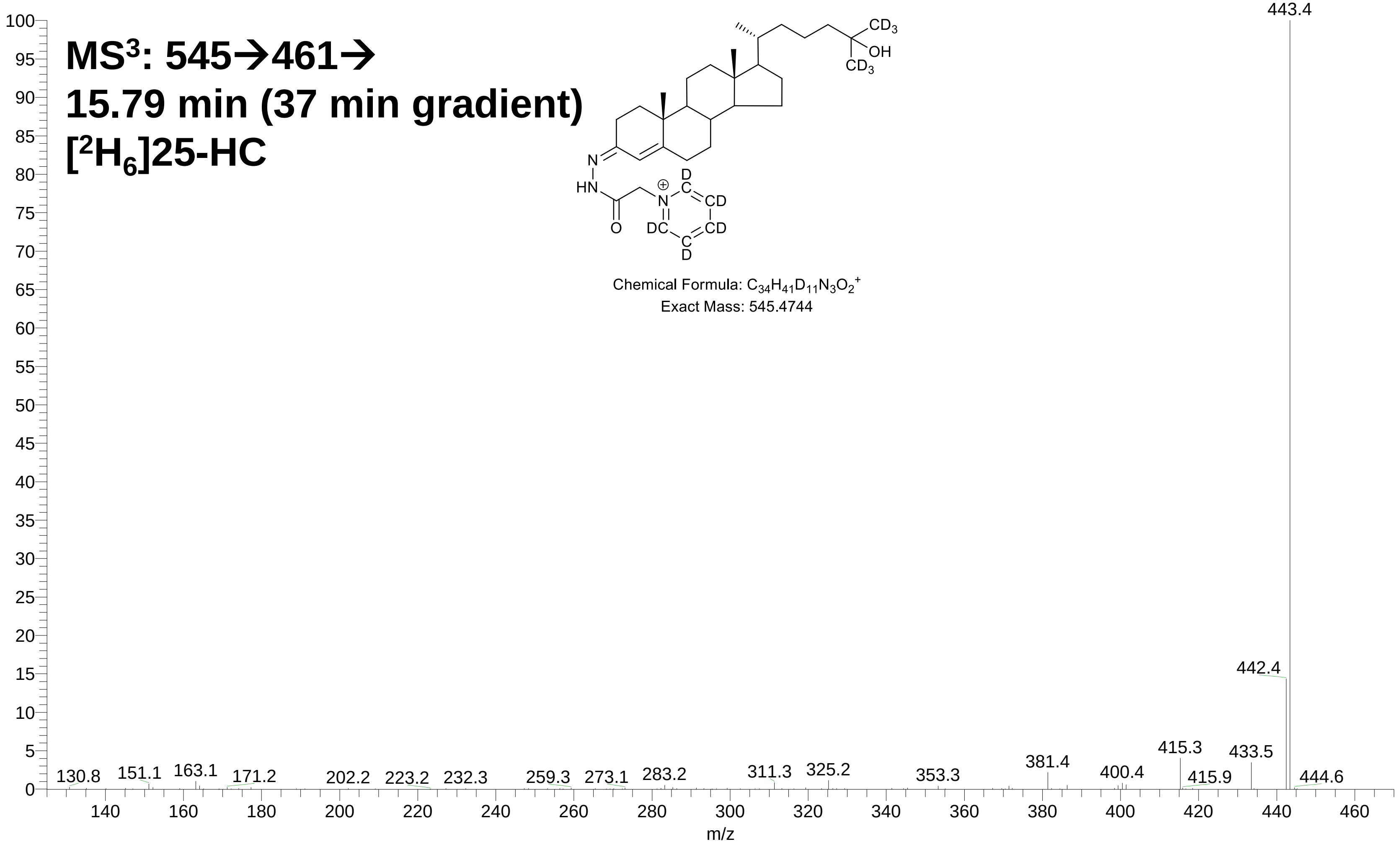

S3G

EY_191014_100uL-NIST-QC_100uL-OxySpla... 10/15/19 02:44:12
100uL NIST (2018) + 100ul OxysterolSPLASH + 10ng 22S-HCO-D7 + 20ug

EY_191014_100uL-NIST-QC_100uL-OxySplash_rep2of5_Fr1A=GPd5_Fr1E
F: ITMS + c ESI Full ms3 539.44@cid30.00 455.36@cid35.00 [125.00-550.0

17.75 AV: 1 NL: 4.63E3

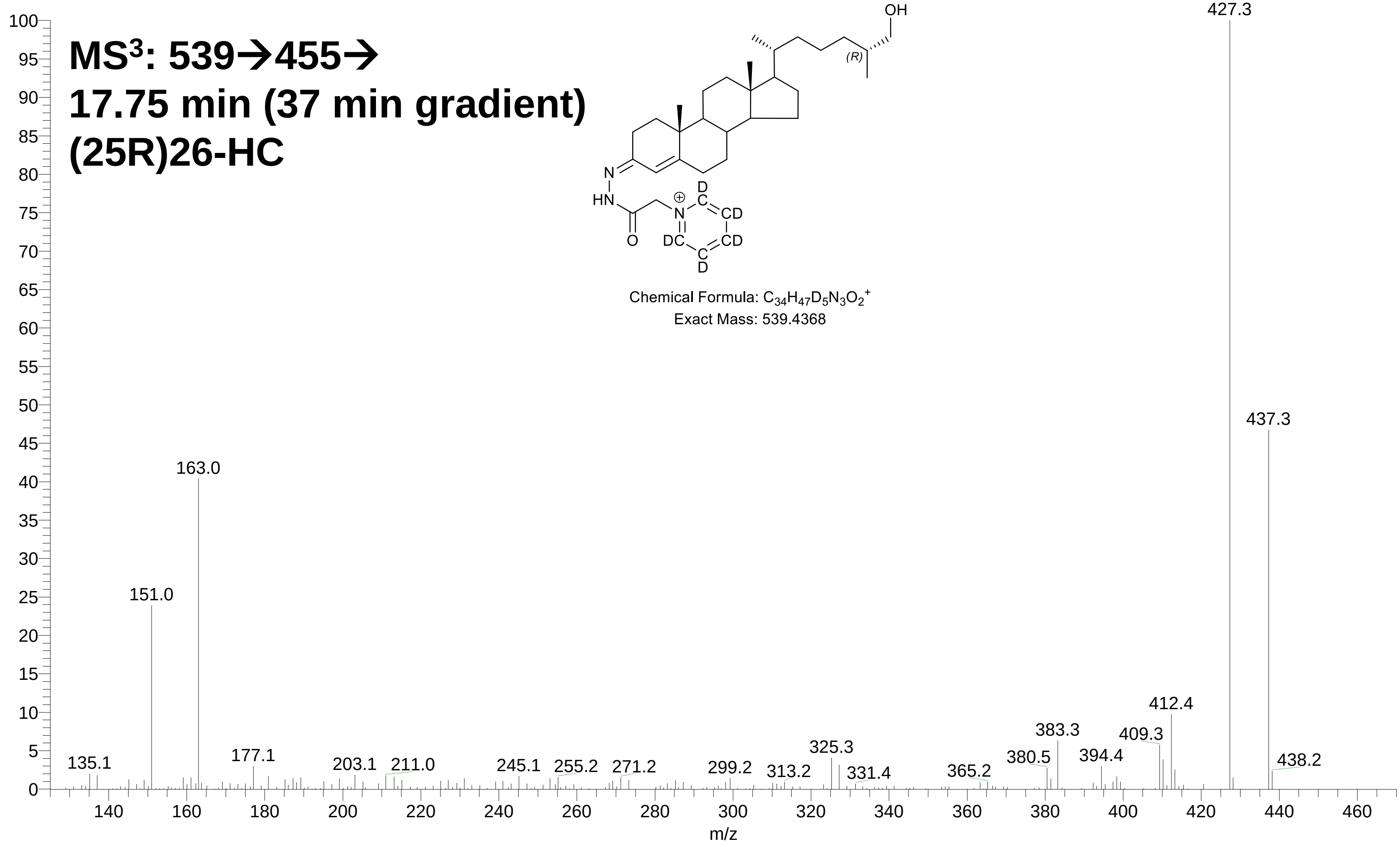

S3H

EY_191014_100uL-NIST-QC_100uL-OxySpla... 10/15/19 02:44:12
100uL NIST (2018) + 100ul OxysterolSPLASH + 10ng 22S-HCO-D7 + 20ug
EY_191014_100uL-NIST-QC_100uL-OxySplash_rep2of5_Fr1A=GPd5_Fr1E
F: ITMS + c ESI Full ms3 545.47@cid30.00 461.40@cid35.00 [125.00-555.0

17.58 AV: 1 NL: 2.24E4

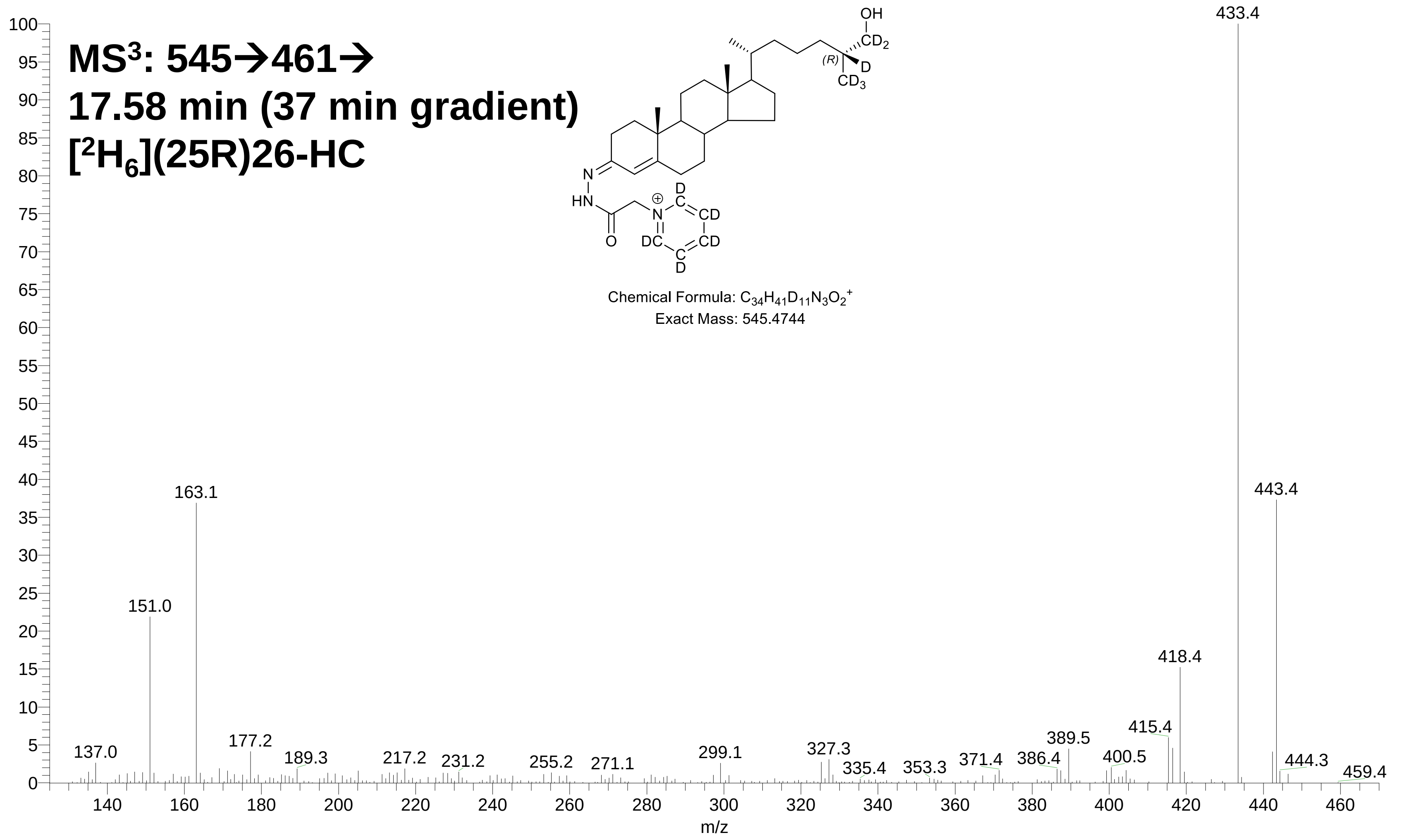

S3I

EY_191014_100uL-NIST-QC_100uL-OxySpla... 10/15/19 02:44:12
100uL NIST (2018) + 100ul OxysterolSPLASH + 10ng 22S-HCO-D7 + 20ug

EY_191014_100uL-NIST-QC_100uL-OxySplash_rep2of5_Fr1A=GPd5_Fr1E 7.86 AV: 1 NL: 1.28E3
F: ITMS + c ESI Full ms3 546.48@cid30.00 462.41@cid35.00 [125.00-555.(

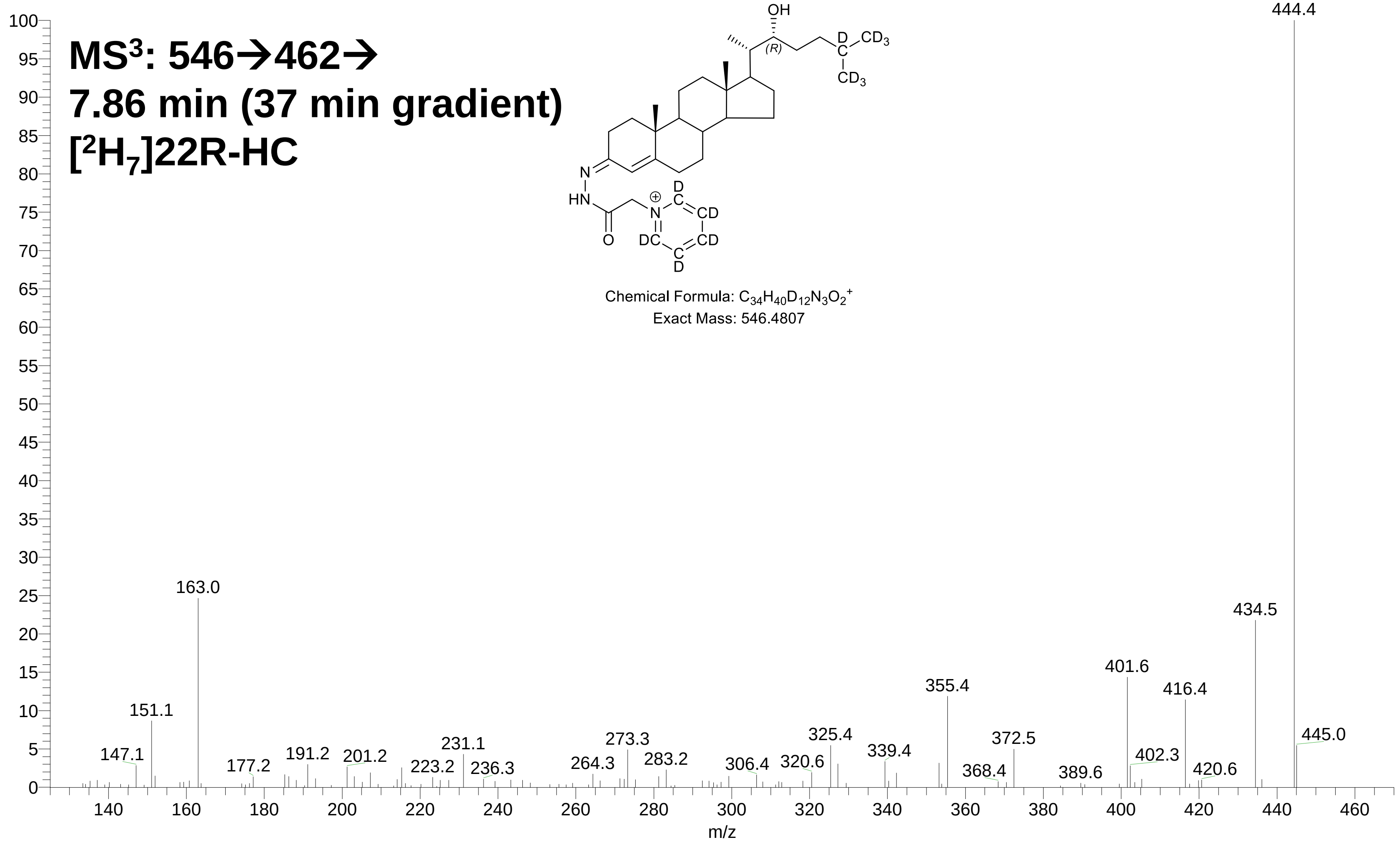

S3J

EY_191014_100uL-NIST-QC_100uL-OxySpla... 10/15/19 02:23:26
100uL NIST (2018) + 100ul OxysterolSPLASH + 10ng 22S-HCO-D7 + 20ug
EY_191014_100uL-NIST-QC_100uL-OxySplash_rep2of5_Fr1A=GPd5_Fr1E 8.35 AV: 1 NL: 4.92E1
F: ITMS + c ESI Full ms3 539.44@cid30.00 455.36@cid35.00 [125.00-550.0

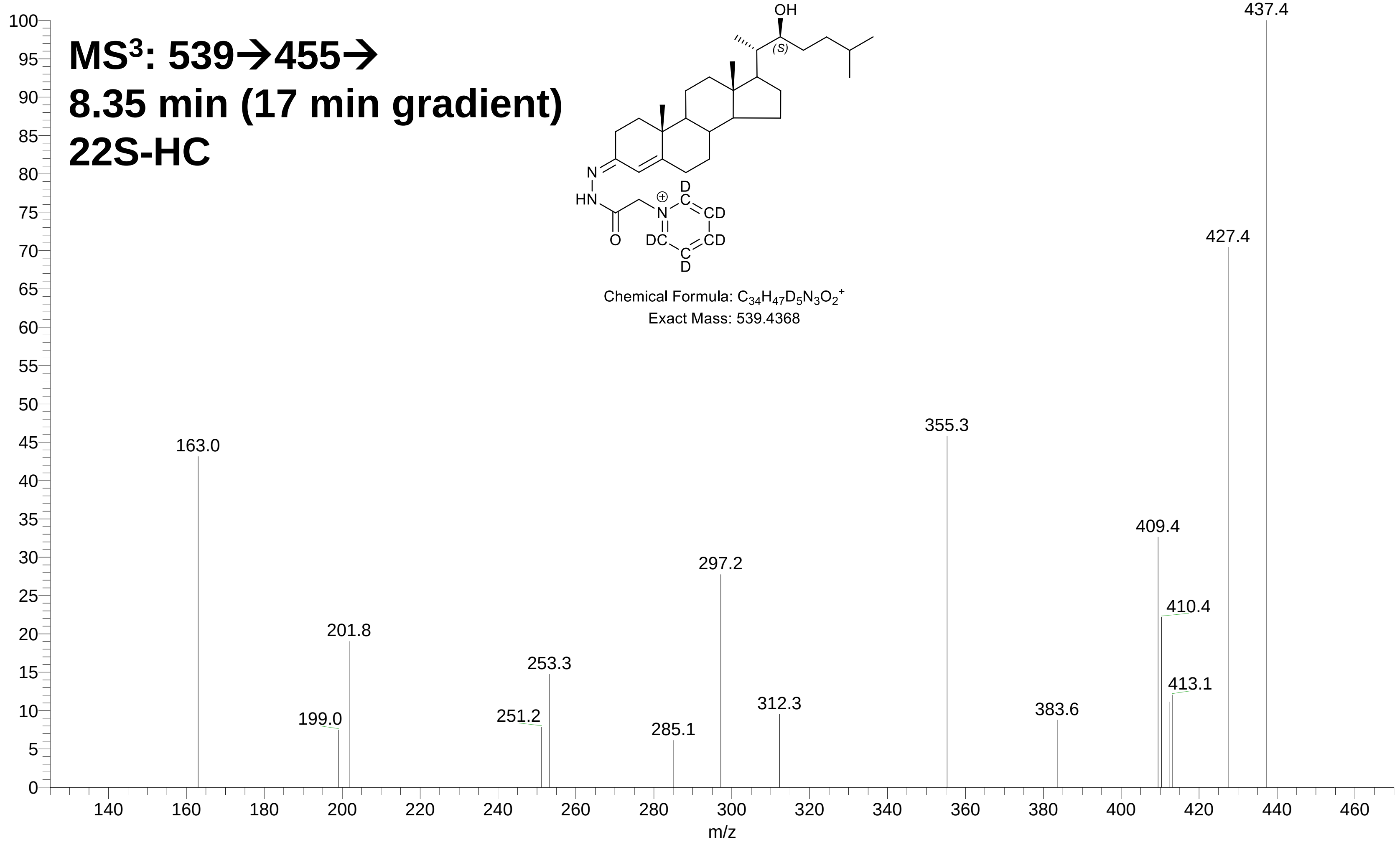

S3K

EY_191014_100uL-NIST-QC_100uL-OxySpla... 10/15/19 02:23:26
100uL NIST (2018) + 100ul OxysterolSPLASH + 10ng 22S-HCO-D7 + 20ug

EY_191014_100uL-NIST-QC_100uL-OxySplash_rep2of5_Fr1A=GPd5_Fr1E 8.34 AV: 1 NL: 1.04E5
F: ITMS + c ESI Full ms3 546.48@cid30.00 462.41@cid35.00 [125.00-555.0

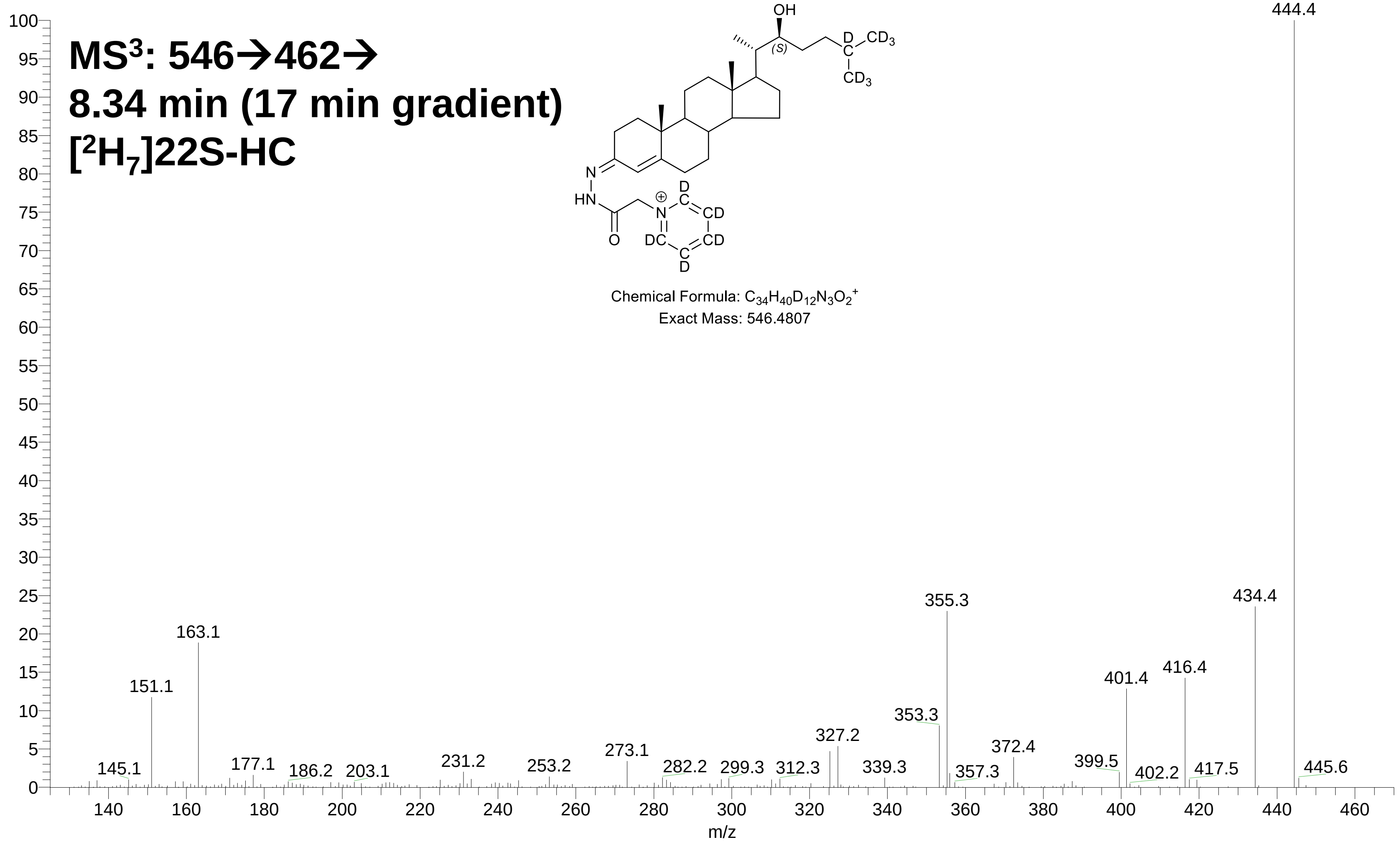

EY_191014_100uL-NIST-QC_100uL-OxySpla... 10/15/19 02:44:12
100uL NIST (2018) + 100ul OxysterolSPLASH + 10ng 22S-HCO-D7 + 20ug
EY_191014_100uL-NIST-QC_100uL-OxySplash_rep2of5_Fr1A=GPd5_Fr1E 18.35 AV: 1 NL: 6.81E3
F: ITMS + c ESI Full ms3 545.47@cid30.00 461.40@cid35.00 [125.00-555.0

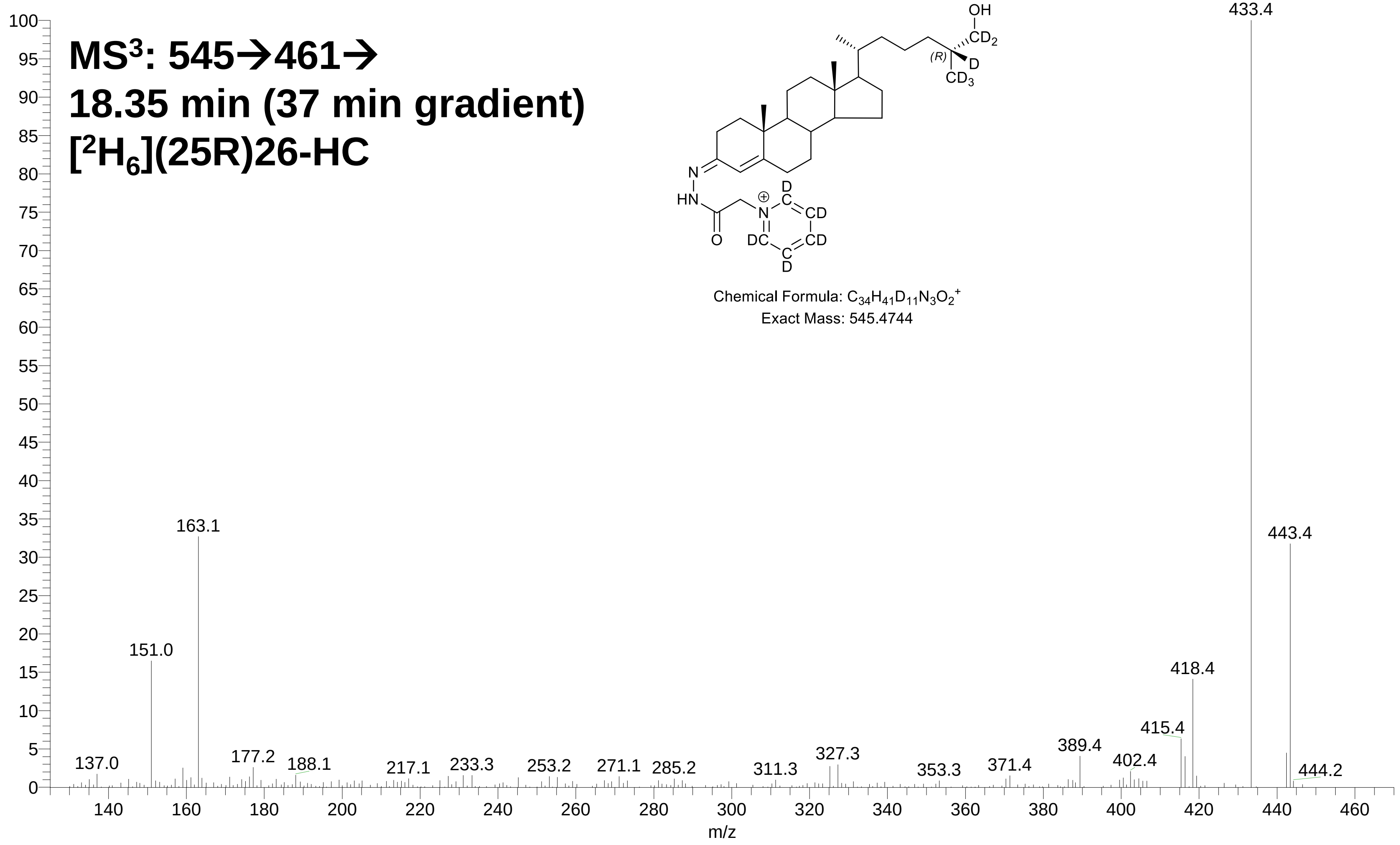

S4A

Figure S4A

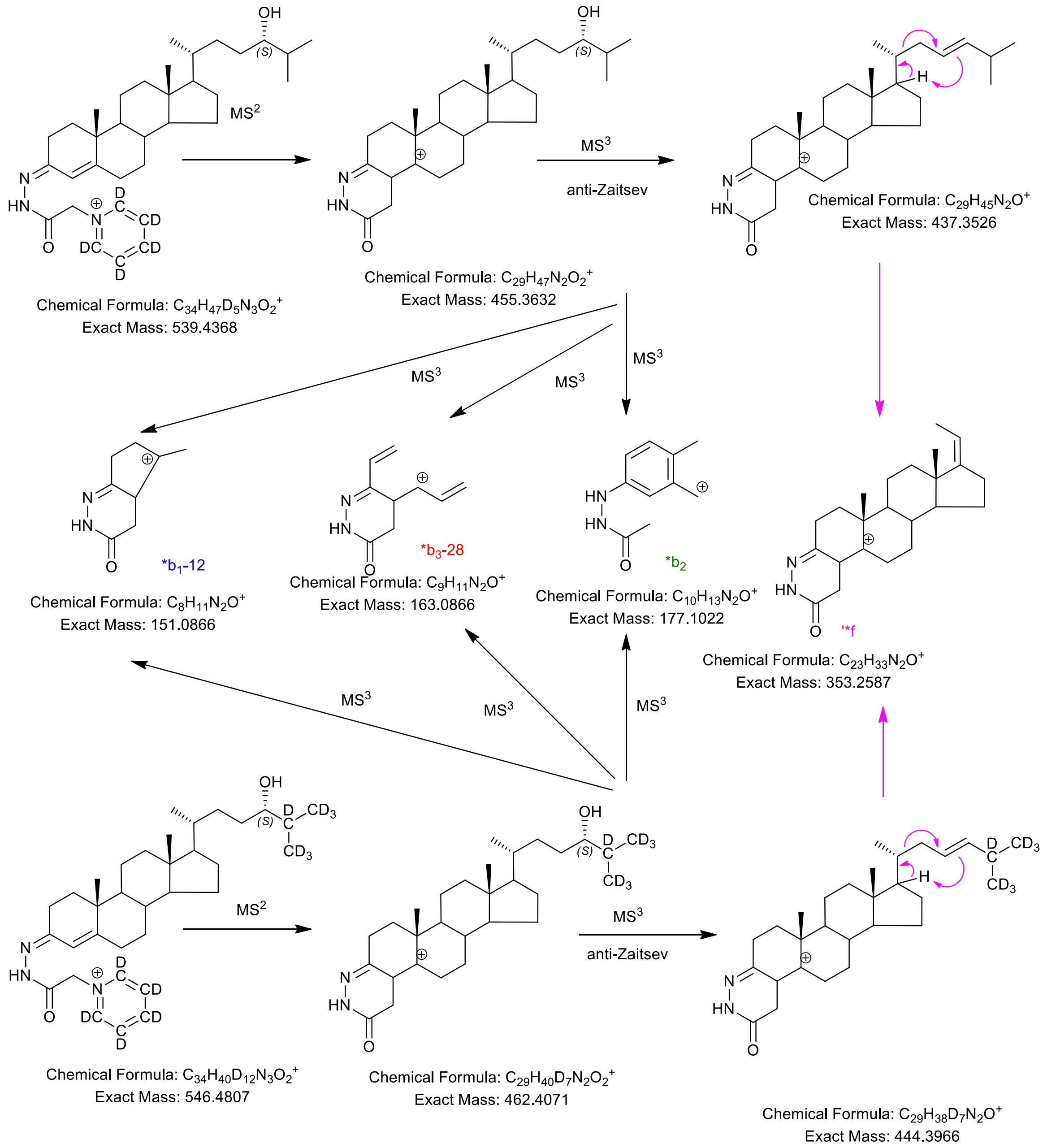

S4B

Figure S4B

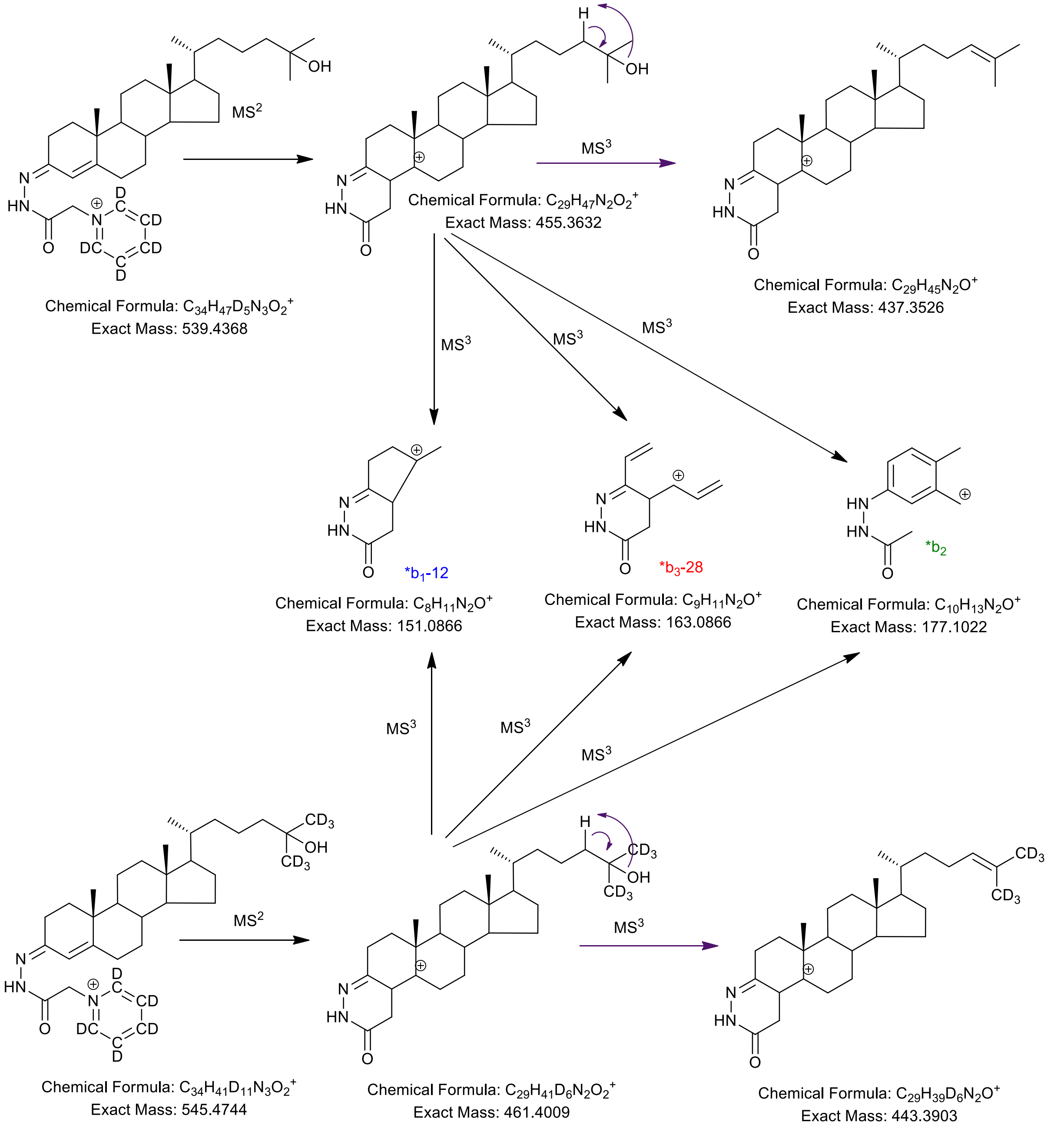

S4C

Figure S4C

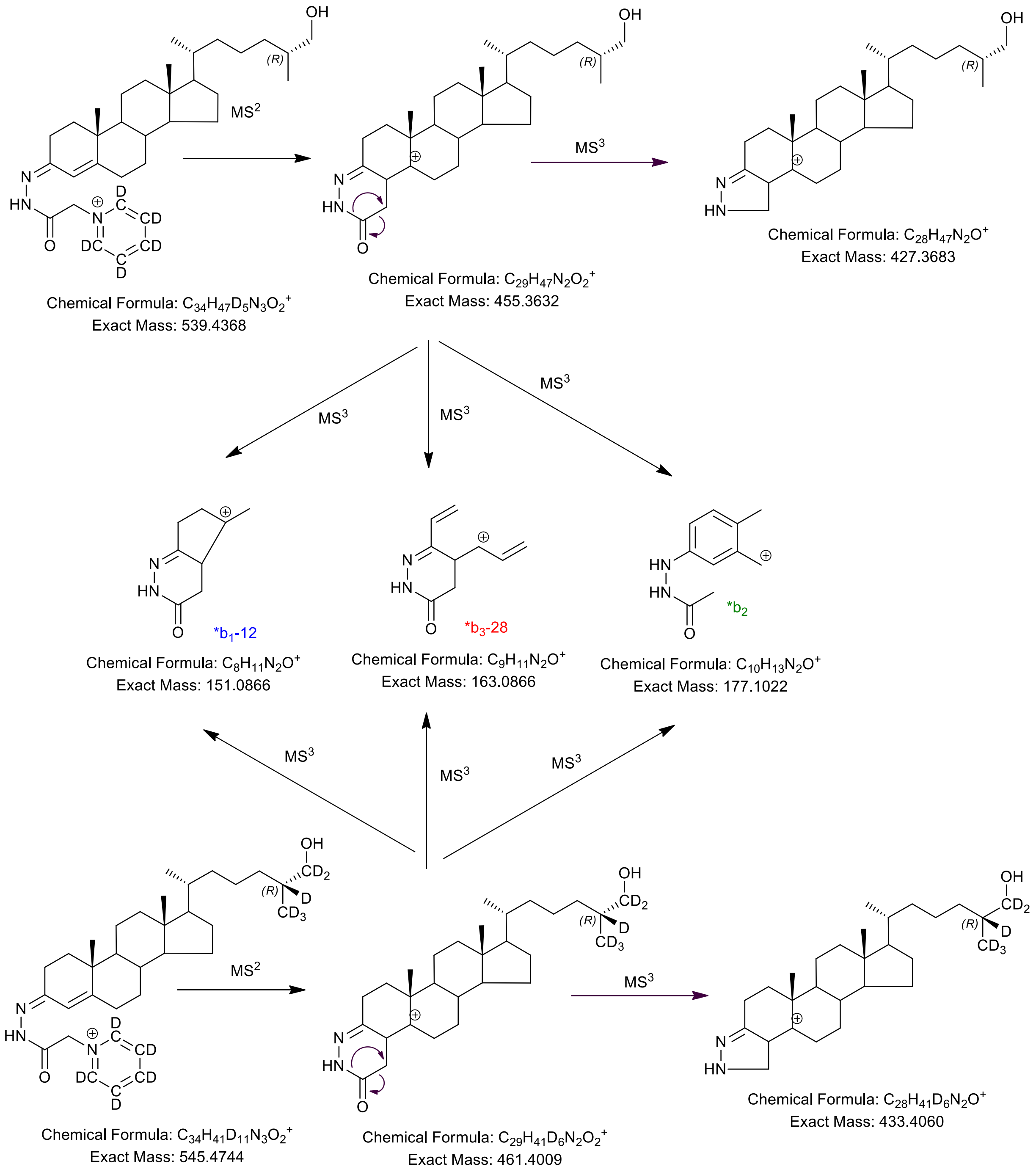

S4D

Figure 4D

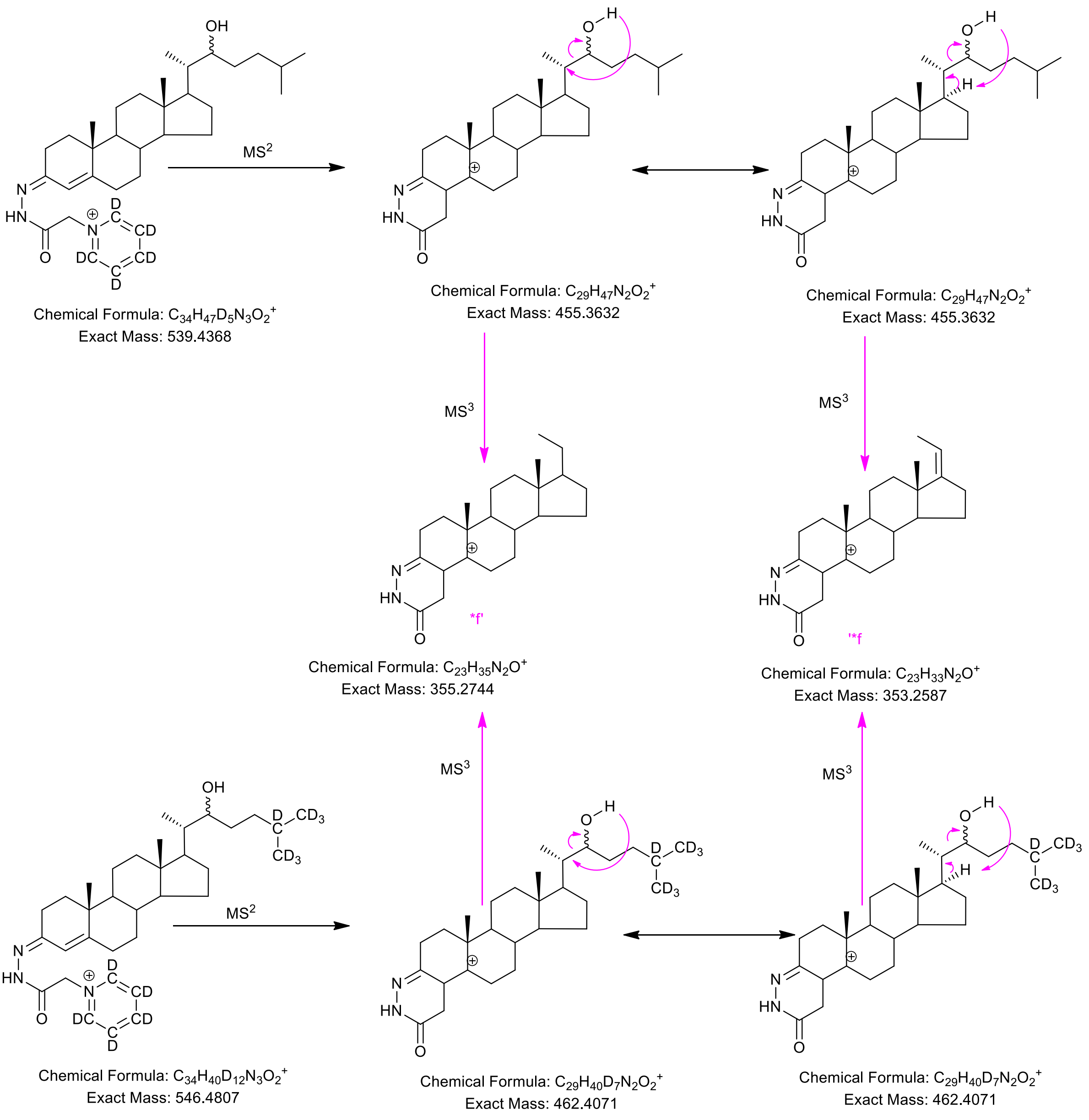

S4E

Figure S4E

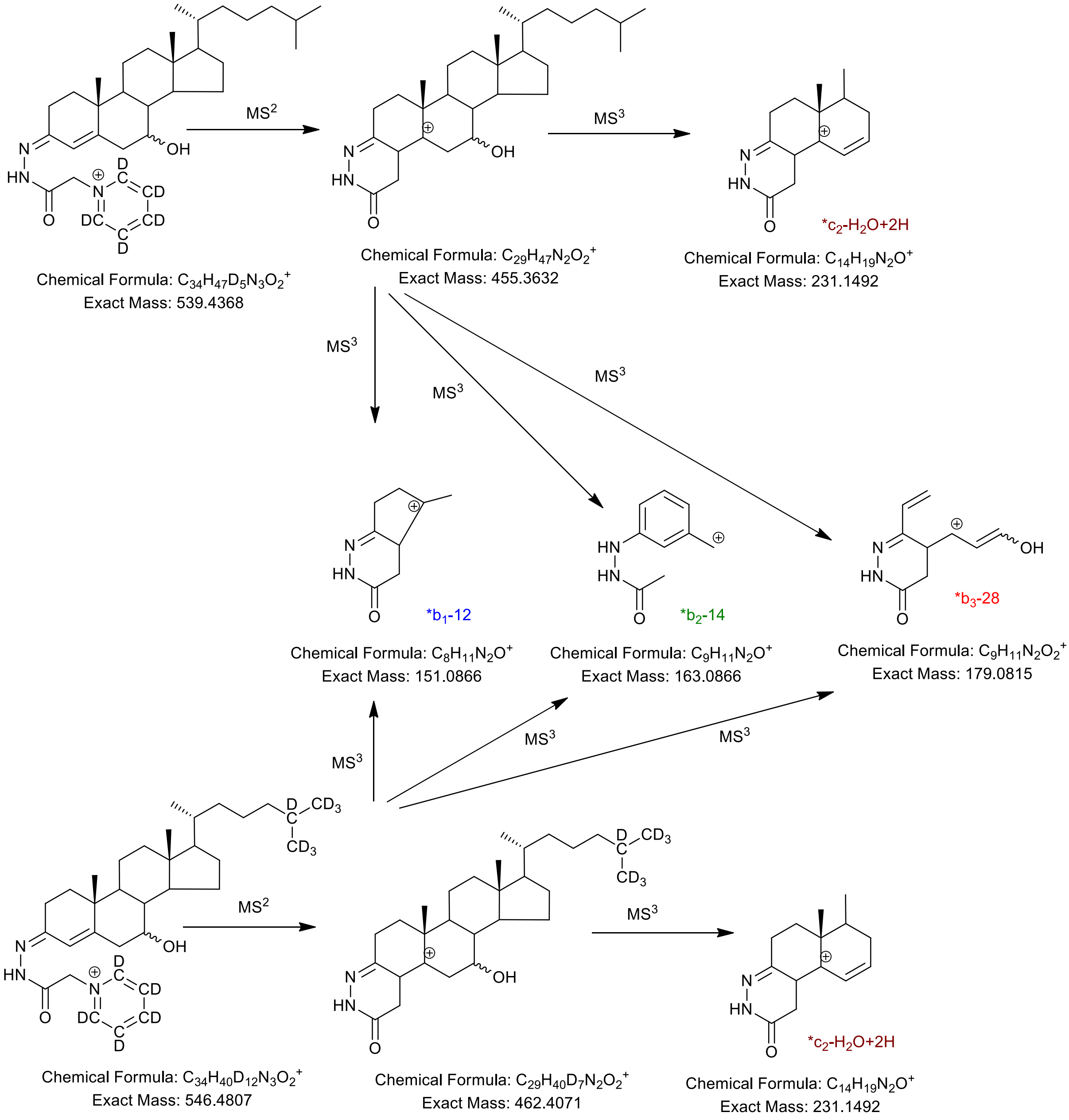

S4F

Figure S4F

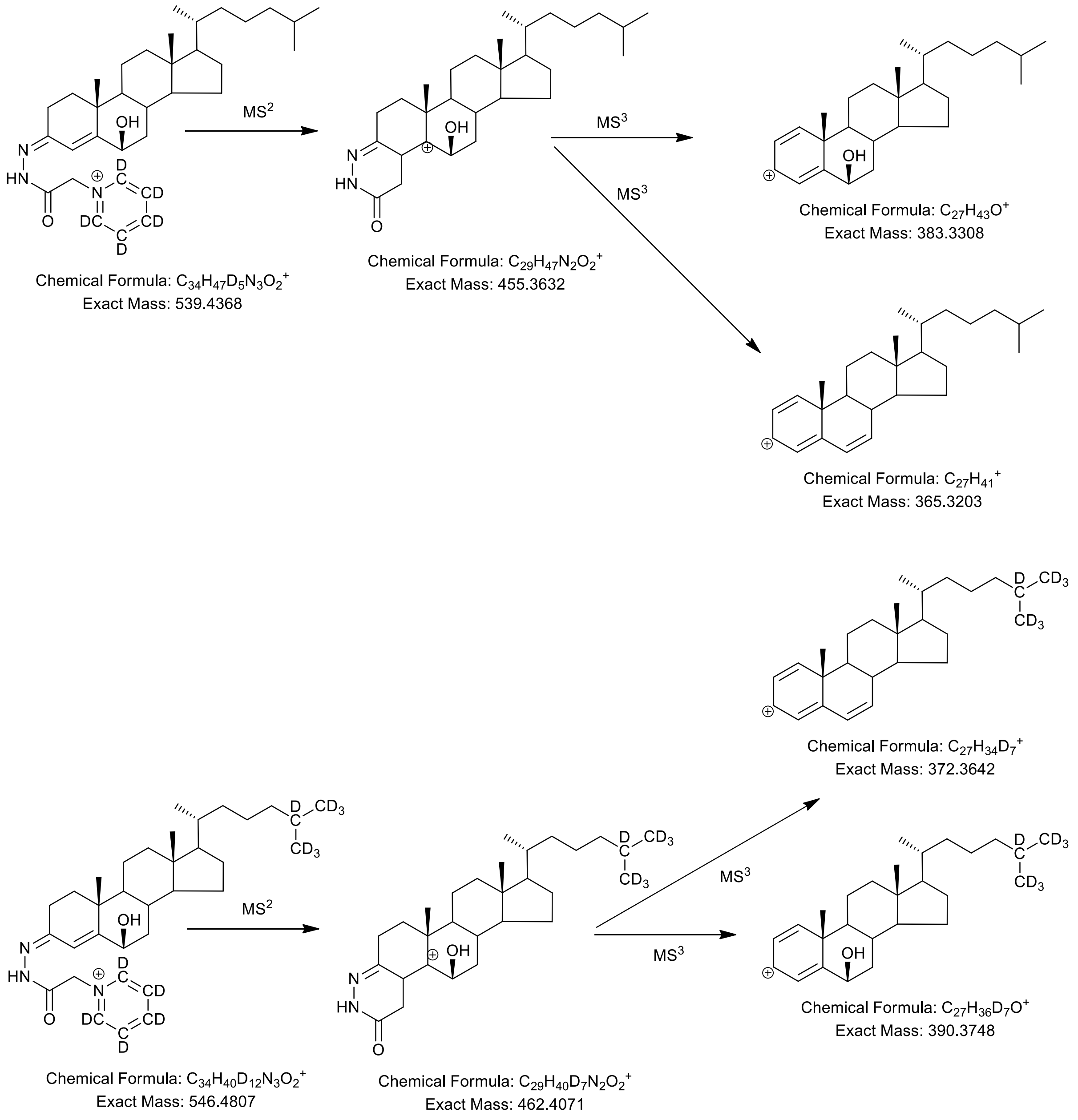

S4G

Figure S4G

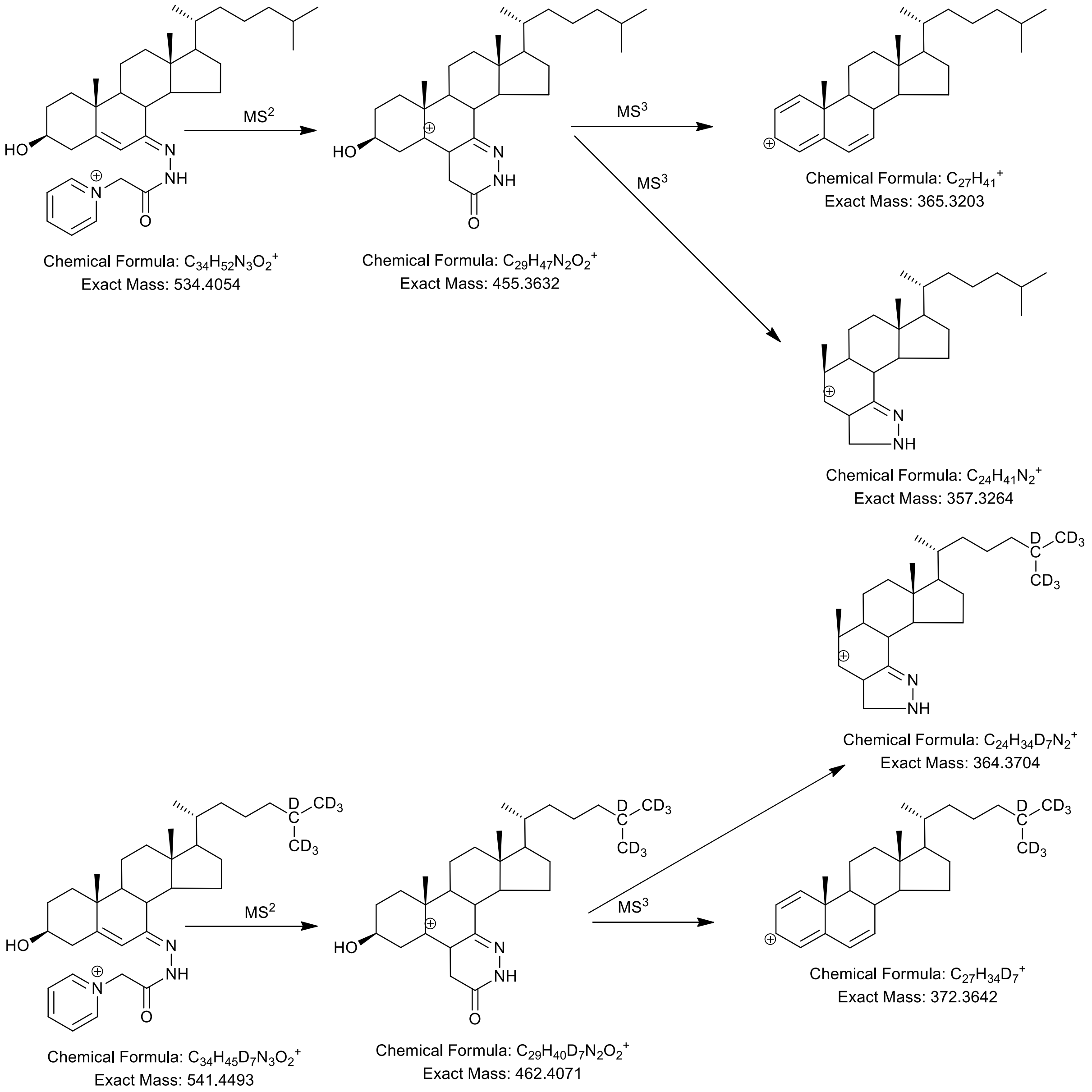

S4H

Figure S4H

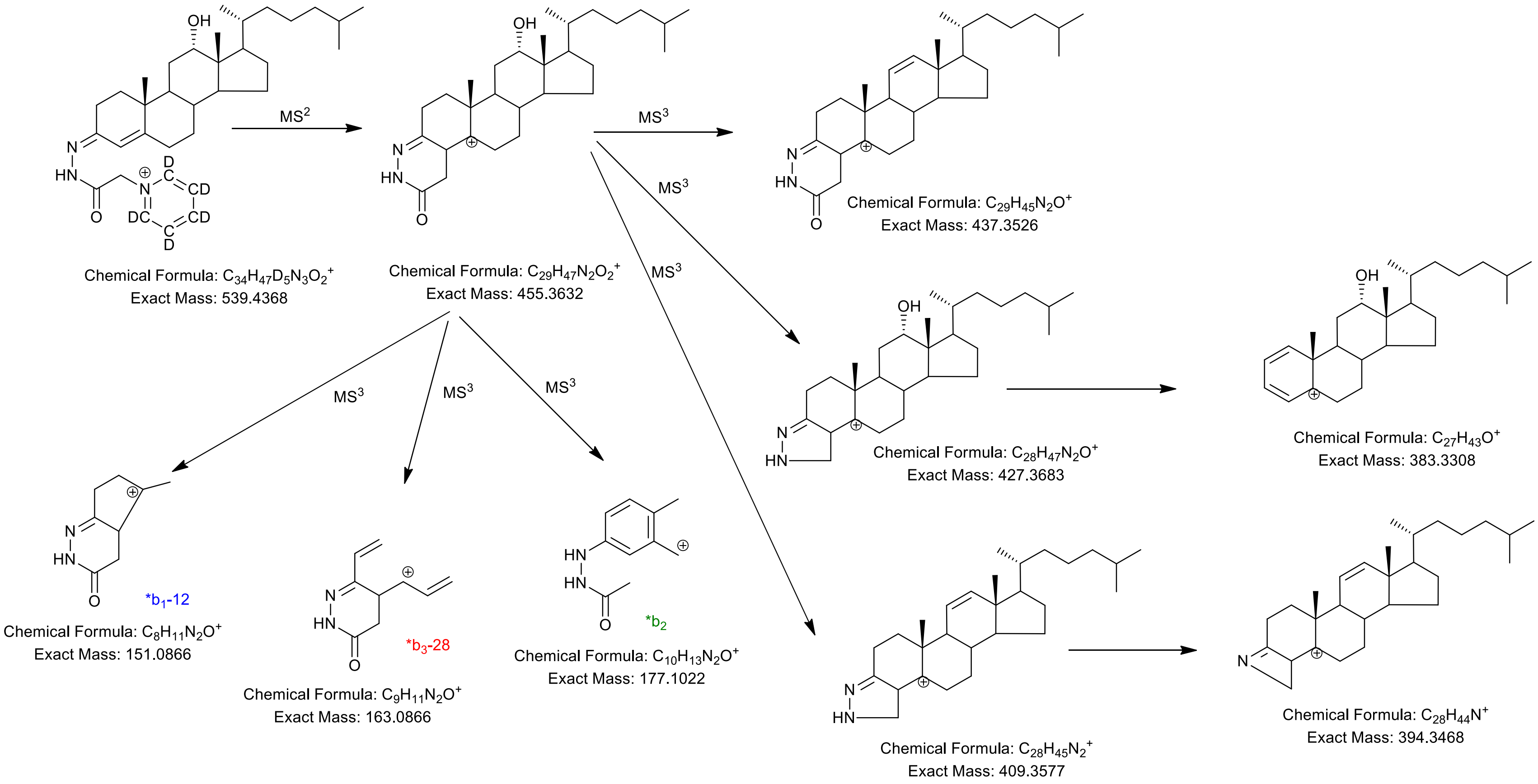

S4I

Figure S4I

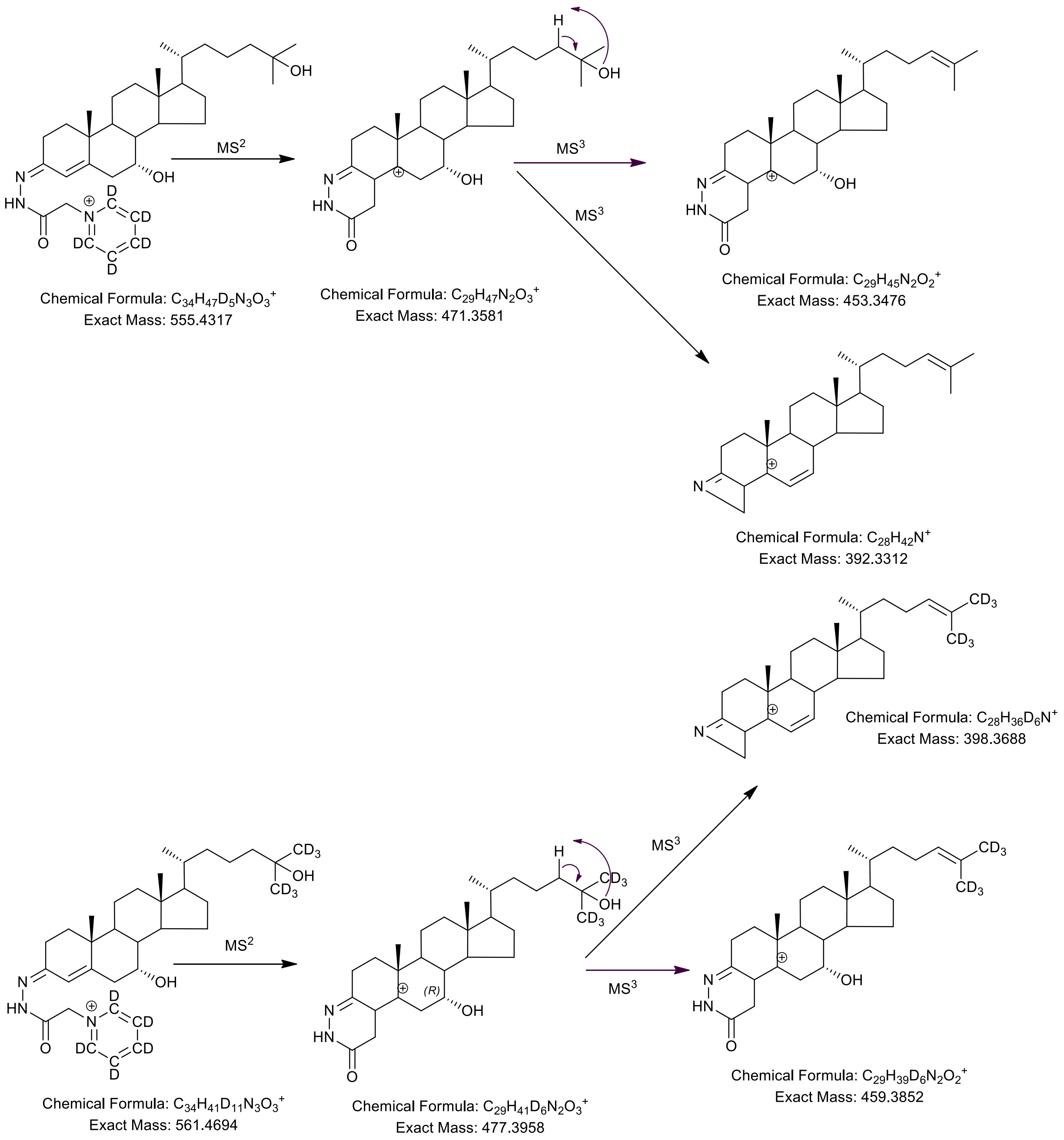

S4J

Figure S4J

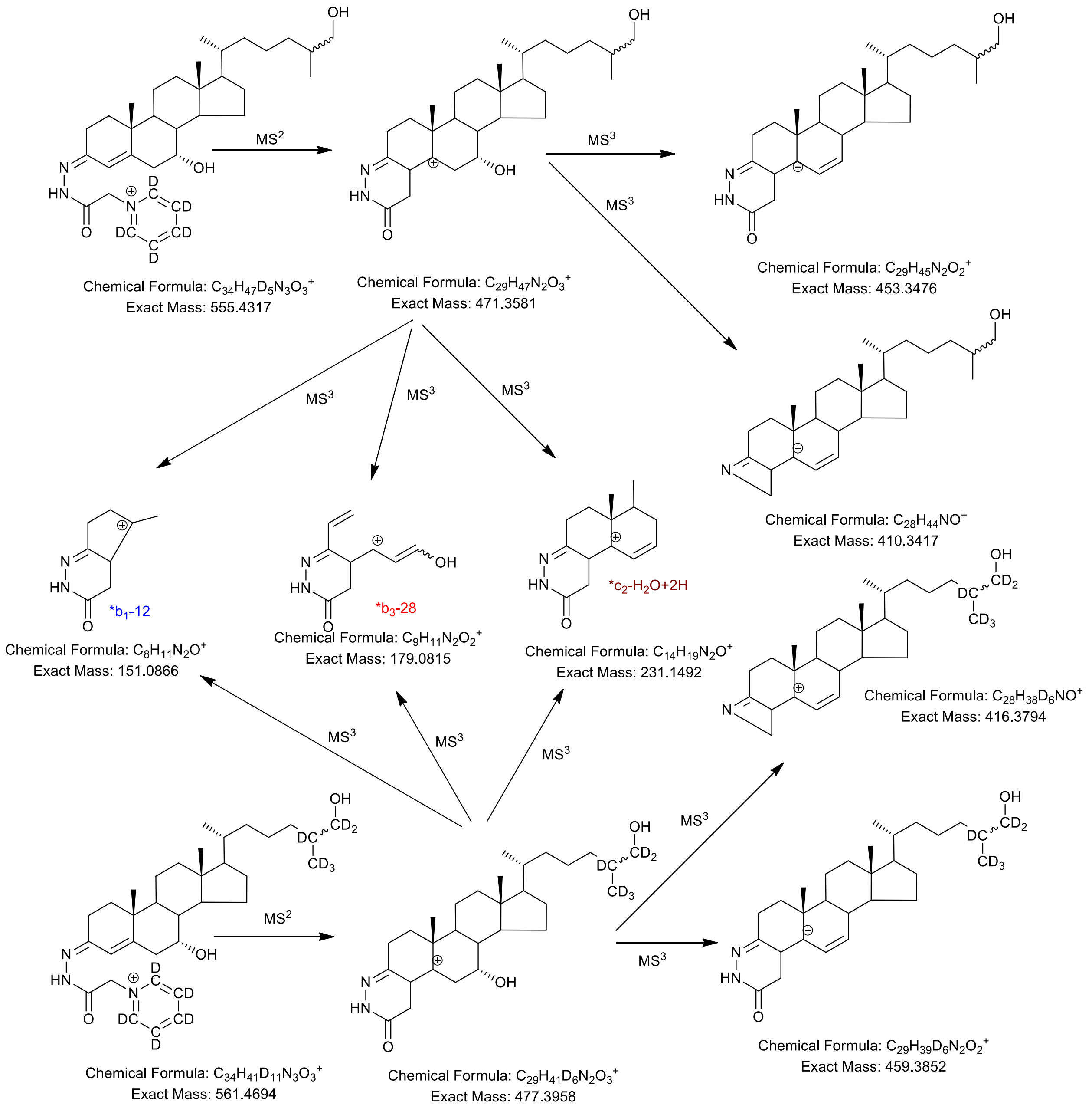

S4K

Figure S4K

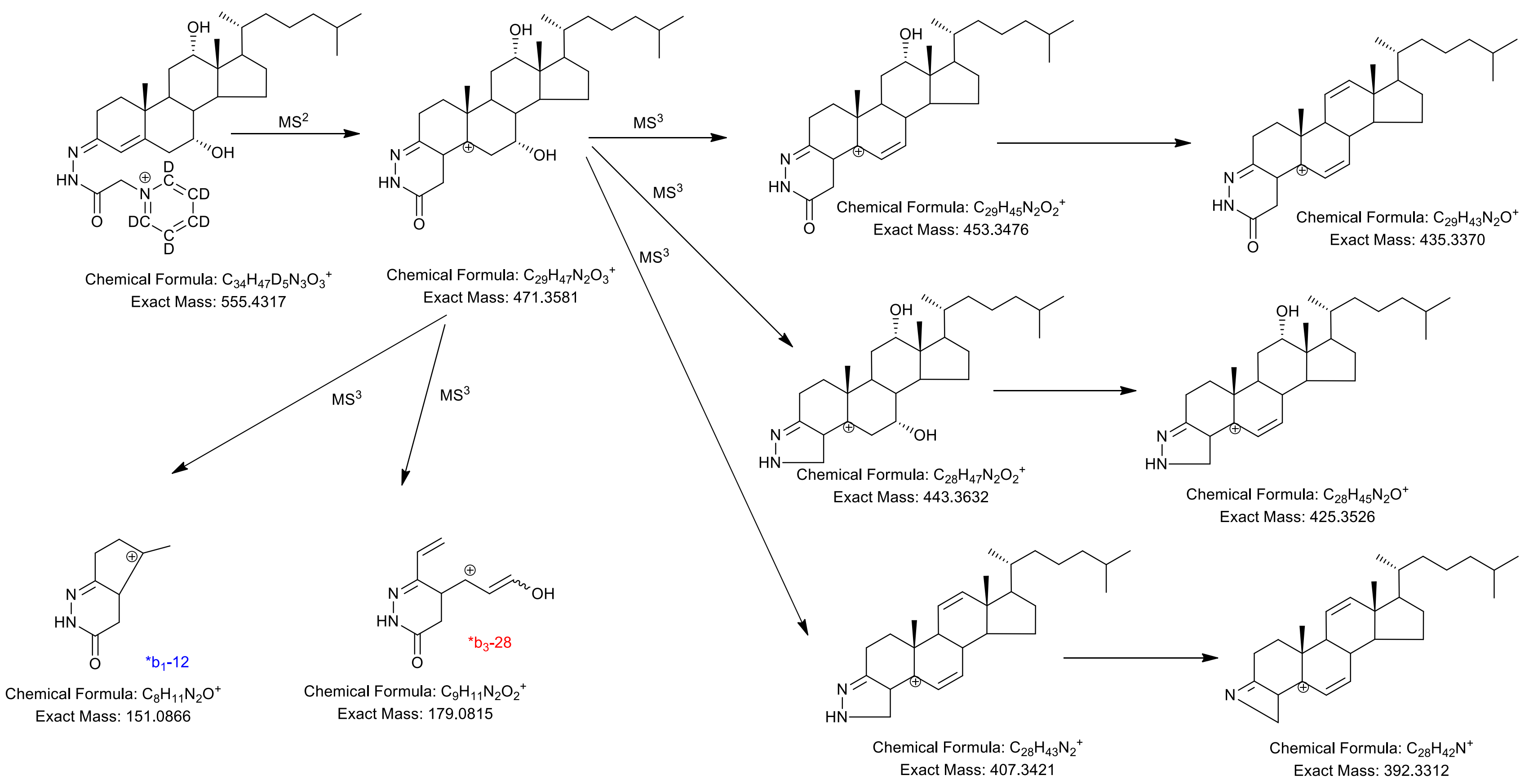

S4L

Figure S4L

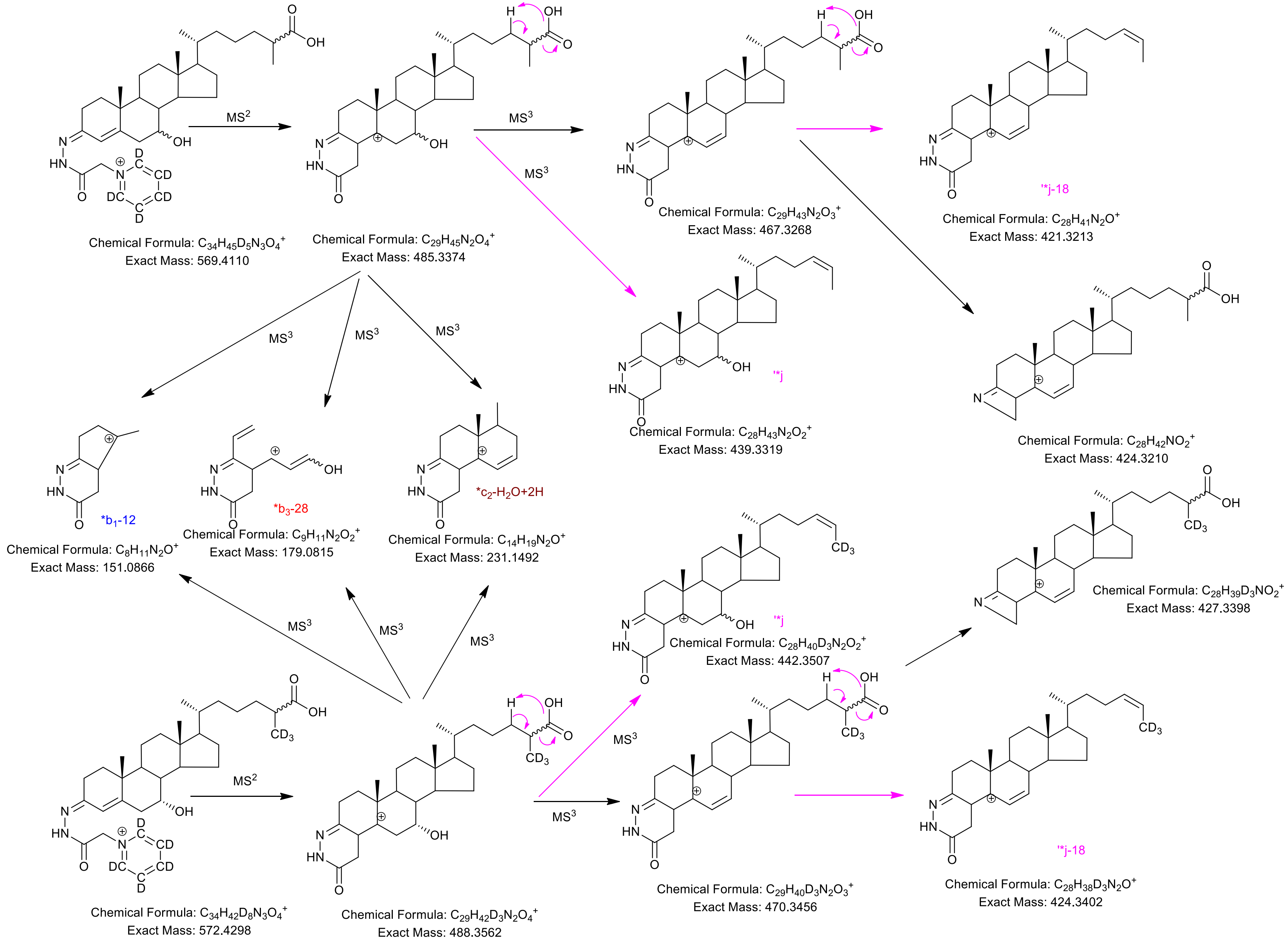

S4M

Figure S4M

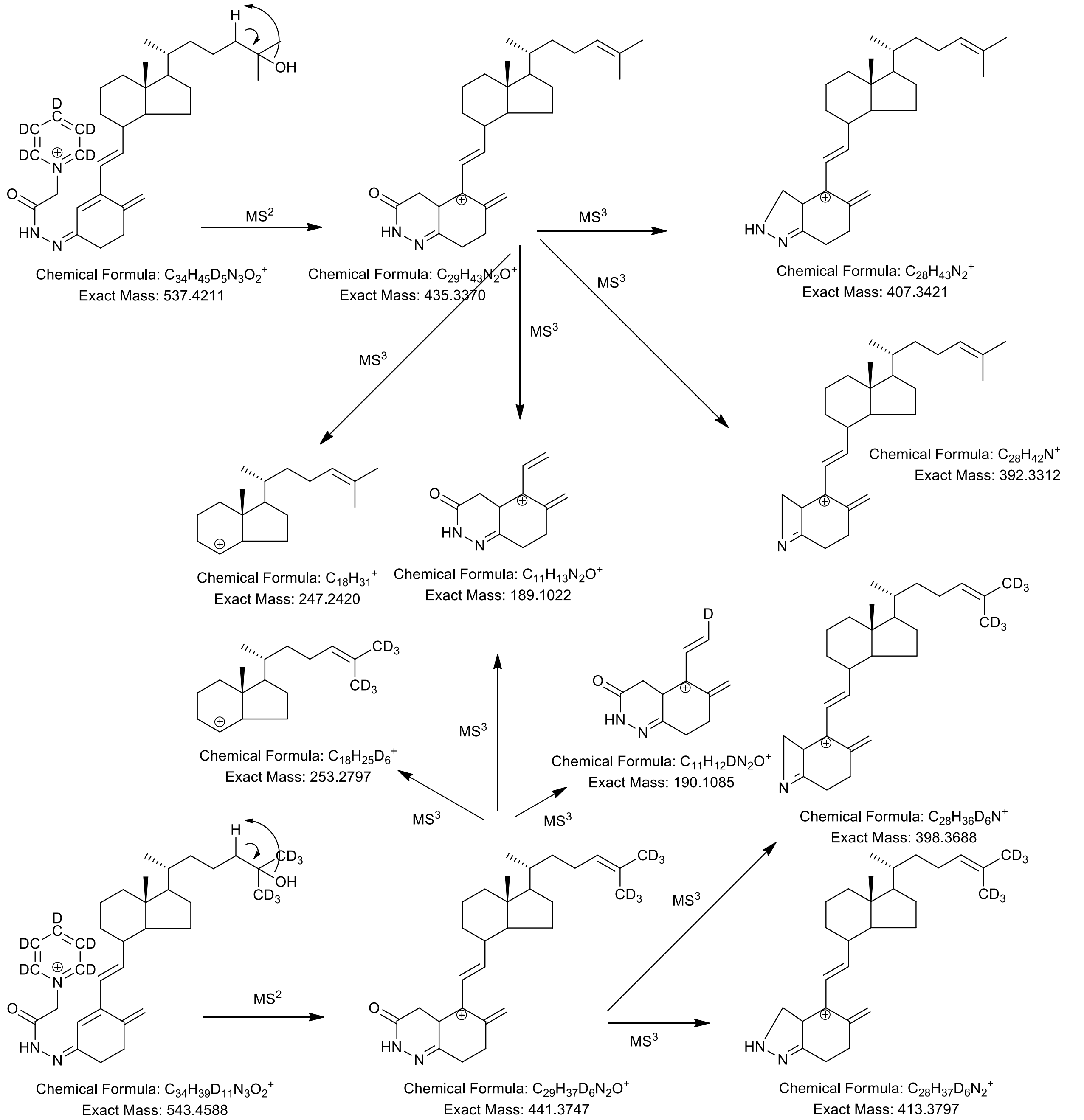

S4N

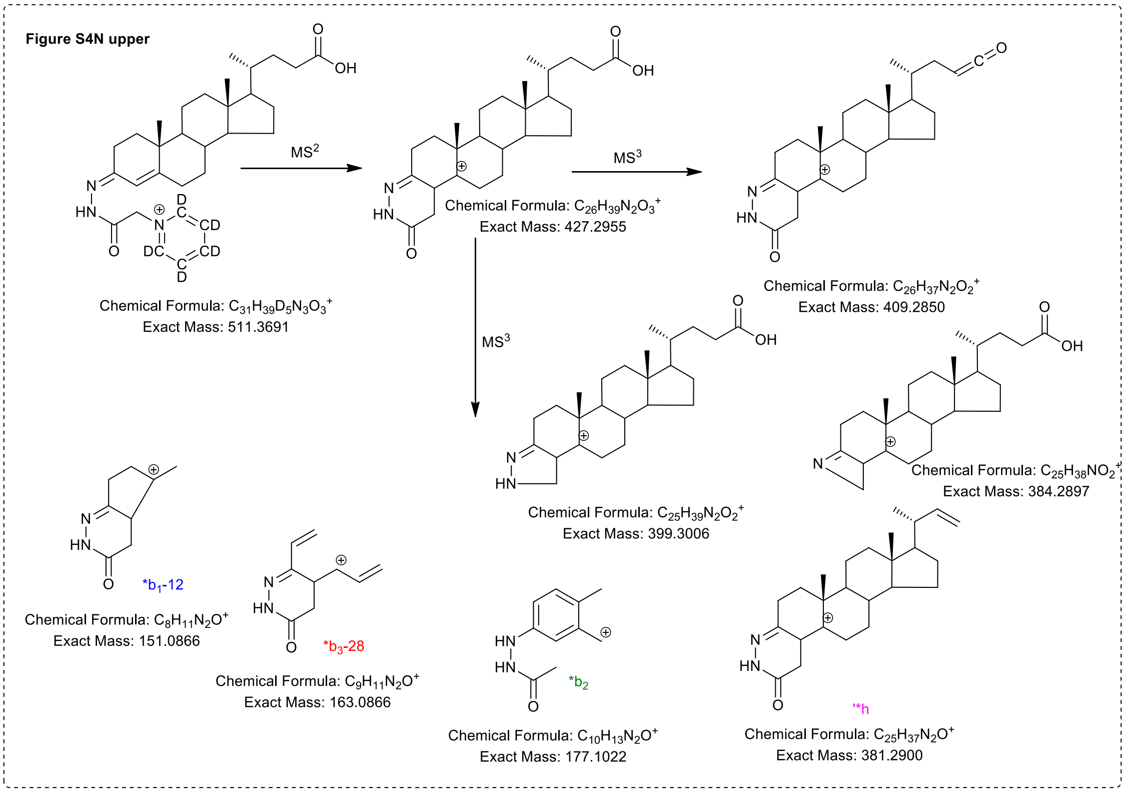
Figure S4N upper
MS2
MS3
MS3
Chemical Formula: C31H39D5N3O3+
Exact Mass: 511.3691
Chemical Formula: C26H39N2O3+
Exact Mass: 427.2955
Chemical Formula: C26H37N2O2+
Exact Mass: 409.2850
Chemical Formula: C25H39N2O2+
Exact Mass: 399.3006
Chemical Formula: C25H38NO2+
Exact Mass: 384.2897
*b1-12
Chemical Formula: C8H11N2O+
Exact Mass: 151.0866
*b3-28
Chemical Formula: C9H11N2O+
Exact Mass: 163.0866
*b2
Chemical Formula: C10H13N2O+
Exact Mass: 177.1022
'*h
Chemical Formula: C25H37N2O+
Exact Mass: 381.2900

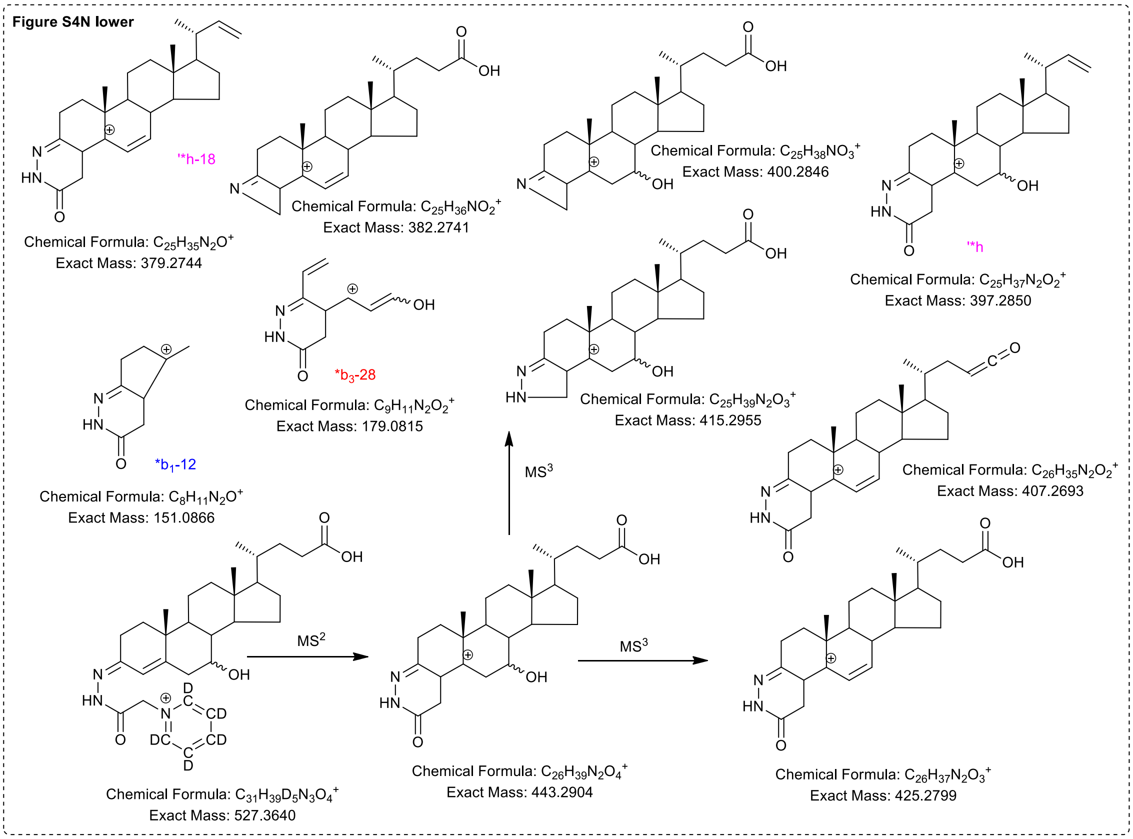
Figure S4N lower
'*h-18
Chemical Formula: C25H35N2O+
Exact Mass: 379.2744
Chemical Formula: C25H36NO2+
Exact Mass: 382.2741
Chemical Formula: C25H38NO3+
Exact Mass: 400.2846
'*h
Chemical Formula: C25H37N2O2+
Exact Mass: 397.2850
*b3-28
Chemical Formula: C9H11N2O2+
Exact Mass: 179.0815
Chemical Formula: C25H39N2O3+
Exact Mass: 415.2955
*b1-12
Chemical Formula: C8H11N2O+
Exact Mass: 151.0866
MS3
Chemical Formula: C26H35N2O2+
Exact Mass: 407.2693
MS2
MS3
Chemical Formula: C31H39D5N3O4+
Exact Mass: 527.3640
Chemical Formula: C26H39N2O4+
Exact Mass: 443.2904
Chemical Formula: C26H37N2O3+
Exact Mass: 425.2799

S4O

Figure S4O

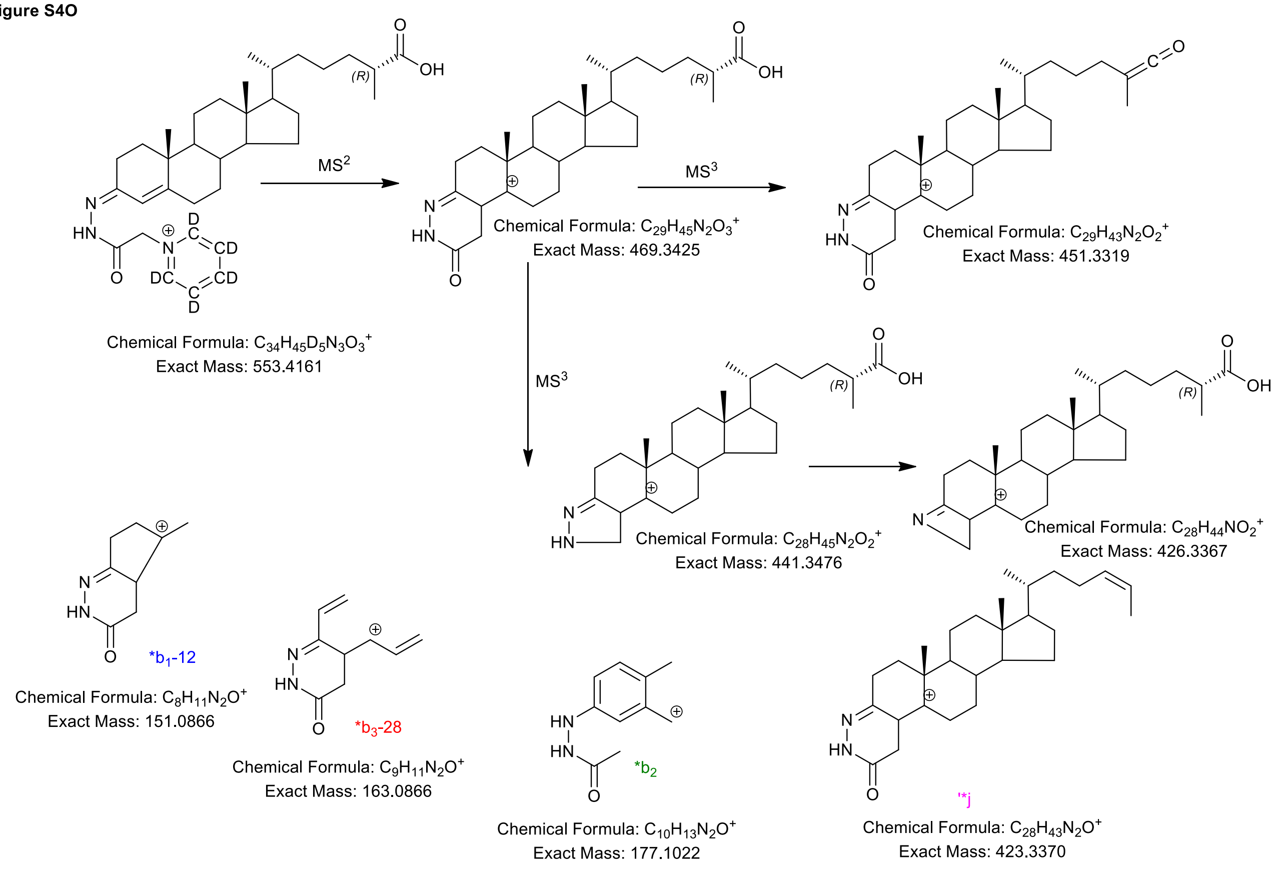

S4P

Figure 4P

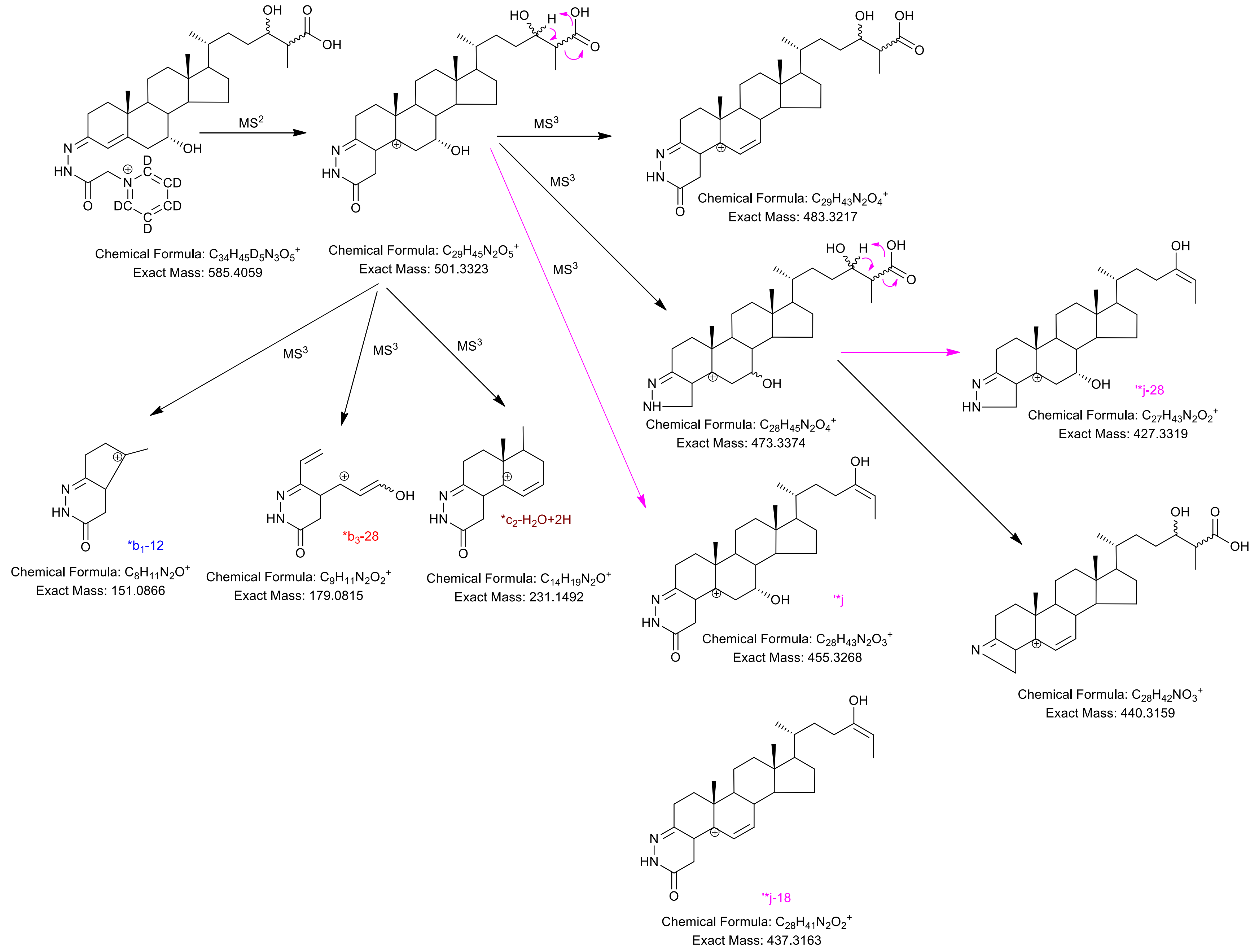

S4Q

Figure 4Q

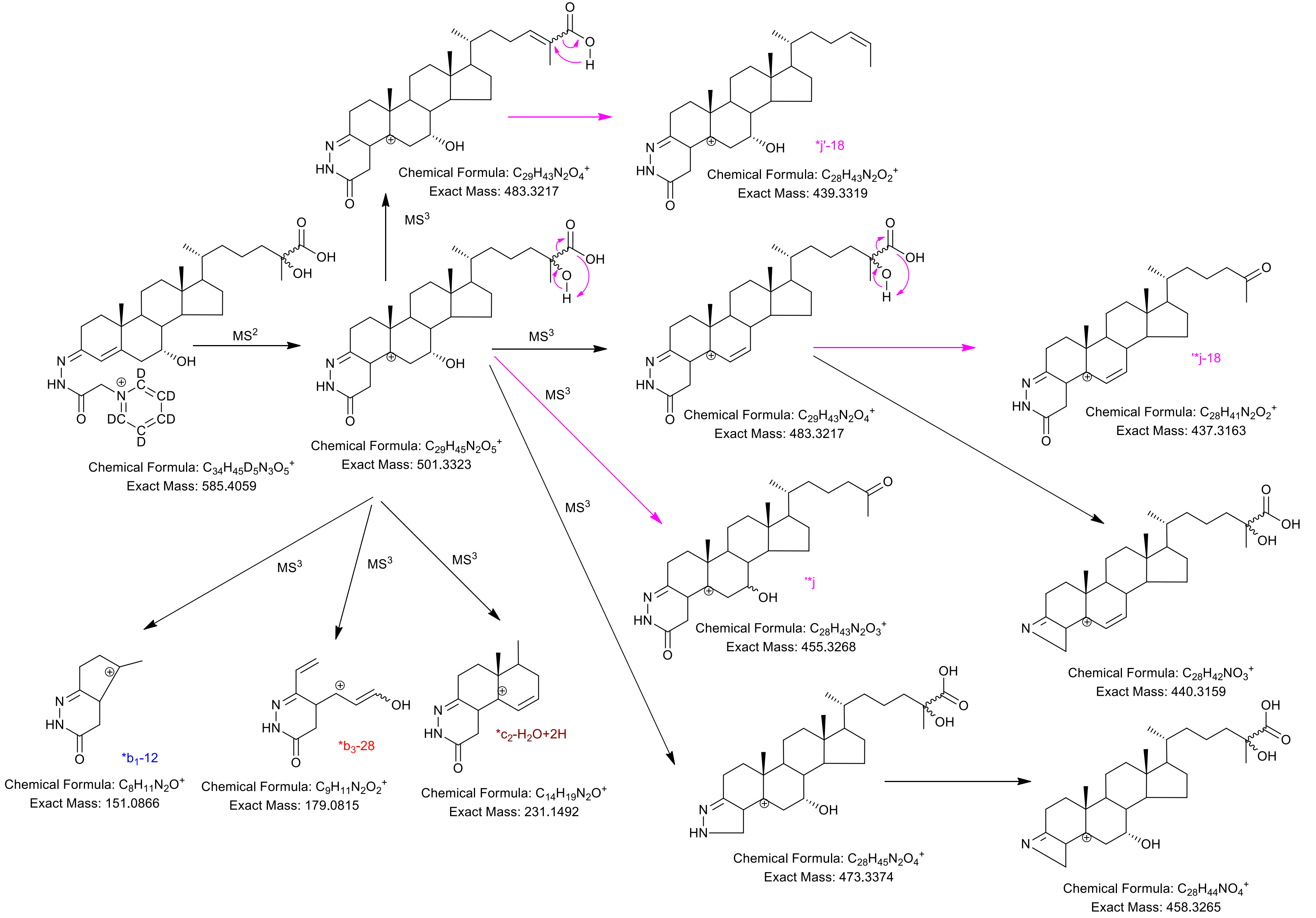

S4R

Figure S4R

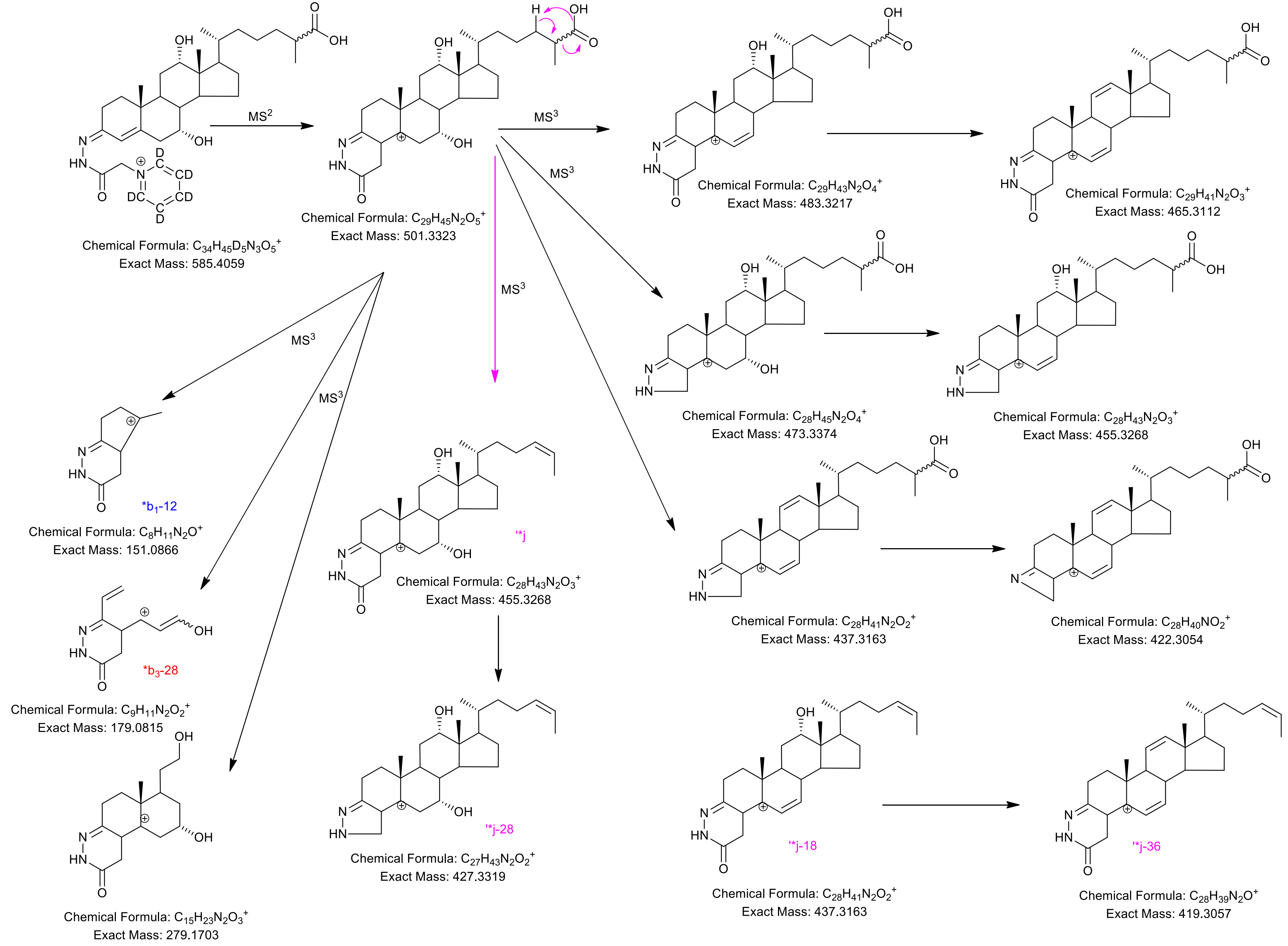

S4S

Figure S4S

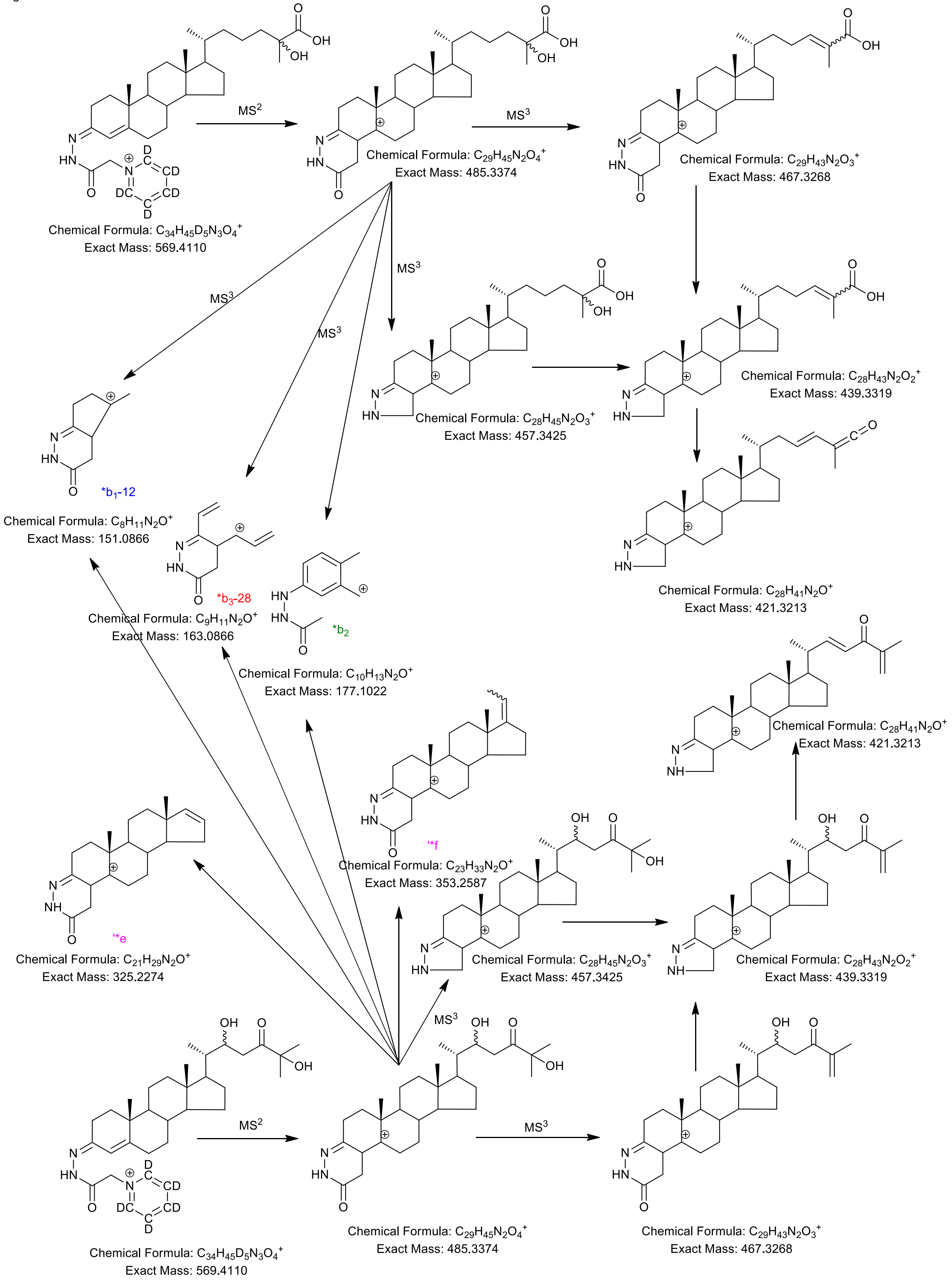

S4T

Figure S4T

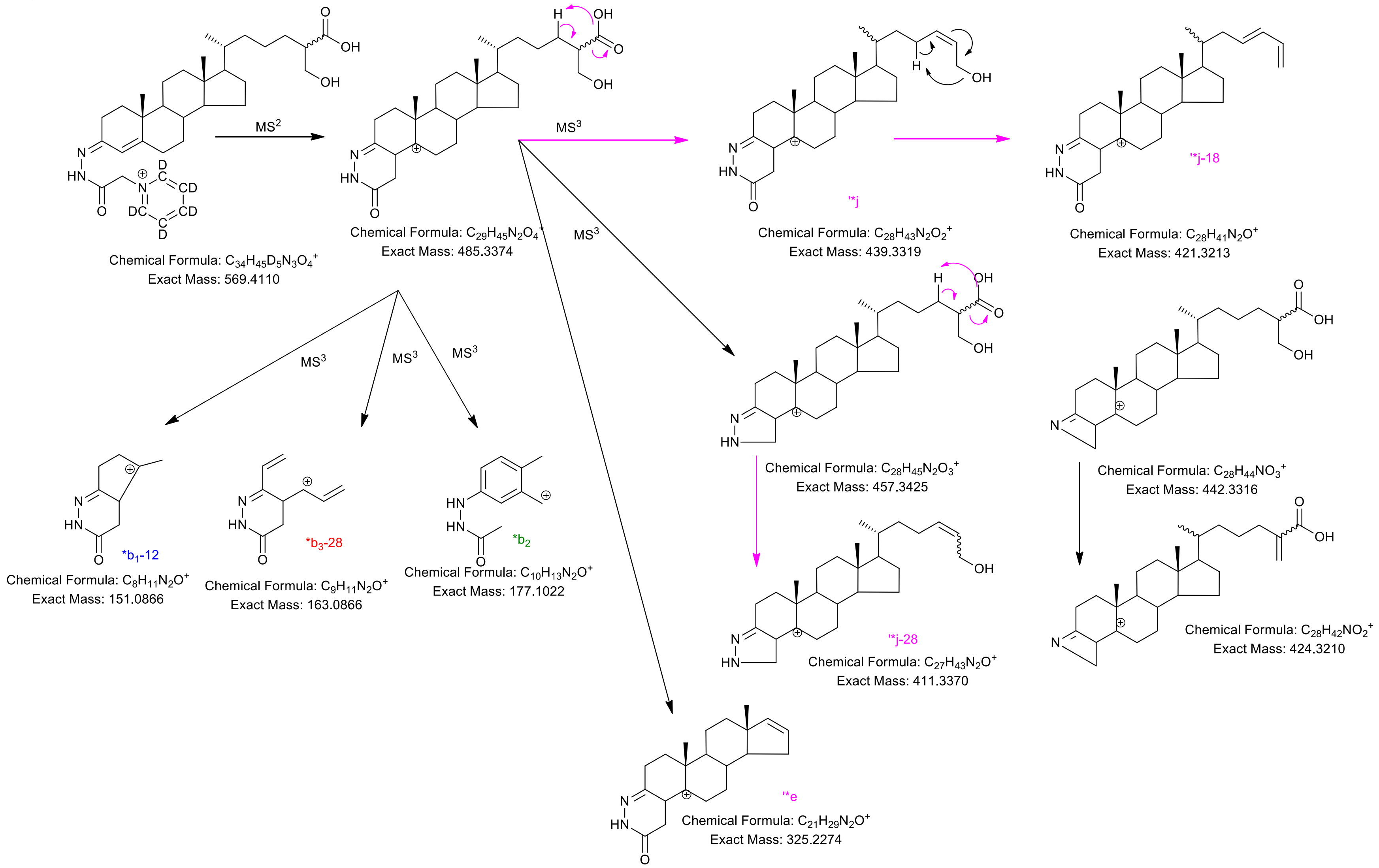

S4U

Figure S4U

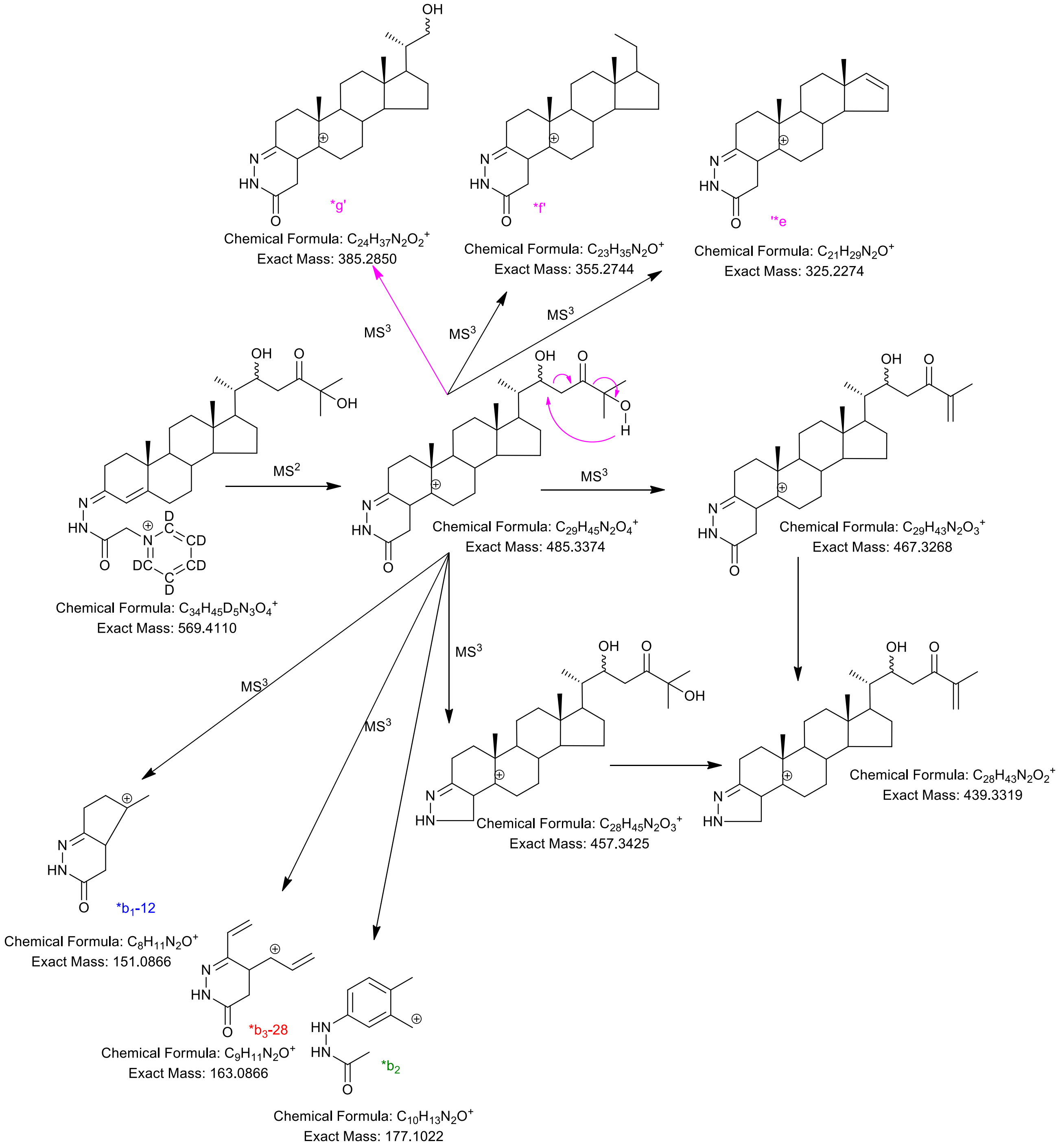

S4V

Figure S4V

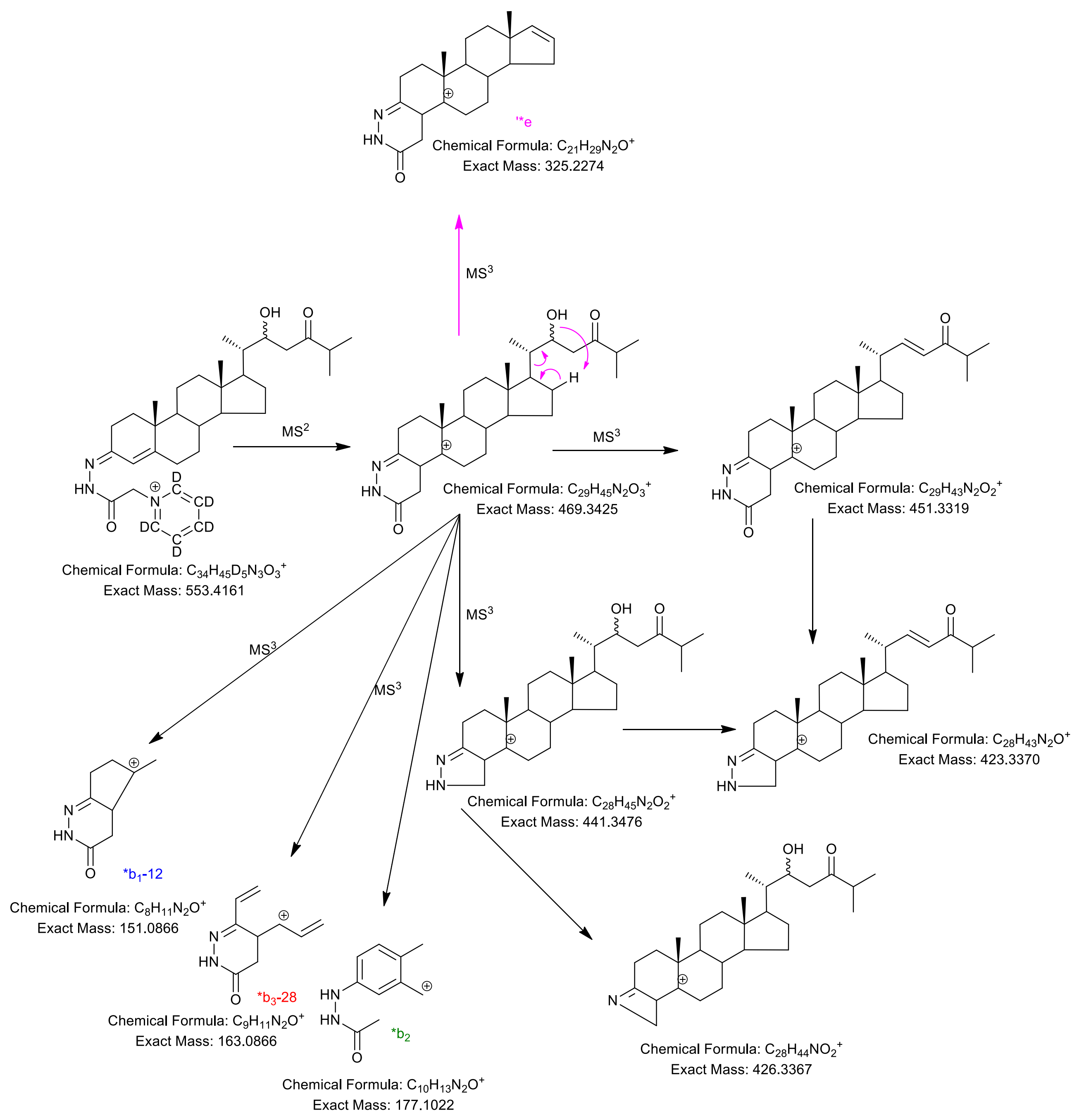

S5A

EY_191014_100uL-NIST-QC_100uL-OxySpla... 10/15/19 02:23:26
100uL NIST (2018) + 100ul OxysterolSPLASH + 10ng 22S-HCO-D7 + 20ug

EY_191014_100uL-NIST-QC_100uL-OxySplash_rep2of5_Fr1A=GPd5_Fr1E 9.13 AV: 1 NL: 5.83E2
F: ITMS + c ESI Full ms3 539.44@cid30.00 455.36@cid35.00 [125.00-550.0

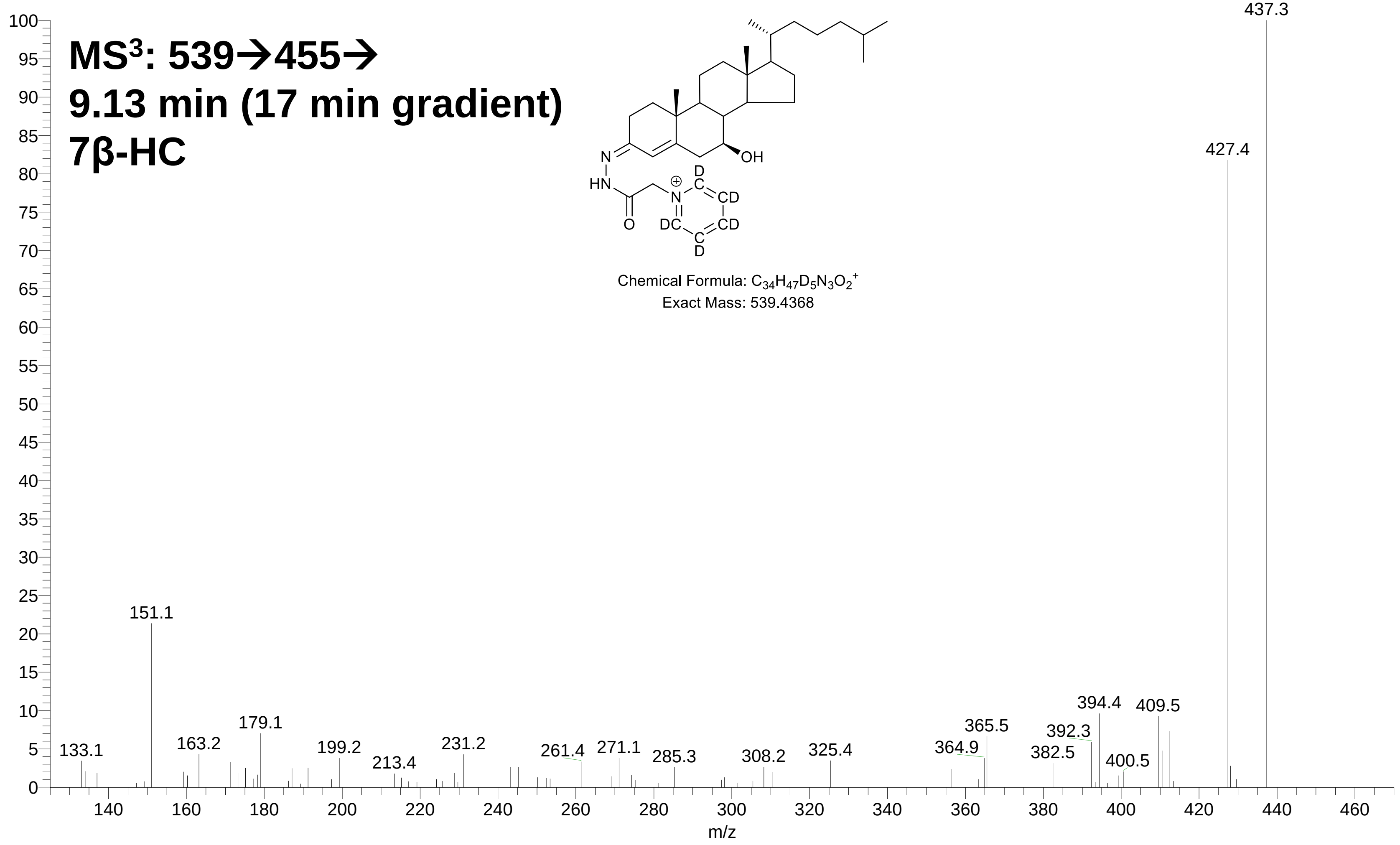

S5B

EY_191014_100uL-NIST-QC_100uL-OxySpla... 10/15/19 02:23:26
100uL NIST (2018) + 100ul OxysterolSPLASH + 10ng 22S-HCO-D7 + 20ug

EY_191014_100uL-NIST-QC_100uL-OxySplash_rep2of5_Fr1A=GPd5_Fr1E 9.10 AV: 1 NL: 1.77E3
F: ITMS + c ESI Full ms3 546.48@cid30.00 462.41@cid35.00 [125.00-555.0

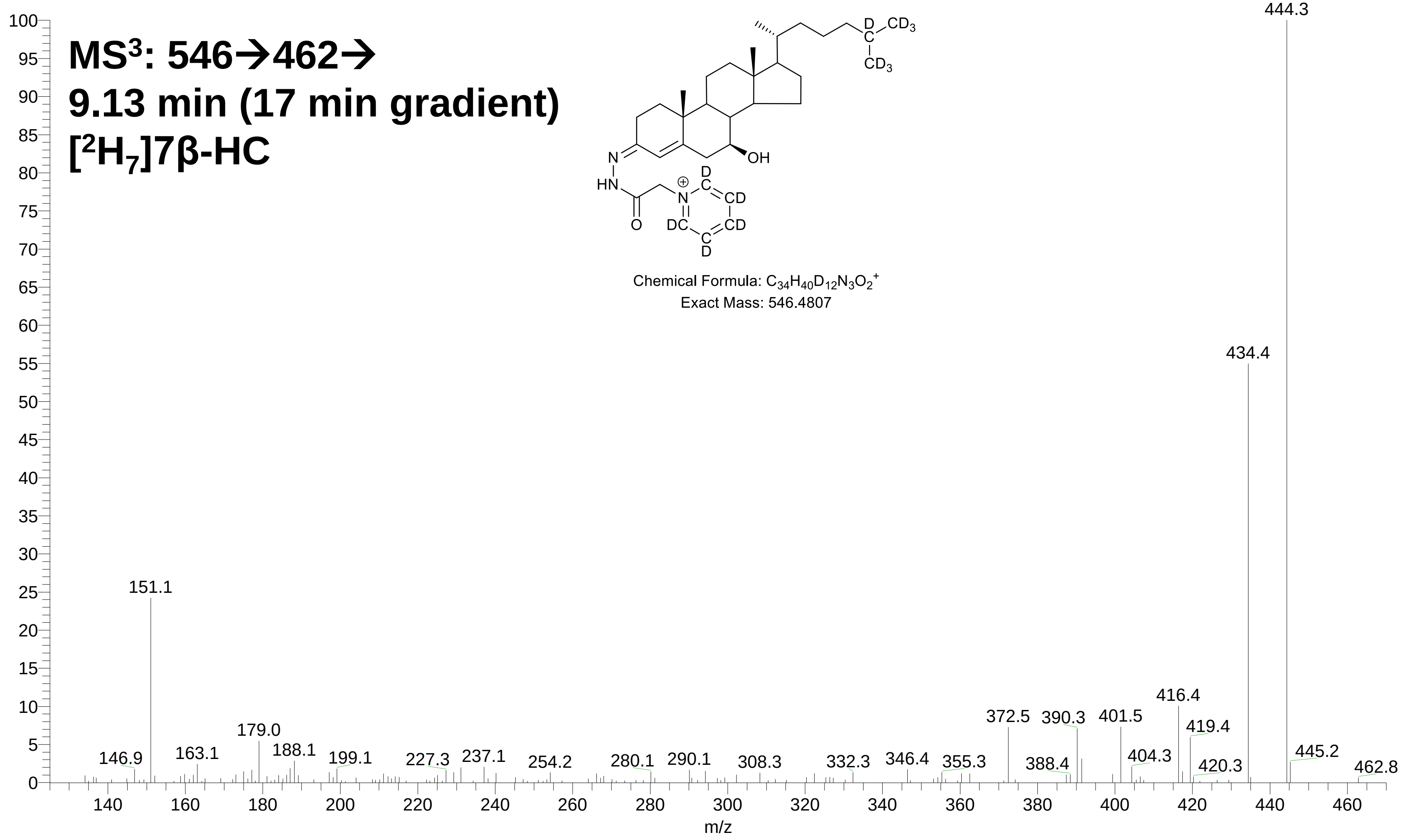

S5C

EY_191014_100uL-NIST-QC_100uL-OxySpla... 10/15/19 02:23:26
100uL NIST (2018) + 100ul OxysterolSPLASH + 10ng 22S-HCO-D7 + 20ug
EY_191014_100uL-NIST-QC_100uL-OxySplash_rep2of5_Fr1A=GPd5_Fr1E 9.70 AV: 1 NL: 1.19E4
F: ITMS + c ESI Full ms3 539.44@cid30.00 455.36@cid35.00 [125.00-550.0

MS^3: 539→455→
9.70 min (17 min gradient)
7α-HC

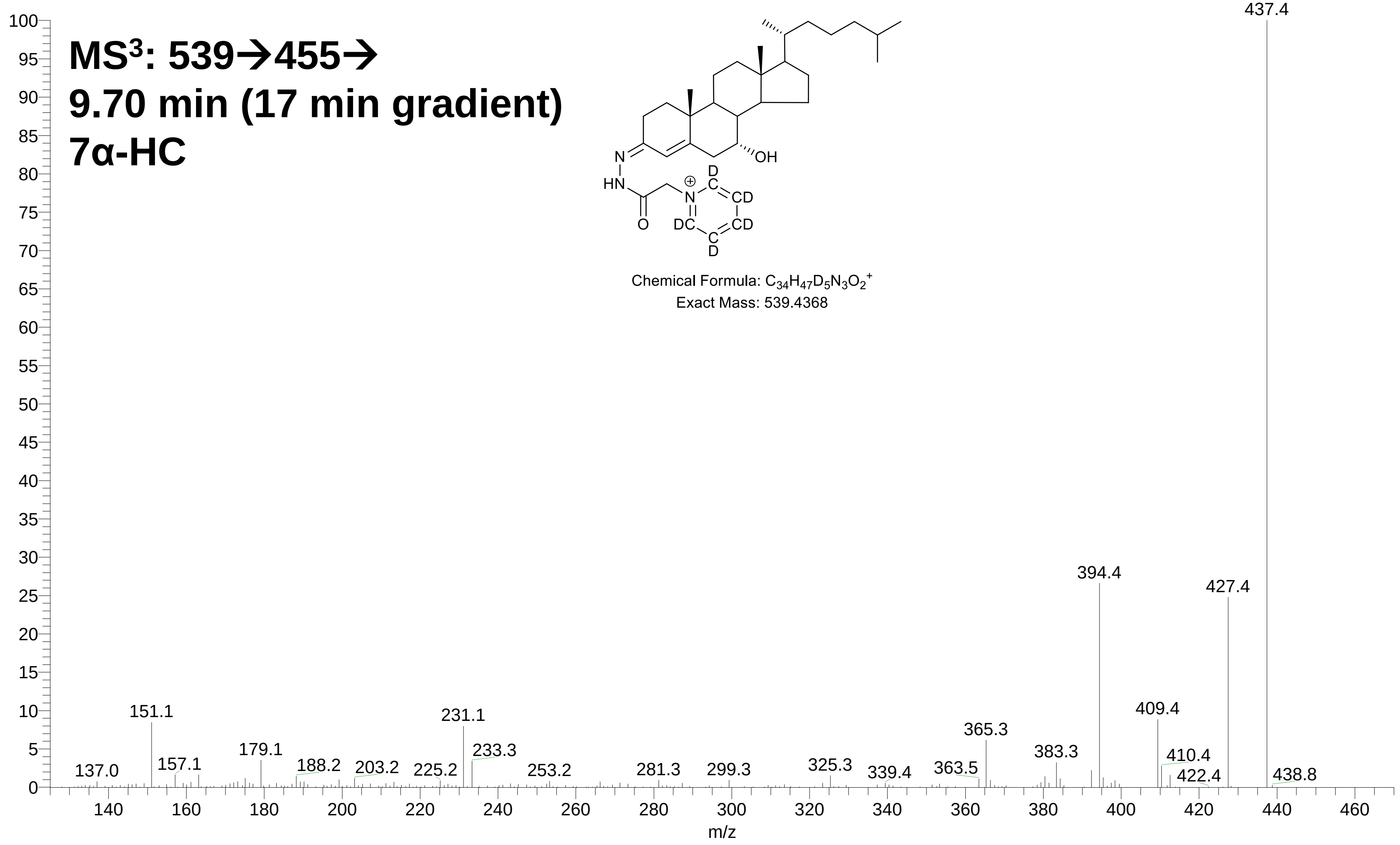

S5D

EY_191014_100uL-NIST-QC_100uL-OxySpla... 10/15/19 02:23:26
100uL NIST (2018) + 100ul OxysterolSPLASH + 10ng 22S-HCO-D7 + 20ug
EY_191014_100uL-NIST-QC_100uL-OxySplash_rep2of5_Fr1A=GPd5_Fr1E 9.66 AV: 1 NL: 2.06E4
F: ITMS + c ESI Full ms3 546.48@cid30.00 462.41@cid35.00 [125.00-555.0

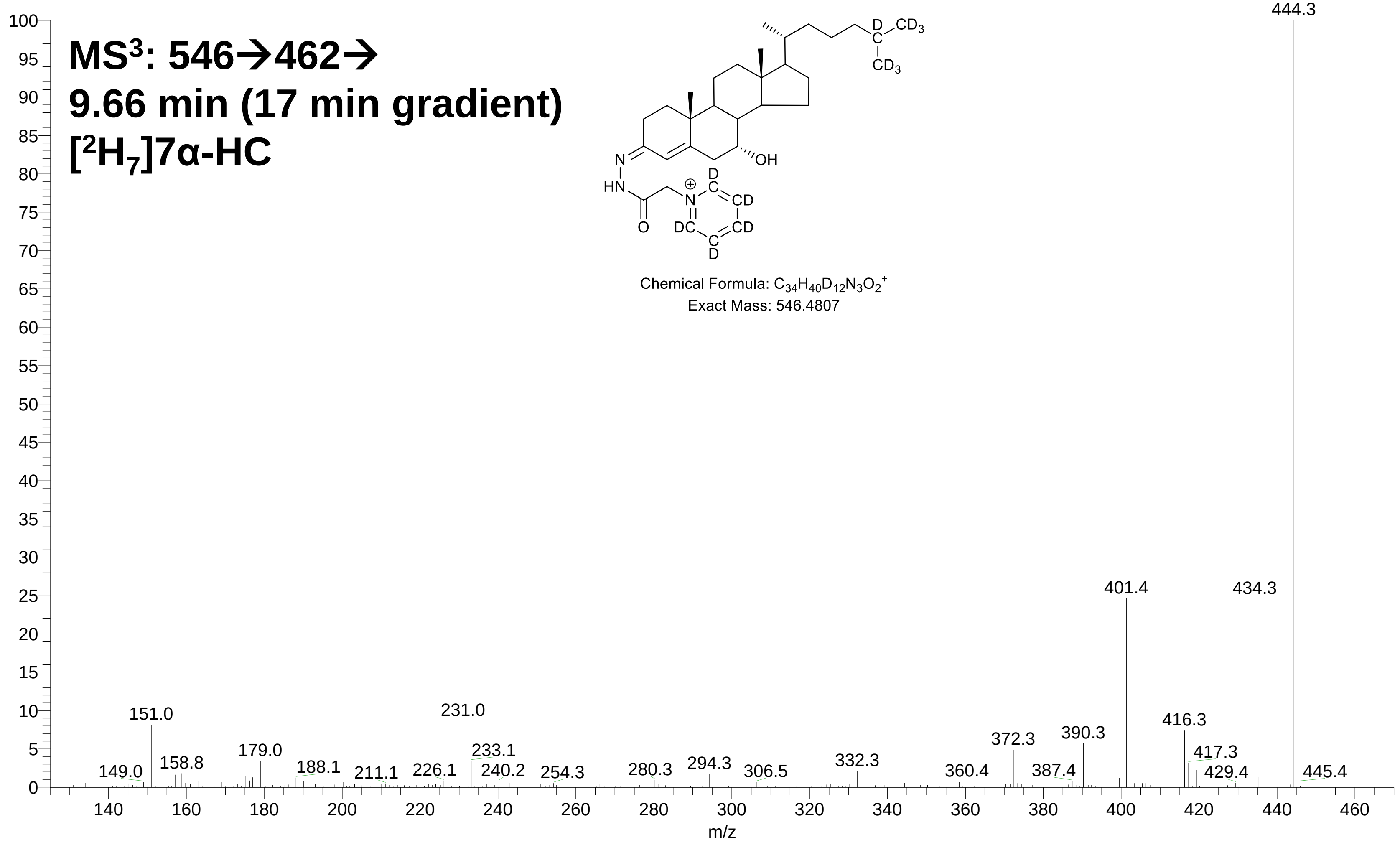

S5E

EY_191014_100uL-NIST-QC_100uL-OxySpla... 10/15/19 02:23:26
100uL NIST (2018) + 100ul OxysterolSPLASH + 10ng 22S-HCO-D7 + 20ug
EY_191014_100uL-NIST-QC_100uL-OxySplash_rep2of5_Fr1A=GPd5_Fr1E 9.97 AV: 1 NL: 1.11E3
F: ITMS + c ESI Full ms3 539.44@cid30.00 455.36@cid35.00 [125.00-550.0

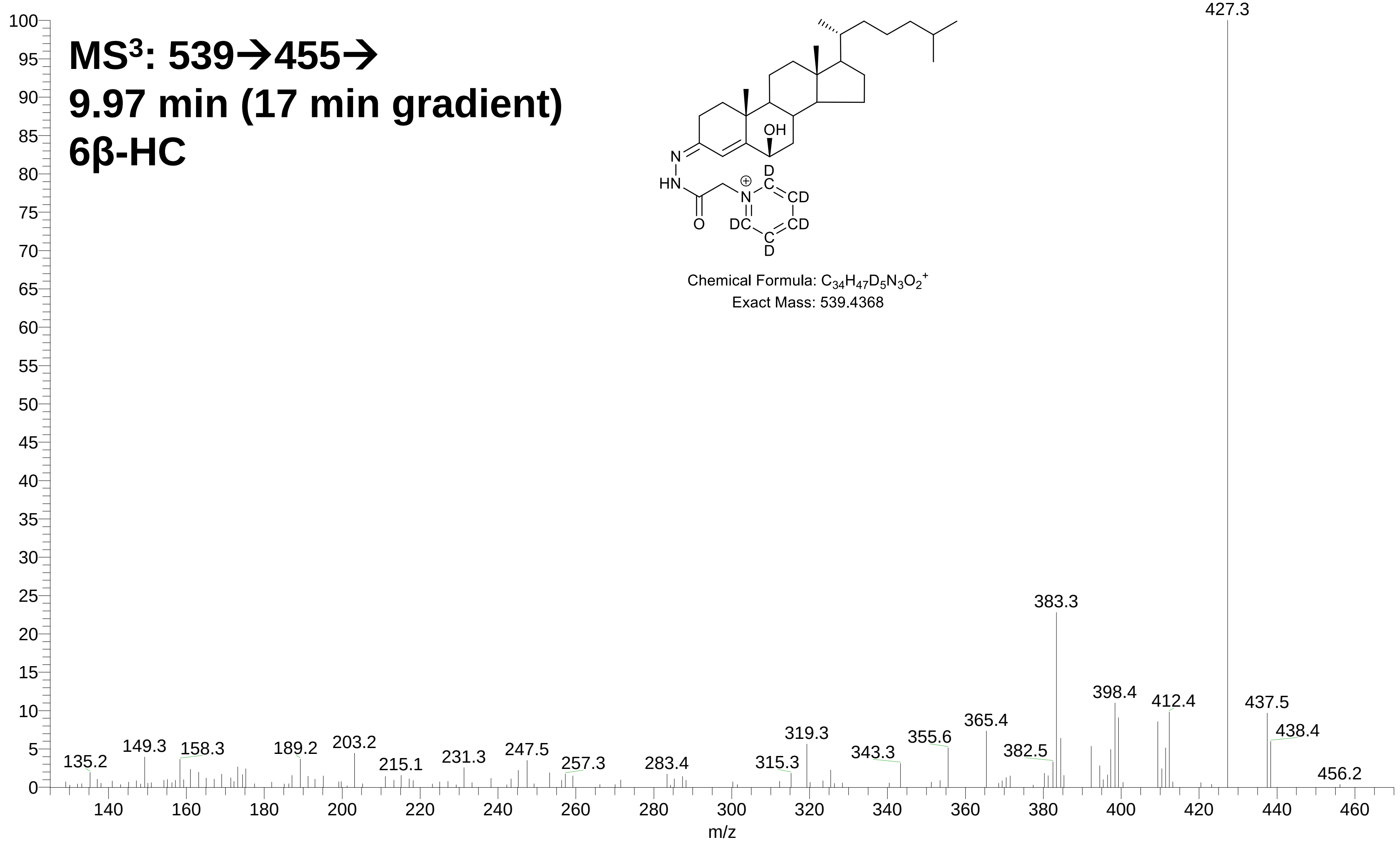

S5F

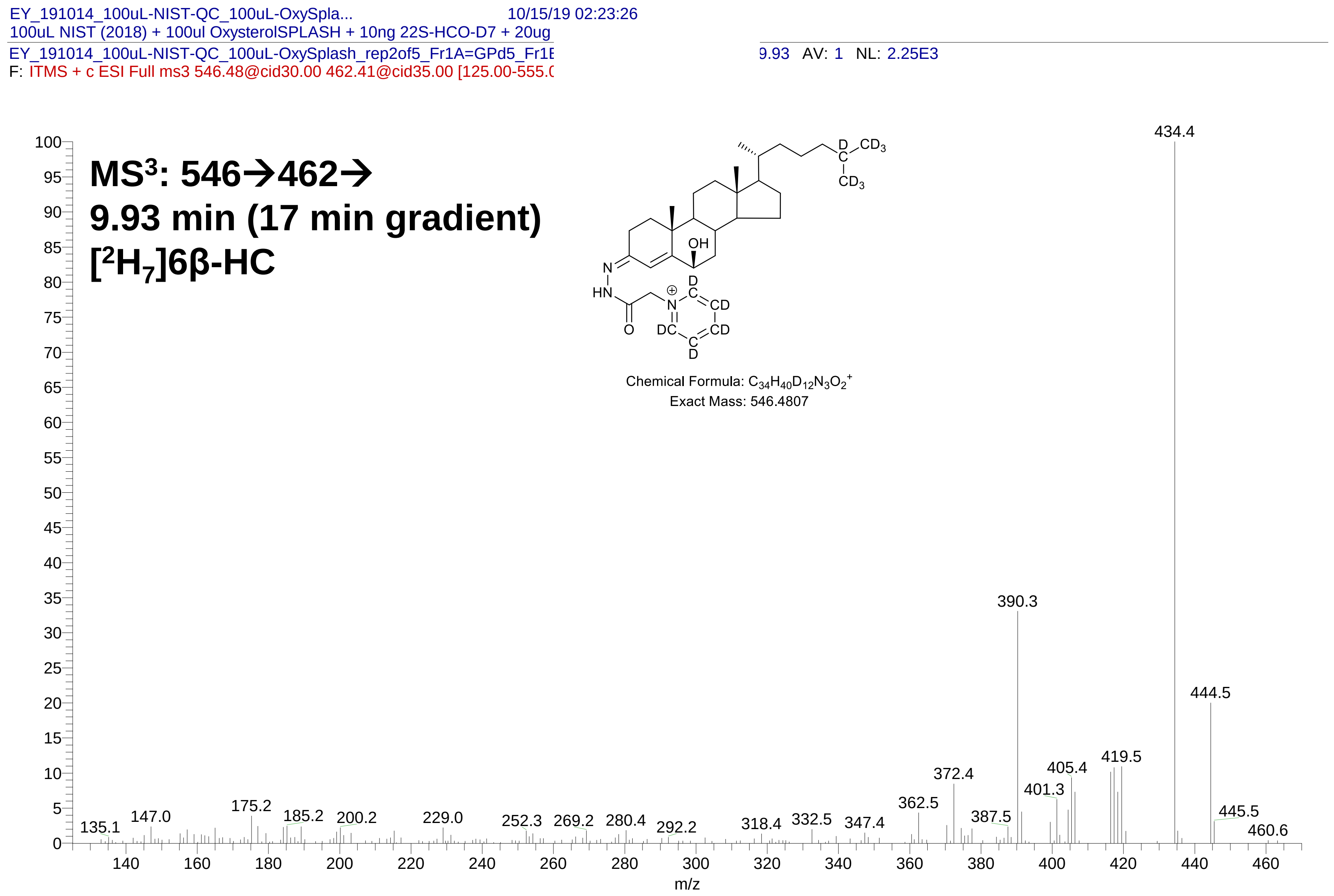
EY_191014_100uL-NIST-QC_100uL-OxySpla...
10/15/19 02:23:26
100uL NIST (2018) + 100ul OxysterolSPLASH + 10ng 22S-HCO-D7 + 20ug
EY_191014_100uL-NIST-QC_100uL-OxySplash_rep2of5_Fr1A=GPd5_Fr1E
9.93 AV: 1 NL: 2.25E3
F: ITMS + c ESI Full ms3 546.48@cid30.00 462.41@cid35.00 [125.00-555.0
MS3: 546→462→
9.93 min (17 min gradient)
[2H7]6β-HC
Chemical Formula: C34H40D12N3O2+
Exact Mass: 546.4807
434.4
390.3
444.5
419.5
405.4
401.3
372.4
362.5
387.5
445.5
460.6
347.4
332.5
318.4
292.2
280.4
269.2
252.3
229.0
200.2
185.2
175.2
147.0
135.1
m/z

S5G

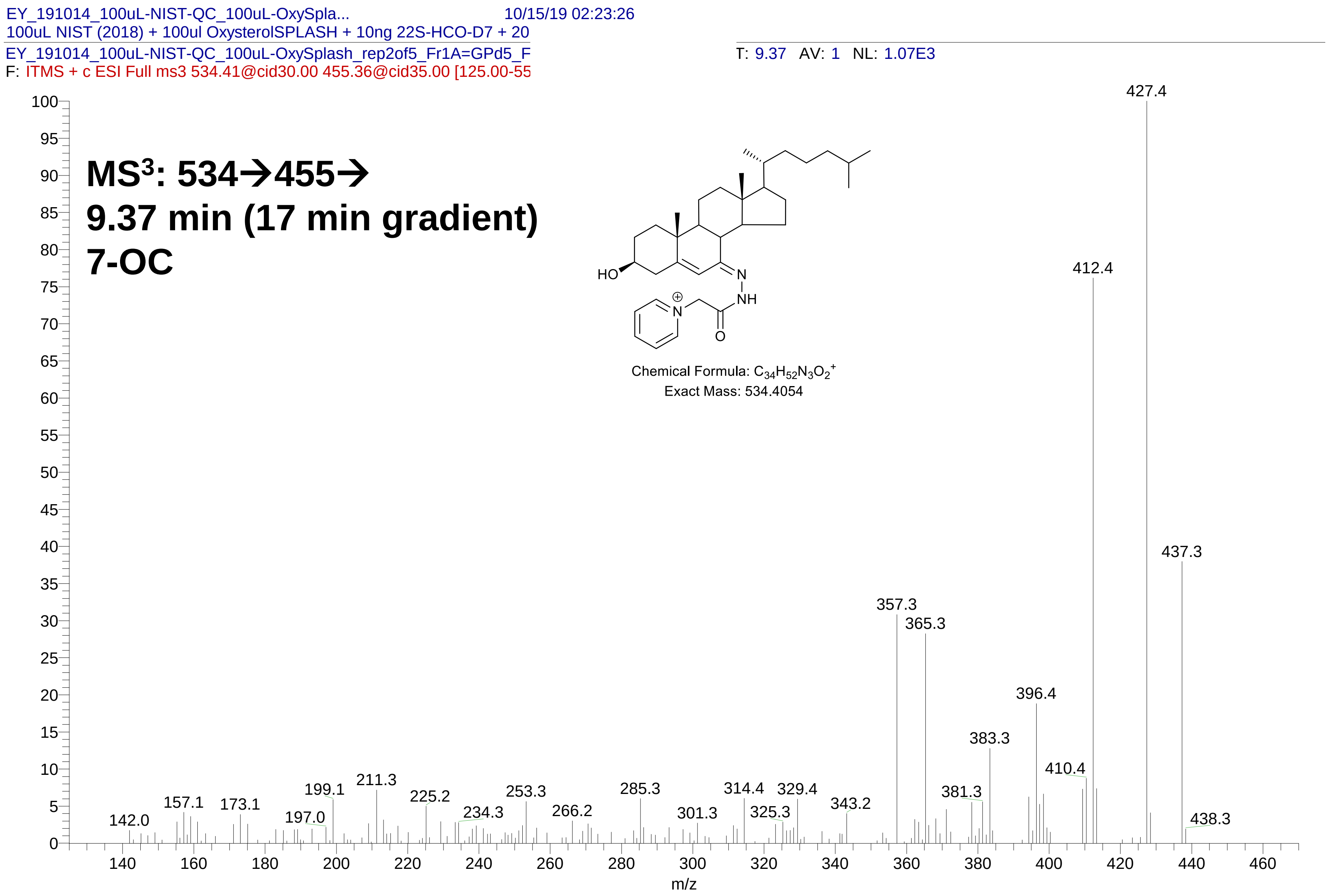
EY_191014_100uL-NIST-QC_100uL-OxySpla...
10/15/19 02:23:26
100uL NIST (2018) + 100ul OxysterolSPLASH + 10ng 22S-HCO-D7 + 20
EY_191014_100uL-NIST-QC_100uL-OxySplash_rep2of5_Fr1A=GPd5_F
F: ITMS + c ESI Full ms3 534.41@cid30.00 455.36@cid35.00 [125.00-55
T: 9.37 AV: 1 NL: 1.07E3
MS³: 534→455→
9.37 min (17 min gradient)
7-OC
HO
N
NH
N
O
Chemical Formula: C34H52N3O2+
Exact Mass: 534.4054
427.4
412.4
437.3
357.3
365.3
396.4
383.3
410.4
381.3
343.2
329.4
314.4
325.3
301.3
285.3
266.2
253.3
234.3
225.2
211.3
199.1
197.0
173.1
157.1
142.0
m/z

S5H

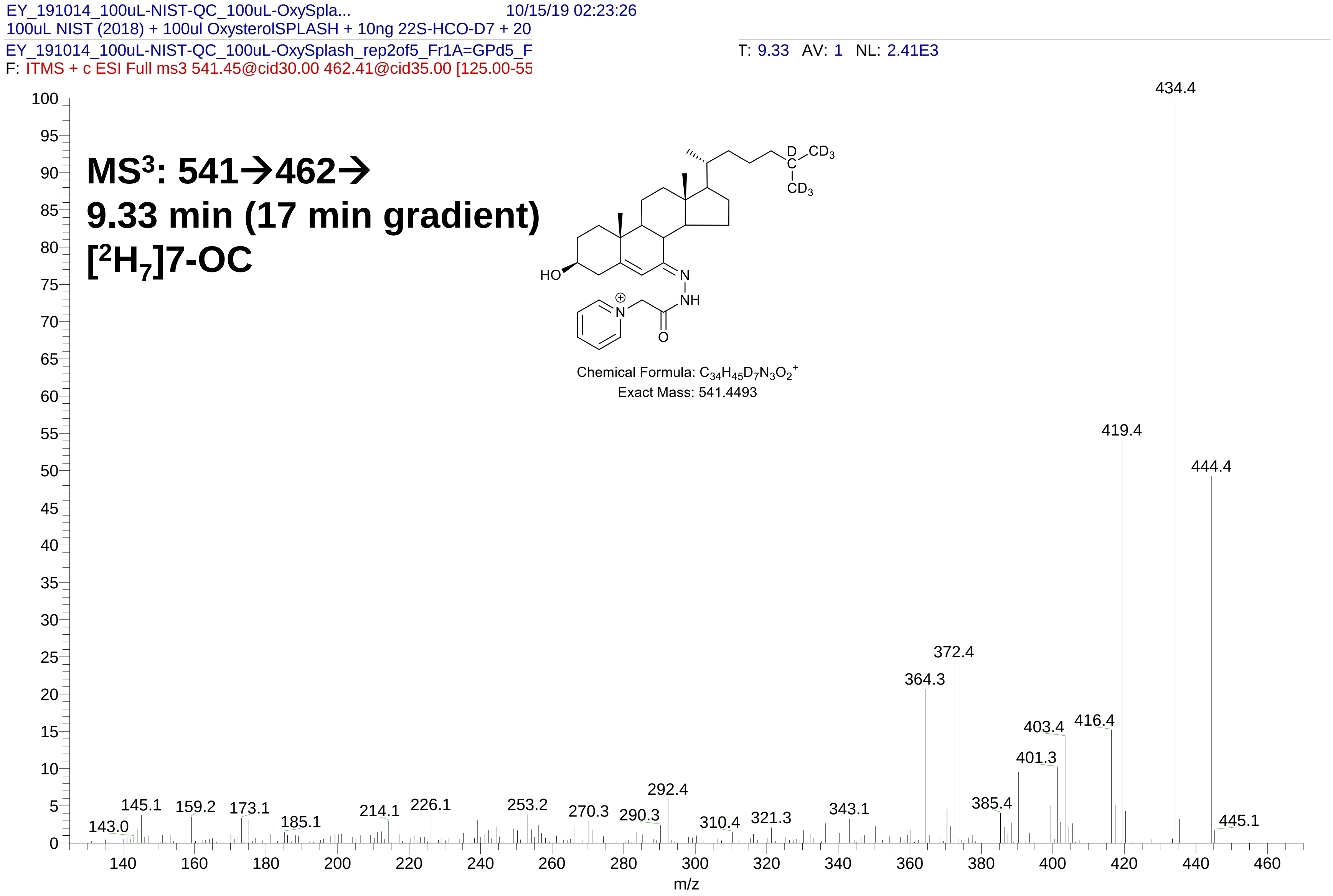

EY_191014_100uL-NIST-QC_100uL-OxySpla...
10/15/19 02:23:26
100uL NIST (2018) + 100ul OxysterolSPLASH + 10ng 22S-HCO-D7 + 20
EY_191014_100uL-NIST-QC_100uL-OxySplash_rep2of5_Fr1A=GPd5_F
T: 9.33 AV: 1 NL: 2.41E3
F: ITMS + c ESI Full ms3 541.45@cid30.00 462.41@cid35.00 [125.00-55
MS3: 541→462→
9.33 min (17 min gradient)
[2H7]7-OC
Chemical Formula: C34H45D7N3O2+
Exact Mass: 541.4493
434.4
419.4
444.4
372.4
364.3
403.4
416.4
401.3
292.4
385.4
145.1
159.2
173.1
214.1
226.1
253.2
270.3
290.3
343.1
321.3
310.4
185.1
143.0
445.1
m/z

S5I

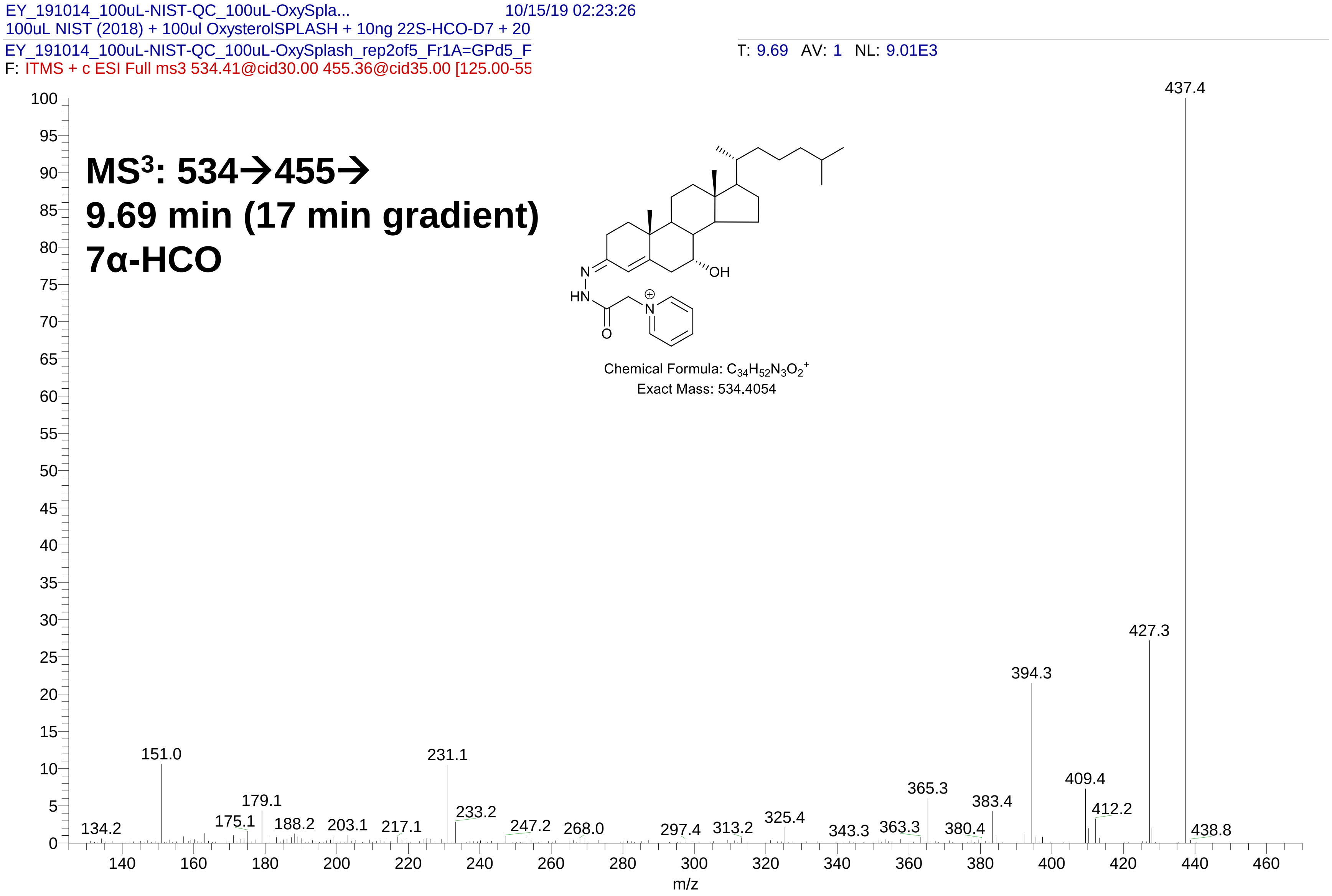
EY_191014_100uL-NIST-QC_100uL-OxySpla...
10/15/19 02:23:26
100uL NIST (2018) + 100ul OxysterolSPLASH + 10ng 22S-HCO-D7 + 20
EY_191014_100uL-NIST-QC_100uL-OxySplash_rep2of5_Fr1A=GPd5_F
F: ITMS + c ESI Full ms3 534.41@cid30.00 455.36@cid35.00 [125.00-55
T: 9.69 AV: 1 NL: 9.01E3
MS³: 534→455→
9.69 min (17 min gradient)
7α-HCO
Chemical Formula: $C_{34}H_{52}N_3O_2^+$
Exact Mass: 534.4054
N
HN
O
OH
N
437.4
427.3
394.3
409.4
412.2
365.3
383.4
151.0
231.1
179.1
175.1
188.2
203.1
217.1
233.2
247.2
268.0
297.4
313.2
325.4
343.3
363.3
380.4
134.2
438.8
m/z

S5J

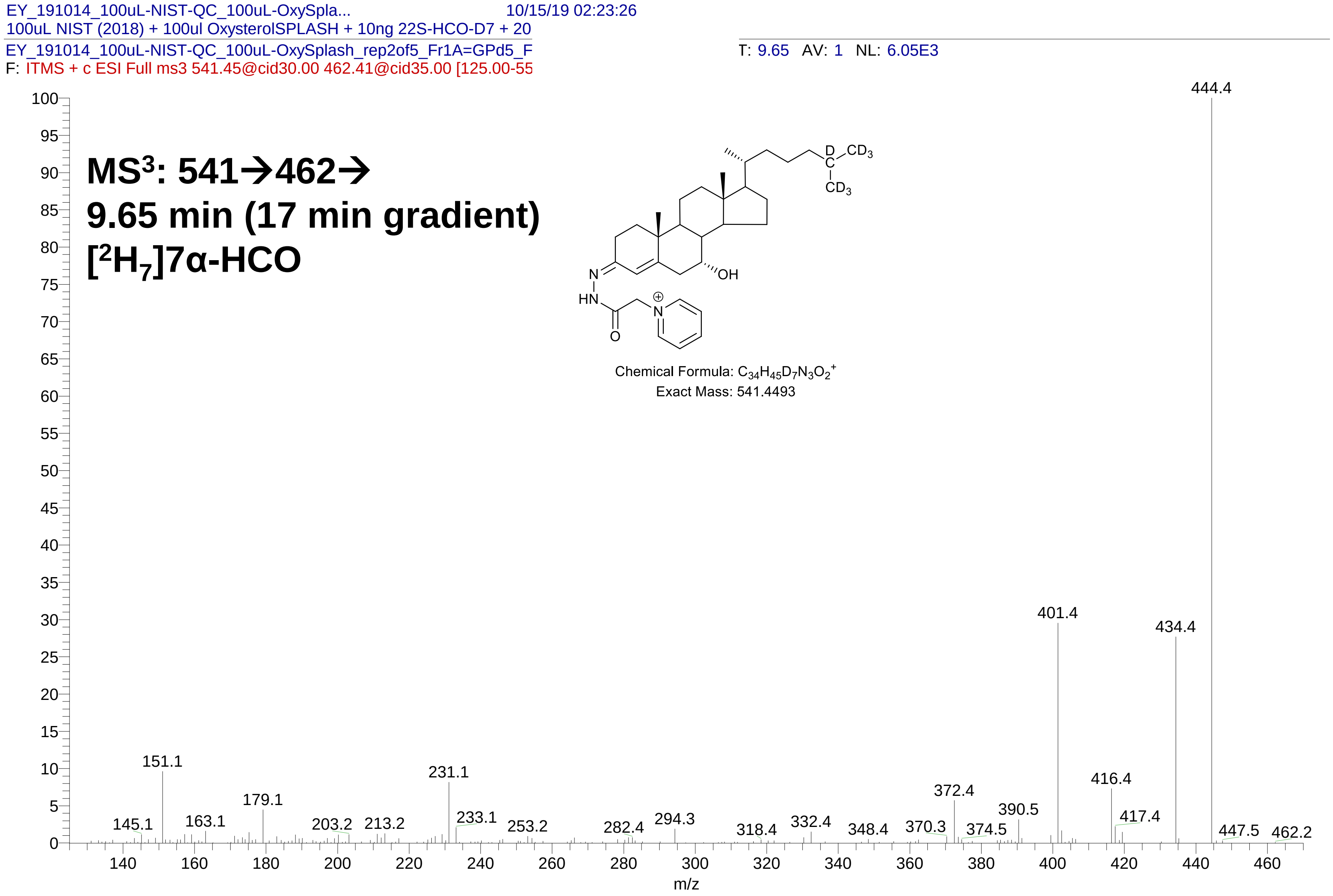

EY_191014_100uL-NIST-QC_100uL-OxySpla...
10/15/19 02:23:26
100uL NIST (2018) + 100ul OxysterolSPLASH + 10ng 22S-HCO-D7 + 20
EY_191014_100uL-NIST-QC_100uL-OxySplash_rep2of5_Fr1A=GPd5_F
F: ITMS + c ESI Full ms3 541.45@cid30.00 462.41@cid35.00 [125.00-55
T: 9.65 AV: 1 NL: 6.05E3
MS3: 541→462→
9.65 min (17 min gradient)
[2H7]7α-HCO
Chemical Formula: C34H45D7N3O2+
Exact Mass: 541.4493
444.4
401.4
434.4
416.4
372.4
390.5
417.4
151.1
231.1
179.1
145.1
163.1
203.2
213.2
233.1
253.2
282.4
294.3
318.4
332.4
348.4
370.3
374.5
447.5
462.2
m/z

S5K

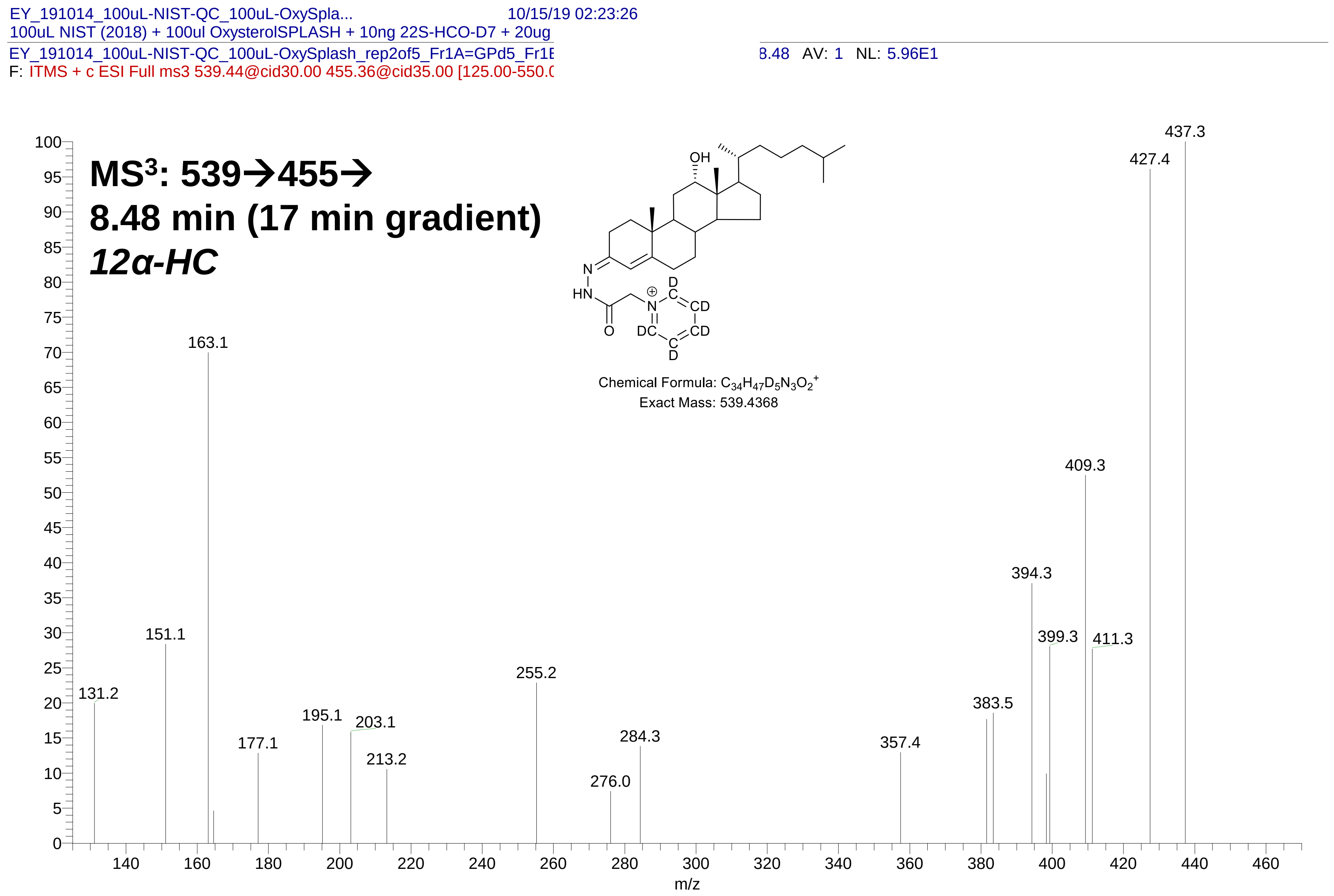
EY_191014_100uL-NIST-QC_100uL-OxySpla... 10/15/19 02:23:26
100uL NIST (2018) + 100ul OxysterolSPLASH + 10ng 22S-HCO-D7 + 20ug
EY_191014_100uL-NIST-QC_100uL-OxySplash_rep2of5_Fr1A=GPd5_Fr1E
8.48 AV: 1 NL: 5.96E1
F: ITMS + c ESI Full ms3 539.44@cid30.00 455.36@cid35.00 [125.00-550.0
MS3: 539→455→
8.48 min (17 min gradient)
12α-HC
OH
N
HN
O
D
C
CD
N
DC
CD
C
D
Chemical Formula: C34H47D5N3O2+
Exact Mass: 539.4368
437.3
427.4
409.3
394.3
399.3
411.3
383.5
357.4
284.3
276.0
255.2
213.2
203.1
195.1
177.1
163.1
151.1
131.2
m/z

S6A

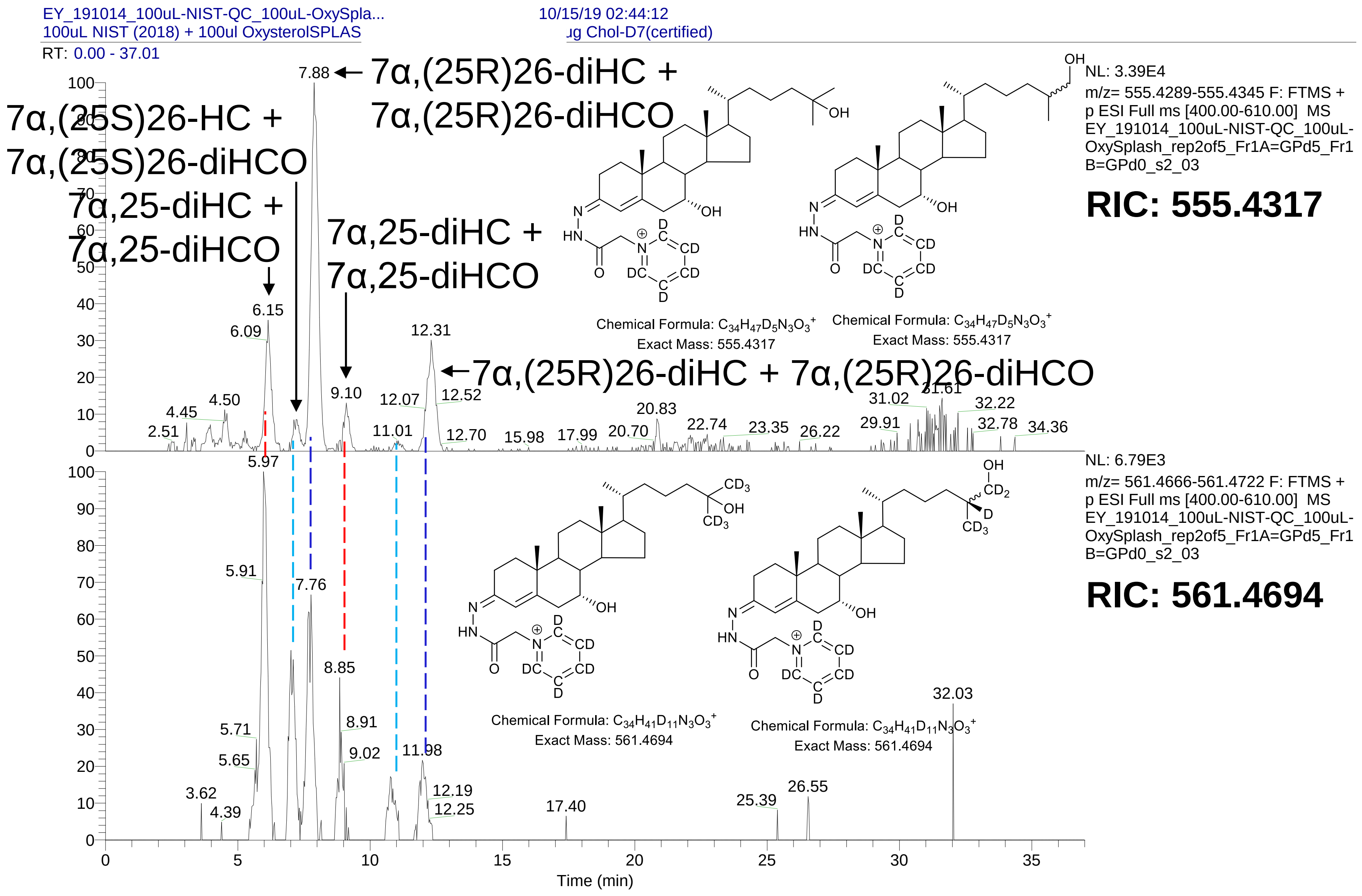
EY_191014_100uL-NIST-QC_100uL-OxySpla...
100uL NIST (2018) + 100ul OxysterolSPLAS
10/15/19 02:44:12
ıg Chol-D7(certified)
RT: 0.00 - 37.01
7α,(25R)26-diHC +
7α,(25R)26-diHCO
7α,(25S)26-HC +
7α,(25S)26-diHCO
7α,25-diHC +
7α,25-diHCO
7α,25-diHC +
7α,25-diHCO
7α,(25R)26-diHC + 7α,(25R)26-diHCO
NL: 3.39E4
m/z= 555.4289-555.4345 F: FTMS + p ESI Full ms [400.00-610.00] MS EY_191014_100uL-NIST-QC_100uL-OxySplash_rep2of5_Fr1A=GPd5_Fr1B=GPd0_s2_03
RIC: 555.4317
Chemical Formula: C34H47D5N3O3+
Exact Mass: 555.4317
Chemical Formula: C34H47D5N3O3+
Exact Mass: 555.4317
NL: 6.79E3
m/z= 561.4666-561.4722 F: FTMS + p ESI Full ms [400.00-610.00] MS EY_191014_100uL-NIST-QC_100uL-OxySplash_rep2of5_Fr1A=GPd5_Fr1B=GPd0_s2_03
RIC: 561.4694
Chemical Formula: C34H41D11N3O3+
Exact Mass: 561.4694
Chemical Formula: C34H41D11N3O3+
Exact Mass: 561.4694
Time (min)

S6B

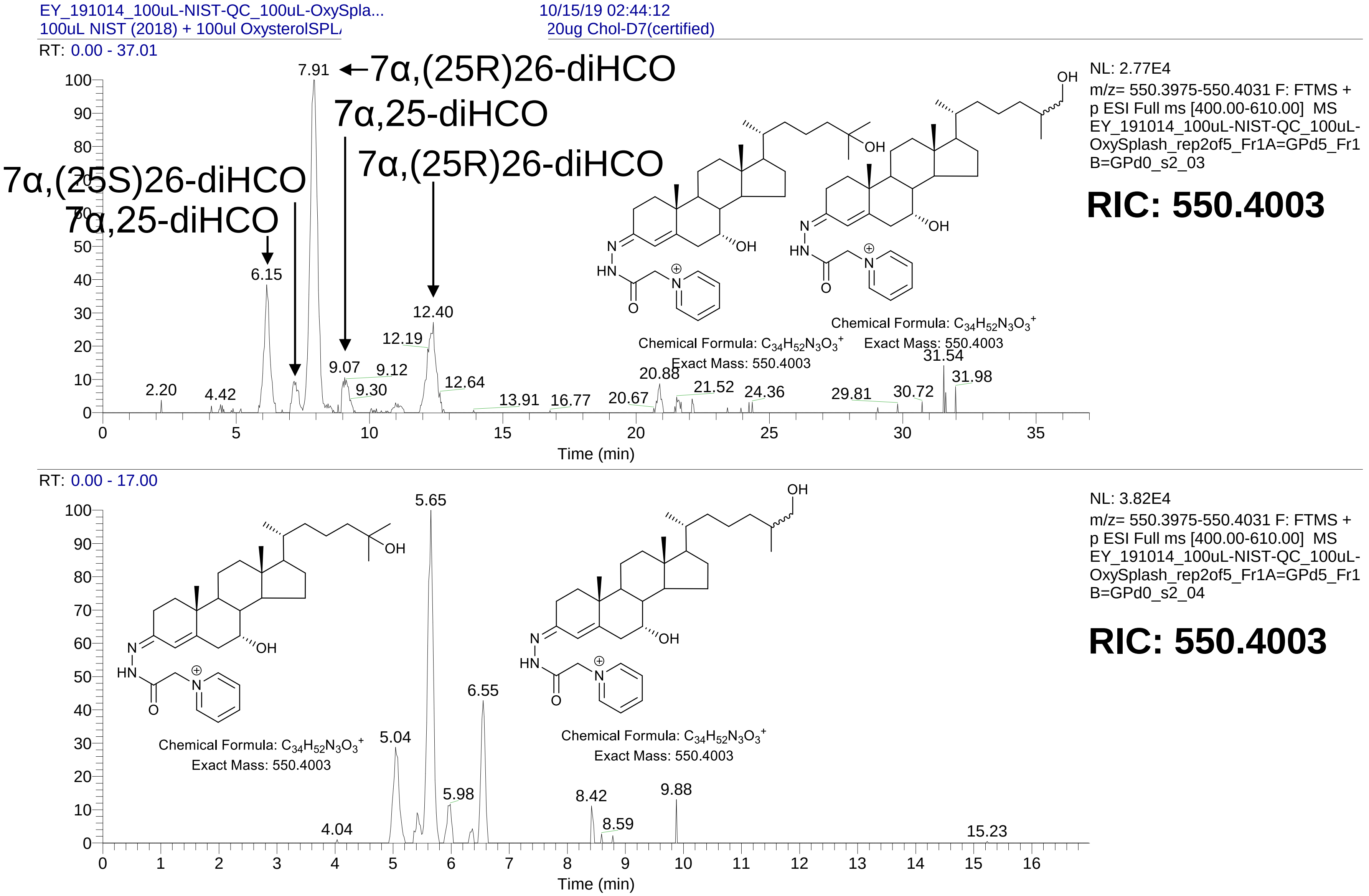

EY_191014_100uL-NIST-QC_100uL-OxySpla...
100uL NIST (2018) + 100ul OxysterolSPL
10/15/19 02:44:12
20ug Chol-D7(certified)
RT: 0.00 - 37.01
7.91 ←7α,(25R)26-diHCO
7α,25-diHCO
7α,(25R)26-diHCO
7α,(25S)26-diHCO
7α,25-diHCO
6.15
12.40
12.19
9.07
9.12
9.30
12.64
13.91
16.77
20.67
20.88
21.52
24.36
29.81
30.72
31.54
31.98
2.20
4.42
Time (min)
OH
OH
OH
OH
N
HN
O
N
Chemical Formula: C34H52N3O3+
Exact Mass: 550.4003
NL: 2.77E4
m/z= 550.3975-550.4031 F: FTMS + p ESI Full ms [400.00-610.00] MS EY_191014_100uL-OxySplash_rep2of5_Fr1A=GPd5_Fr1B=GPd0_s2_03
RIC: 550.4003
RT: 0.00 - 17.00
5.65
6.55
5.04
5.98
4.04
8.42
8.59
9.88
15.23
Time (min)
NL: 3.82E4
m/z= 550.3975-550.4031 F: FTMS + p ESI Full ms [400.00-610.00] MS EY_191014_100uL-NIST-QC_100uL-OxySplash_rep2of5_Fr1A=GPd5_Fr1B=GPd0_s2_04
RIC: 550.4003

S6C

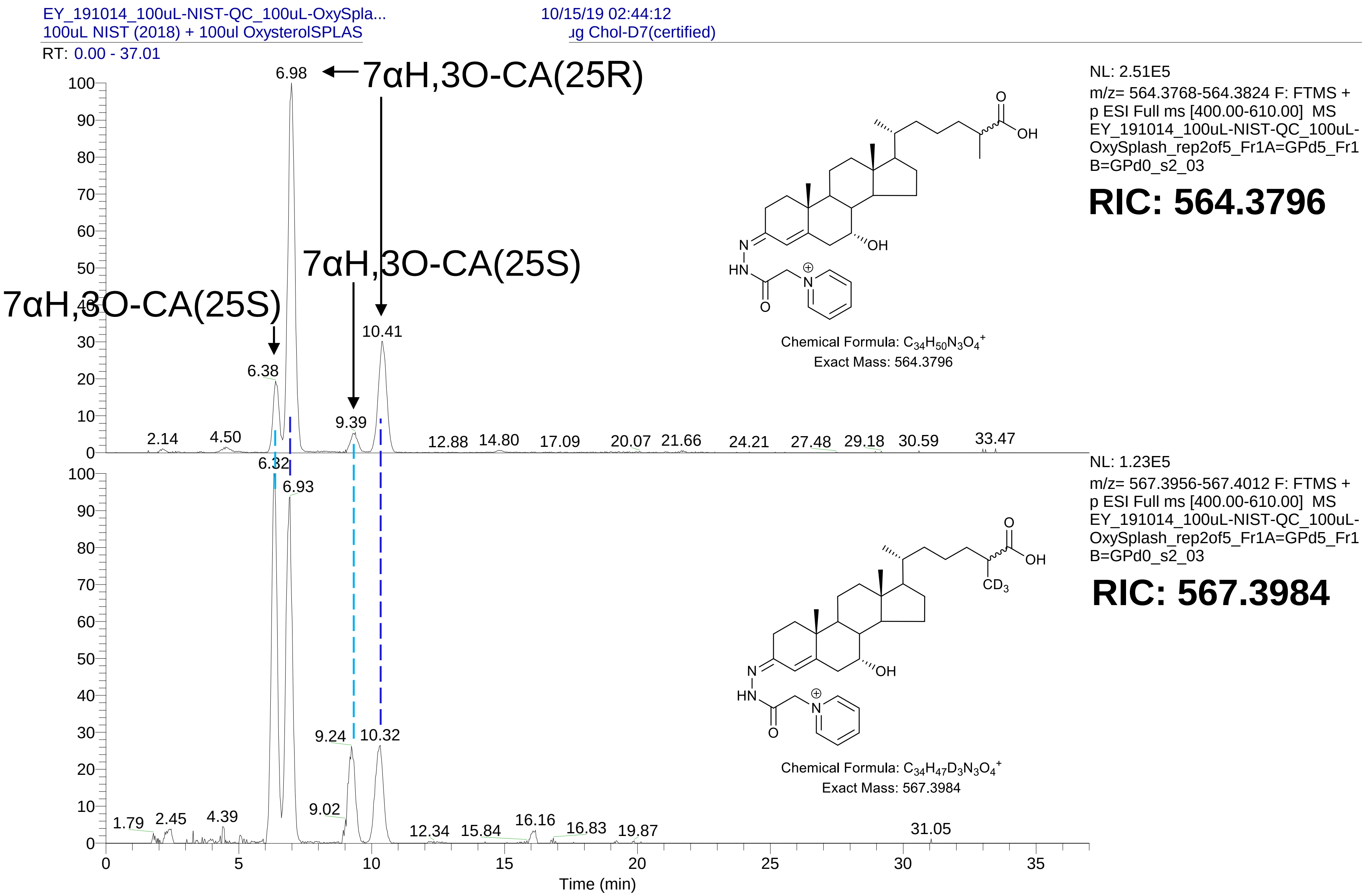
EY_191014_100uL-NIST-QC_100uL-OxySpla...
100uL NIST (2018) + 100ul OxysterolSPLAS
10/15/19 02:44:12
ug Chol-D7(certified)
RT: 0.00 - 37.01
7αH,3O-CA(25R)
7αH,3O-CA(25S)
7αH,3O-CA(25S)
NL: 2.51E5
m/z= 564.3768-564.3824 F: FTMS + p ESI Full ms [400.00-610.00] MS EY_191014_100uL-NIST-QC_100uL-OxySplash_rep2of5_Fr1A=GPd5_Fr1B=GPd0_s2_03
RIC: 564.3796
Chemical Formula: $C_{34}H_{50}N_3O_4^+$
Exact Mass: 564.3796
NL: 1.23E5
m/z= 567.3956-567.4012 F: FTMS + p ESI Full ms [400.00-610.00] MS EY_191014_100uL-NIST-QC_100uL-OxySplash_rep2of5_Fr1A=GPd5_Fr1B=GPd0_s2_03
RIC: 567.3984
Chemical Formula: $C_{34}H_{47}D_3N_3O_4^+$
Exact Mass: 567.3984
Time (min)

S6D

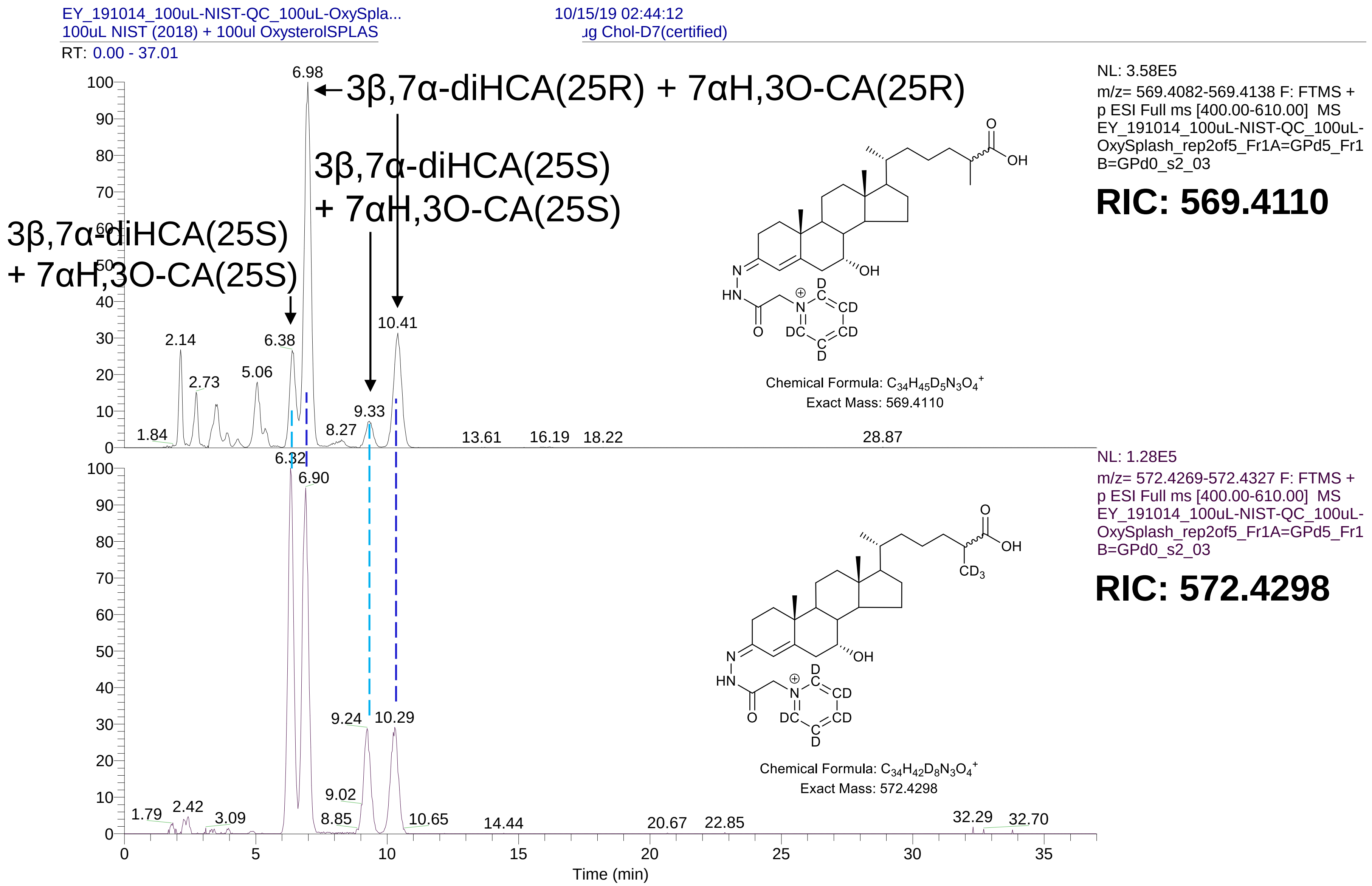
EY_191014_100uL-NIST-QC_100uL-OxySpla...
100uL NIST (2018) + 100ul OxysterolSPLAS
10/15/19 02:44:12
ɹg Chol-D7(certified)
RT: 0.00 - 37.01
NL: 3.58E5
m/z= 569.4082-569.4138 F: FTMS + p ESI Full ms [400.00-610.00] MS EY_191014_100uL-NIST-QC_100uL-OxySplash_rep2of5_Fr1A=GPd5_Fr1B=GPd0_s2_03
RIC: 569.4110
3β,7α-diHCA(25R) + 7αH,3O-CA(25R)
3β,7α-diHCA(25S) + 7αH,3O-CA(25S)
3β,7α-diHCA(25S) + 7αH,3O-CA(25S)
6.98
2.14
2.73
5.06
6.38
10.41
9.33
8.27
1.84
13.61
16.19
18.22
28.87
Chemical Formula: $C_{34}H_{45}D_5N_3O_4^+$
Exact Mass: 569.4110
NL: 1.28E5
m/z= 572.4269-572.4327 F: FTMS + p ESI Full ms [400.00-610.00] MS EY_191014_100uL-NIST-QC_100uL-OxySplash_rep2of5_Fr1A=GPd5_Fr1B=GPd0_s2_03
RIC: 572.4298
6.32
6.90
9.24
10.29
9.02
8.85
10.65
1.79
2.42
3.09
14.44
20.67
22.85
32.29
32.70
Chemical Formula: $C_{34}H_{42}D_8N_3O_4^+$
Exact Mass: 572.4298
Time (min)

S7A

EY_191014_100uL-NIST-QC_100uL-OxySpla... 10/15/19 03:26:25
100uL NIST (2018) + 100ul OxysterolSPLASH + 10ng 22S-HCO-D7 + 20ug

EY_191014_100uL-NIST-QC_100uL-OxySplash_rep2of5_Fr1A=GPd5_Fr1E 5.03 AV: 1 NL: 2.77E3
F: ITMS + c ESI Full ms3 555.43@cid30.00 471.36@cid35.00 [125.00-560.0

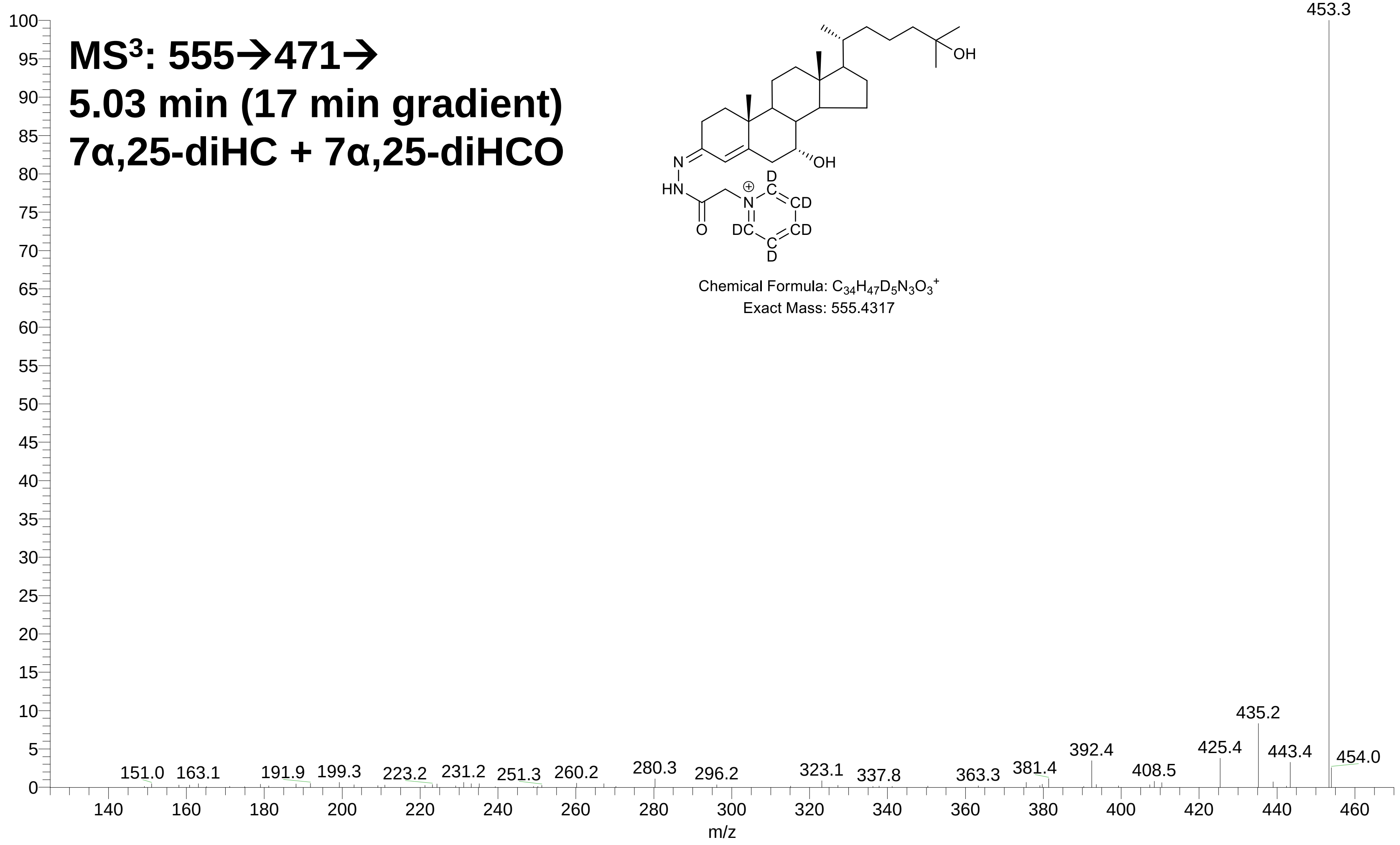

S7B

EY_191014_100uL-NIST-QC_100uL-OxySpla... 10/15/19 03:26:25
100uL NIST (2018) + 100ul OxysterolSPLASH + 10ng 22S-HCO-D7 + 20ug

EY_191014_100uL-NIST-QC_100uL-OxySplash_rep2of5_Fr1A=GPd5_Fr1E 4.97 AV: 1 NL: 1.29E3
F: ITMS + c ESI Full ms3 561.47@cid30.00 477.40@cid35.00 [130.00-570.0

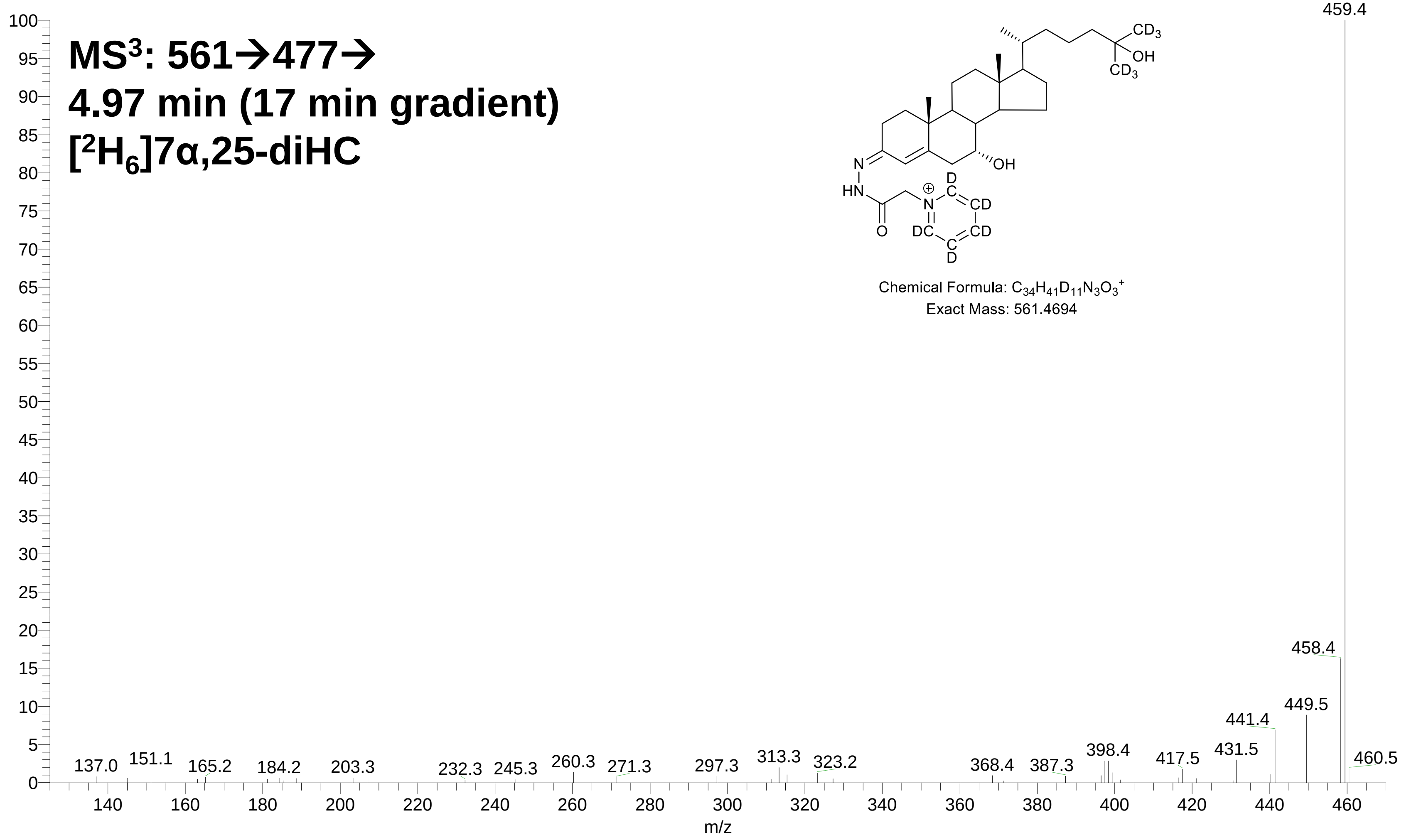

S7C

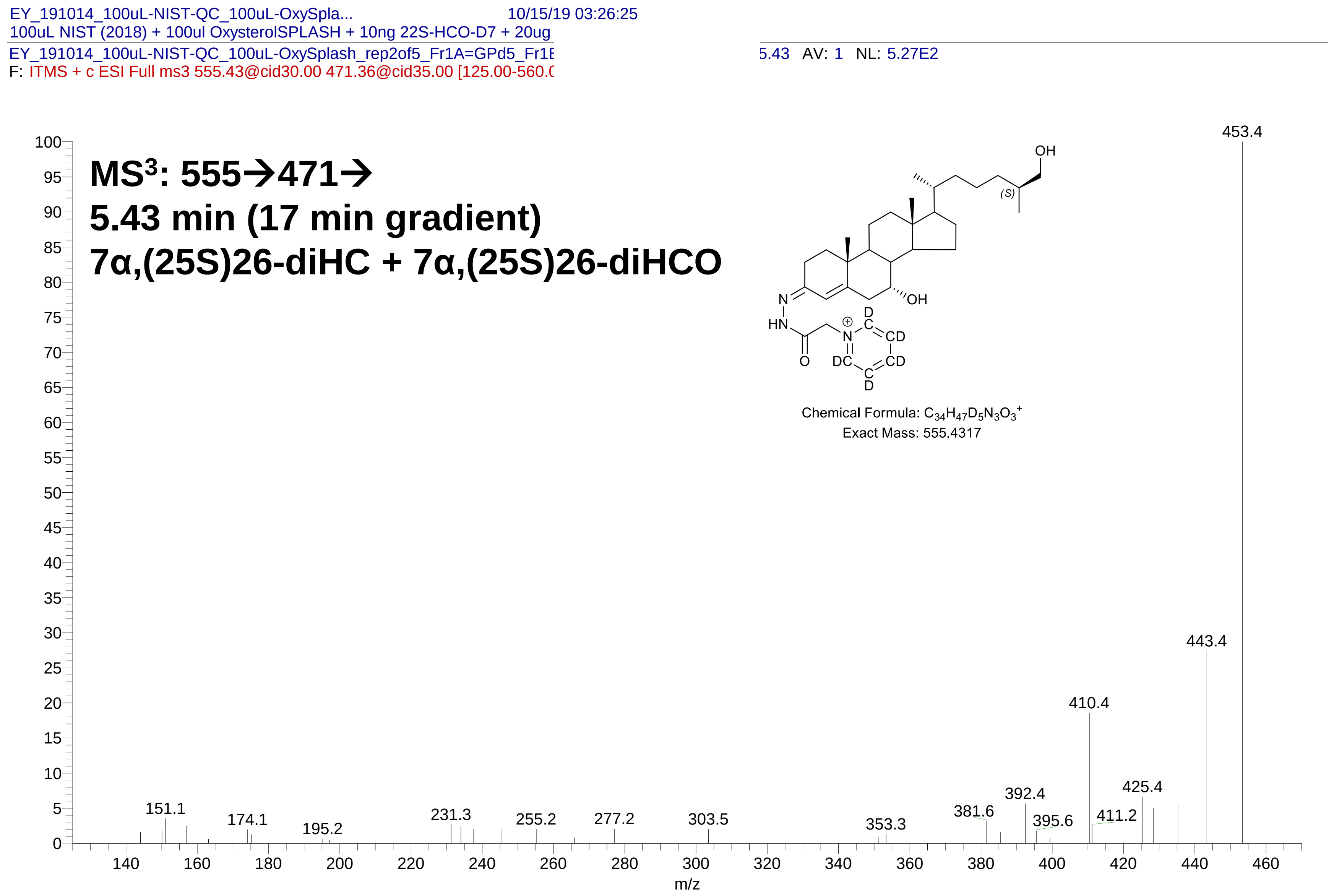

EY_191014_100uL-NIST-QC_100uL-OxySpla...
10/15/19 03:26:25
100uL NIST (2018) + 100ul OxysterolSPLASH + 10ng 22S-HCO-D7 + 20ug
EY_191014_100uL-NIST-QC_100uL-OxySplash_rep2of5_Fr1A=GPd5_Fr1E
F: ITMS + c ESI Full ms3 555.43@cid30.00 471.36@cid35.00 [125.00-560.0
5.43 AV: 1 NL: 5.27E2
MS3: 555→471→
5.43 min (17 min gradient)
7α,(25S)26-diHC + 7α,(25S)26-diHCO
Chemical Formula: C34H47D5N3O3+
Exact Mass: 555.4317
OH
(S)
OH
N
HN
O
N
D
C
CD
CD
DC
C
D
453.4
443.4
410.4
425.4
392.4
381.6
395.6
411.2
353.3
303.5
277.2
255.2
231.3
195.2
174.1
151.1
m/z

S7D

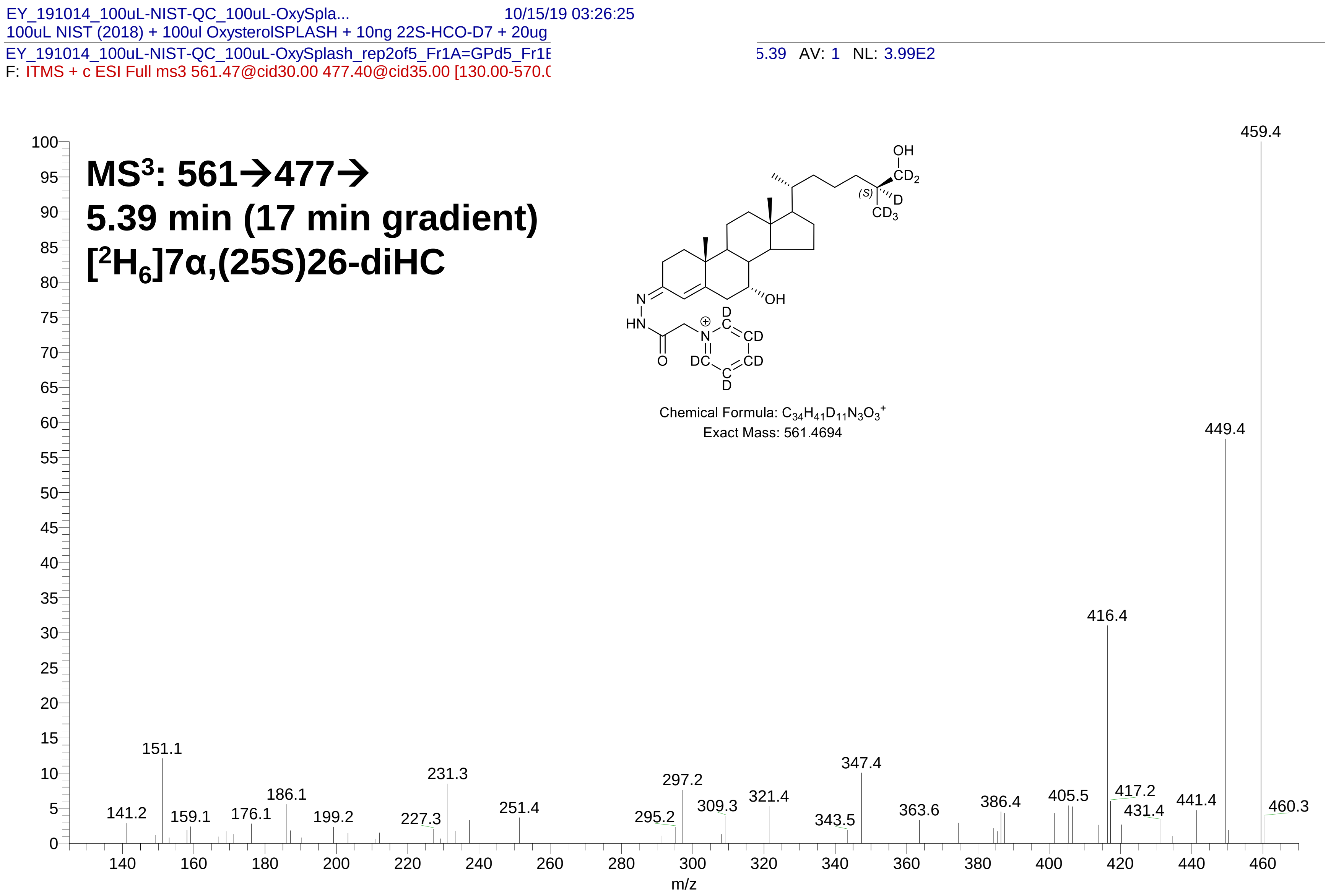
EY_191014_100uL-NIST-QC_100uL-OxySpla...
10/15/19 03:26:25
100uL NIST (2018) + 100ul OxysterolSPLASH + 10ng 22S-HCO-D7 + 20ug
EY_191014_100uL-NIST-QC_100uL-OxySplash_rep2of5_Fr1A=GPd5_Fr1E
5.39 AV: 1 NL: 3.99E2
F: ITMS + c ESI Full ms3 561.47@cid30.00 477.40@cid35.00 [130.00-570.0
MS3: 561→477→
5.39 min (17 min gradient)
[2H6]7α,(25S)26-diHC
Chemical Formula: C34H41D11N3O3+
Exact Mass: 561.4694
459.4
449.4
416.4
151.1
231.3
297.2
347.4
186.1
141.2
159.1
176.1
199.2
227.3
251.4
295.2
309.3
321.4
343.5
363.6
386.4
405.5
417.2
431.4
441.4
460.3
m/z

S7E

EY_191014_100uL-NIST-QC_100uL-OxySpla... 10/15/19 03:26:25
100uL NIST (2018) + 100ul OxysterolSPLASH + 10ng 22S-HCO-D7 + 20ug
EY_191014_100uL-NIST-QC_100uL-OxySplash_rep2of5_Fr1A=GPd5_Fr1E 5.64 AV: 1 NL: 3.64E3
F: ITMS + c ESI Full ms3 555.43@cid30.00 471.36@cid35.00 [125.00-560.0

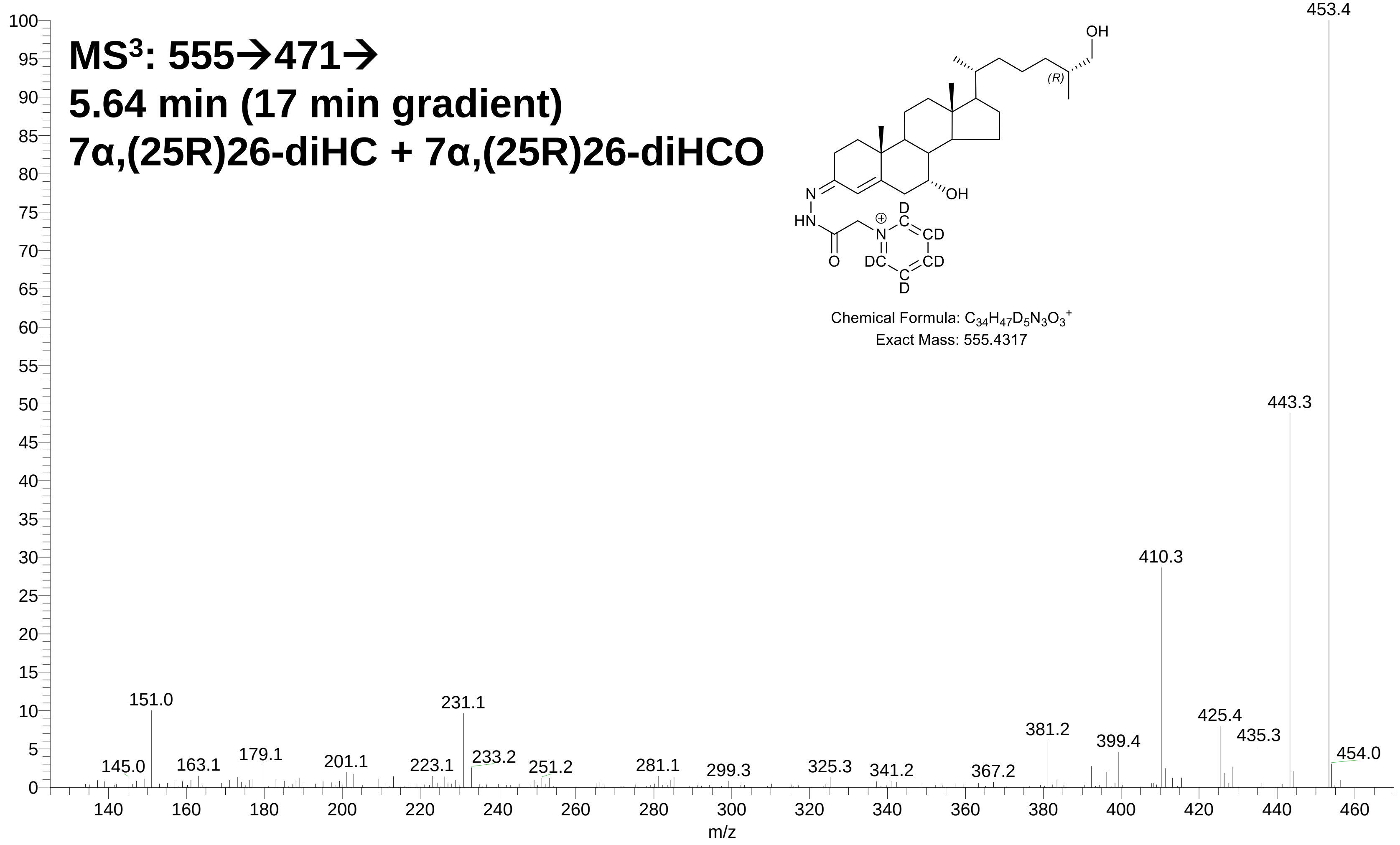

S7F

EY_191014_100uL-NIST-QC_100uL-OxySpla... 10/15/19 03:26:25
100uL NIST (2018) + 100ul OxysterolSPLASH + 10ng 22S-HCO-D7 + 20ug

EY_191014_100uL-NIST-QC_100uL-OxySplash_rep2of5_Fr1A=GPd5_Fr1E 5.59 AV: 1 NL: 4.27E2
F: ITMS + c ESI Full ms3 561.47@cid30.00 477.40@cid35.00 [130.00-570.0

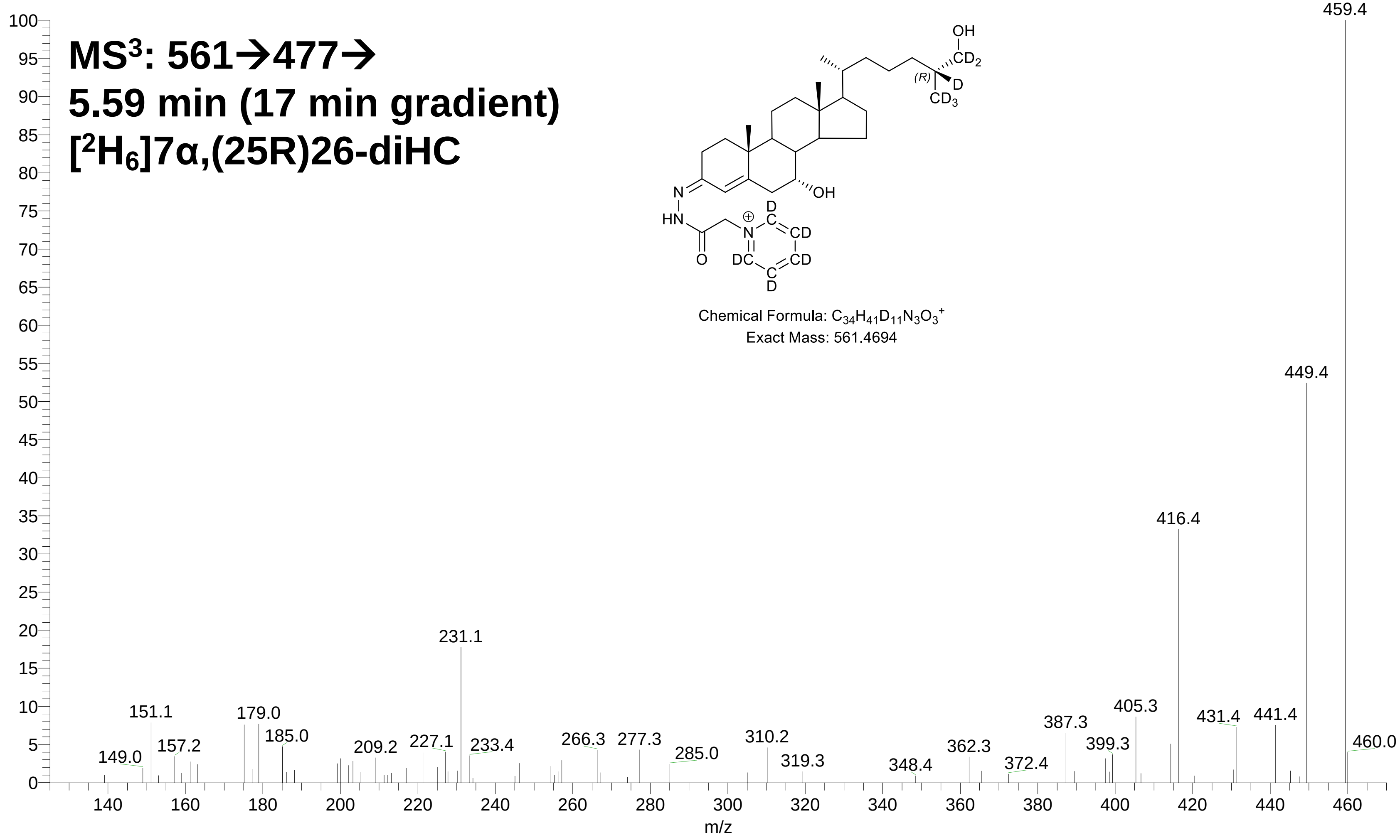

S7G

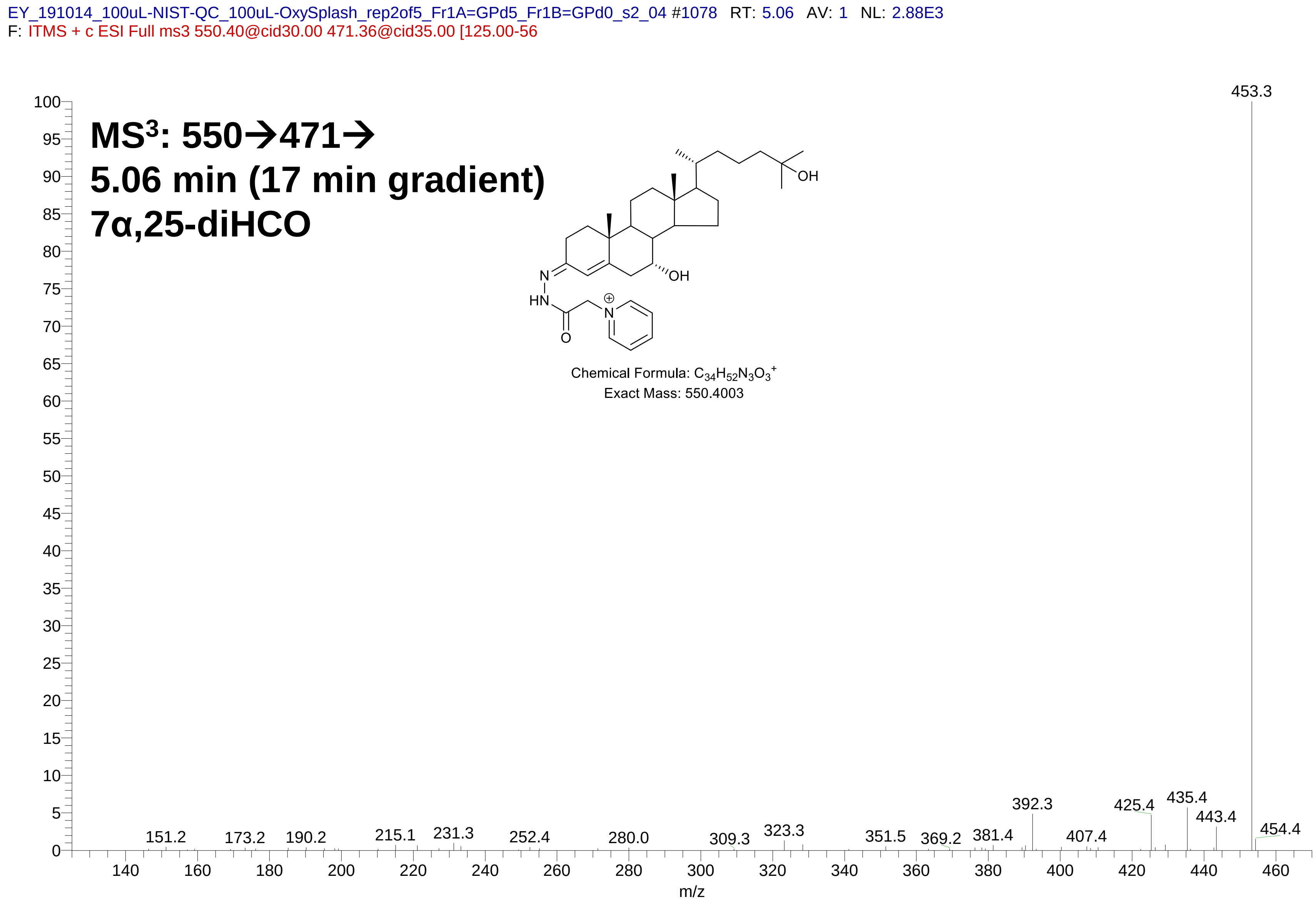
EY_191014_100uL-NIST-QC_100uL-OxySplash_rep2of5_Fr1A=GPd5_Fr1B=GPd0_s2_04 #1078 RT: 5.06 AV: 1 NL: 2.88E3
F: ITMS + c ESI Full ms3 550.40@cid30.00 471.36@cid35.00 [125.00-56
MS3: 550→471→
5.06 min (17 min gradient)
7α,25-diHCO
OH
OH
N
HN
N
O
Chemical Formula: C34H52N3O3+
Exact Mass: 550.4003
453.3
392.3
425.4
435.4
443.4
454.4
151.2
173.2
190.2
215.1
231.3
252.4
280.0
309.3
323.3
351.5
369.2
381.4
407.4
m/z

S7H

EY_191014_100uL-NIST-QC_100uL-OxySplash_rep2of5_Fr1A=GPd5_Fr1B=GPd0_s2_04 #1202 RT: 5.63 AV: 1 NL: 3.56E3
F: ITMS + c ESI Full ms3 550.40@cid30.00 471.36@cid35.00 [125.00-56

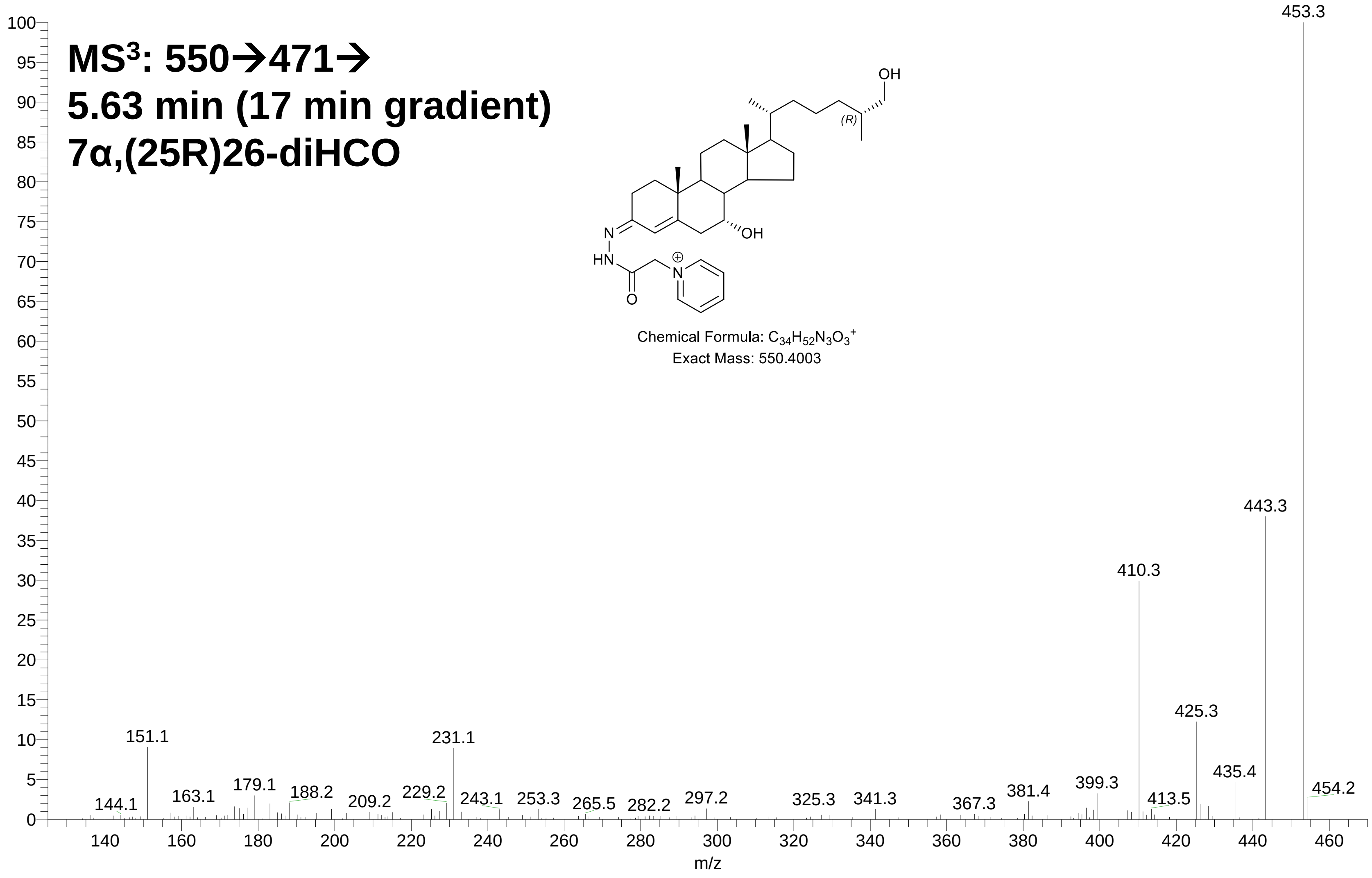

S7I

EY_191014_100uL-NIST-QC_100uL-OxySpla... 10/15/19 03:26:25
100uL NIST (2018) + 100ul OxysterolSPLASH + 10ng 22S-HCO-D7 + 20ug

EY_191014_100uL-NIST-QC_100uL-OxySplash_rep2of5_Fr1A=GPd5_Fr1E 8.41 AV: 1 NL: 2.53E2
F: ITMS + c ESI Full ms3 555.43@cid30.00 471.36@cid35.00 [125.00-560.0

MS³: 555→471→
8.41 min (17 min gradient)
7α,12α-diHC + 7α,12α-diHCO

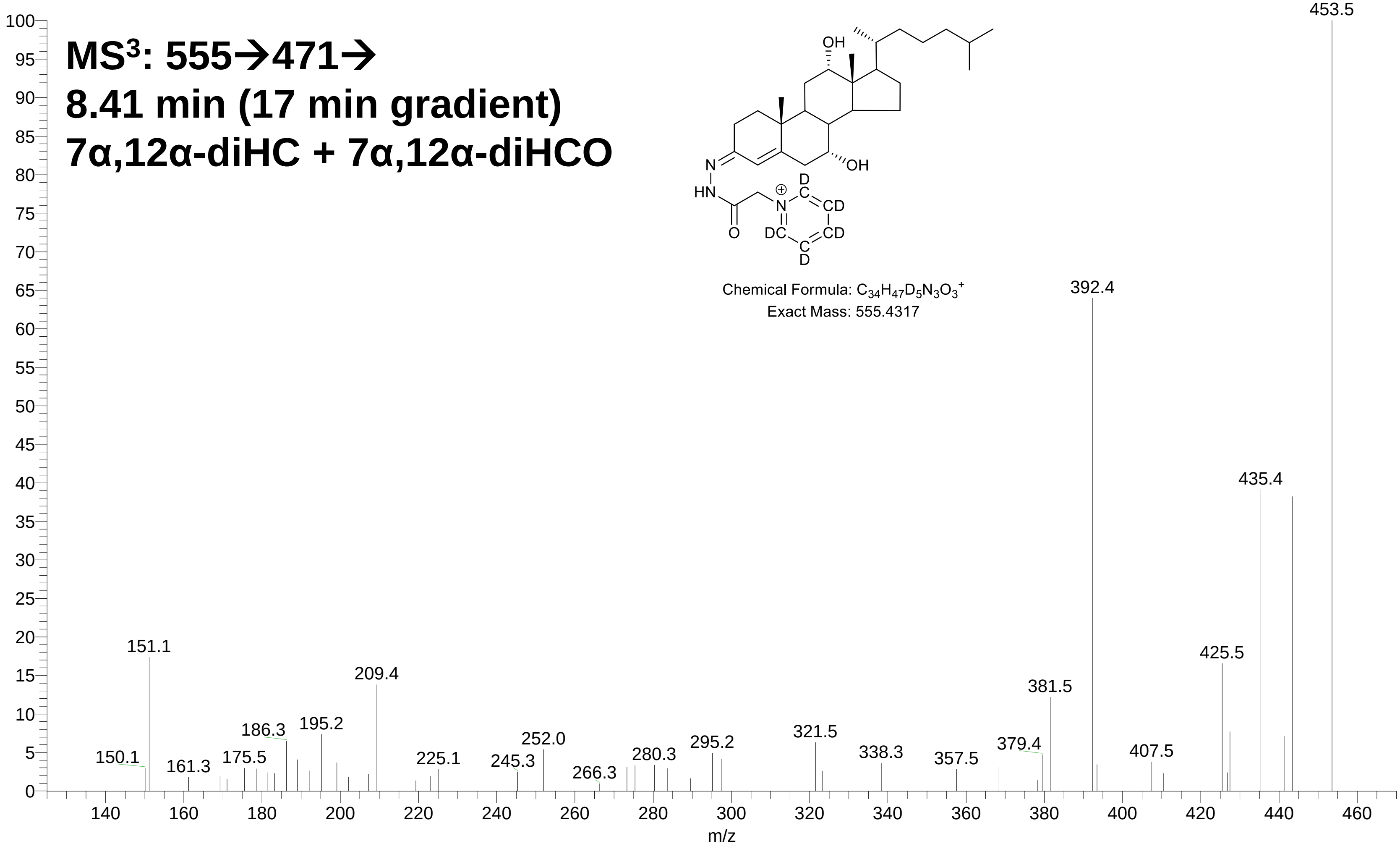

Chemical Formula: $C_{34}H_{47}D_5N_3O_3^+$
Exact Mass: 555.4317

S8A

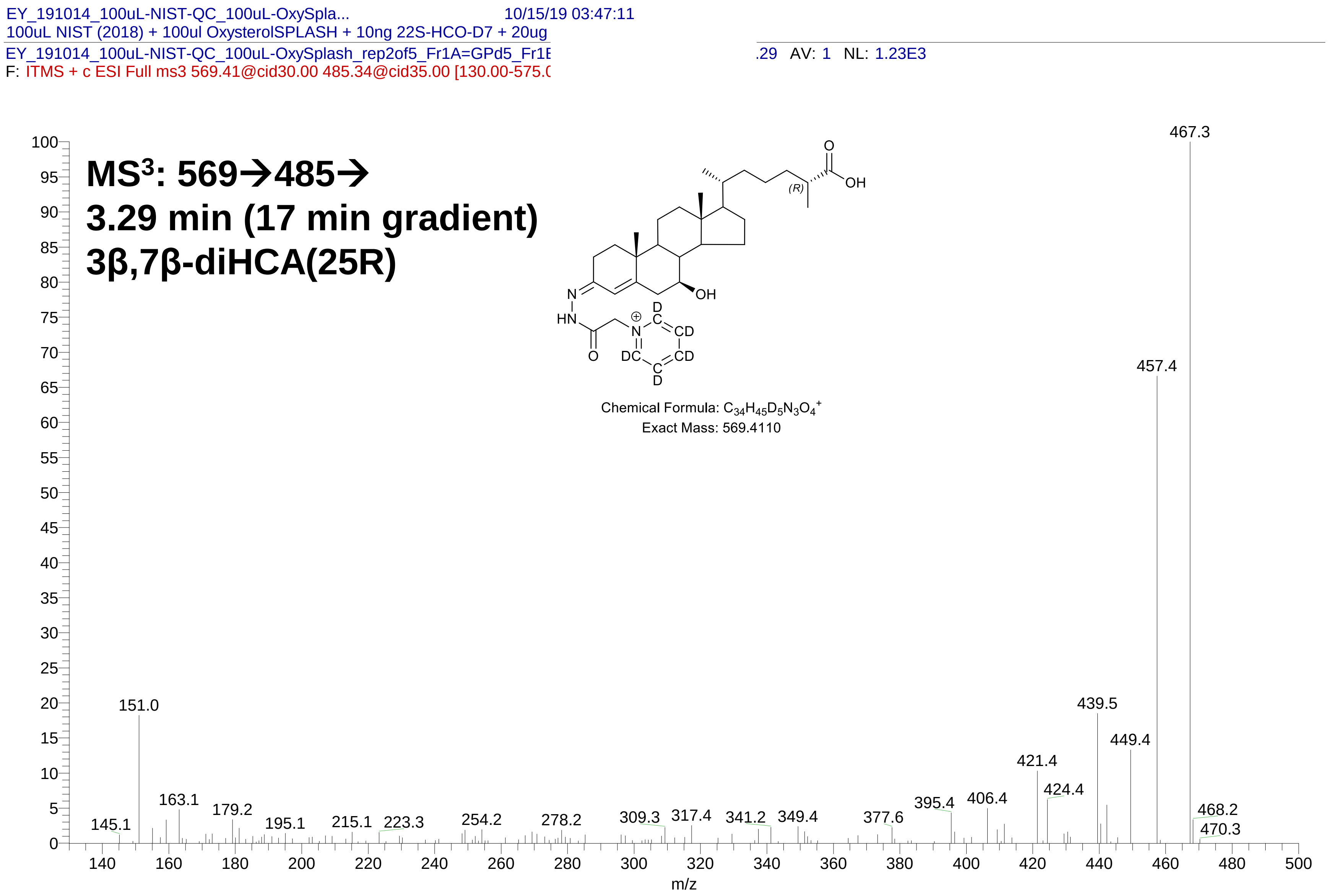
EY_191014_100uL-NIST-QC_100uL-OxySpla... 10/15/19 03:47:11
100uL NIST (2018) + 100ul OxysterolSPLASH + 10ng 22S-HCO-D7 + 20ug
EY_191014_100uL-NIST-QC_100uL-OxySplash_rep2of5_Fr1A=GPd5_Fr1E
.29 AV: 1 NL: 1.23E3
F: ITMS + c ESI Full ms3 569.41@cid30.00 485.34@cid35.00 [130.00-575.0
MS3: 569→485→
3.29 min (17 min gradient)
3β,7β-diHCA(25R)
Chemical Formula: C34H45D5N3O4+
Exact Mass: 569.4110
467.3
457.4
439.5
449.4
421.4
424.4
406.4
395.4
377.6
349.4
341.2
317.4
309.3
278.2
254.2
223.3
215.1
195.1
179.2
163.1
151.0
145.1
468.2
470.3
m/z

S8B

EY_191014_100uL-NIST-QC_100uL-OxySpla... 10/15/19 03:47:11
100uL NIST (2018) + 100ul OxysterolSPLASH + 10ng 22S-HCO-D7 + 20ug

EY_191014_100uL-NIST-QC_100uL-OxySplash_rep2of5_Fr1A=GPd5_Fr1E 5.13 AV: 1 NL: 7.92E3
F: ITMS + c ESI Full ms3 569.41@cid30.00 485.34@cid35.00 [130.00-575.(

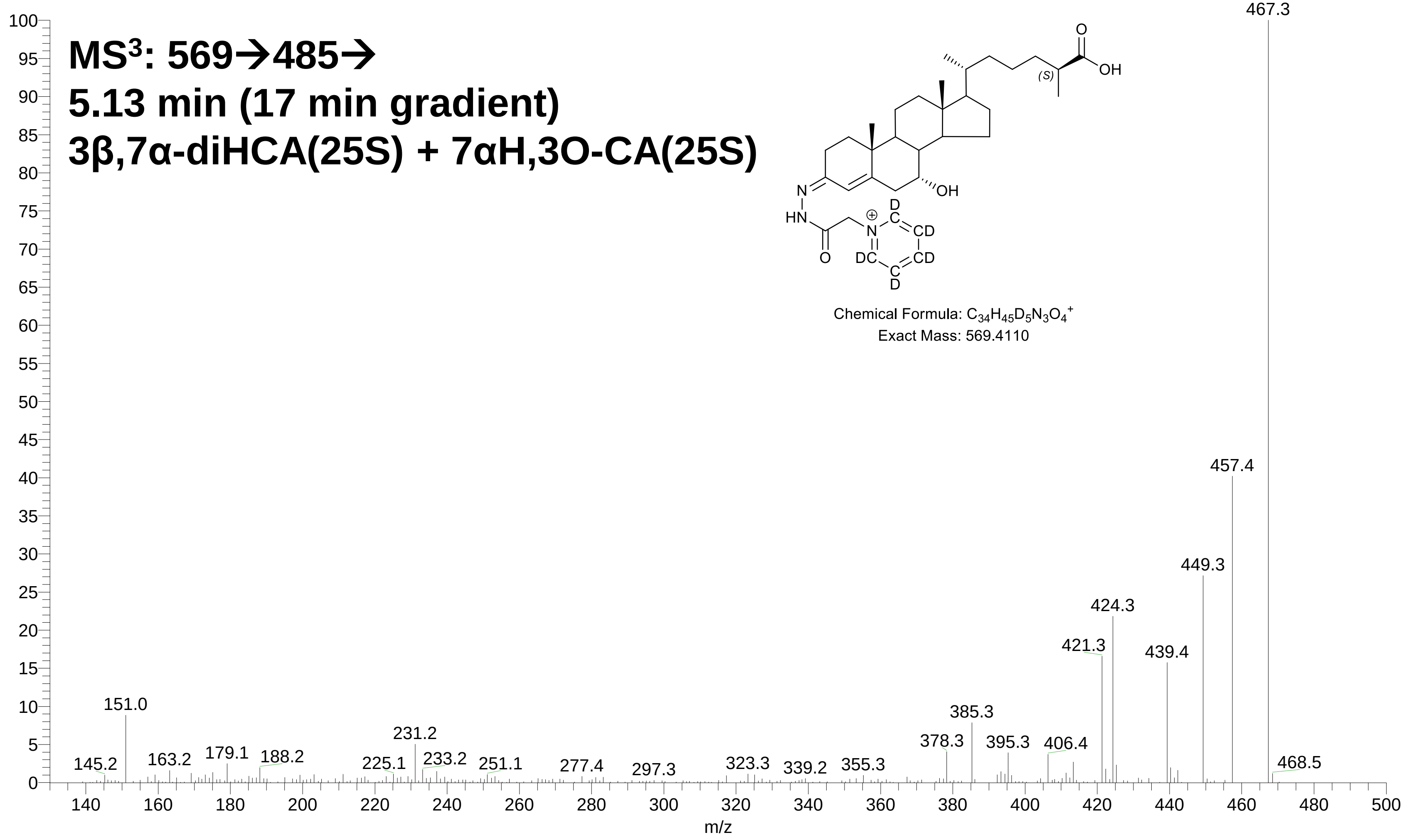

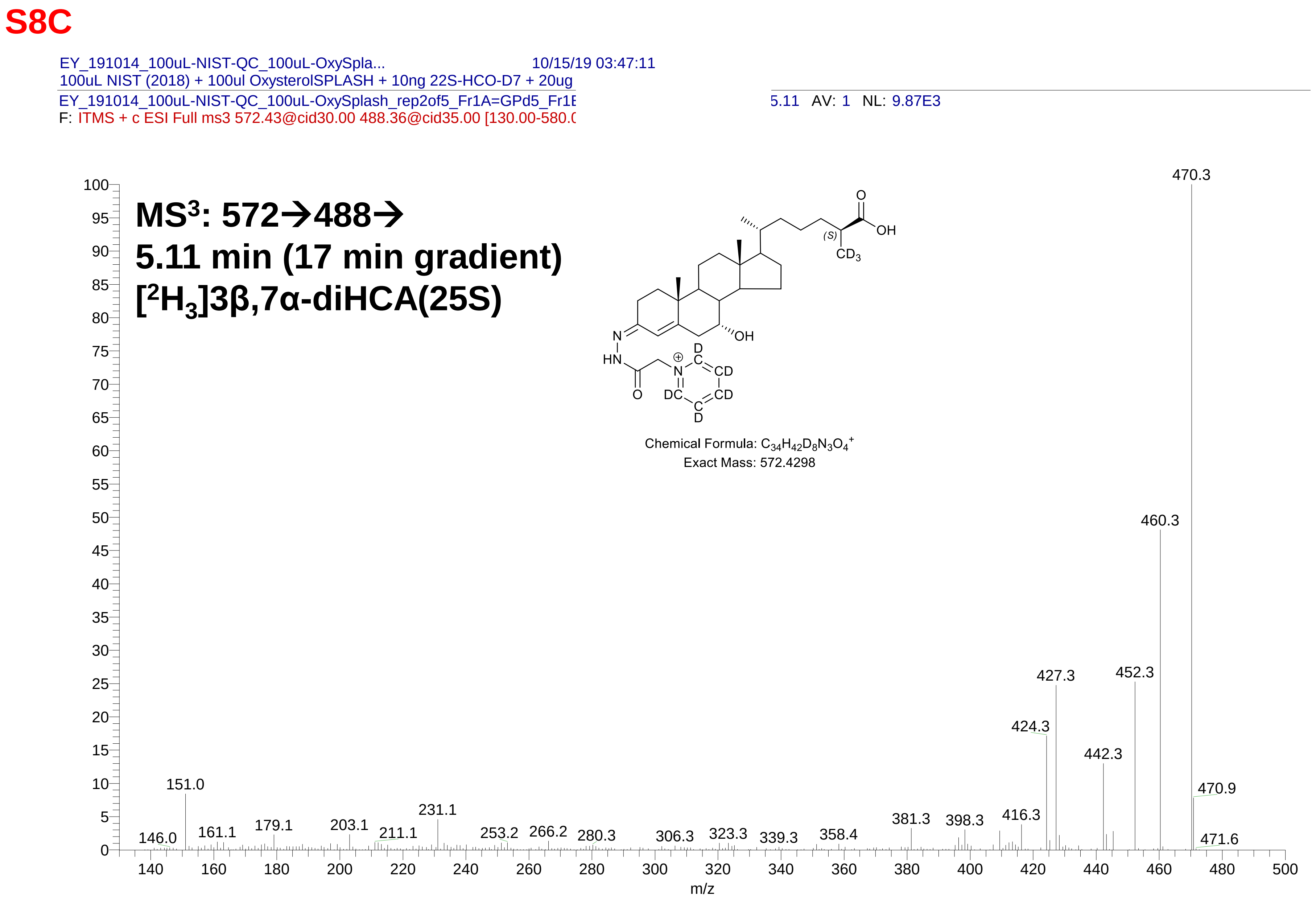
S8C
EY_191014_100uL-NIST-QC_100uL-OxySpla... 10/15/19 03:47:11
100uL NIST (2018) + 100ul OxysterolSPLASH + 10ng 22S-HCO-D7 + 20ug
EY_191014_100uL-NIST-QC_100uL-OxySplash_rep2of5_Fr1A=GPd5_Fr1E
F: ITMS + c ESI Full ms3 572.43@cid30.00 488.36@cid35.00 [130.00-580.0
5.11 AV: 1 NL: 9.87E3
MS3: 572→488→
5.11 min (17 min gradient)
[2H3]3β,7α-diHCA(25S)
Chemical Formula: C34H42D8N3O4+
Exact Mass: 572.4298
470.3
460.3
427.3
452.3
424.3
442.3
470.9
416.3
381.3
398.3
471.6
151.0
146.0
161.1
179.1
203.1
211.1
231.1
253.2
266.2
280.3
306.3
323.3
339.3
358.4
m/z

S8D

EY_191014_100uL-NIST-QC_100uL-OxySpla... 10/15/19 03:47:11
100uL NIST (2018) + 100ul OxysterolSPLASH + 10ng 22S-HCO-D7 + 20ug
EY_191014_100uL-NIST-QC_100uL-OxySplash_rep2of5_Fr1A=GPd5_Fr1E 5.36 AV: 1 NL: 3.45E4
F: ITMS + c ESI Full ms3 569.41@cid30.00 485.34@cid35.00 [130.00-575.0

MS^3: 569→485→
5.36 min (17 min gradient)
3β,7α-diHCA(25R) + 7αH,3O-CA(25R)

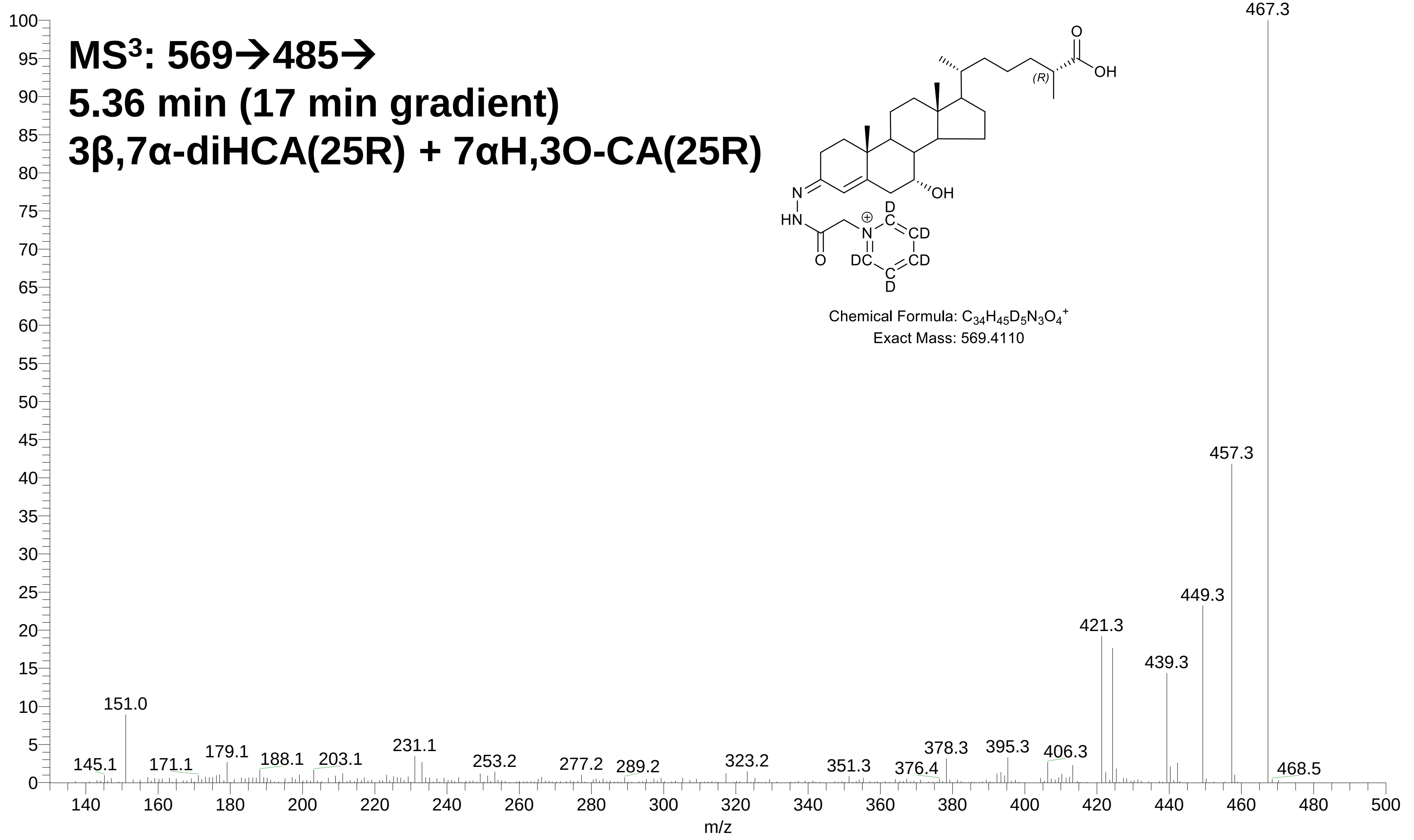

S8E

EY_191014_100uL-NIST-QC_100uL-OxySpla... 10/15/19 03:47:11
100uL NIST (2018) + 100ul OxysterolSPLASH + 10ng 22S-HCO-D7 + 20ug

EY_191014_100uL-NIST-QC_100uL-OxySplash_rep2of5_Fr1A=GPd5_Fr1E 5.30 AV: 1 NL: 1.20E4
F: ITMS + c ESI Full ms3 572.43@cid30.00 488.36@cid35.00 [130.00-580.0

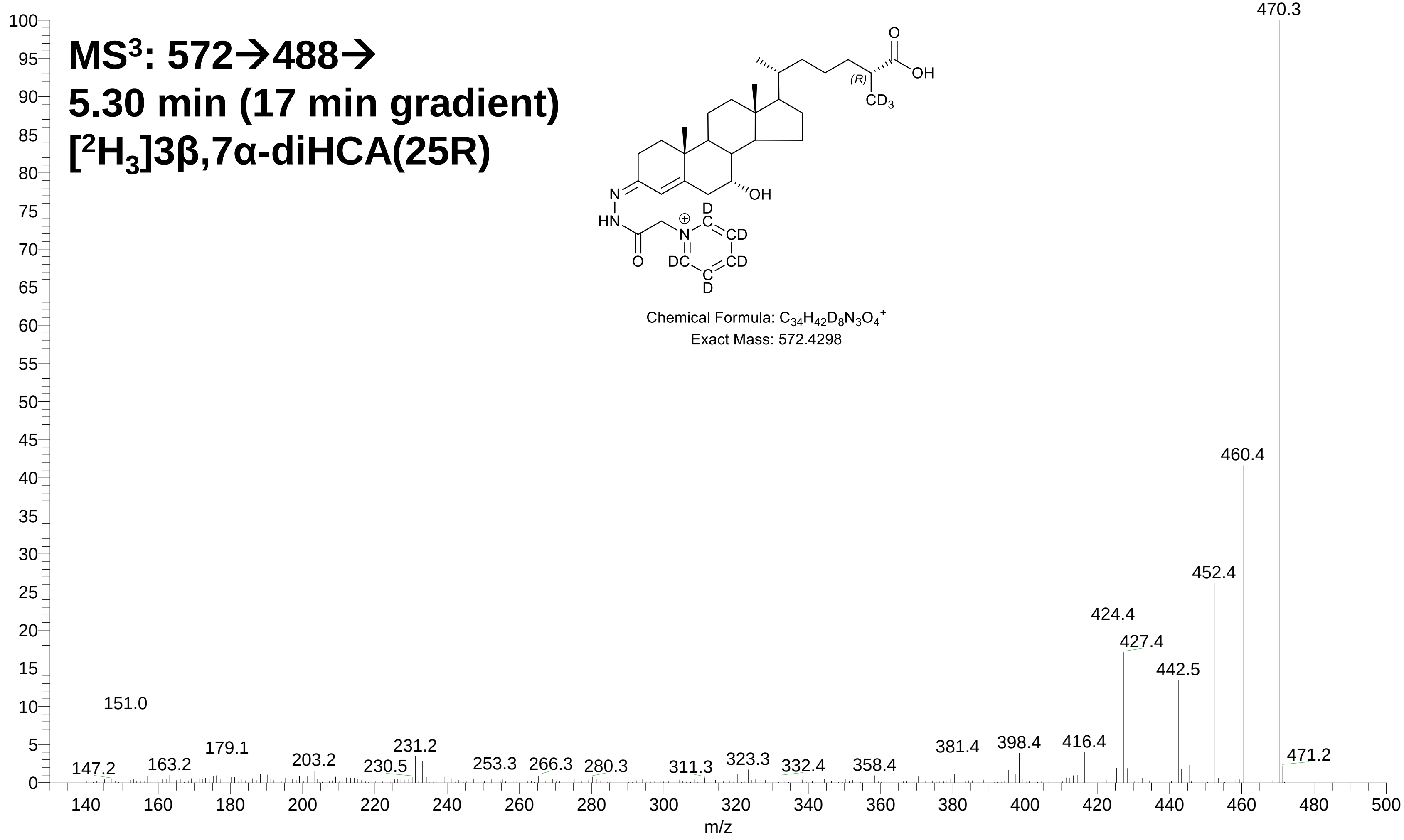

S8F

EY_191014_100uL-NIST-QC_100uL-OxySpla... 10/15/19 03:47:11
100uL NIST (2018) + 100ul OxysterolSPLASH + 10ng 22S-HCO-D7 + 20ug
EY_191014_100uL-NIST-QC_100uL-OxySplash_rep2of5_Fr1A=GPd5_Fr1E 5.12 AV: 1 NL: 4.86E3
F: ITMS + c ESI Full ms3 564.38@cid30.00 485.34@cid35.00 [130.00-575.0

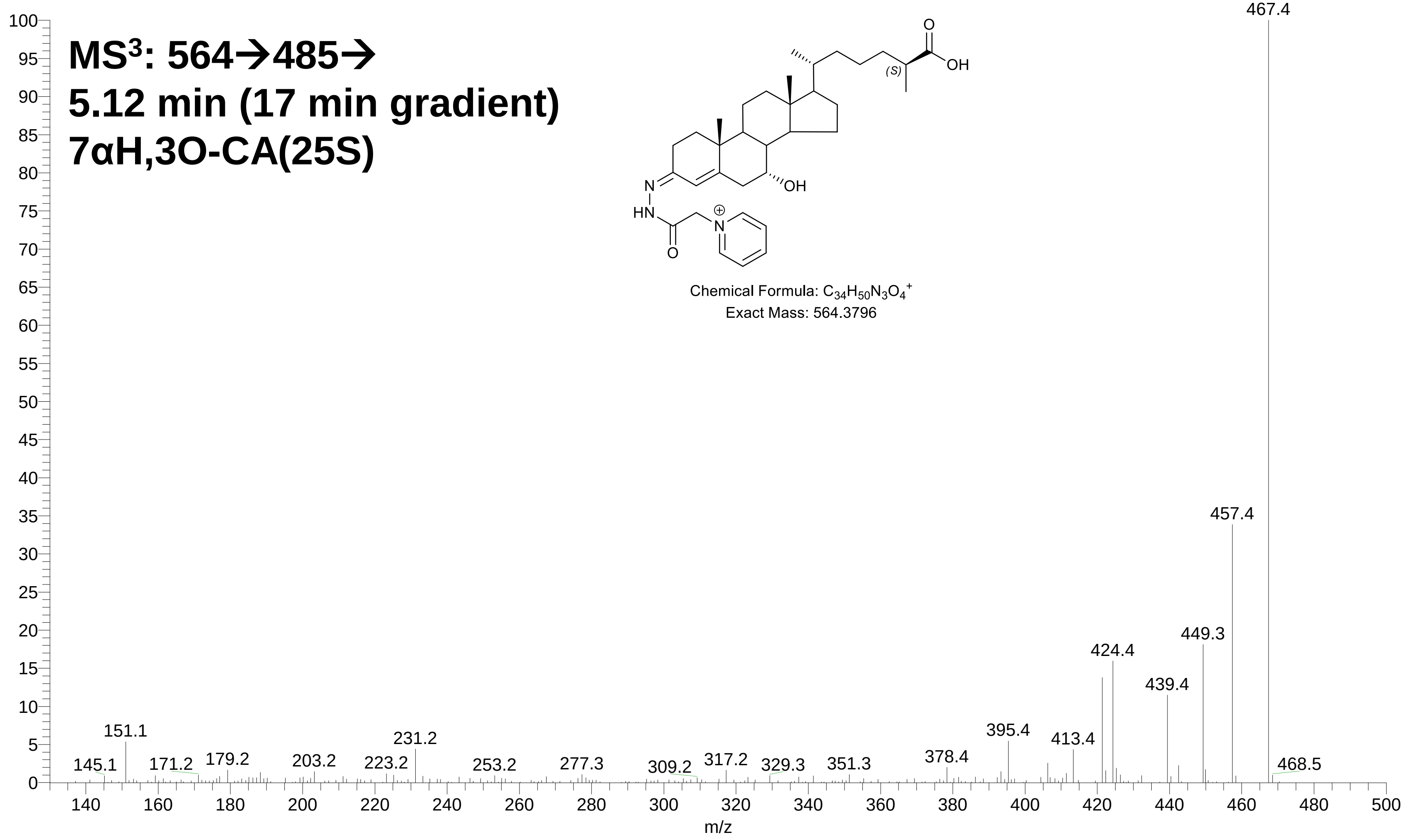

S8G

EY_191014_100uL-NIST-QC_100uL-OxySpla... 10/15/19 03:47:11
100uL NIST (2018) + 100ul OxysterolSPLASH + 10ng 22S-HCO-D7 + 20ug
EY_191014_100uL-NIST-QC_100uL-OxySplash_rep2of5_Fr1A=GPd5_Fr1E 5.35 AV: 1 NL: 2.29E4
F: ITMS + c ESI Full ms3 564.38@cid30.00 485.34@cid35.00 [130.00-575.0

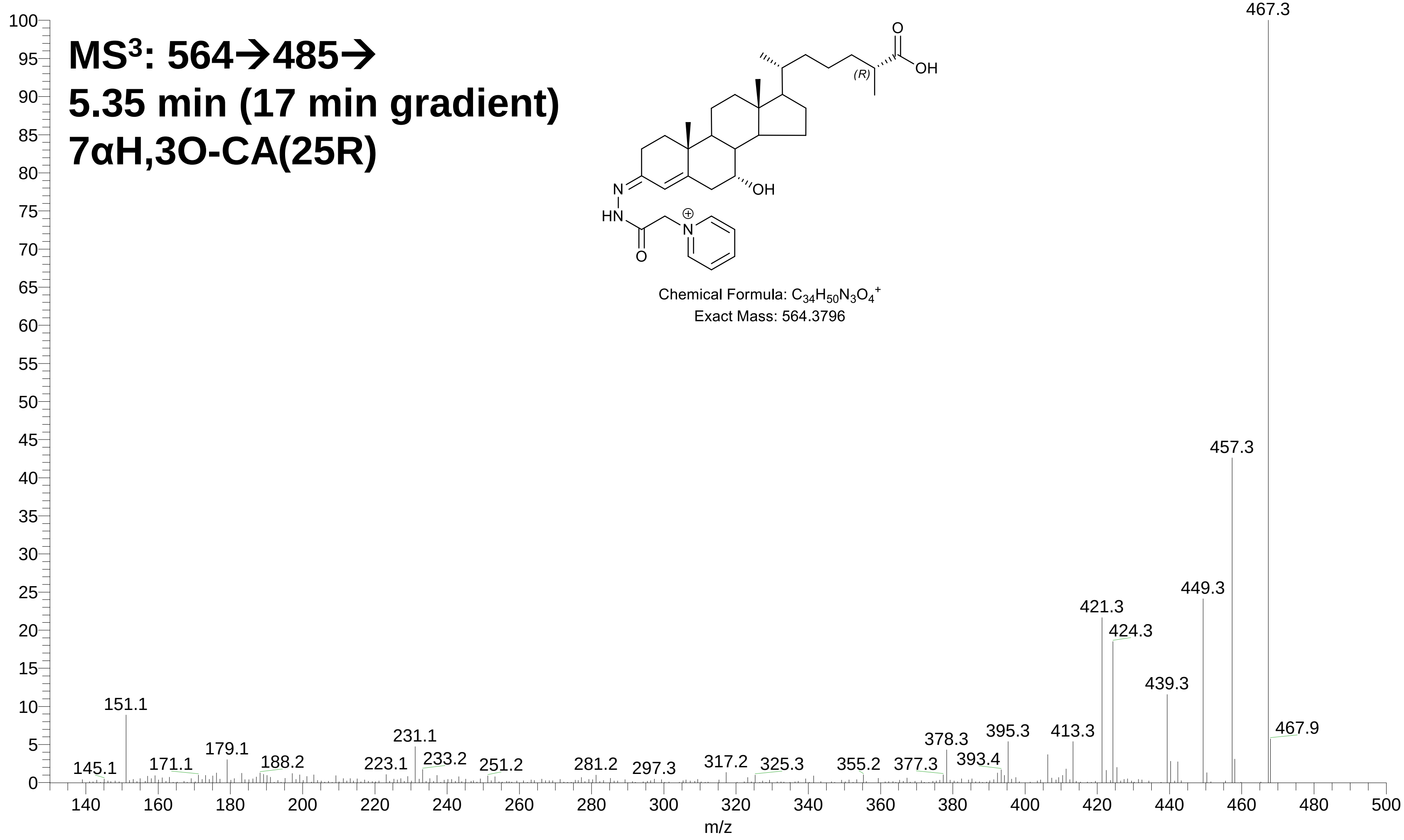

S8H

EY_191014_100uL-NIST-QC_100uL-OxySpla... 10/15/19 03:47:11
100uL NIST (2018) + 100ul OxysterolSPLASH + 10ng 22S-HCO-D7 + 20ug

EY_191014_100uL-NIST-QC_100uL-OxySplash_rep2of5_Fr1A=GPd5_Fr1E 5.30 AV: 1 NL: 1.21E4
F: ITMS + c ESI Full ms3 567.40@cid30.00 488.36@cid35.00 [130.00-580.0

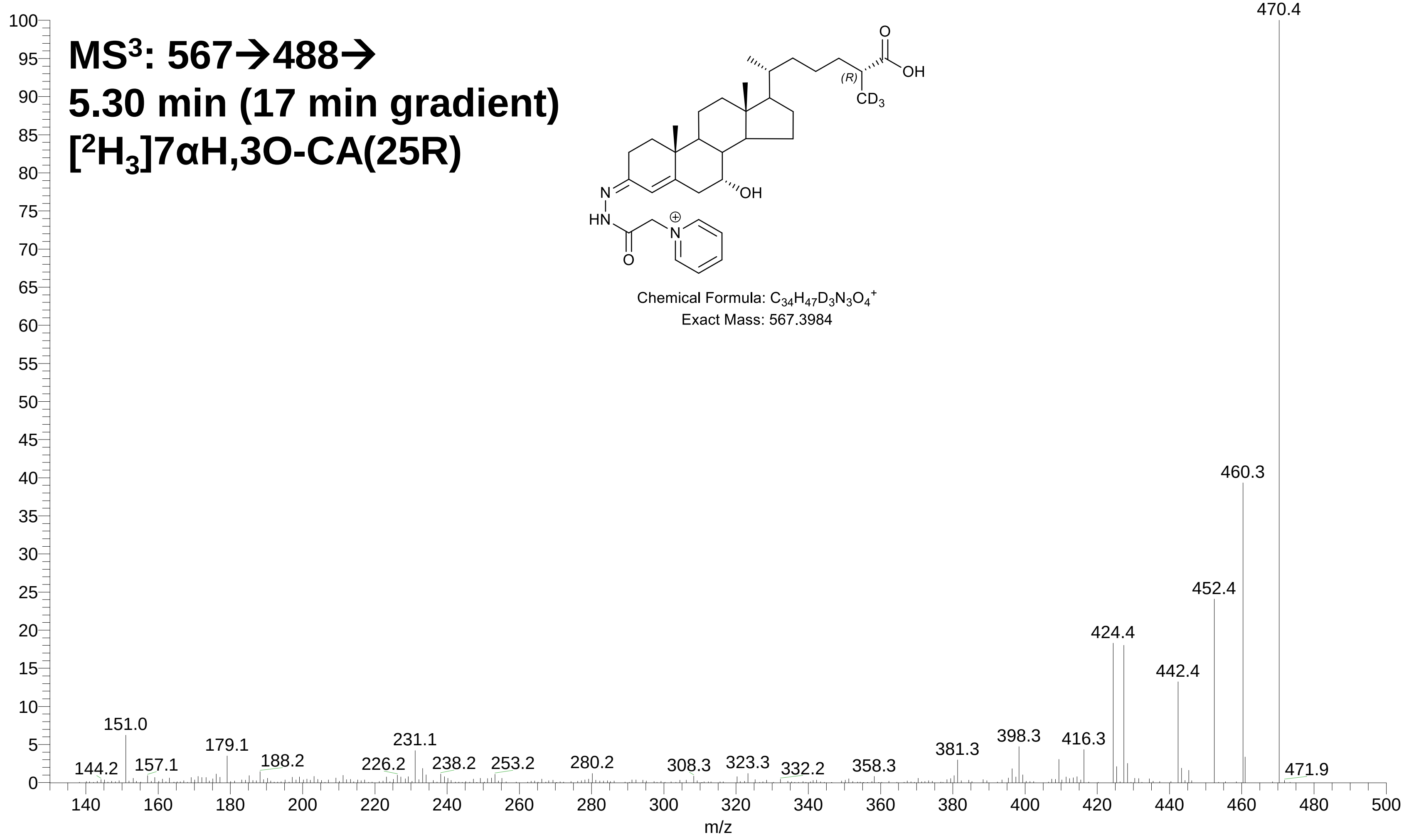

S9A

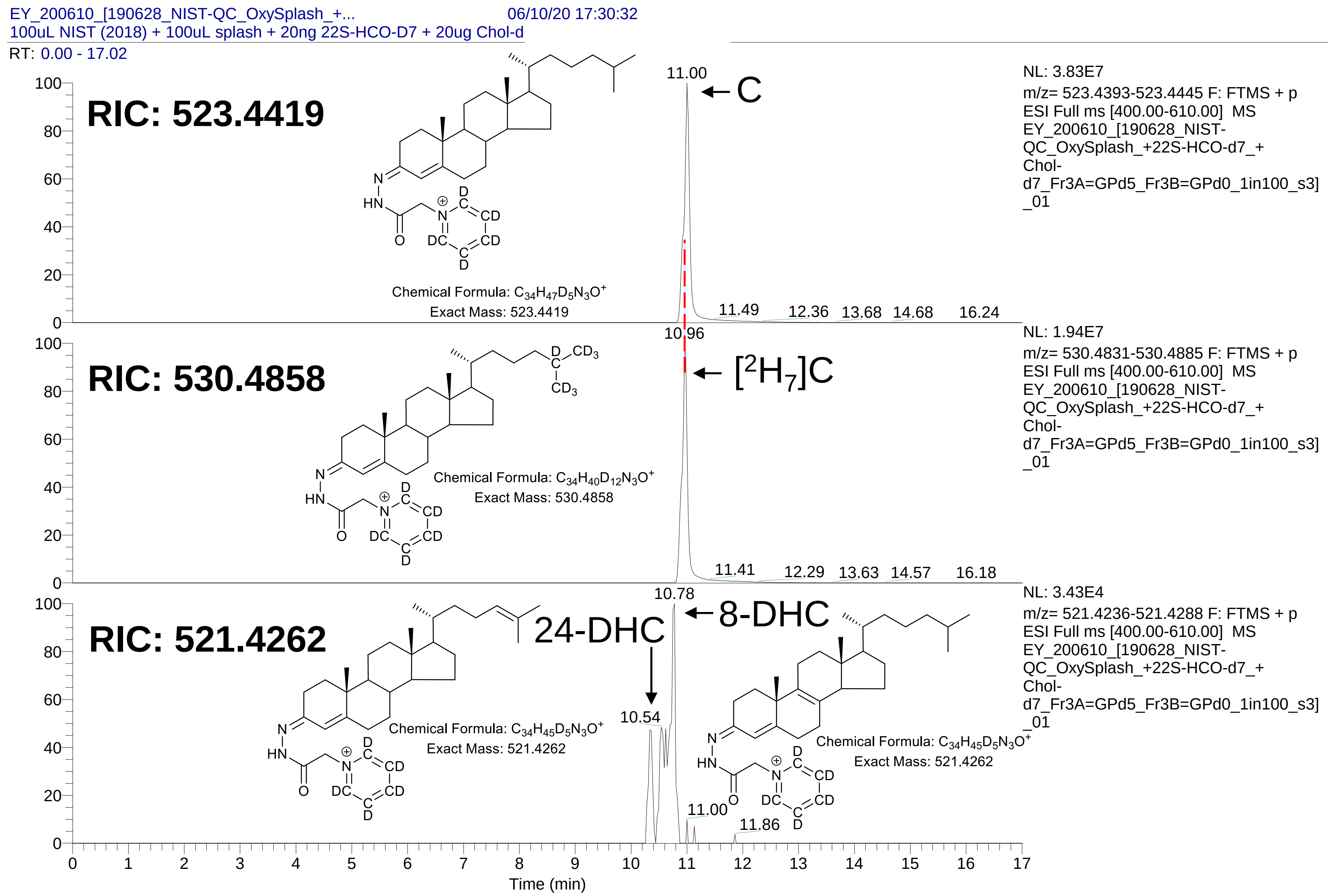

EY_200610_[190628_NIST-QC_OxySplash_+...
06/10/20 17:30:32
100uL NIST (2018) + 100uL splash + 20ng 22S-HCO-D7 + 20ug Chol-d
RT: 0.00 - 17.02
RIC: 523.4419
11.00
C
11.49
12.36
13.68
14.68
16.24
Chemical Formula: C34H47D5N3O+
Exact Mass: 523.4419
NL: 3.83E7
m/z= 523.4393-523.4445 F: FTMS + p ESI Full ms [400.00-610.00] MS EY_200610_[190628_NIST-QC_OxySplash_+22S-HCO-d7_+Chol-d7_Fr3A=GPd5_Fr3B=GPd0_1in100_s3]_01
RIC: 530.4858
10.96
[2H7]C
11.41
12.29
13.63
14.57
16.18
Chemical Formula: C34H40D12N3O+
Exact Mass: 530.4858
NL: 1.94E7
m/z= 530.4831-530.4885 F: FTMS + p ESI Full ms [400.00-610.00] MS EY_200610_[190628_NIST-QC_OxySplash_+22S-HCO-d7_+Chol-d7_Fr3A=GPd5_Fr3B=GPd0_1in100_s3]_01
RIC: 521.4262
10.78
24-DHC
8-DHC
10.54
11.00
11.86
Chemical Formula: C34H45D5N3O+
Exact Mass: 521.4262
Chemical Formula: C34H45D5N3O+
Exact Mass: 521.4262
NL: 3.43E4
m/z= 521.4236-521.4288 F: FTMS + p ESI Full ms [400.00-610.00] MS EY_200610_[190628_NIST-QC_OxySplash_+22S-HCO-d7_+Chol-d7_Fr3A=GPd5_Fr3B=GPd0_1in100_s3]_01
Time (min)

S9B

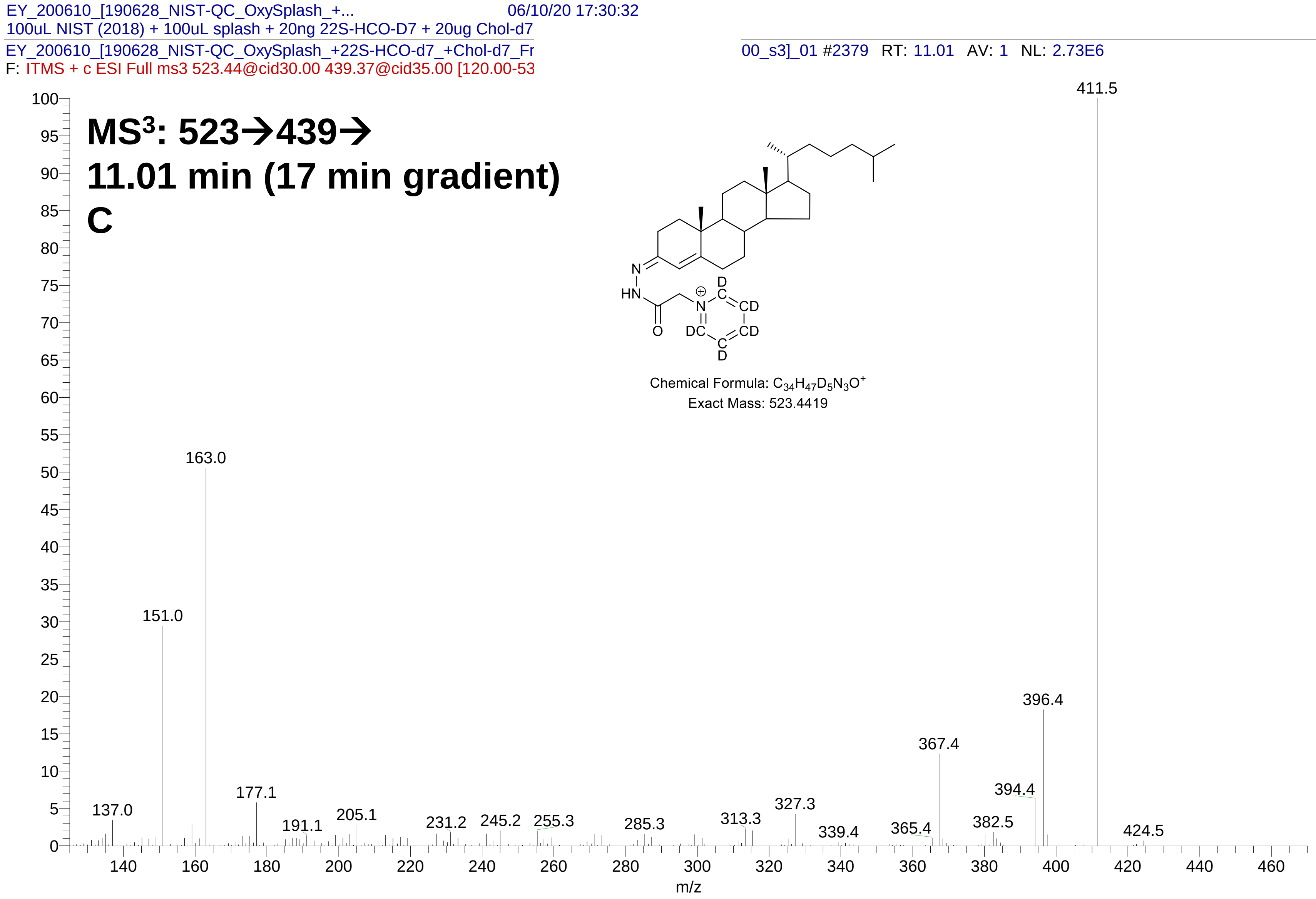
EY_200610_[190628_NIST-QC_OxySplash_+...
06/10/20 17:30:32
100uL NIST (2018) + 100uL splash + 20ng 22S-HCO-D7 + 20ug Chol-d7
EY_200610_[190628_NIST-QC_OxySplash_+22S-HCO-d7_+Chol-d7_Fr
00_s3]_01 #2379 RT: 11.01 AV: 1 NL: 2.73E6
F: ITMS + c ESI Full ms3 523.44@cid30.00 439.37@cid35.00 [120.00-53
MS³: 523→439→
11.01 min (17 min gradient)
C
Chemical Formula: C34H47D5N3O+
Exact Mass: 523.4419
411.5
163.0
151.0
396.4
367.4
394.4
177.1
137.0
191.1
205.1
231.2
245.2
255.3
285.3
313.3
327.3
339.4
365.4
382.5
424.5
m/z

S9C

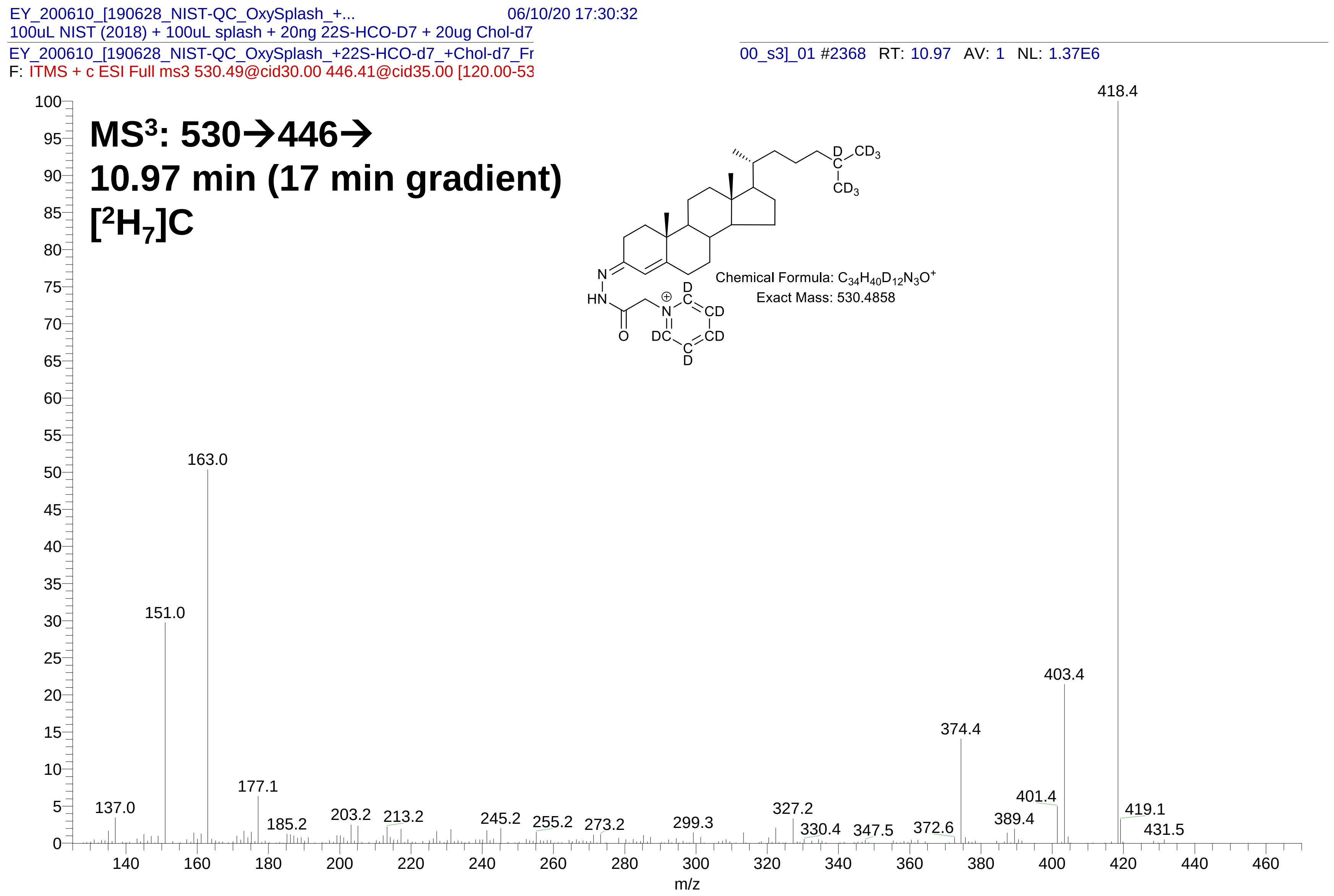

EY_200610_[190628_NIST-QC_OxySplash_+...
06/10/20 17:30:32
100uL NIST (2018) + 100uL splash + 20ng 22S-HCO-D7 + 20ug Chol-d7
EY_200610_[190628_NIST-QC_OxySplash_+22S-HCO-d7_+Chol-d7_Fr
00_s3]_01 #2368 RT: 10.97 AV: 1 NL: 1.37E6
F: ITMS + c ESI Full ms3 530.49@cid30.00 446.41@cid35.00 [120.00-53
MS3: 530→446→
10.97 min (17 min gradient)
[2H7]C
Chemical Formula: C34H40D12N3O+
Exact Mass: 530.4858
418.4
403.4
374.4
163.0
151.0
177.1
137.0
185.2
203.2
213.2
245.2
255.2
273.2
299.3
327.2
330.4
347.5
372.6
389.4
401.4
419.1
431.5
m/z

S9D

EY_200610_[190628_NIST-QC_OxySplash_+... 06/10/20 17:50:25
100uL NIST (2018) + 100uL splash + 20ng 22S-HCO-D7 + 20ug Chol-d7 (c
EY_200610_[190628_NIST-QC_OxySplash_+22S-HCO-d7_+Chol-d7_Fr3A _s3]_02 #2232 RT: 10.36 AV: 1 NL: 1.14E3
F: ITMS + c ESI Full ms3 521.43@cid30.00 437.35@cid35.00 [120.00-530.0

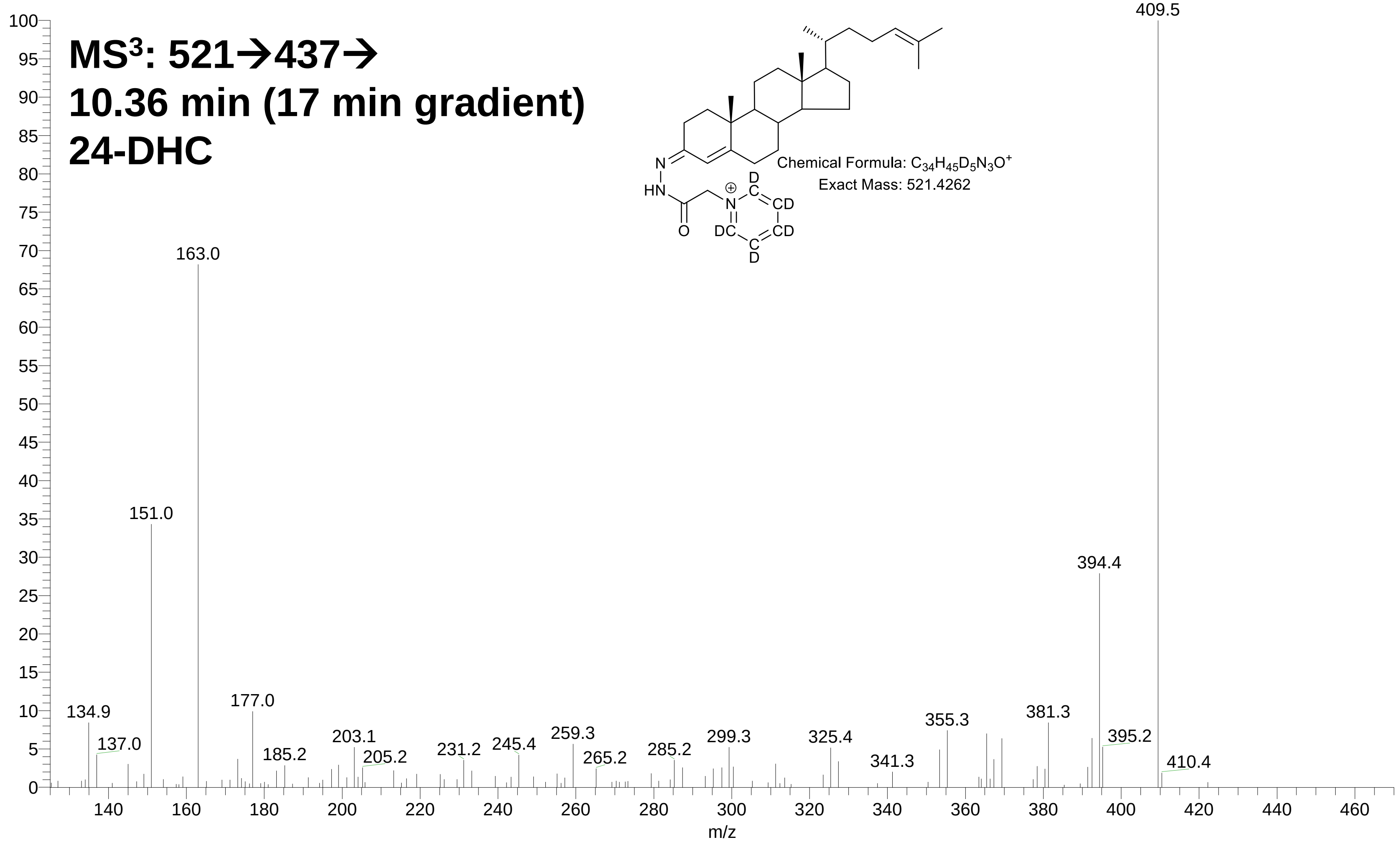

S9E

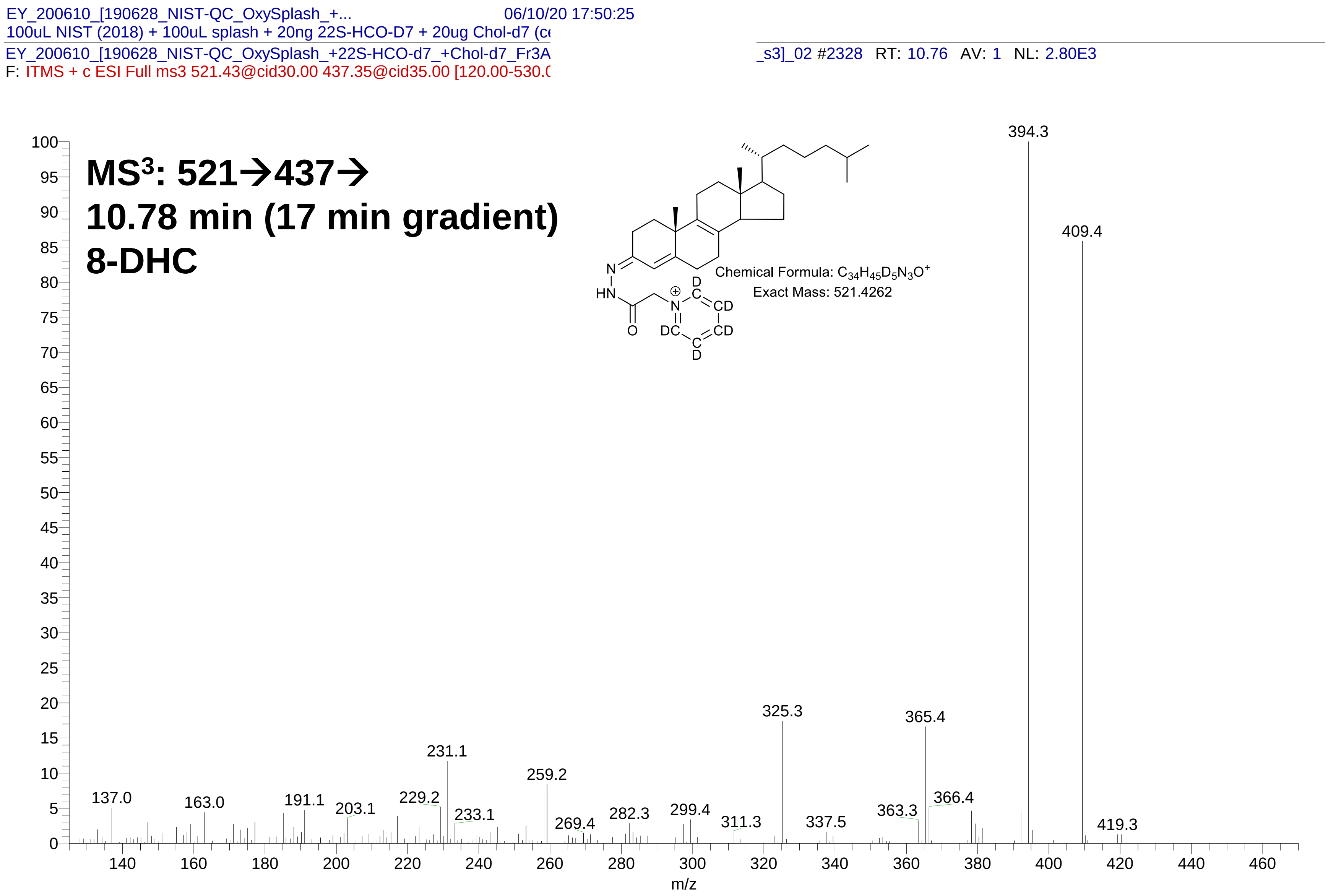
EY_200610_[190628_NIST-QC_OxySplash_+... 06/10/20 17:50:25
100uL NIST (2018) + 100uL splash + 20ng 22S-HCO-D7 + 20ug Chol-d7 (c
EY_200610_[190628_NIST-QC_OxySplash_+22S-HCO-d7_+Chol-d7_Fr3A _s3]_02 #2328 RT: 10.76 AV: 1 NL: 2.80E3
F: ITMS + c ESI Full ms3 521.43@cid30.00 437.35@cid35.00 [120.00-530.0
MS^3: 521→437→
10.78 min (17 min gradient)
8-DHC
Chemical Formula: $C_{34}H_{45}D_5N_3O^+$
Exact Mass: 521.4262
394.3
409.4
325.3
365.4
231.1
259.2
137.0
163.0
191.1
203.1
229.2
233.1
269.4
282.3
299.4
311.3
337.5
363.3
366.4
419.3
m/z

S9F

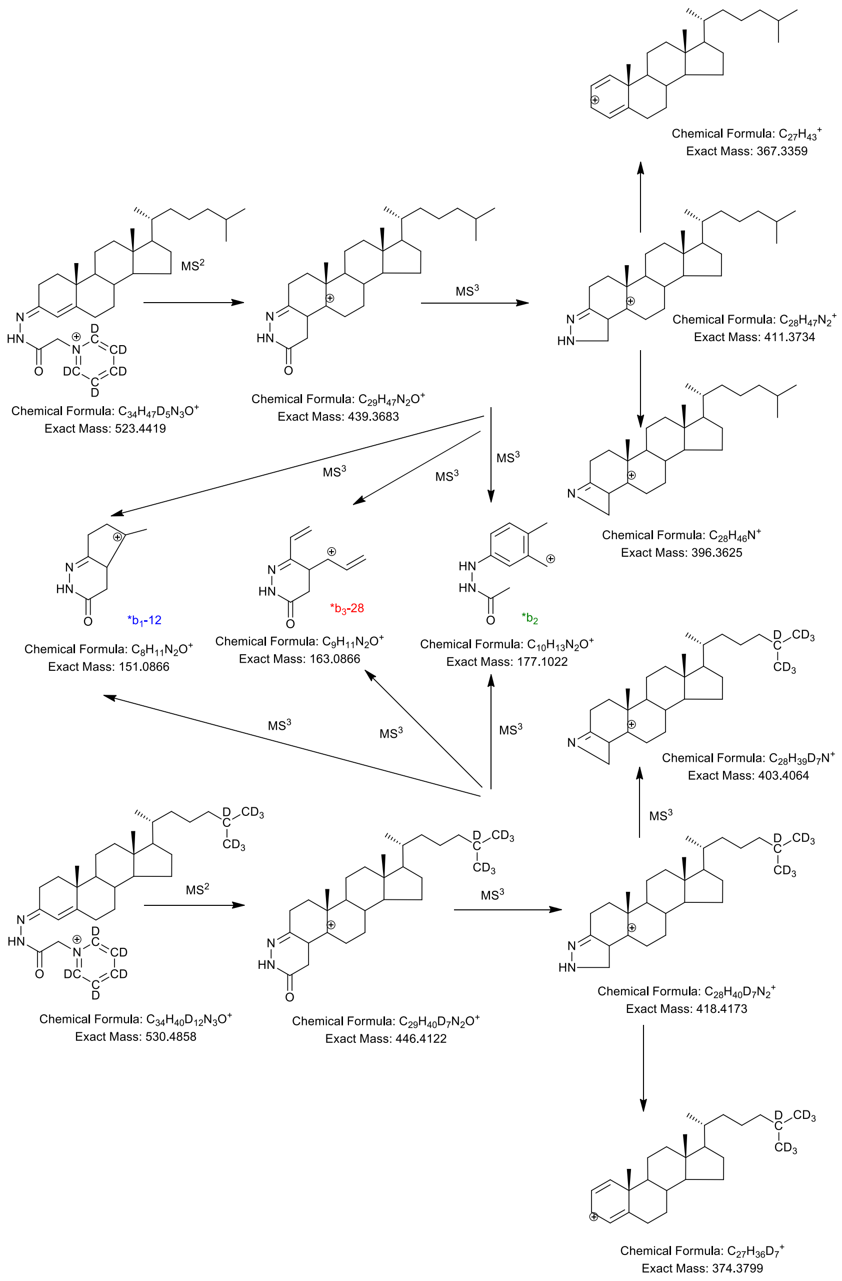

MS2
Chemical Formula: C34H47D5N3O+
Exact Mass: 523.4419
Chemical Formula: C29H47N2O+
Exact Mass: 439.3683
MS3
Chemical Formula: C28H47N2+
Exact Mass: 411.3734
Chemical Formula: C27H43+
Exact Mass: 367.3359
Chemical Formula: C28H46N+
Exact Mass: 396.3625
*b1-12
Chemical Formula: C8H11N2O+
Exact Mass: 151.0866
*b3-28
Chemical Formula: C9H11N2O+
Exact Mass: 163.0866
*b2
Chemical Formula: C10H13N2O+
Exact Mass: 177.1022
Chemical Formula: C28H39D7N+
Exact Mass: 403.4064
Chemical Formula: C34H40D12N3O+
Exact Mass: 530.4858
Chemical Formula: C29H40D7N2O+
Exact Mass: 446.4122
Chemical Formula: C28H40D7N2+
Exact Mass: 418.4173
Chemical Formula: C27H36D7+
Exact Mass: 374.3799

S9G

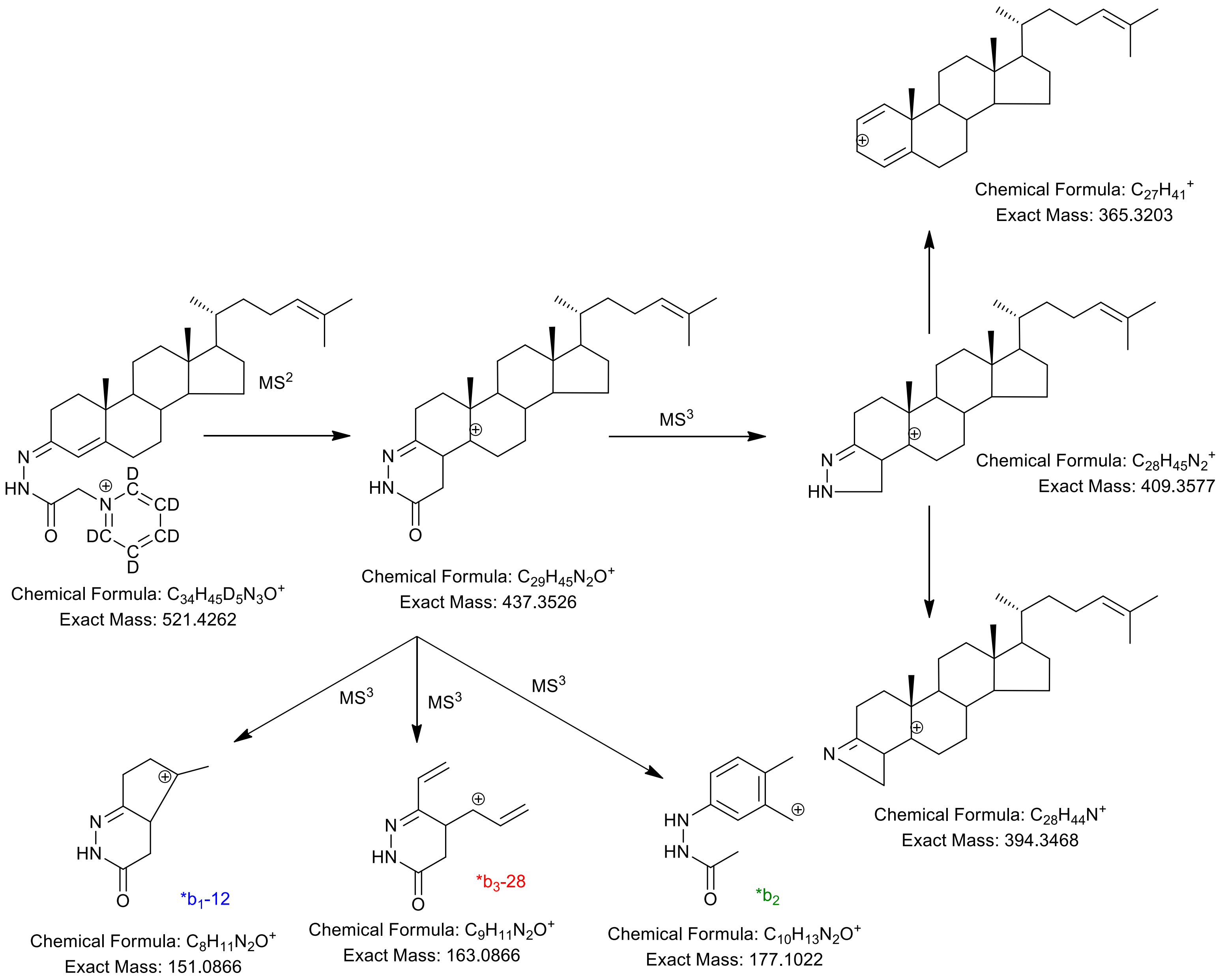
MS^2
MS^3
Chemical Formula: $C_{34}H_{45}D_5N_3O^+$
Exact Mass: 521.4262
Chemical Formula: $C_{29}H_{45}N_2O^+$
Exact Mass: 437.3526
Chemical Formula: $C_{28}H_{45}N_2^+$
Exact Mass: 409.3577
Chemical Formula: $C_{27}H_{41}^+$
Exact Mass: 365.3203
Chemical Formula: $C_{28}H_{44}N^+$
Exact Mass: 394.3468
MS^3
MS^3
MS^3
*b_1-12
Chemical Formula: $C_8H_{11}N_2O^+$
Exact Mass: 151.0866
*b_3-28
Chemical Formula: $C_9H_{11}N_2O^+$
Exact Mass: 163.0866
*b_2
Chemical Formula: $C_{10}H_{13}N_2O^+$
Exact Mass: 177.1022

S9H

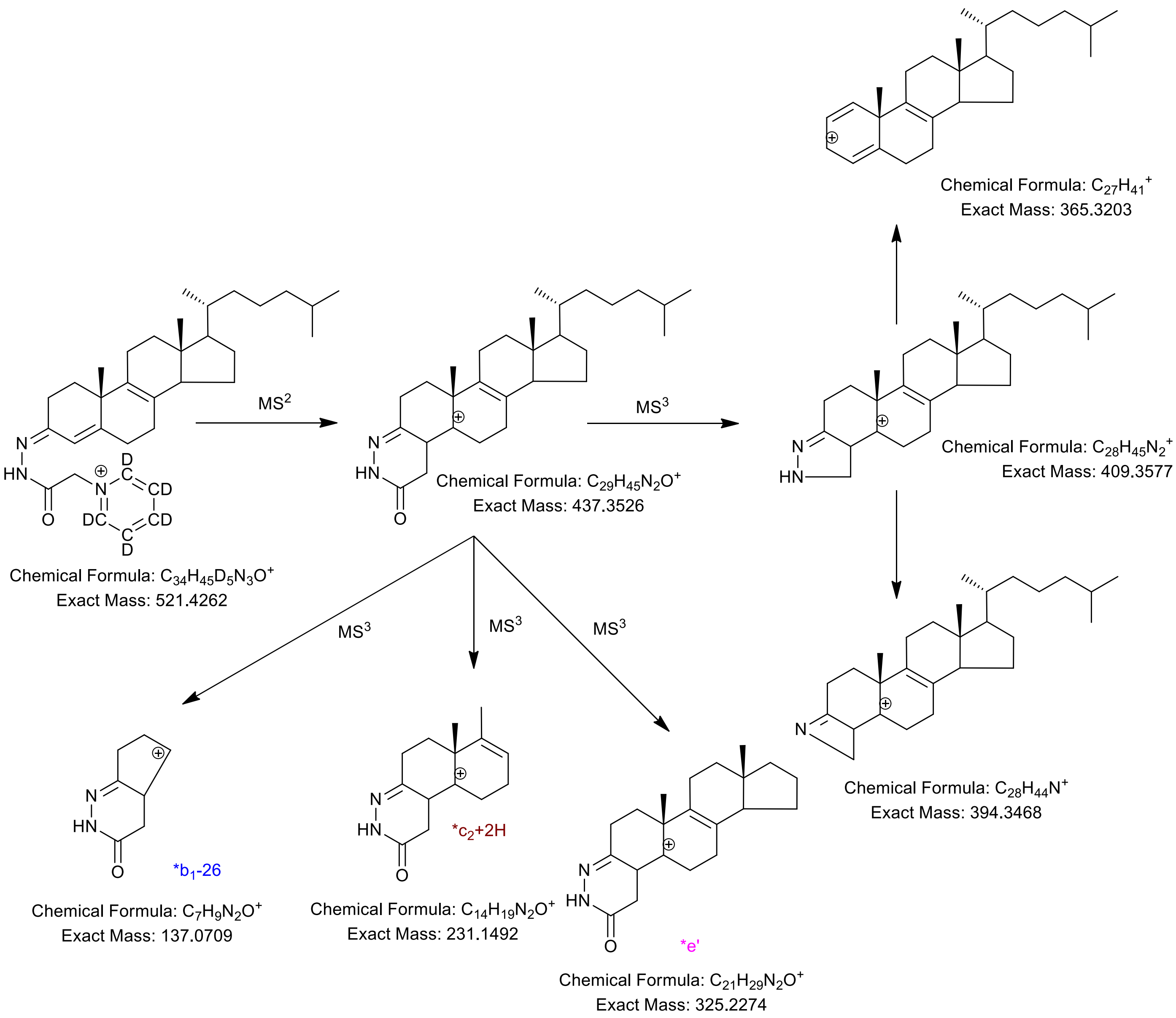

N
HN
O
N
D
C
CD
CD
DC
C
D
Chemical Formula: C34H45D5N3O+
Exact Mass: 521.4262
MS2
N
HN
O
Chemical Formula: C29H45N2O+
Exact Mass: 437.3526
MS3
N
HN
Chemical Formula: C28H45N2+
Exact Mass: 409.3577
Chemical Formula: C27H41+
Exact Mass: 365.3203
N
Chemical Formula: C28H44N+
Exact Mass: 394.3468
MS3
MS3
MS3
N
HN
O
*b1-26
Chemical Formula: C7H9N2O+
Exact Mass: 137.0709
N
HN
O
*c2+2H
Chemical Formula: C14H19N2O+
Exact Mass: 231.1492
N
HN
O
*e'
Chemical Formula: C21H29N2O+
Exact Mass: 325.2274

S10A

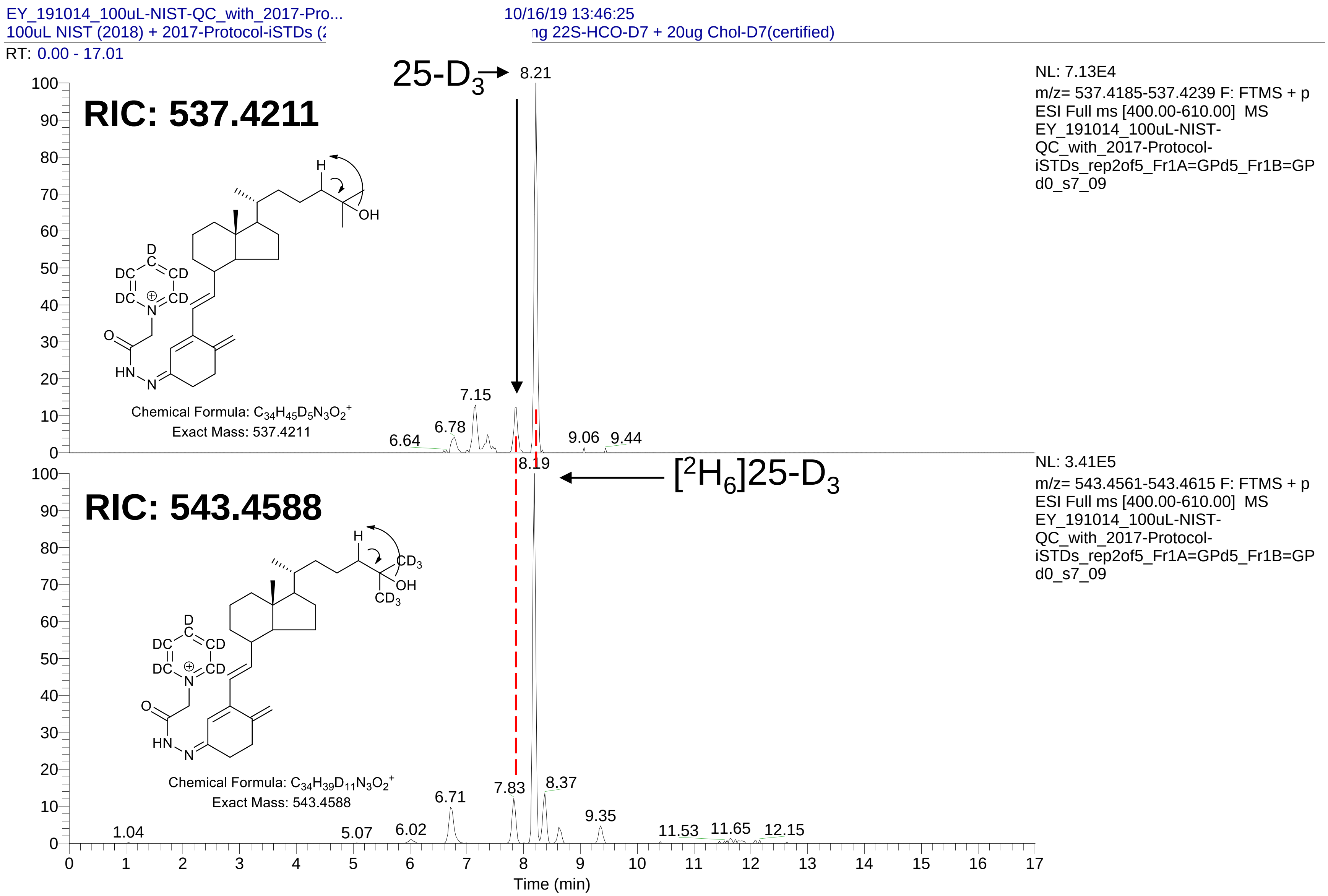

EY_191014_100uL-NIST-QC_with_2017-Pro...
10/16/19 13:46:25
100uL NIST (2018) + 2017-Protocol-iSTDs (2
ng 22S-HCO-D7 + 20ug Chol-D7(certified)
RT: 0.00 - 17.01
25-D3
8.21
NL: 7.13E4
m/z= 537.4185-537.4239 F: FTMS + p ESI Full ms [400.00-610.00] MS EY_191014_100uL-NIST-QC_with_2017-Protocol-iSTDs_rep2of5_Fr1A=GPd5_Fr1B=GPd0_s7_09
RIC: 537.4211
Chemical Formula: C34H45D5N3O2+
Exact Mass: 537.4211
6.64
6.78
7.15
9.06
9.44
8.19
[2H6]25-D3
NL: 3.41E5
m/z= 543.4561-543.4615 F: FTMS + p ESI Full ms [400.00-610.00] MS EY_191014_100uL-NIST-QC_with_2017-Protocol-iSTDs_rep2of5_Fr1A=GPd5_Fr1B=GPd0_s7_09
RIC: 543.4588
Chemical Formula: C34H39D11N3O2+
Exact Mass: 543.4588
1.04
5.07
6.02
6.71
7.83
8.37
9.35
11.53
11.65
12.15
Time (min)

S10B

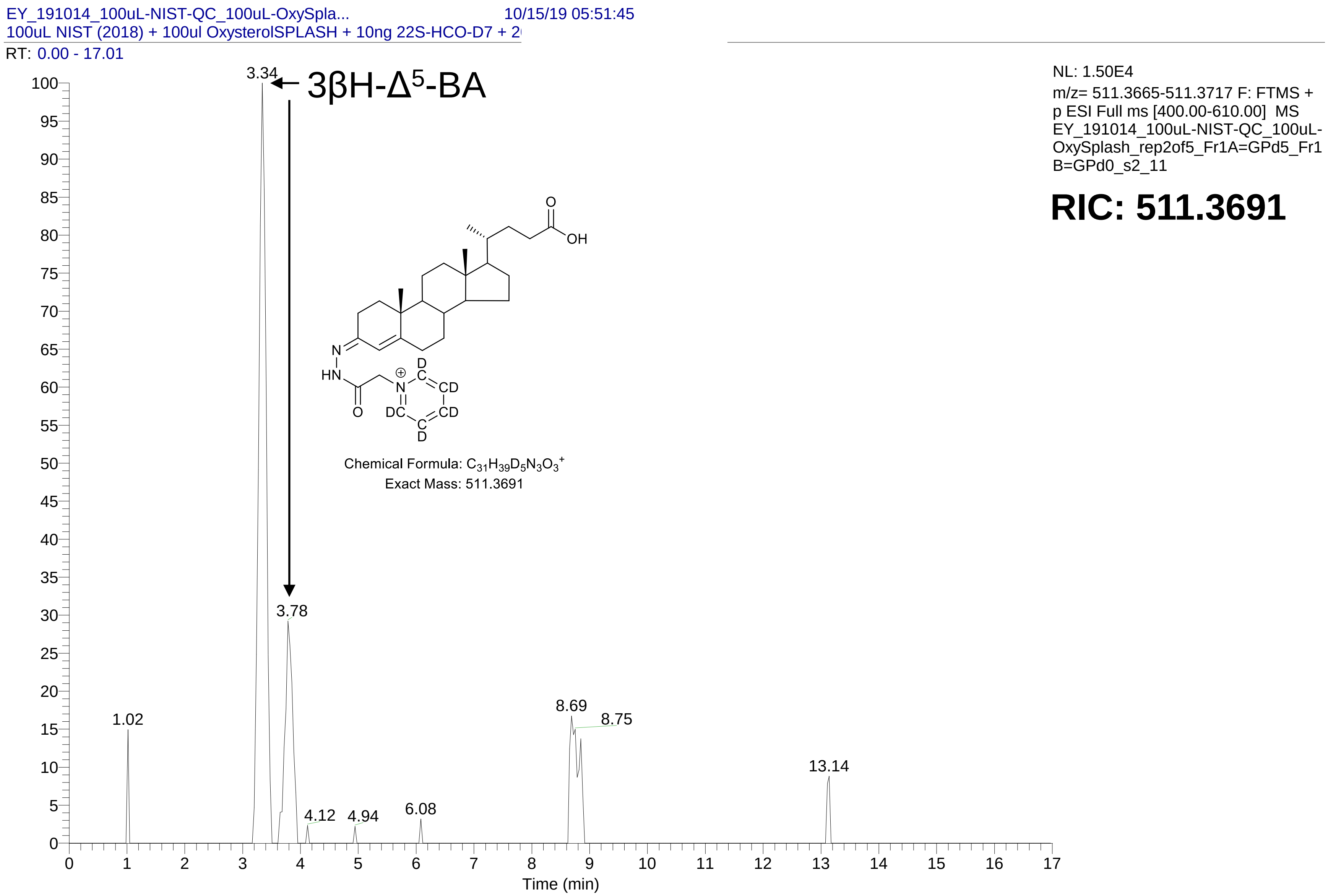
EY_191014_100uL-NIST-QC_100uL-OxySpla...
10/15/19 05:51:45
100uL NIST (2018) + 100ul OxysterolSPLASH + 10ng 22S-HCO-D7 + 2
RT: 0.00 - 17.01
NL: 1.50E4
m/z= 511.3665-511.3717 F: FTMS + p ESI Full ms [400.00-610.00] MS EY_191014_100uL-NIST-QC_100uL-OxySplash_rep2of5_Fr1A=GPd5_Fr1B=GPd0_s2_11
RIC: 511.3691
3βH-Δ5-BA
3.34
3.78
1.02
4.12
4.94
6.08
8.69
8.75
13.14
Chemical Formula: C31H39D5N3O3+
Exact Mass: 511.3691
Time (min)

S10C

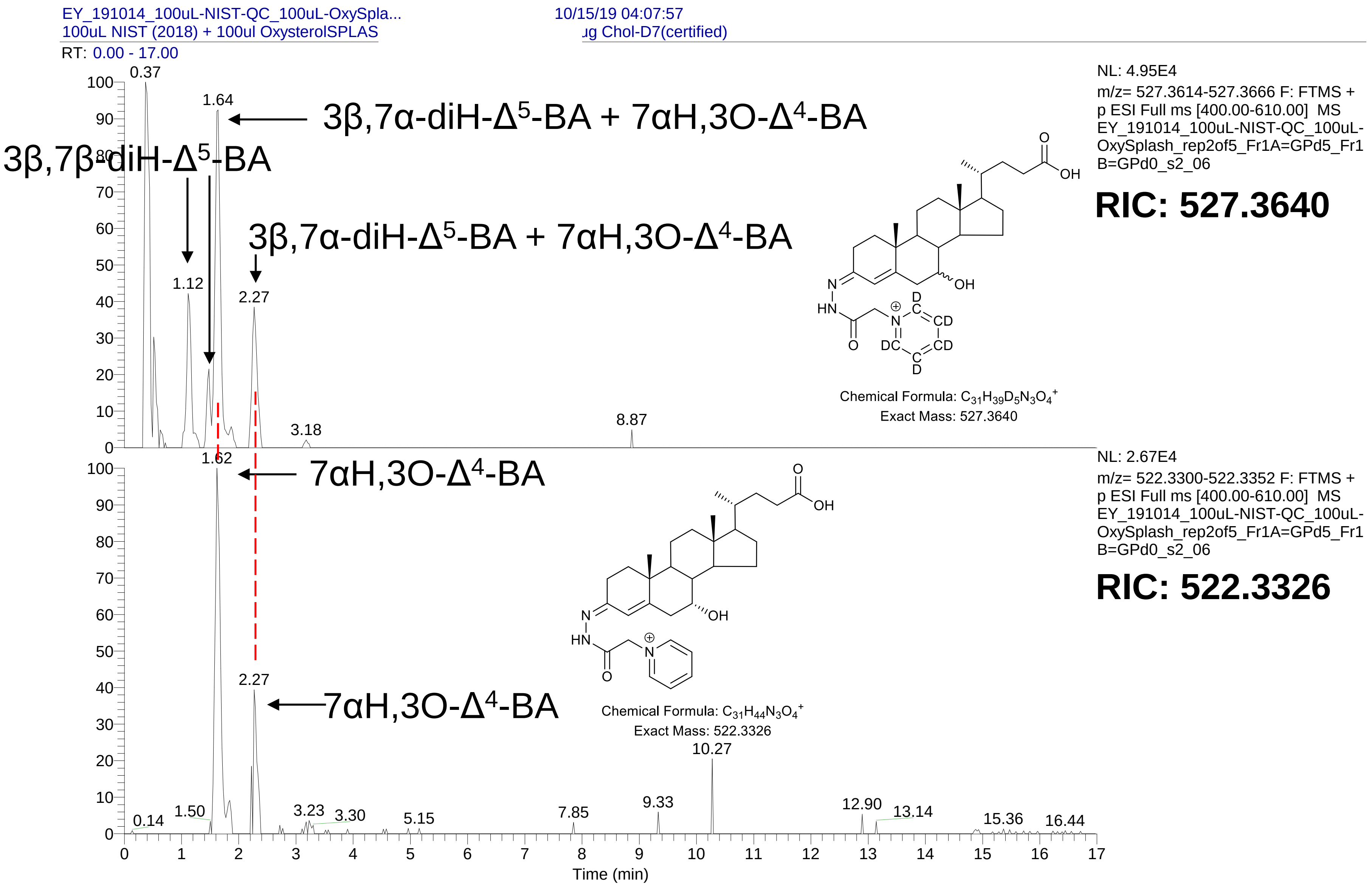
EY_191014_100uL-NIST-QC_100uL-OxySpla...
100uL NIST (2018) + 100ul OxysterolSPLAS
10/15/19 04:07:57
μg Chol-D7(certified)
RT: 0.00 - 17.00
NL: 4.95E4
m/z= 527.3614-527.3666 F: FTMS + p ESI Full ms [400.00-610.00] MS EY_191014_100uL-NIST-QC_100uL-OxySplash_rep2of5_Fr1A=GPd5_Fr1 B=GPd0_s2_06
RIC: 527.3640
3β,7α-diH-Δ5-BA + 7αH,3O-Δ4-BA
3β,7β-diH-Δ5-BA
3β,7α-diH-Δ5-BA + 7αH,3O-Δ4-BA
0.37
1.64
1.12
2.27
3.18
8.87
Chemical Formula: C31H39D5N3O4+
Exact Mass: 527.3640
NL: 2.67E4
m/z= 522.3300-522.3352 F: FTMS + p ESI Full ms [400.00-610.00] MS EY_191014_100uL-NIST-QC_100uL-OxySplash_rep2of5_Fr1A=GPd5_Fr1 B=GPd0_s2_06
RIC: 522.3326
7αH,3O-Δ4-BA
7αH,3O-Δ4-BA
1.62
2.27
0.14
1.50
3.23
3.30
5.15
7.85
9.33
10.27
12.90
13.14
15.36
16.44
Chemical Formula: C31H44N3O4+
Exact Mass: 522.3326
Time (min)

S10D

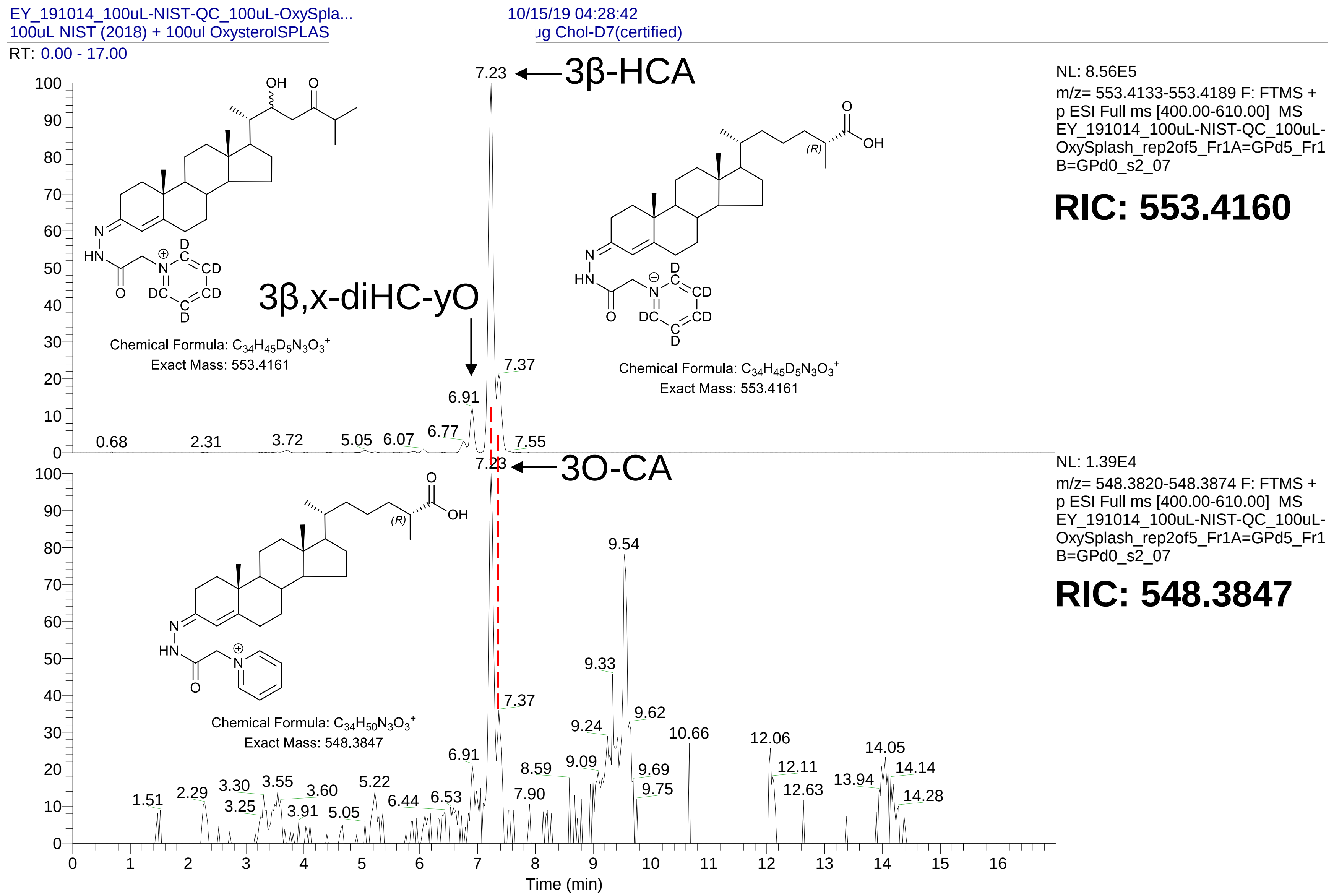
EY_191014_100uL-NIST-QC_100uL-OxySpla...
100uL NIST (2018) + 100ul OxysterolSPLAS
10/15/19 04:28:42
ɹg Chol-D7(certified)
RT: 0.00 - 17.00
3β-HCA
3β,x-diHC-yO
3O-CA
NL: 8.56E5
m/z= 553.4133-553.4189 F: FTMS + p ESI Full ms [400.00-610.00] MS EY_191014_100uL-NIST-QC_100uL-OxySplash_rep2of5_Fr1A=GPd5_Fr1B=GPd0_s2_07
RIC: 553.4160
NL: 1.39E4
m/z= 548.3820-548.3874 F: FTMS + p ESI Full ms [400.00-610.00] MS EY_191014_100uL-NIST-QC_100uL-OxySplash_rep2of5_Fr1A=GPd5_Fr1B=GPd0_s2_07
RIC: 548.3847
Chemical Formula: $C_{34}H_{45}D_5N_3O_3^+$
Exact Mass: 553.4161
Chemical Formula: $C_{34}H_{45}D_5N_3O_3^+$
Exact Mass: 553.4161
Chemical Formula: $C_{34}H_{50}N_3O_3^+$
Exact Mass: 548.3847
Time (min)

S10E

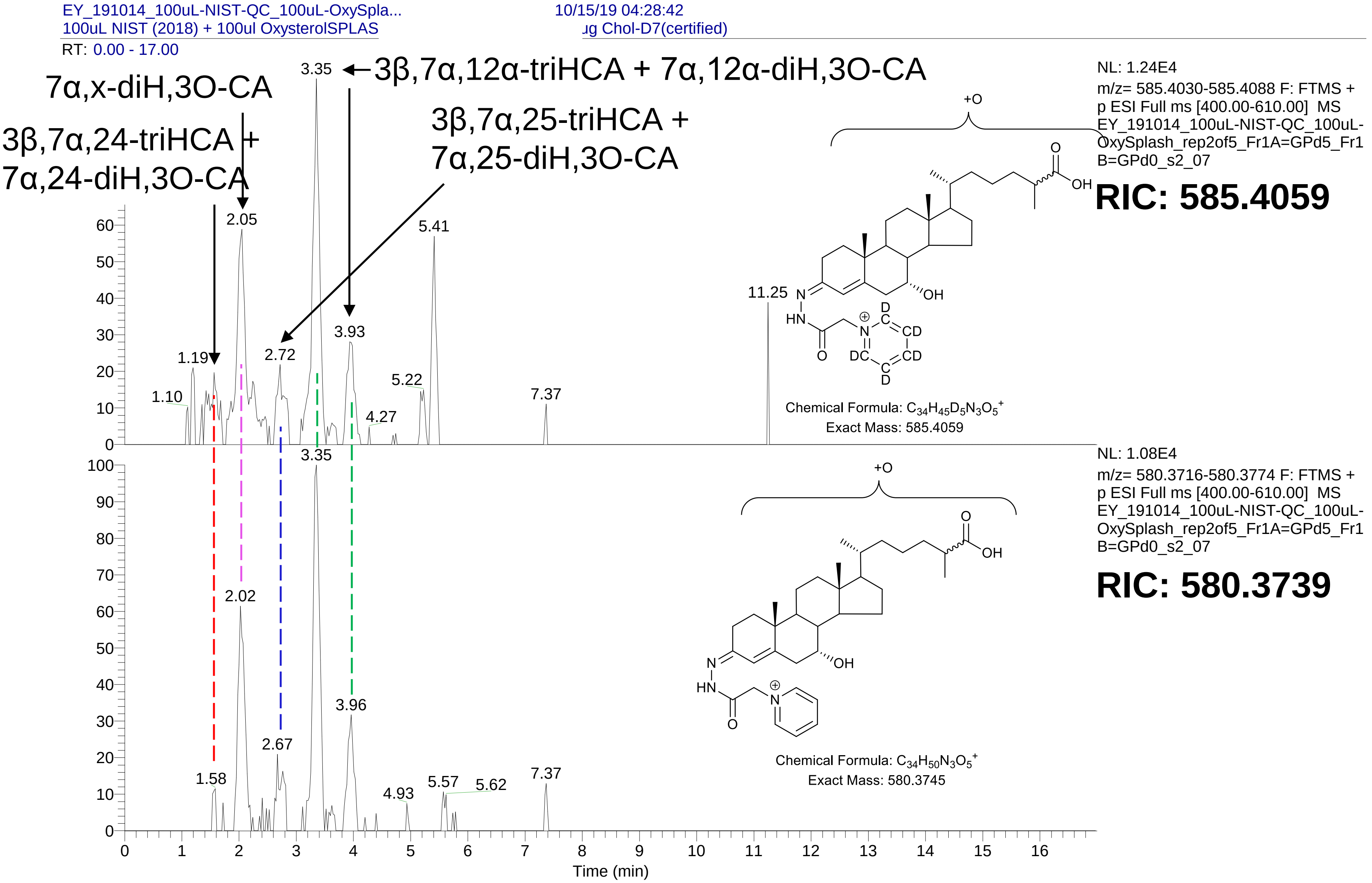
EY_191014_100uL-NIST-QC_100uL-OxySpla...
100uL NIST (2018) + 100ul OxysterolSPLAS
10/15/19 04:28:42
ɹg Chol-D7(certified)
RT: 0.00 - 17.00
7α,x-diH,3O-CA
3β,7α,24-triHCA + 7α,24-diH,3O-CA
3β,7α,12α-triHCA + 7α,12α-diH,3O-CA
3β,7α,25-triHCA + 7α,25-diH,3O-CA
NL: 1.24E4
m/z= 585.4030-585.4088 F: FTMS + p ESI Full ms [400.00-610.00] MS EY_191014_100uL-NIST-QC_100uL-OxySplash_rep2of5_Fr1A=GPd5_Fr1B=GPd0_s2_07
RIC: 585.4059
Chemical Formula: C34H45D5N3O5+
Exact Mass: 585.4059
NL: 1.08E4
m/z= 580.3716-580.3774 F: FTMS + p ESI Full ms [400.00-610.00] MS EY_191014_100uL-NIST-QC_100uL-OxySplash_rep2of5_Fr1A=GPd5_Fr1B=GPd0_s2_07
RIC: 580.3739
Chemical Formula: C34H50N3O5+
Exact Mass: 580.3745
+O
Time (min)
1.10
1.19
2.05
2.72
3.35
3.93
4.27
5.22
5.41
7.37
11.25
1.58
2.02
2.67
3.35
3.96
4.93
5.57
5.62
7.37

S10F

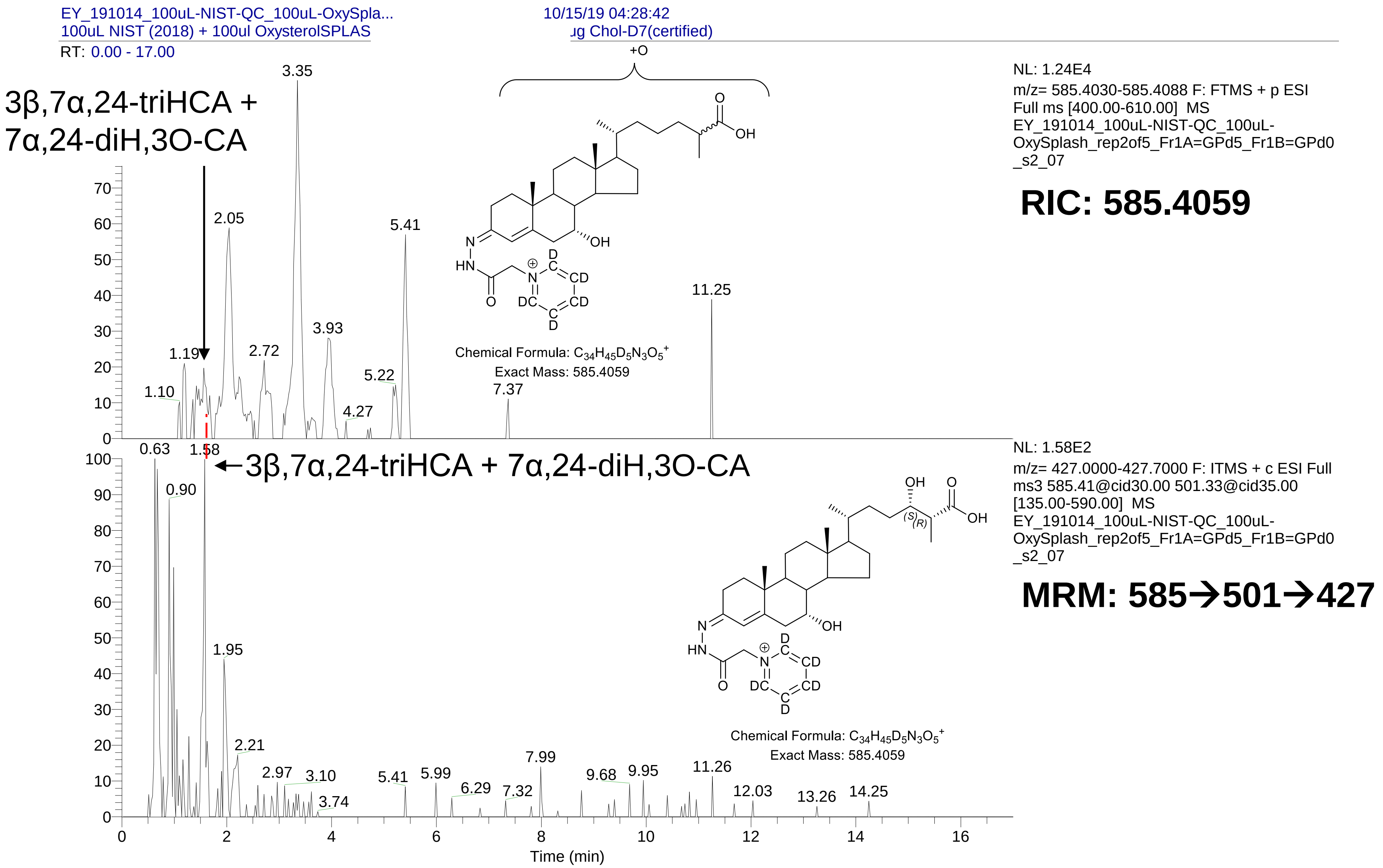
EY_191014_100uL-NIST-QC_100uL-OxySpla...
100uL NIST (2018) + 100ul OxysterolSPLAS
RT: 0.00 - 17.00
10/15/19 04:28:42
ıg Chol-D7(certified)
+O
3β,7α,24-triHCA + 7α,24-diH,3O-CA
NL: 1.24E4
m/z= 585.4030-585.4088 F: FTMS + p ESI Full ms [400.00-610.00] MS EY_191014_100uL-NIST-QC_100uL-OxySplash_rep2of5_Fr1A=GPd5_Fr1B=GPd0_s2_07
RIC: 585.4059
Chemical Formula: $C_{34}H_{45}D_5N_3O_5^+$
Exact Mass: 585.4059
3.35
2.05
5.41
11.25
3.93
1.19
2.72
5.22
1.10
4.27
7.37
0.63
1.58
←3β,7α,24-triHCA + 7α,24-diH,3O-CA
0.90
1.95
2.21
2.97
3.10
3.74
5.41
5.99
6.29
7.32
7.99
9.68
9.95
11.26
12.03
13.26
14.25
NL: 1.58E2
m/z= 427.0000-427.7000 F: ITMS + c ESI Full ms3 585.41@cid30.00 501.33@cid35.00 [135.00-590.00] MS EY_191014_100uL-NIST-QC_100uL-OxySplash_rep2of5_Fr1A=GPd5_Fr1B=GPd0_s2_07
MRM: 585→501→427
Chemical Formula: $C_{34}H_{45}D_5N_3O_5^+$
Exact Mass: 585.4059
Time (min)

S10G

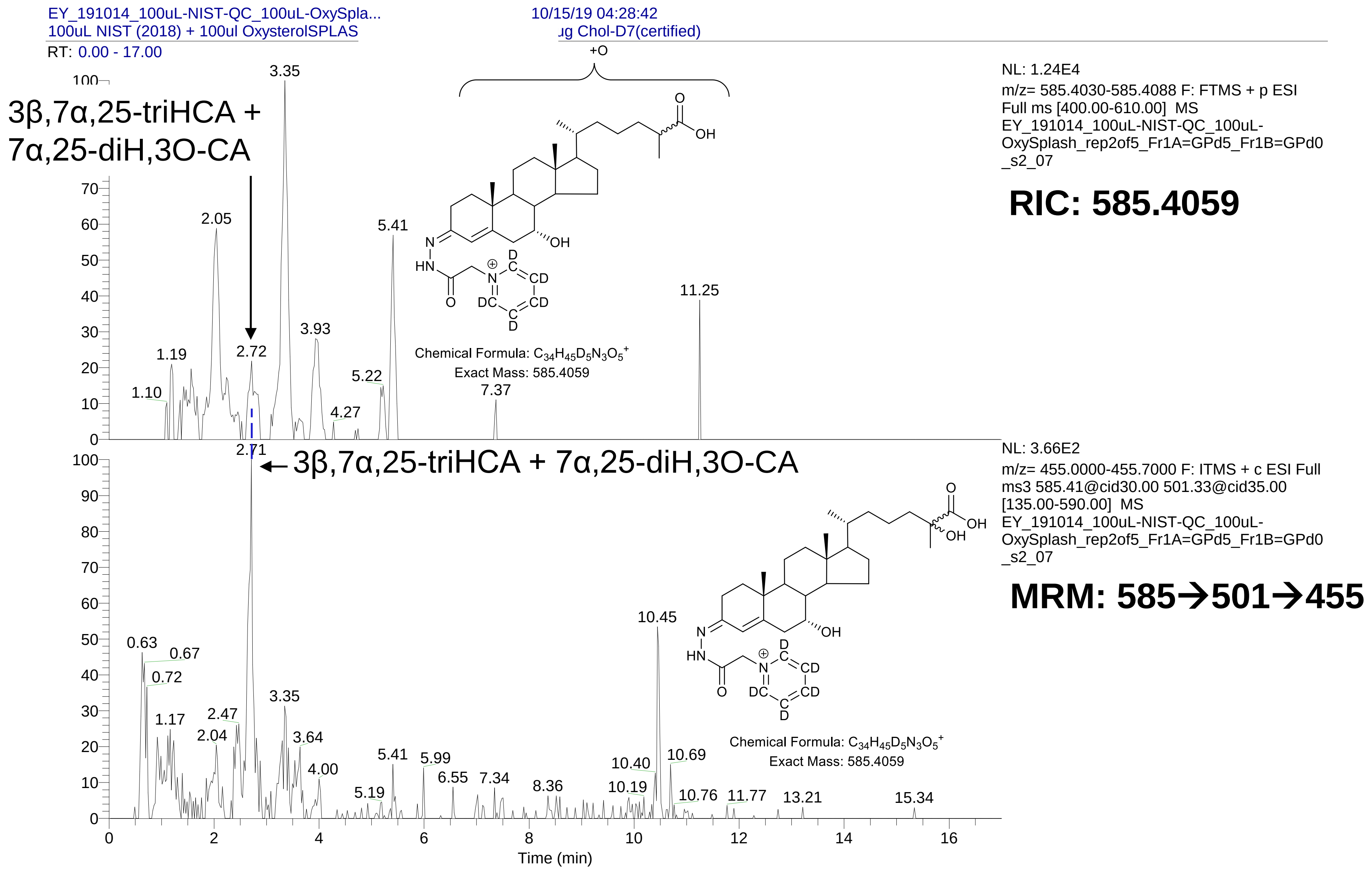

EY_191014_100uL-NIST-QC_100uL-OxySpla...
100uL NIST (2018) + 100ul OxysterolSPLAS
RT: 0.00 - 17.00
10/15/19 04:28:42
ug Chol-D7(certified)
NL: 1.24E4
m/z= 585.4030-585.4088 F: FTMS + p ESI Full ms [400.00-610.00] MS EY_191014_100uL-NIST-QC_100uL-OxySplash_rep2of5_Fr1A=GPd5_Fr1B=GPd0_s2_07
RIC: 585.4059
3β,7α,25-triHCA + 7α,25-diH,3O-CA
+O
Chemical Formula: $C_{34}H_{45}D_5N_3O_5^+$
Exact Mass: 585.4059
NL: 3.66E2
m/z= 455.0000-455.7000 F: ITMS + c ESI Full ms3 585.41@cid30.00 501.33@cid35.00 [135.00-590.00] MS EY_191014_100uL-NIST-QC_100uL-OxySplash_rep2of5_Fr1A=GPd5_Fr1B=GPd0_s2_07
MRM: 585→501→455
3β,7α,25-triHCA + 7α,25-diH,3O-CA
Chemical Formula: $C_{34}H_{45}D_5N_3O_5^+$
Exact Mass: 585.4059
Time (min)

S10H

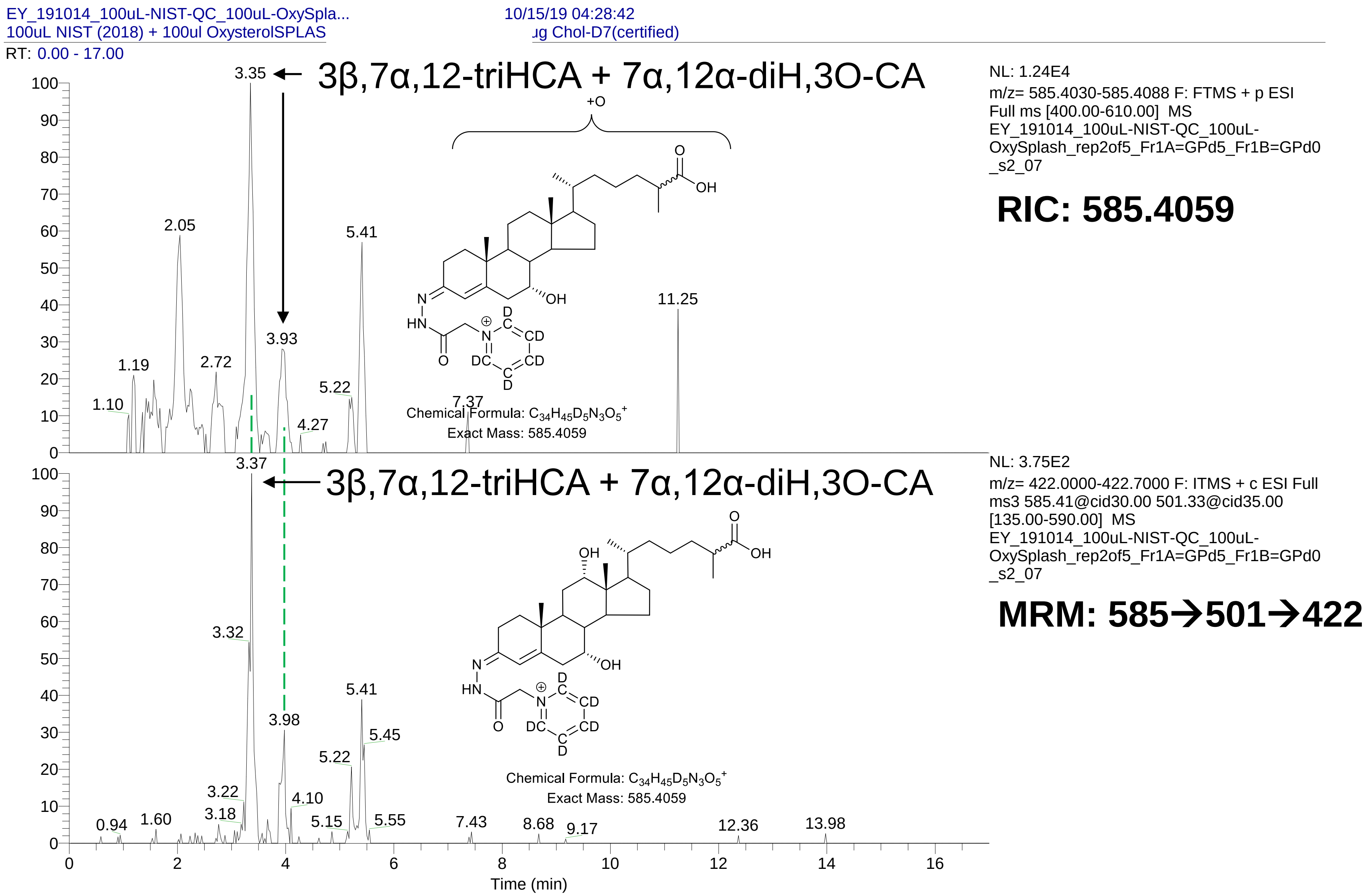

EY_191014_100uL-NIST-QC_100uL-OxySpla...
10/15/19 04:28:42
100uL NIST (2018) + 100ul OxysterolSPLAS
ıg Chol-D7(certified)
RT: 0.00 - 17.00
3β,7α,12-triHCA + 7α,12α-diH,3O-CA
+O
NL: 1.24E4
m/z= 585.4030-585.4088 F: FTMS + p ESI Full ms [400.00-610.00] MS EY_191014_100uL-NIST-QC_100uL-OxySplash_rep2of5_Fr1A=GPd5_Fr1B=GPd0_s2_07
RIC: 585.4059
3.35
2.05
5.41
11.25
3.93
1.19
2.72
5.22
1.10
4.27
7.37
Chemical Formula: $C_{34}H_{45}D_5N_3O_5^+$
Exact Mass: 585.4059
3.37
3β,7α,12-triHCA + 7α,12α-diH,3O-CA
NL: 3.75E2
m/z= 422.0000-422.7000 F: ITMS + c ESI Full ms3 585.41@cid30.00 501.33@cid35.00 [135.00-590.00] MS EY_191014_100uL-NIST-QC_100uL-OxySplash_rep2of5_Fr1A=GPd5_Fr1B=GPd0_s2_07
MRM: 585→501→422
3.32
5.41
3.98
5.45
5.22
3.22
4.10
3.18
0.94
1.60
5.15
5.55
7.43
8.68
9.17
12.36
13.98
Chemical Formula: $C_{34}H_{45}D_5N_3O_5^+$
Exact Mass: 585.4059
Time (min)

11A

EY_191014_100uL-NIST-QC_100uL-OxySpla... 10/15/19 05:10:14
100uL NIST (2018) + 100ul OxysterolSPLASH + 10ng 22S-HCO-D7 + 20ug

EY_191014_100uL-NIST-QC_100uL-OxySplash_rep2of5_Fr1A=GPd5_Fr1E 8.19 AV: 1 NL: 4.20E3
F: ITMS + c ESI Full ms3 537.42@cid30.00 435.34@cid35.00 [115.00-545.(

MS^3: 537→435→
8.19 min (17 min gradient)
25-D_3

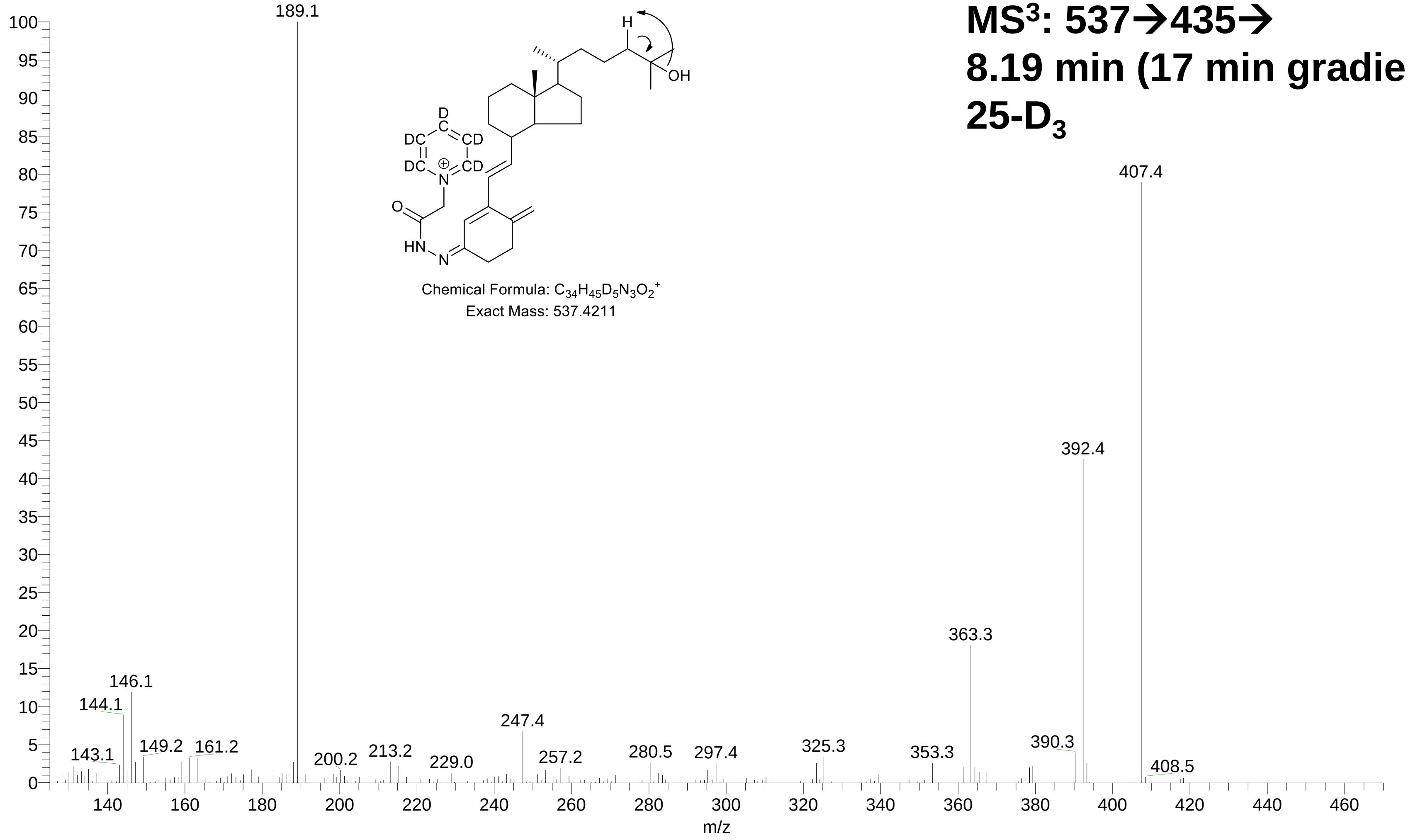

11B

EY_191014_100uL-NIST-QC_with_2017-Pro... 10/16/19 13:46:25
100uL NIST (2018) + 2017-Protocol-iSTDs (20ng 24RS-HC-d6-cert) + 10ng [illegible]7(certified)
EY_191014_100uL-NIST-QC_with_2017-Protocol-iSTDs_rep2of5_Fr1A=GF[illegible] [illegible]50 RT: 8.19 AV: 1 NL: 1.28E4
F: ITMS + c ESI Full ms3 543.46@cid30.00 441.37@cid35.00 [120.00-550.0[illegible]

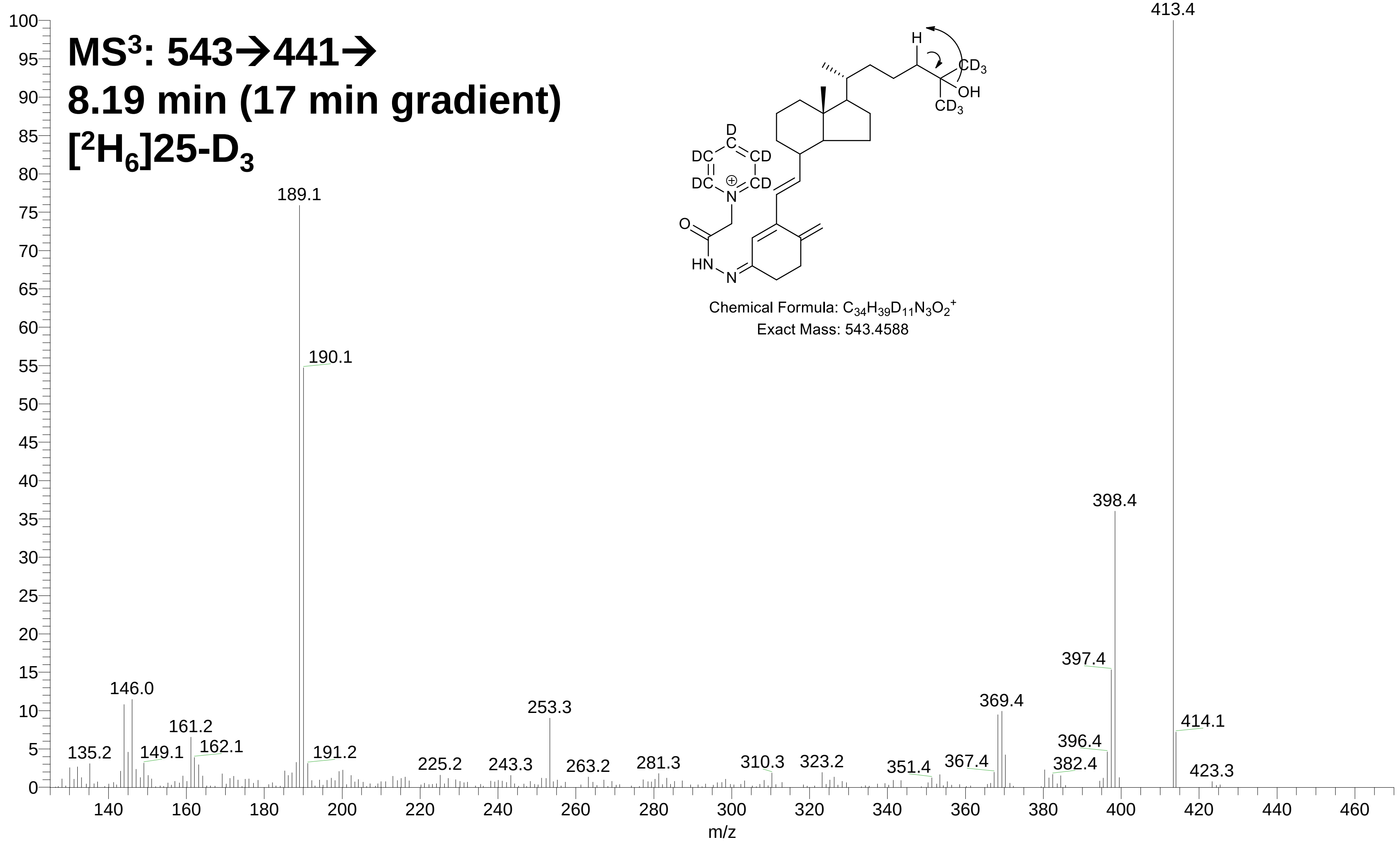

11C

EY_191014_100uL-NIST-QC_100uL-OxySpla... 10/15/19 05:51:45
100uL NIST (2018) + 100ul OxysterolSPLASH + 10ng 22S-HCO-D7 + 20ug

EY_191014_100uL-NIST-QC_100uL-OxySplash_rep2of5_Fr1A=GPd5_Fr1E .35 AV: 1 NL: 6.55E2
F: ITMS + c ESI Full ms3 511.37@cid30.00 427.30@cid35.00 [115.00-515.0

MS³: 511→427→
3.35 min (17 min gradient)
3βH-Δ⁵-BA

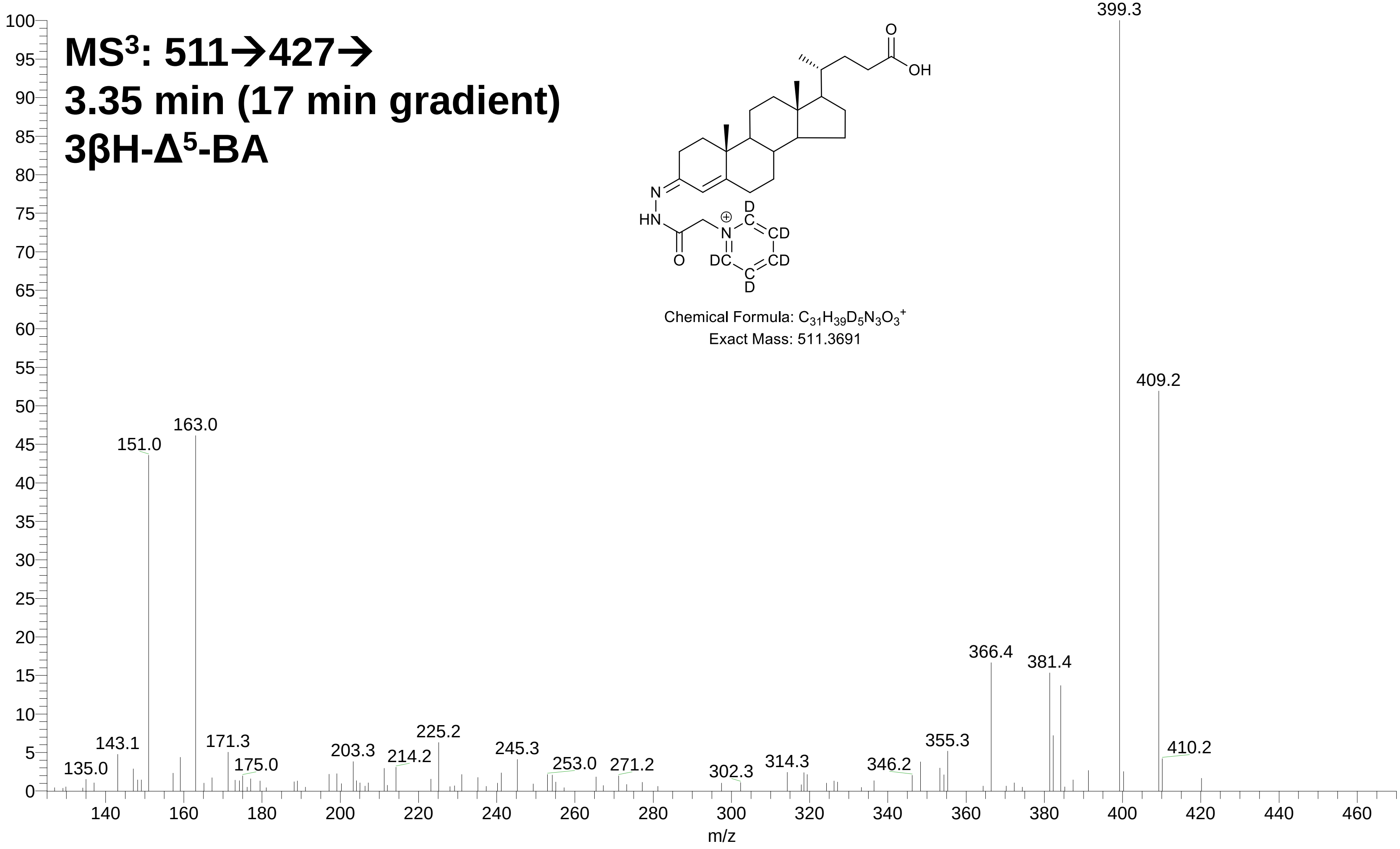

11D

EY_191014_100uL-NIST-QC_100uL-OxySpla... 10/15/19 04:07:57
100uL NIST (2018) + 100ul OxysterolSPLASH + 10ng 22S-HCO-D7 + 20ug
EY_191014_100uL-NIST-QC_100uL-OxySplash_rep2of5_Fr1A=GPd5_Fr1E .12 AV: 1 NL: 1.08E3
F: ITMS + c ESI Full ms3 527.36@cid30.00 443.29@cid35.00 [120.00-535.0

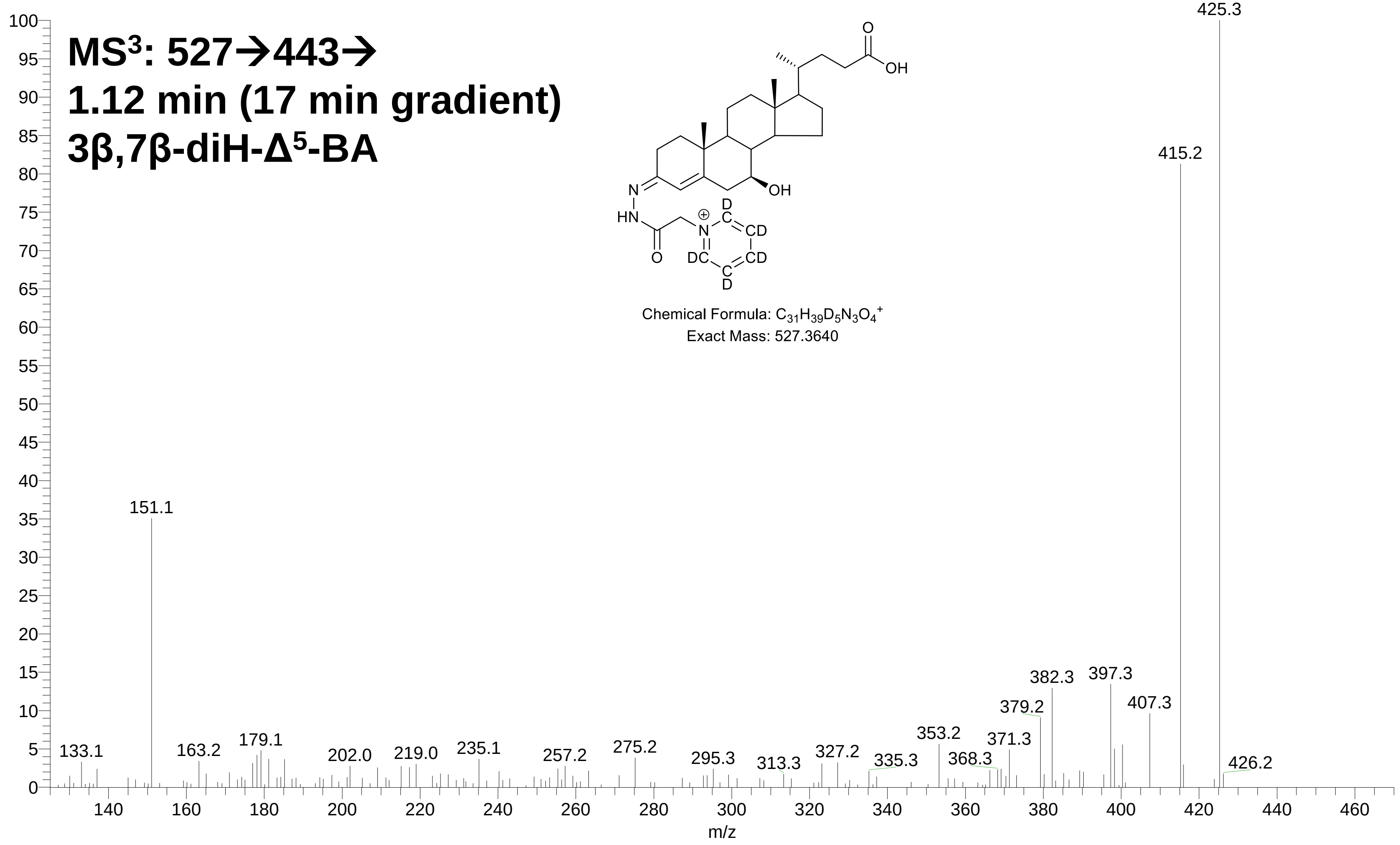

11E

EY_191014_100uL-NIST-QC_100uL-OxySpla... 10/15/19 04:07:57
100uL NIST (2018) + 100ul OxysterolSPLASH + 10ng 22S-HCO-D7 + 20ug
EY_191014_100uL-NIST-QC_100uL-OxySplash_rep2of5_Fr1A=GPd5_Fr1E .62 AV: 1 NL: 3.59E3
F: ITMS + c ESI Full ms3 527.36@cid30.00 443.29@cid35.00 [120.00-535.0

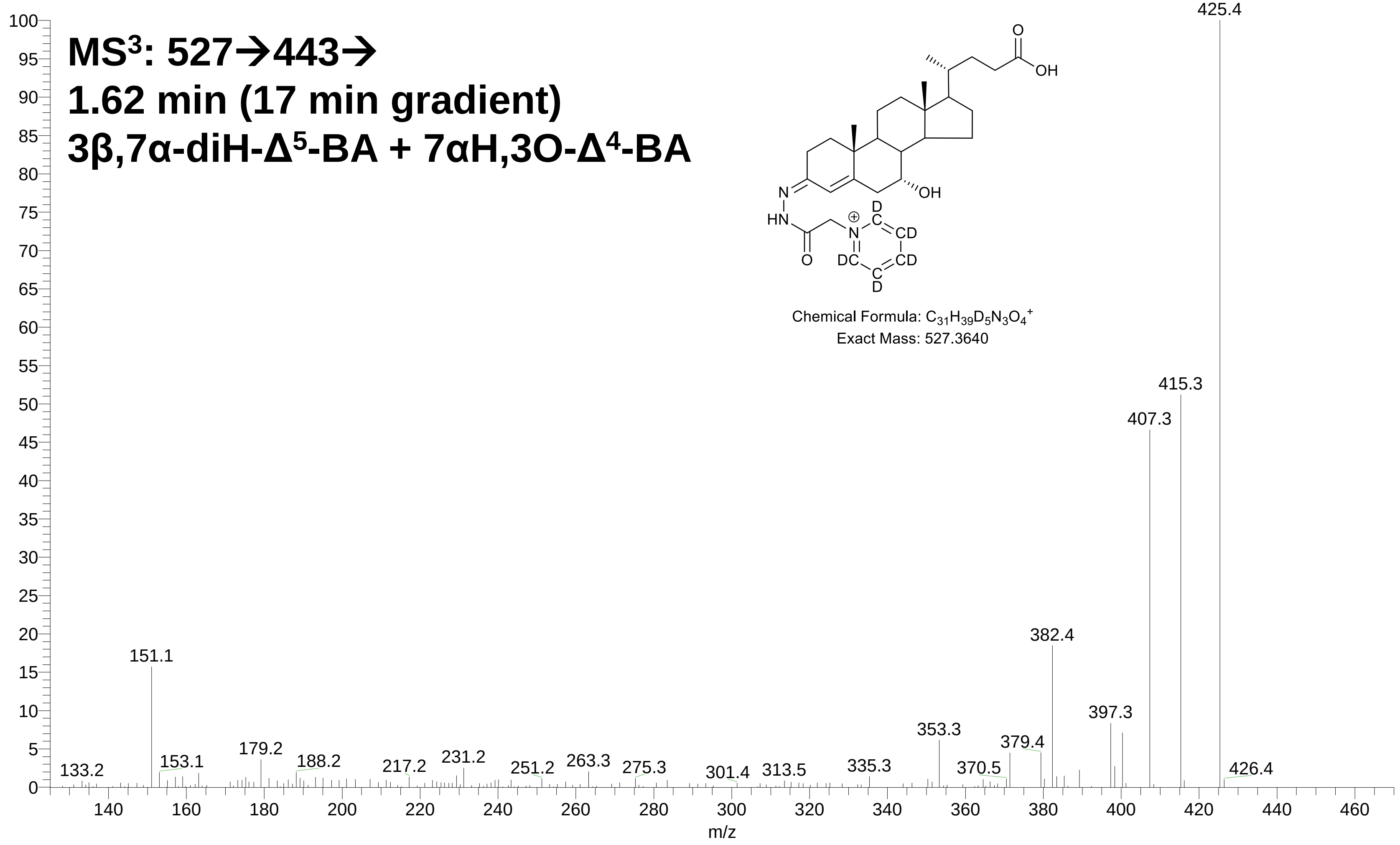

11F

EY_191014_100uL-NIST-QC_100uL-OxySpla... 10/15/19 04:07:57
100uL NIST (2018) + 100ul OxysterolSPLASH + 10ng 22S-HCO-D7 + 20ug
EY_191014_100uL-NIST-QC_100uL-OxySplash_rep2of5_Fr1A=GPd5_Fr1E .64 AV: 1 NL: 2.12E3
F: ITMS + c ESI Full ms3 522.33@cid30.00 443.29@cid35.00 [120.00-535.0

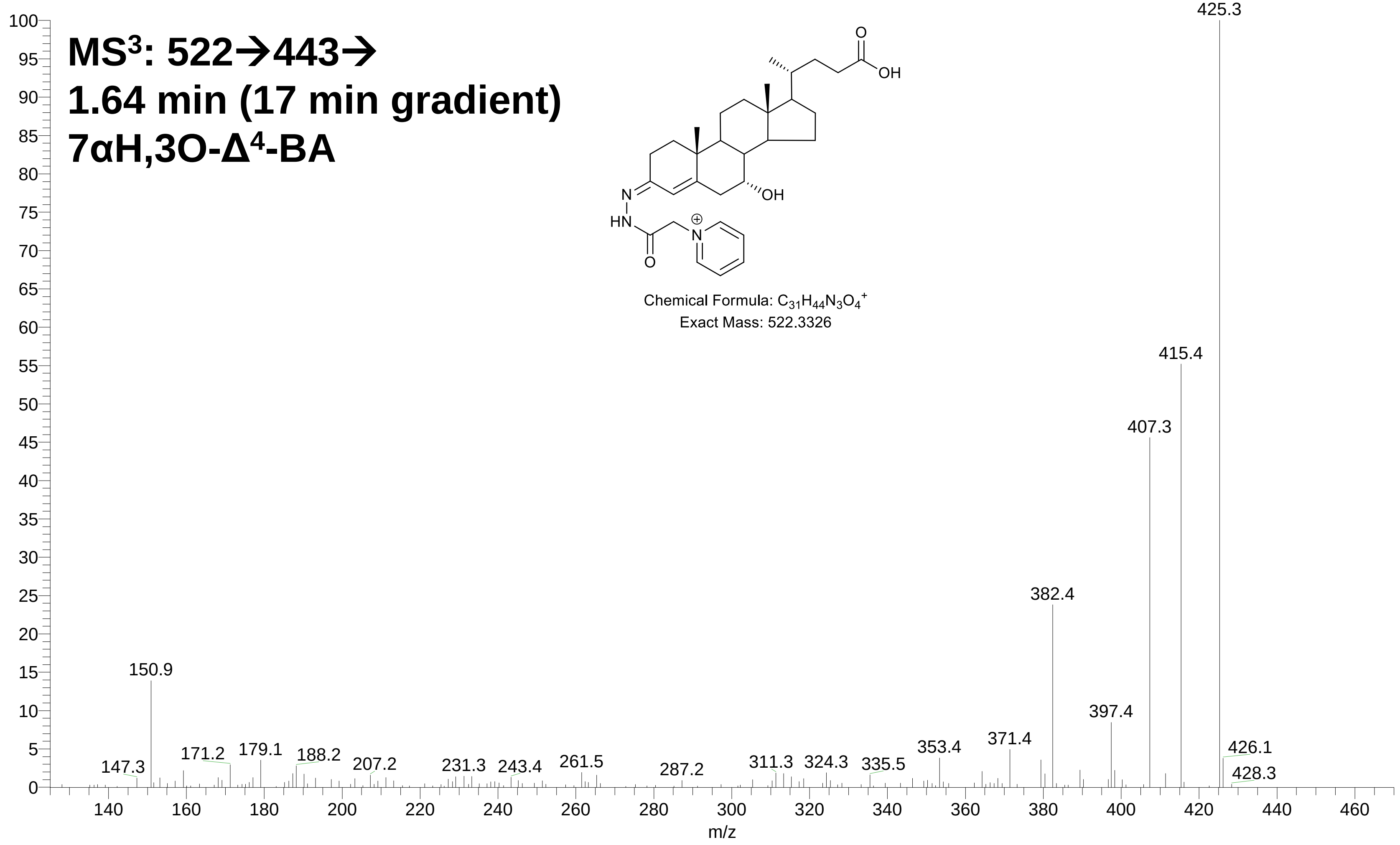

11G

EY_191014_100uL-NIST-QC_100uL-OxySpla... 10/15/19 04:28:42
100uL NIST (2018) + 100ul OxysterolSPLASH + 10ng 22S-HCO-D7 + 20ug
EY_191014_100uL-NIST-QC_100uL-OxySplash_rep2of5_Fr1A=GPd5_Fr1E 7.22 AV: 1 NL: 3.11E4
F: ITMS + c ESI Full ms3 553.42@cid30.00 469.34@cid35.00 [125.00-560.0

MS^3: 553→469→
7.22 min (17 min gradient)
3β-HCA

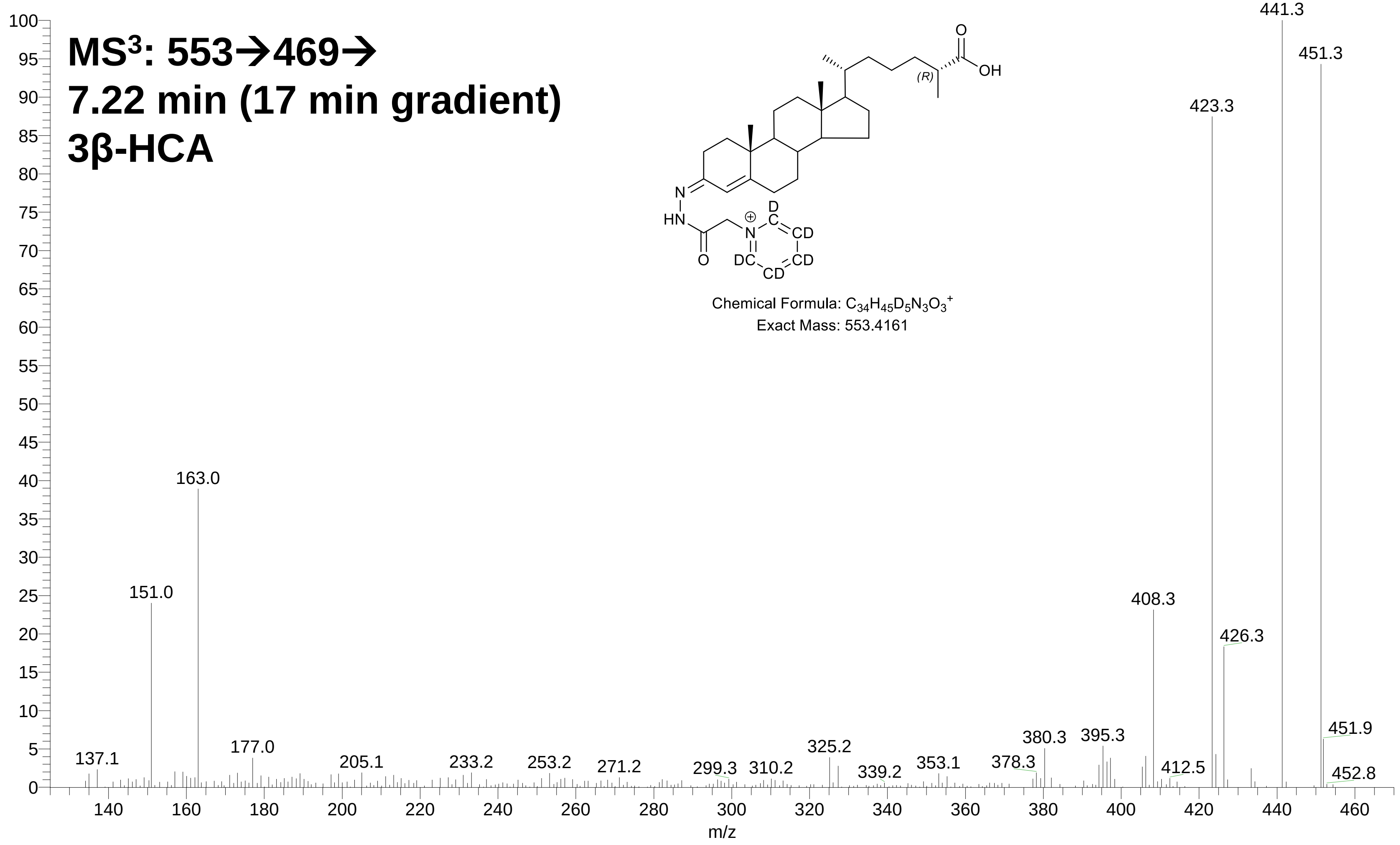

Chemical Formula: $C_{34}H_{45}D_5N_3O_3^+$
Exact Mass: 553.4161

11H

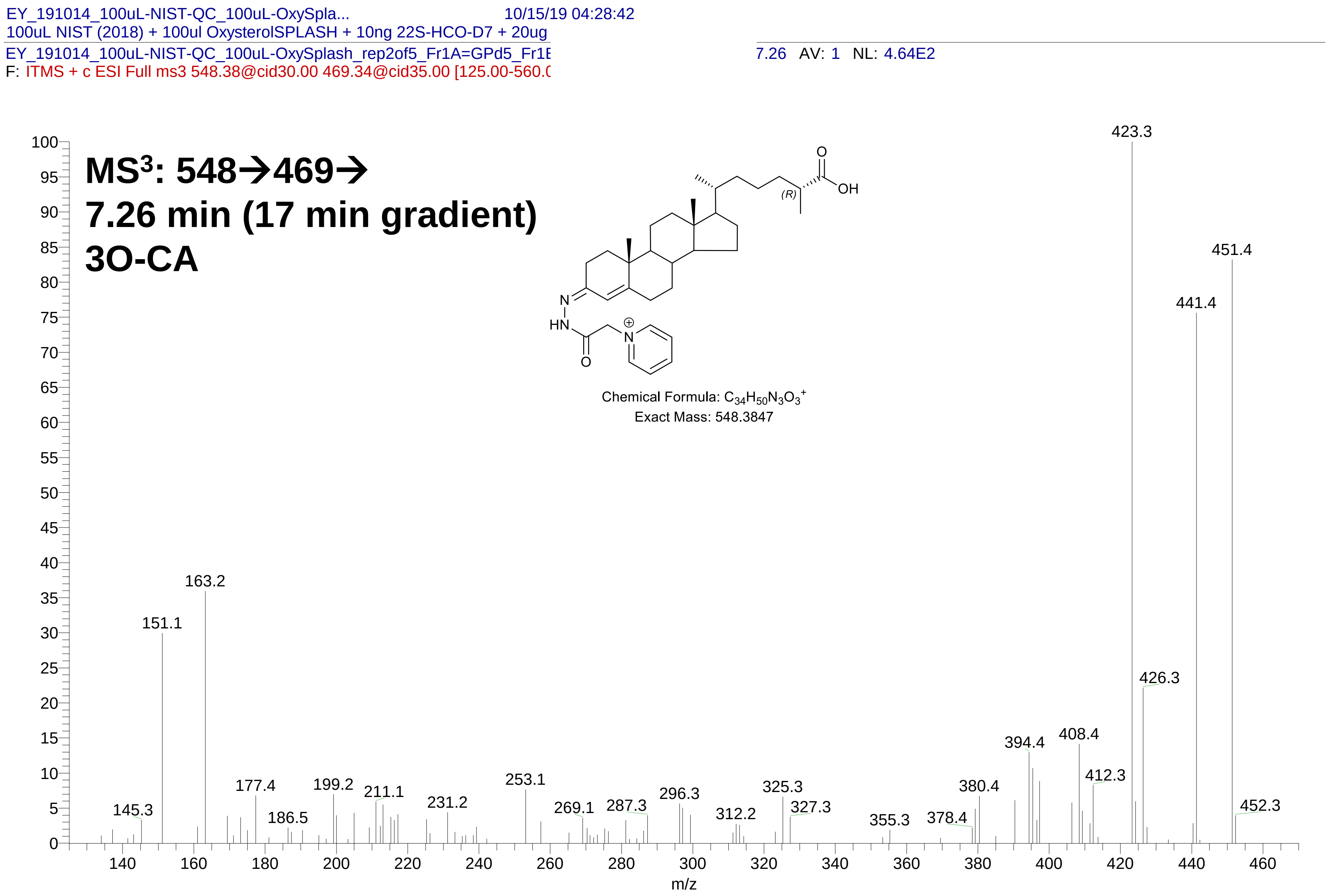
EY_191014_100uL-NIST-QC_100uL-OxySpla... 10/15/19 04:28:42
100uL NIST (2018) + 100ul OxysterolSPLASH + 10ng 22S-HCO-D7 + 20ug
EY_191014_100uL-NIST-QC_100uL-OxySplash_rep2of5_Fr1A=GPd5_Fr1E
7.26 AV: 1 NL: 4.64E2
F: ITMS + c ESI Full ms3 548.38@cid30.00 469.34@cid35.00 [125.00-560.0
MS3: 548→469→
7.26 min (17 min gradient)
3O-CA
Chemical Formula: C34H50N3O3+
Exact Mass: 548.3847
423.3
451.4
441.4
426.3
408.4
394.4
412.3
380.4
378.4
355.3
327.3
325.3
312.2
296.3
287.3
269.1
253.1
231.2
211.1
199.2
186.5
177.4
163.2
151.1
145.3
452.3
m/z

S12A

EY_191014_100uL-NIST-QC_100uL-OxySpla... 10/15/19 04:28:42
100uL NIST (2018) + 100ul OxysterolSPLASH + 10ng 22S-HCO-D7 + 20ug
EY_191014_100uL-NIST-QC_100uL-OxySplash_rep2of5_Fr1A=GPd5_Fr1E [illegible] T: 1.56-1.60 AV: 3 NL: 5.27E1
F: ITMS + c ESI Full ms3 585.41@cid30.00 501.33@cid35.00 [135.00-590.0

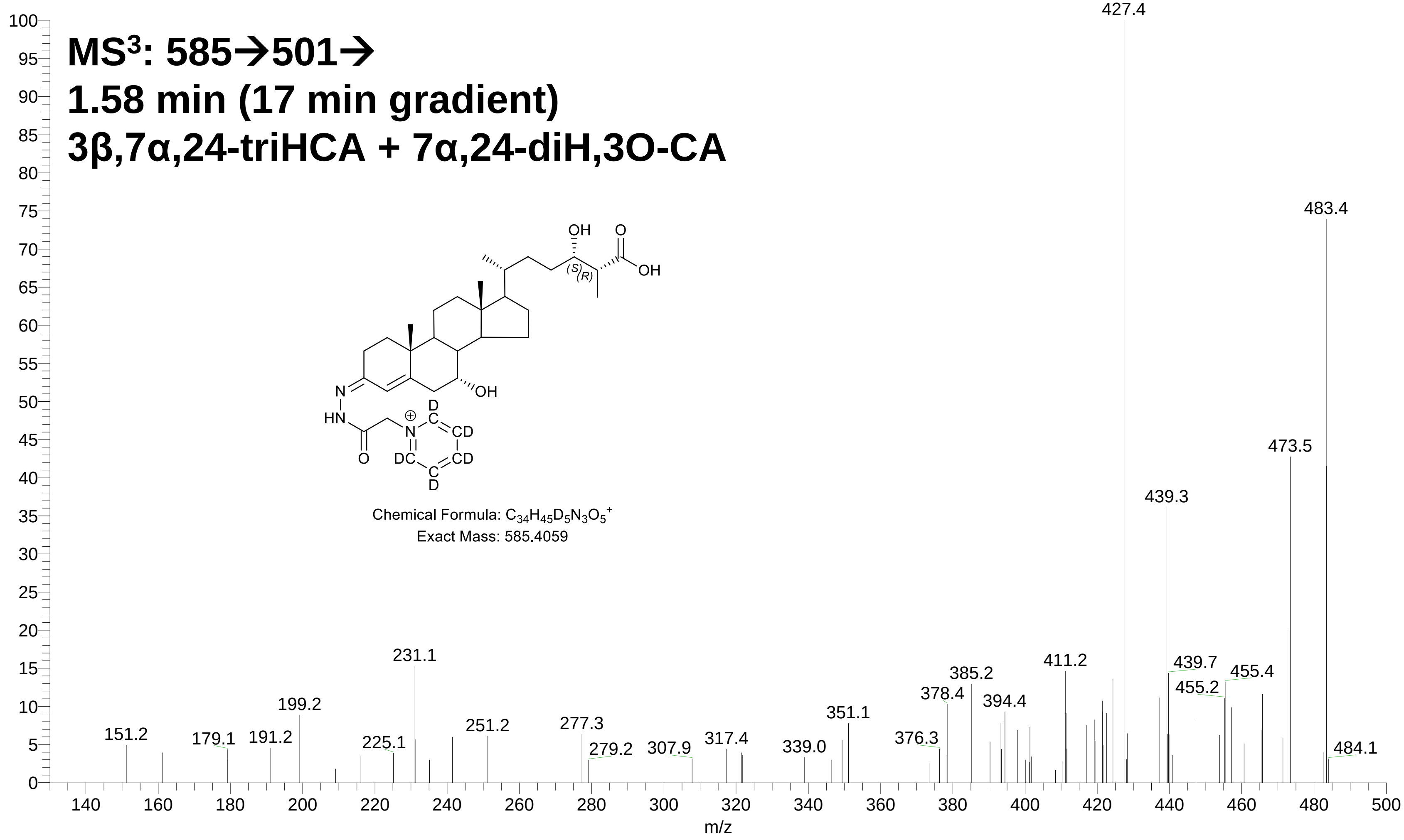

S12B

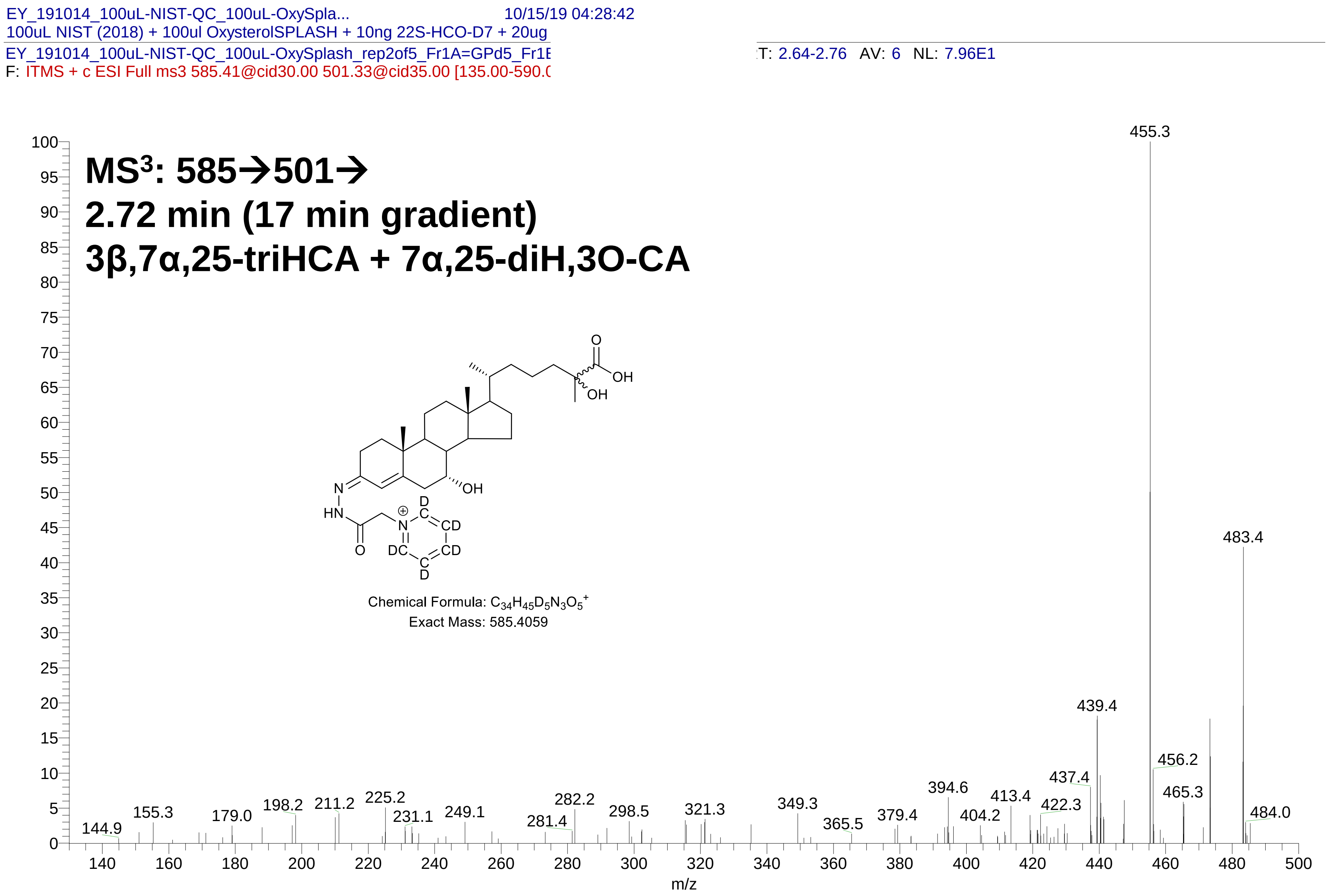
EY_191014_100uL-NIST-QC_100uL-OxySpla... 10/15/19 04:28:42
100uL NIST (2018) + 100ul OxysterolSPLASH + 10ng 22S-HCO-D7 + 20ug
EY_191014_100uL-NIST-QC_100uL-OxySplash_rep2of5_Fr1A=GPd5_Fr1E
F: ITMS + c ESI Full ms3 585.41@cid30.00 501.33@cid35.00 [135.00-590.0
T: 2.64-2.76 AV: 6 NL: 7.96E1
MS3: 585→501→
2.72 min (17 min gradient)
3β,7α,25-triHCA + 7α,25-diH,3O-CA
Chemical Formula: C34H45D5N3O5+
Exact Mass: 585.4059
455.3
483.4
439.4
456.2
437.4
465.3
394.6
413.4
422.3
484.0
144.9
155.3
179.0
198.2
211.2
225.2
231.1
249.1
281.4
282.2
298.5
321.3
349.3
365.5
379.4
404.2
m/z

12C

EY_191014_100uL-NIST-QC_100uL-OxySpla... 10/15/19 04:28:42
100uL NIST (2018) + 100ul OxysterolSPLASH + 10ng 22S-HCO-D7 + 20ug

EY_191014_100uL-NIST-QC_100uL-OxySplash_rep2of5_Fr1A=GPd5_Fr1E .37 AV: 1 NL: 5.89E2
F: ITMS + c ESI Full ms3 585.41@cid30.00 501.33@cid35.00 [135.00-590.(

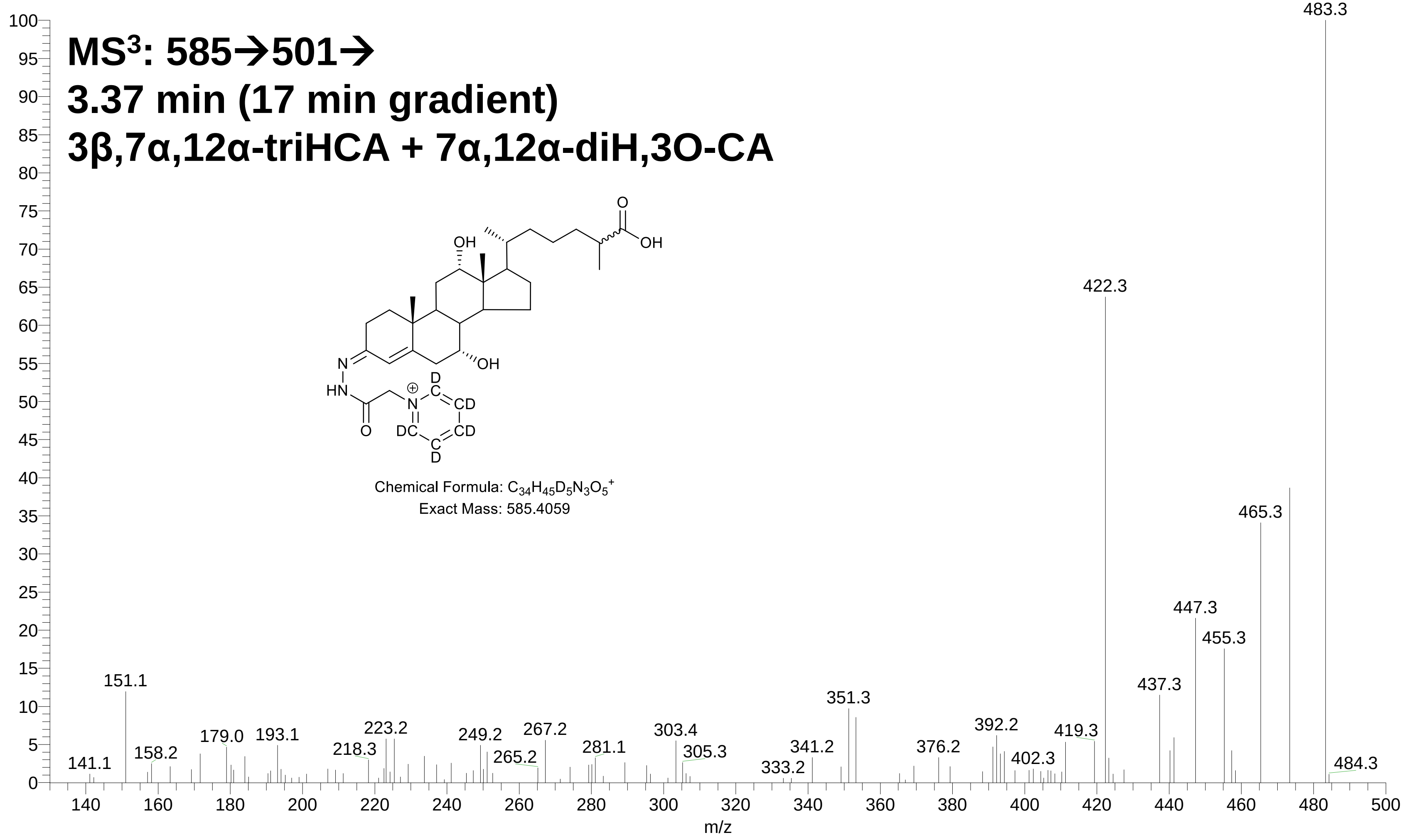

S13A

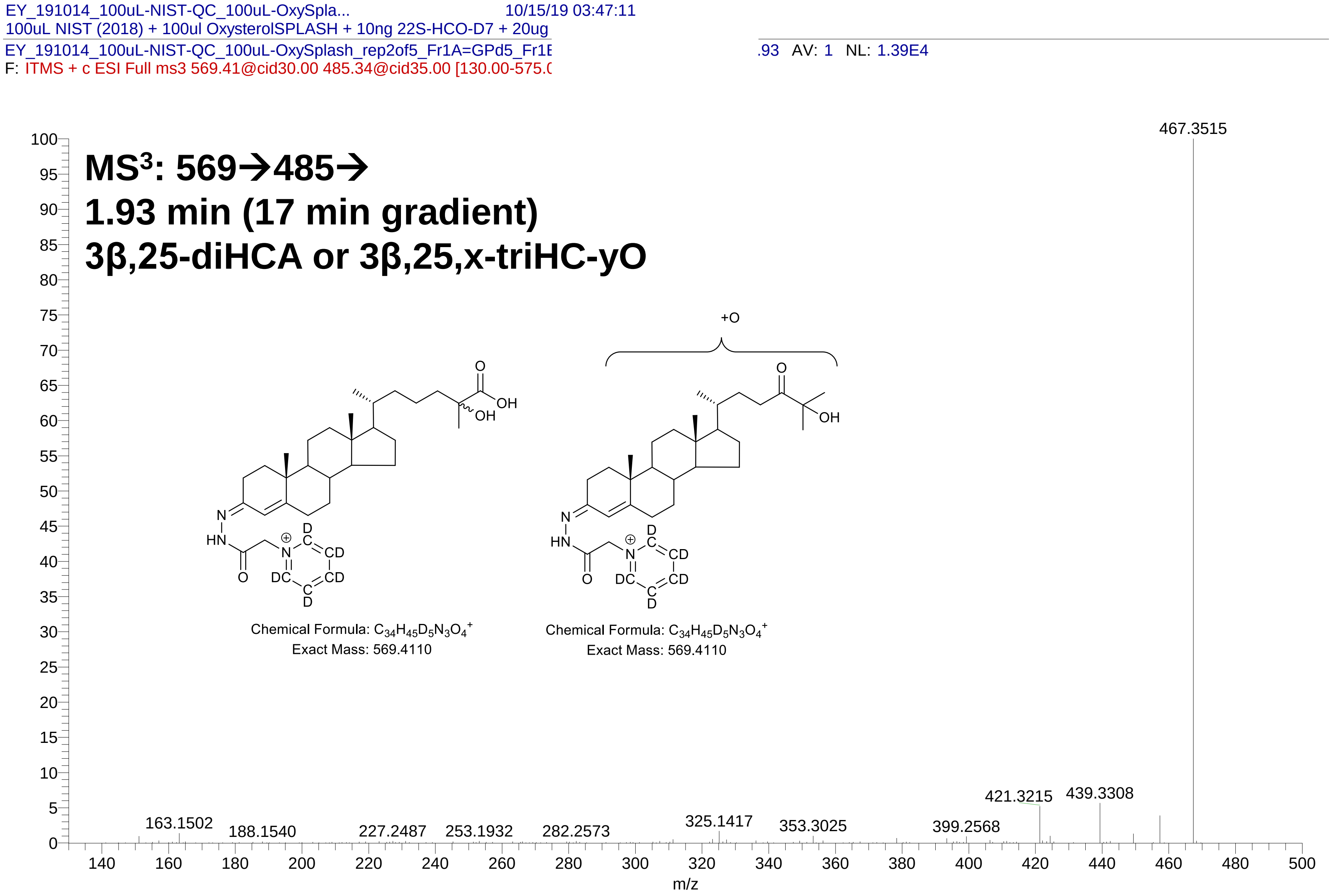

EY_191014_100uL-NIST-QC_100uL-OxySpla... 10/15/19 03:47:11
100uL NIST (2018) + 100ul OxysterolSPLASH + 10ng 22S-HCO-D7 + 20ug
EY_191014_100uL-NIST-QC_100uL-OxySplash_rep2of5_Fr1A=GPd5_Fr1E
.93 AV: 1 NL: 1.39E4
F: ITMS + c ESI Full ms3 569.41@cid30.00 485.34@cid35.00 [130.00-575.0
MS3: 569→485→
1.93 min (17 min gradient)
3β,25-diHCA or 3β,25,x-triHC-yO
+O
Chemical Formula: C34H45D5N3O4+
Exact Mass: 569.4110
Chemical Formula: C34H45D5N3O4+
Exact Mass: 569.4110
467.3515
439.3308
421.3215
399.2568
353.3025
325.1417
282.2573
253.1932
227.2487
188.1540
163.1502
m/z

S13B

EY_191014_100uL-NIST-QC_100uL-OxySpla... 10/15/19 03:47:11
100uL NIST (2018) + 100ul OxysterolSPLASH + 10ng 22S-HCO-D7 + 20ug

EY_191014_100uL-NIST-QC_100uL-OxySplash_rep2of5_Fr1A=GPd5_Fr1E .47 AV: 1 NL: 5.48E2
F: ITMS + c ESI Full ms3 569.41@cid30.00 485.34@cid35.00 [130.00-575.0

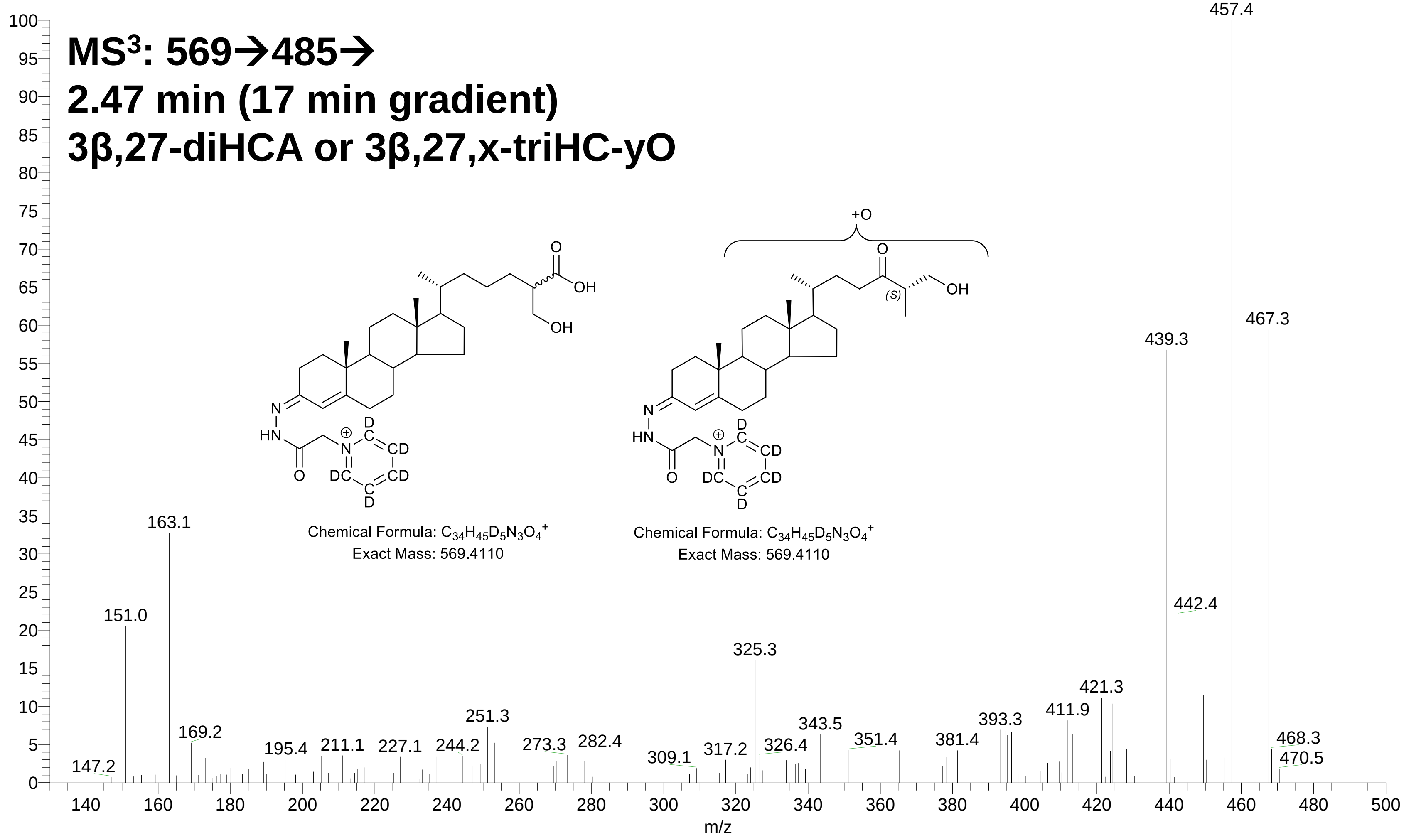

S13C

EY_191014_100uL-NIST-QC_100uL-OxySpla... 10/15/19 03:47:11
100uL NIST (2018) + 100ul OxysterolSPLASH + 10ng 22S-HCO-D7 + 20ug
EY_191014_100uL-NIST-QC_100uL-OxySplash_rep2of5_Fr1A=GPd5_Fr1E .97 AV: 1 NL: 1.70E2
F: ITMS + c ESI Full ms3 569.41@cid30.00 485.34@cid35.00 [130.00-575.0

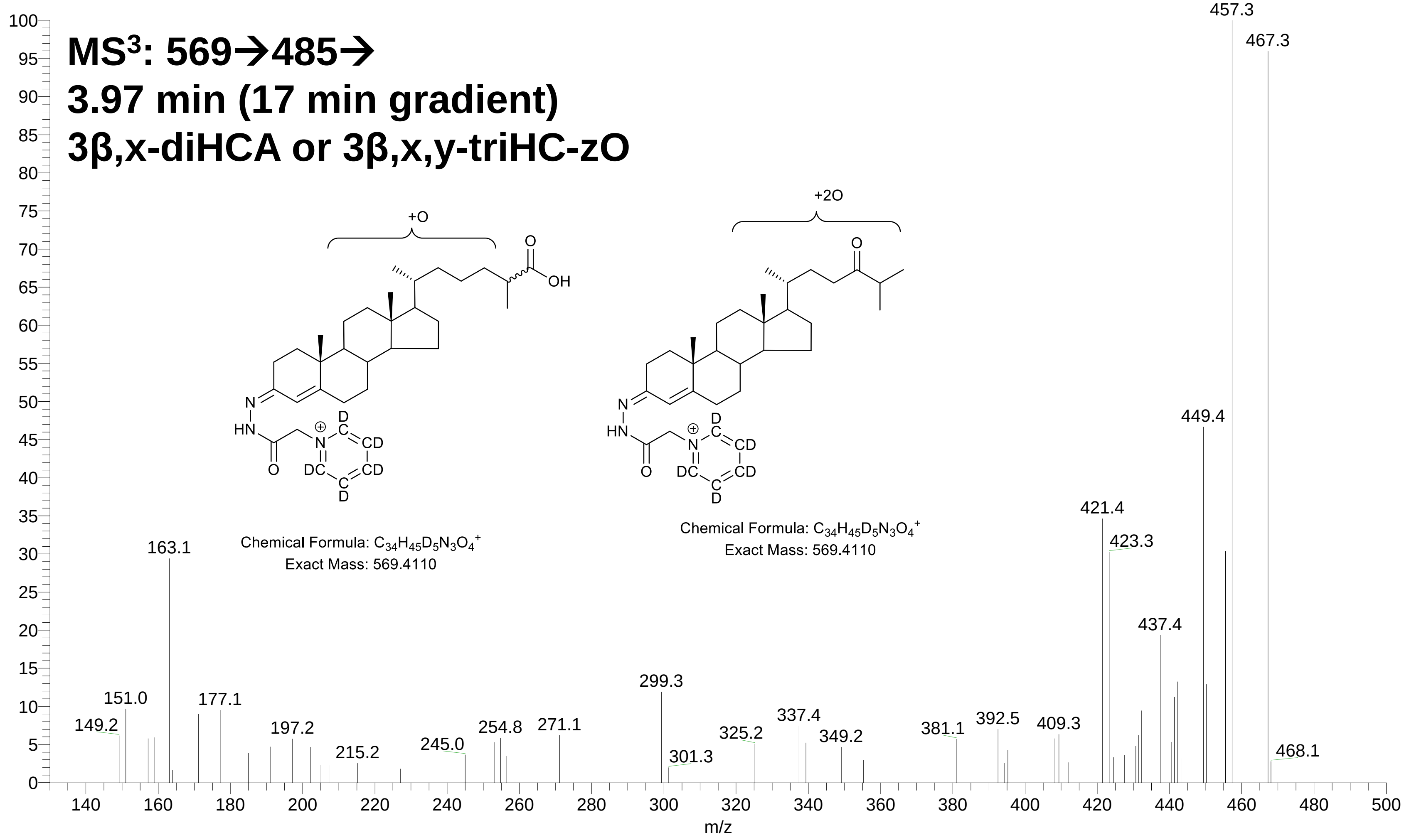

S13D

EY_191014_100uL-NIST-QC_100uL-OxySpla... 10/15/19 03:47:11
100uL NIST (2018) + 100ul OxysterolSPLASH + 10ng 22S-HCO-D7 + 20ug

EY_191014_100uL-NIST-QC_100uL-OxySplash_rep2of5_Fr1A=GPd5_Fr1E .48 AV: 1 NL: 6.92E3
F: ITMS + c ESI Full ms3 569.41@cid30.00 485.34@cid35.00 [130.00-575.0

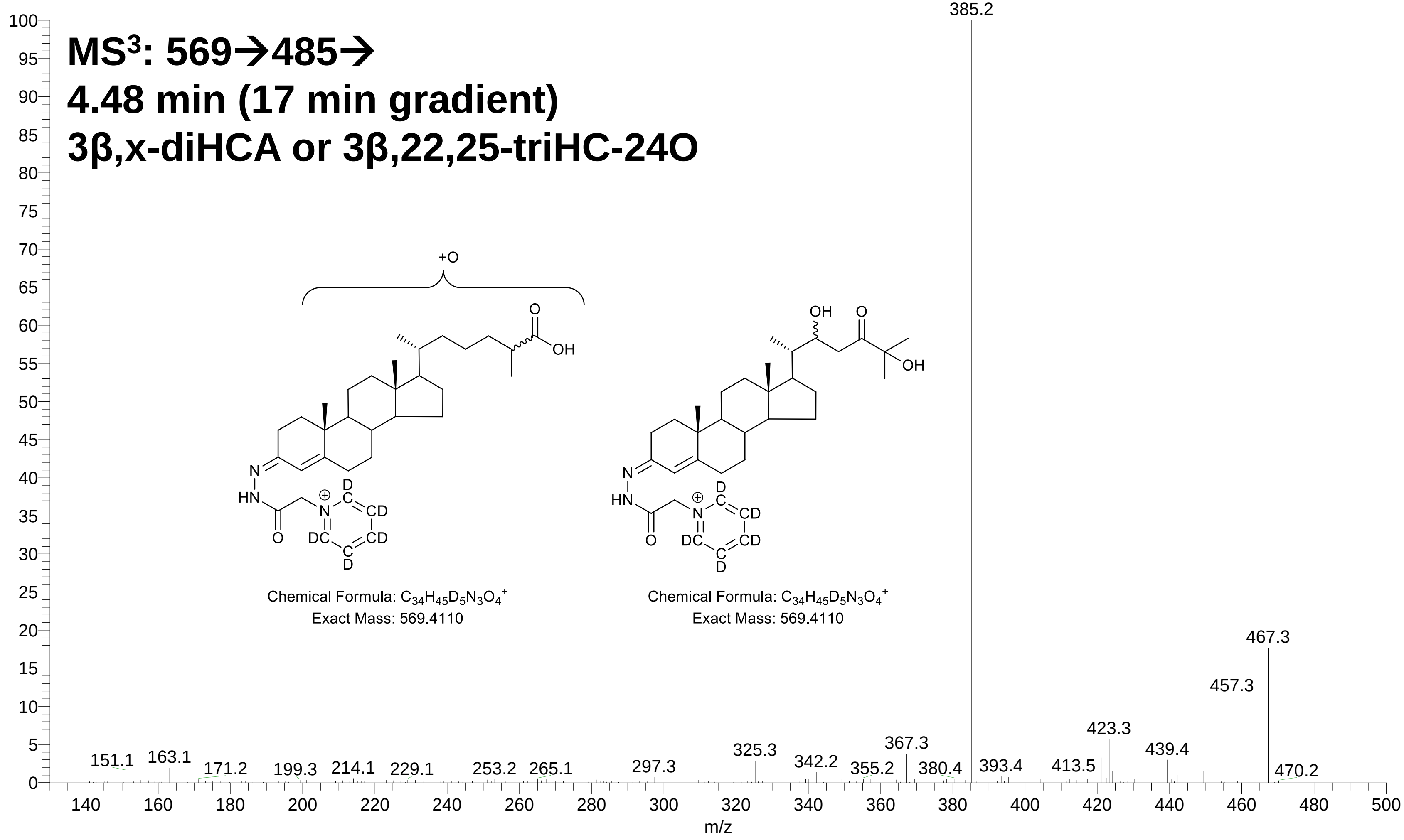

S13E

EY_191014_100uL-NIST-QC_100uL-OxySpla... 10/15/19 04:28:42
100uL NIST (2018) + 100ul OxysterolSPLASH + 10ng 22S-HCO-D7 + 20ug
EY_191014_100uL-NIST-QC_100uL-OxySplash_rep2of5_Fr1A=GPd5_Fr1E 6.90 AV: 1 NL: 4.69E3
F: ITMS + c ESI Full ms3 553.42@cid30.00 469.34@cid35.00 [125.00-560.0

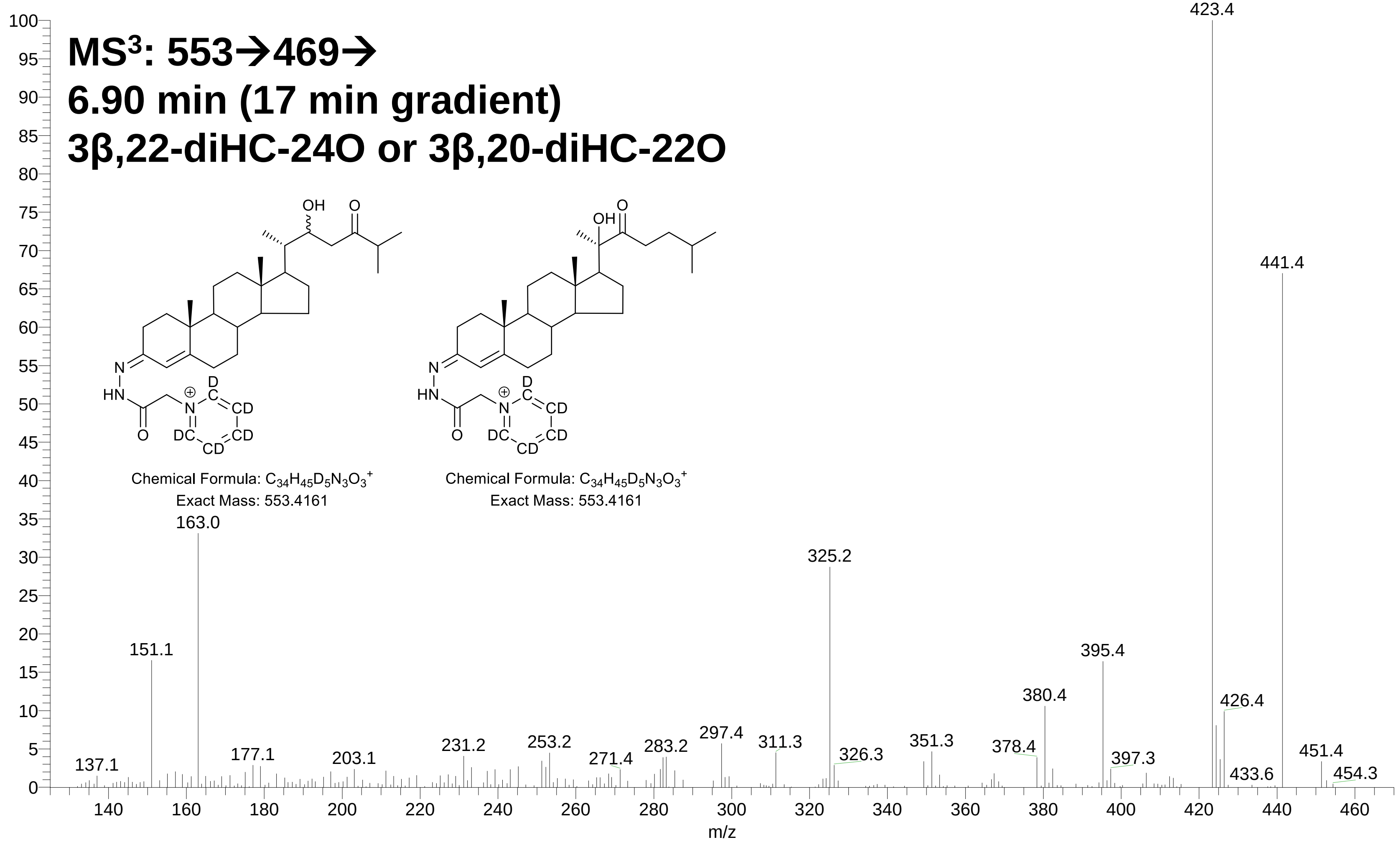

S13F

EY_191014_100uL-NIST-QC_100uL-OxySpla... 10/15/19 04:28:42
100uL NIST (2018) + 100ul OxysterolSPLASH + 10ng 22S-HCO-D7 + 20ug
EY_191014_100uL-NIST-QC_100uL-OxySplash_rep2of5_Fr1A=GPd5_Fr1E .04 AV: 1 NL: 5.27E2
F: ITMS + c ESI Full ms3 585.41@cid30.00 501.33@cid35.00 [135.00-590.(

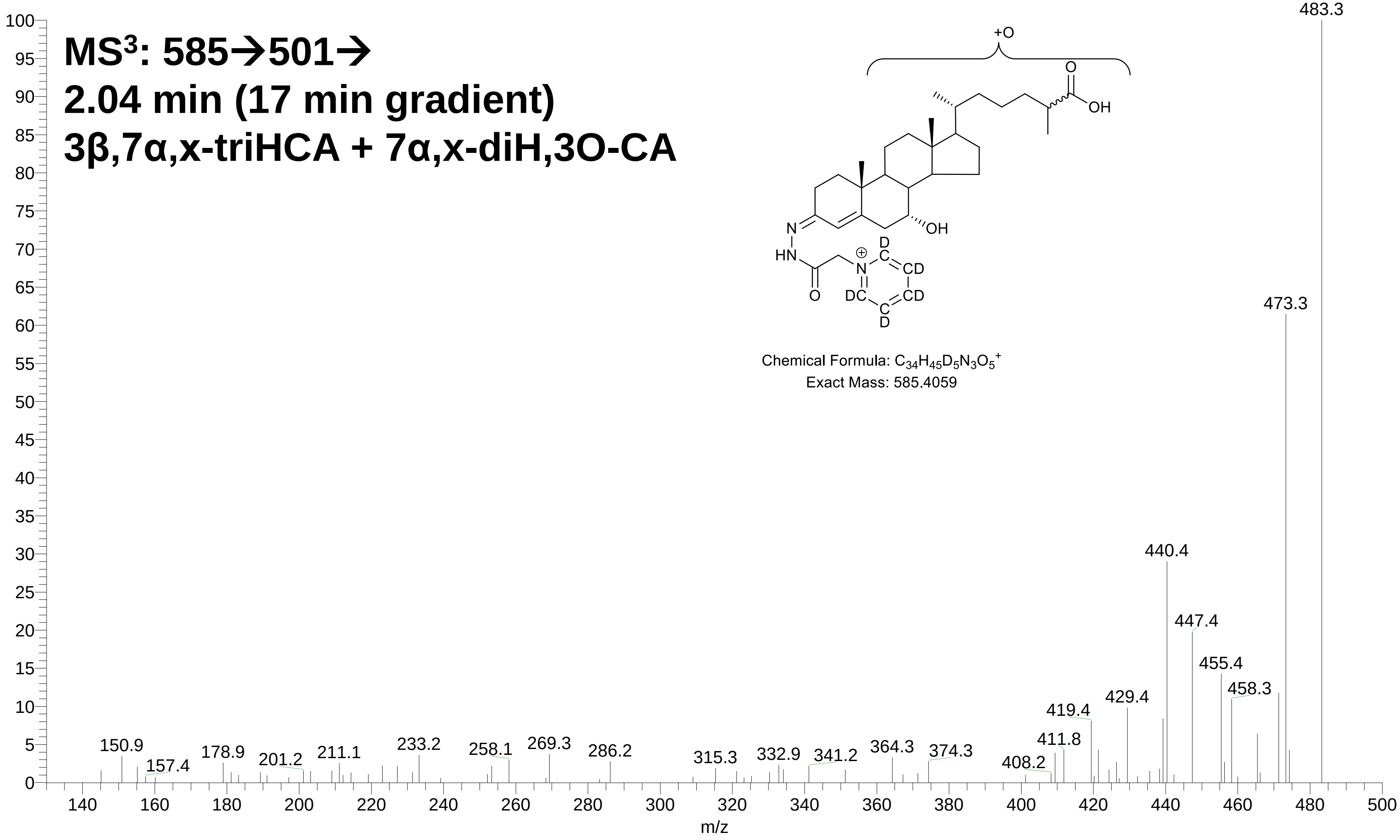

S14A

EY_191029_100uL-Cambridge-CSF-pool_20... 10/29/19 21:38:19
100uL Cambridge Pooled CSF (2019) + 20ul OxysterolSPLASH + 1ng 22S-I rtified)
EY_191029_100uL-Cambridge-CSF-pool_20uL-OxySPLASH_r2_NON-HYD A=GPd5_Fr1B=GPd0_s2_neat_07 #425 RT: 1.87 AV: 1 NL: 3.01E2
F: ITMS + c ESI Full ms3 585.41@cid30.00 501.33@cid35.00 [135.00-590.0

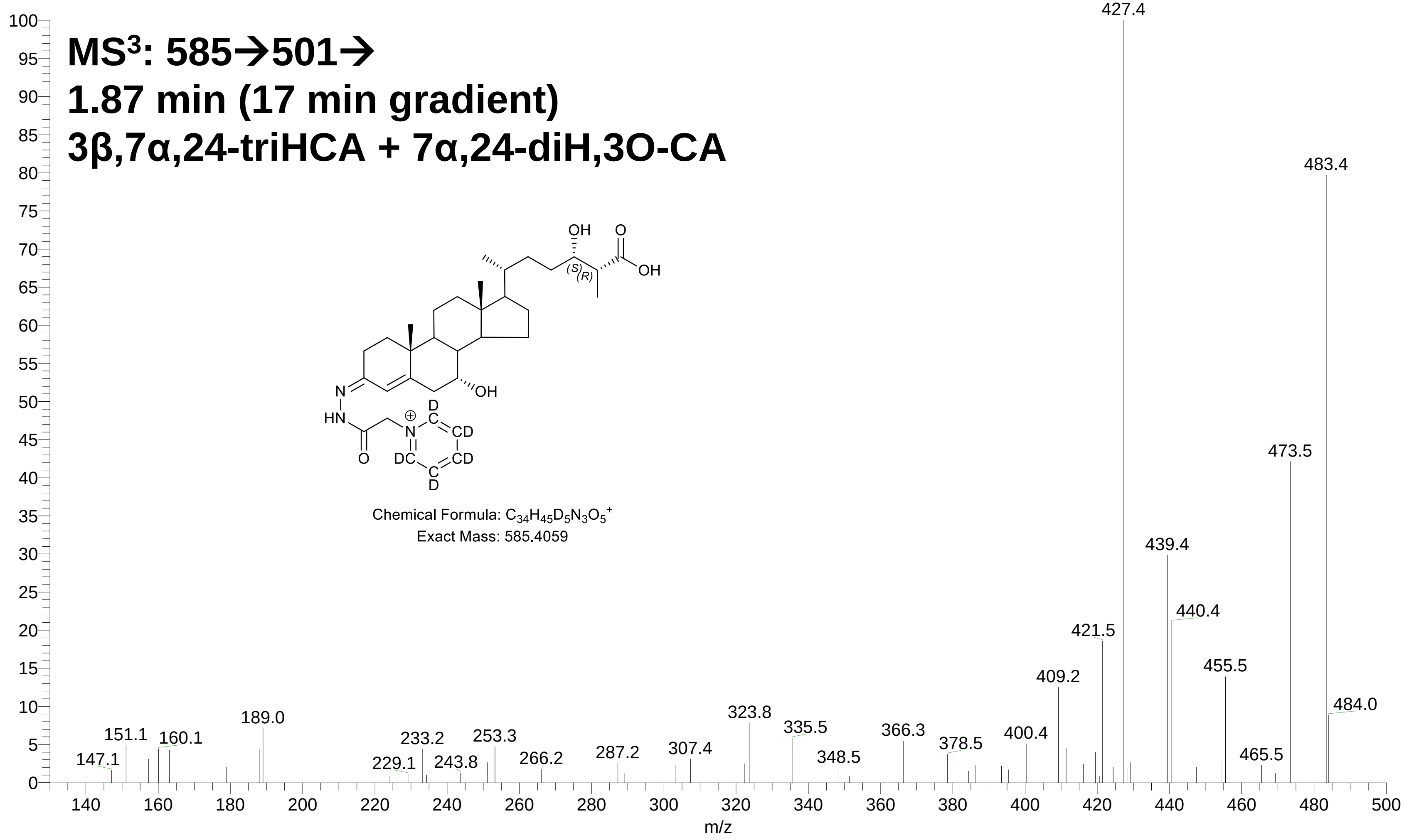

S14B

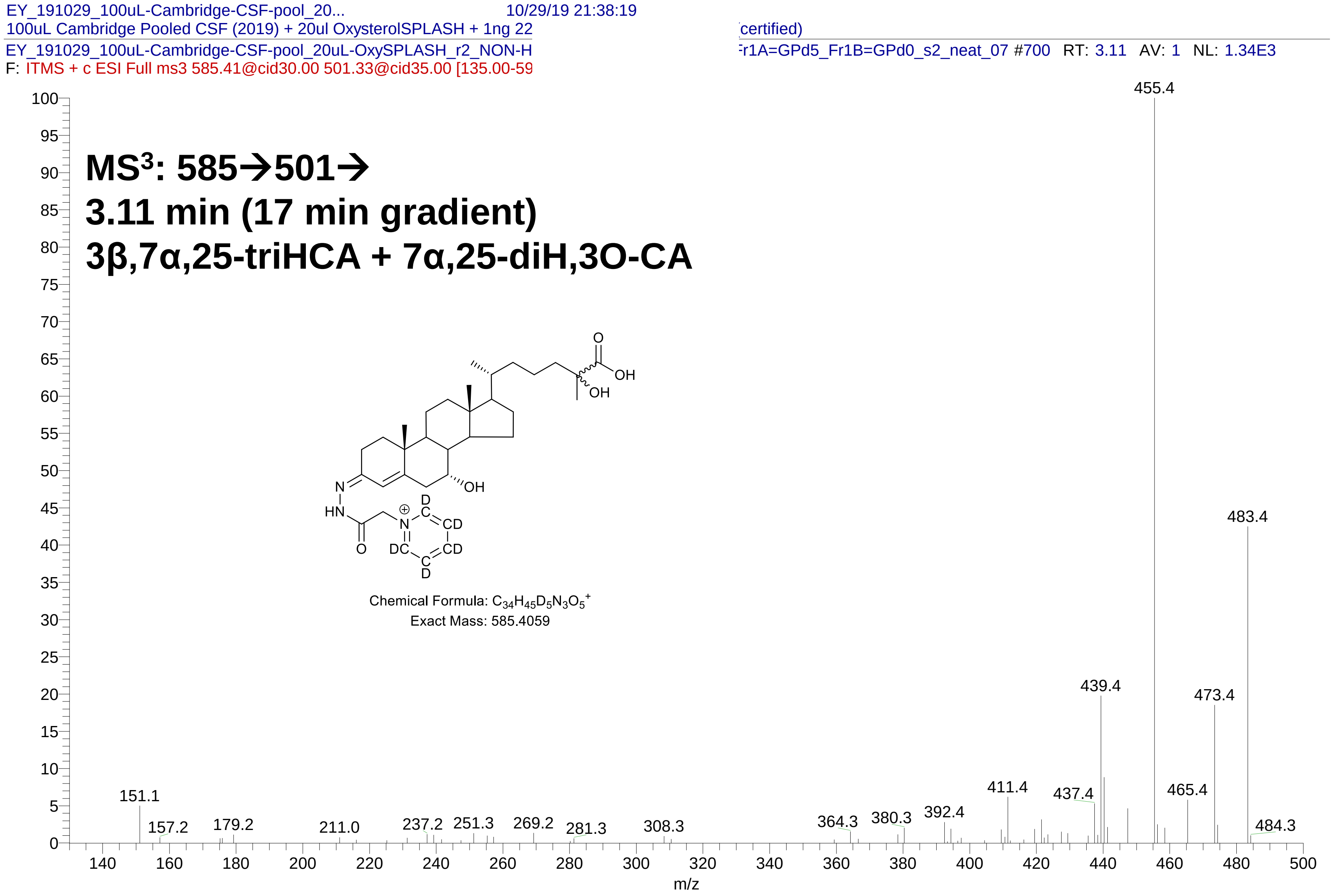
EY_191029_100uL-Cambridge-CSF-pool_20...
10/29/19 21:38:19
100uL Cambridge Pooled CSF (2019) + 20ul OxysterolSPLASH + 1ng 22
certified)
EY_191029_100uL-Cambridge-CSF-pool_20uL-OxySPLASH_r2_NON-H
r1A=GPd5_Fr1B=GPd0_s2_neat_07 #700 RT: 3.11 AV: 1 NL: 1.34E3
F: ITMS + c ESI Full ms3 585.41@cid30.00 501.33@cid35.00 [135.00-59
MS³: 585→501→
3.11 min (17 min gradient)
3β,7α,25-triHCA + 7α,25-diH,3O-CA
Chemical Formula: C34H45D5N3O5+
Exact Mass: 585.4059
455.4
483.4
439.4
473.4
411.4
437.4
465.4
151.1
157.2
179.2
211.0
237.2
251.3
269.2
281.3
308.3
364.3
380.3
392.4
484.3
m/z

S14C

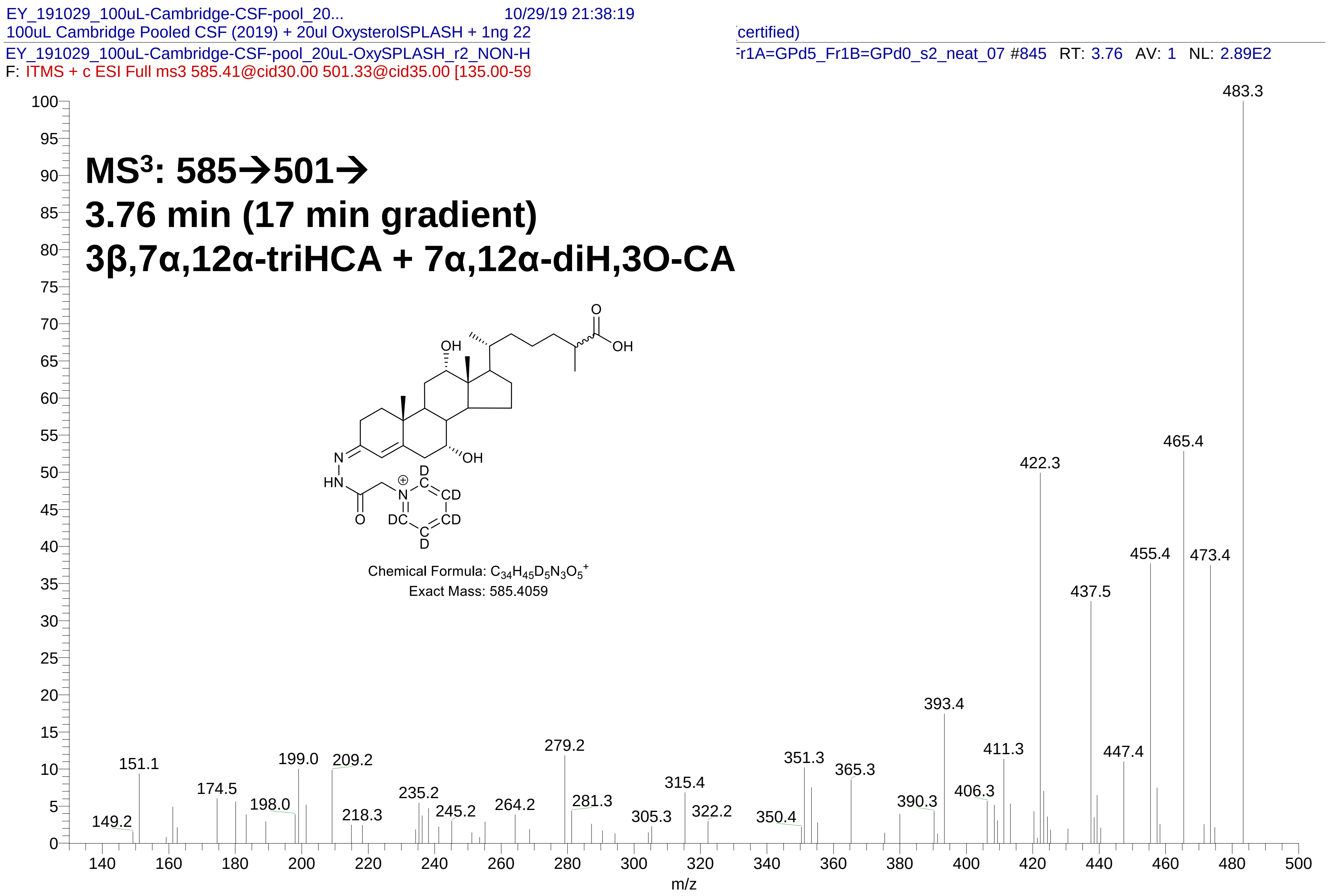

EY_191029_100uL-Cambridge-CSF-pool_20... 10/29/19 21:38:19
100uL Cambridge Pooled CSF (2019) + 20ul OxysterolSPLASH + 1ng 22 certified)
EY_191029_100uL-Cambridge-CSF-pool_20uL-OxySPLASH_r2_NON-H Fr1A=GPd5_Fr1B=GPd0_s2_neat_07 #845 RT: 3.76 AV: 1 NL: 2.89E2
F: ITMS + c ESI Full ms3 585.41@cid30.00 501.33@cid35.00 [135.00-59
MS3: 585→501→
3.76 min (17 min gradient)
3β,7α,12α-triHCA + 7α,12α-diH,3O-CA
Chemical Formula: C34H45D5N3O5+
Exact Mass: 585.4059
483.3
465.4
422.3
455.4
473.4
437.5
393.4
411.3
447.4
406.3
390.3
365.3
351.3
350.4
322.2
315.4
305.3
281.3
279.2
264.2
245.2
235.2
218.3
209.2
199.0
198.0
174.5
151.1
149.2
m/z

S14D

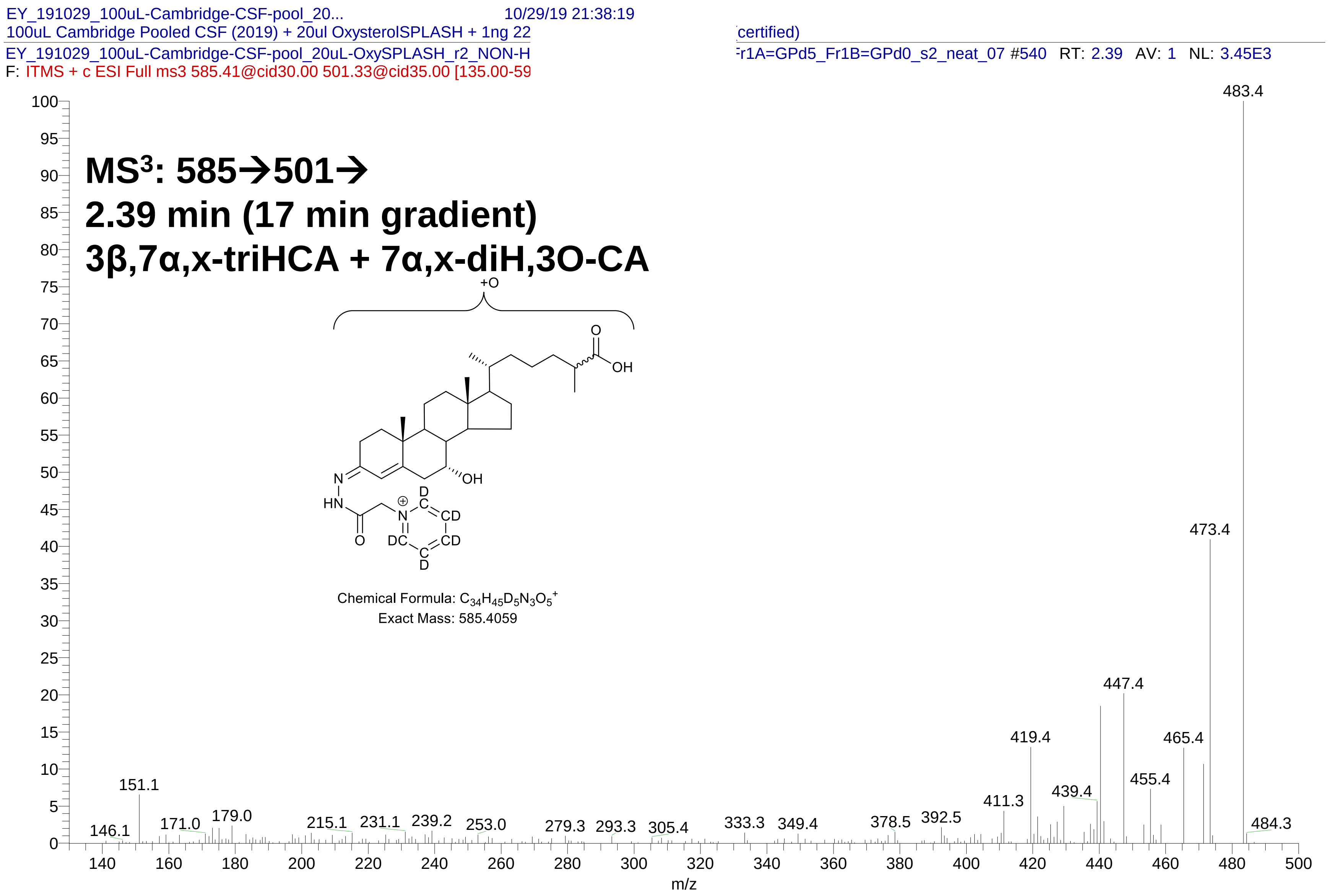

EY_191029_100uL-Cambridge-CSF-pool_20... 10/29/19 21:38:19
100uL Cambridge Pooled CSF (2019) + 20ul OxysterolSPLASH + 1ng 22 certified)
EY_191029_100uL-Cambridge-CSF-pool_20uL-OxySPLASH_r2_NON-H r1A=GPd5_Fr1B=GPd0_s2_neat_07 #540 RT: 2.39 AV: 1 NL: 3.45E3
F: ITMS + c ESI Full ms3 585.41@cid30.00 501.33@cid35.00 [135.00-59
MS³: 585→501→
2.39 min (17 min gradient)
3β,7α,x-triHCA + 7α,x-diH,3O-CA
+O
O
OH
OH
N
HN
O
D
C
CD
CD
DC
C
D
N
Chemical Formula: C34H45D5N3O5+
Exact Mass: 585.4059
483.4
473.4
447.4
419.4
465.4
455.4
439.4
411.3
392.5
378.5
349.4
333.3
305.4
293.3
279.3
253.0
239.2
231.1
215.1
179.0
171.0
151.1
146.1
484.3
m/z
